# Diastereoselective Formal 1,3-Dipolar Cycloaddition of Trifluoroethyl Amine-Derived Ketimines Enables the Desymmetrization of Cyclopentenediones

**DOI:** 10.3390/molecules28145372

**Published:** 2023-07-13

**Authors:** Lin-Qiang Li, Jian-Qiang Zhao, Yan-Ping Zhang, Yong You, Zhen-Hua Wang, Zhen-Zhen Ge, Ming-Qiang Zhou, Wei-Cheng Yuan

**Affiliations:** 1 Innovation Research Center of Chiral Drugs, Institute for Advanced Study, Chengdu University, Chengdu 610106, China; 2 School of Pharmacy, Zunyi Medical University, Zunyi 563000, China; 3 National Engineering Research Center of Chiral Drugs, Chengdu Institute of Organic Chemistry, Chinese Academy of Sciences, Chengdu 610041, China; zhenzhen.ge@foxmail.com

**Keywords:** desymmetrization, 1,3-dipolar cycloaddition, cyclopentene-1,3-diones, trifluoroethyl amine-derived ketimines, polycyclic heterocycles

## Abstract

In this research, a metal-free diastereoselective formal 1,3-dipolar cycloaddition of *N*-2,2,2-trifluoroethylisatin ketimines and cyclopentene-1,3-diones which can efficiently lead to the desymmetrization of cyclopentene-1,3-diones is developed. With the developed protocol, a series of tetracyclic spirooxindoles containing pyrrolidine and cyclopentane subunits can be smoothly obtained with good results (up to 99% yield and 91:9 dr). Furthermore, the methodology can be extended to trifluoromethyl-substituted iminomalonate, and the corresponding formal [3+2] cycloaddition reaction affords bicyclic heterocycles containing fused pyrrolidine and cyclopentane moieties in moderate yields with >20:1 dr. The synthetic potential of the methodology is demonstrated by the scale-up experiment and by versatile transformations of the products.

## 1. Introduction

**Figure 1:**
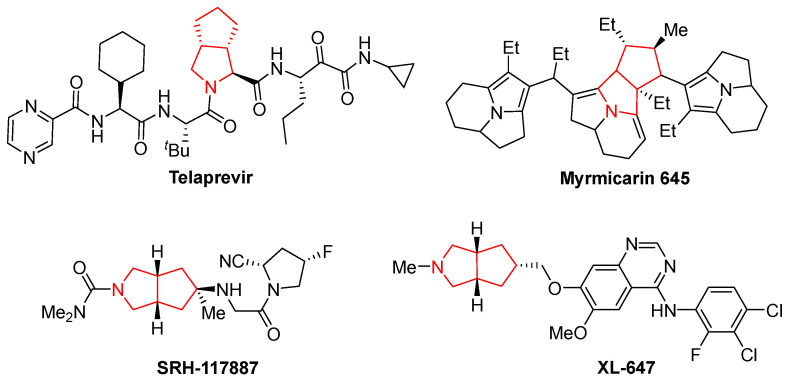
Representative bioactive molecules containing fused pyrrolidine and cyclopentane moieties.

Desymmetric transformation of symmetrical molecules has long been considered one of the most powerful strategies for the construction of structurally diverse complex molecules with multiple stereogenic centers in organic synthesis, such as enzymatic desymmetrizations [1], organocatalytic desymmetrizations [2], and transition metal catalyzed desymmetrizations [3,4]. In addition, reviews on desymmetrization reactions about meso-diols [5], cyclohexanones [6,7], and cyclobutanones [8] are available. As a consequence, a wide variety of molecules possessing symmetries have been exploited for diverse desymmetrization reactions during the past several decades. For example, the desymmetrization of meso-cyclic ketones and kinetic resolution of racemic 2-arylcyclohexanones though Baeyer–Villiger oxidation was reported by the Feng group in 2012 [9]. The desymmetrization of cyclohexanones and their derivatives has been reported by several groups [10,11,12,13]. In 2013, Cu(I)-catalyzed azide–alkyne cycloaddition via asymmetric desymmetrization of oxindole-based 1,6-heptadiynes was reported by Zhou and coworkers [14]. The Cai group described copper-catalyzed intramolecular desymmetric aryl C-O coupling for the enantioselective construction of chiral dihydrobenzofurans and dihydrobenzopyrans [15]. The NHC-catalyzed desymmetrization of bisphenols to P-stereogenic phosphinates was reported by Chi and coworkers in 2015 [16]. Later, an iridium-catalyzed desymmetrization reaction involving allylic dearomatization of indoles was developed [17]. The desymmetrization of a centrosymmetric *pseudo-para*-diformyl[2.2]paracyclophane was realized by the group of Micouin [18]. In 2021, an unprecedented desymmetrization of meso-1,3-diones by enantioselective intermolecular condensation was presented by Zhao and coworkers [19]. Recently, the desymmetrization of oxetanes and silanediols have been respectively reported in [20,21]. Prochiral cyclopentene-1,3-diones, as readily available, inexpensive, and air-stable compounds, have proven to be ideal electrophiles in reactions with various nucleophiles via a desymmetrization process for the synthesis of highly functionalized 1,3-cyclopentanedione via the Michael addition process [22,23,24,25]. As an alternative, desymmetrization of cyclopentenediones via [3+2] or [4+2] cycloaddition has been reported for the synthesis of 1,3-cyclopentanediones [26,27,28,29]. However, an extensive literature search revealed that the related 1,3-dipolar cycloaddition reaction with cyclopentene-1,3-diones as electrophilic dipolarophiles has been little explored. In 2015, the Wang and Singh groups independently reported Ag(I)-catalyzed desymmetrization of prochiral cyclopentenediones via formal 1,3-dipolar cycloaddition with azomethine-ylide for the synthesis of highly functionalized 5,5-fused bicyclic pyrrolidine derivatives [30,31]. Later, using DIPEA as the base, the diastereoselective 1,3-dipolar cycloaddition reaction of cyclopentene-1,3-diones and *N*-phenacylbenzothiazolium bromides for the synthesis of tetracyclic products bearing five stereogenic centers was described by the group of Pan [32]. In 2018, an efficient kinetic resolution of readily available racemic cyclopentene-1,3-diones was developed via Ag(I)-catalyzed asymmetric 1,3-dipolar cycloaddition of azomethine ylides [33]. Notably, these 1,3-dipolar cycloaddition reactions of cyclopentenediones and azomethine-ylides could provide an efficient approach to accessing bicyclic heterocycles containing fused pyrrolidine and cyclopentane moieties. These are well-known structural cores of many biologically active natural products and pharmaceuticals, such as the HCV NS3-4A protease inhibitors Telaprevir [34] and SRH-117887 [35,36], the alkaloid Myrmicarin 645 [37], and the tyrosine kinase inhibitor XL-647 [38] (Figure 1). Considering the outstanding potential of such bicyclic heterocycles for drug research and development, it remains attractive to explore efficient and convenient means of synthesizing structurally diverse fused pyrrolidine–cyclopentane skeletons from readily available starting materials.

**Scheme 1:**
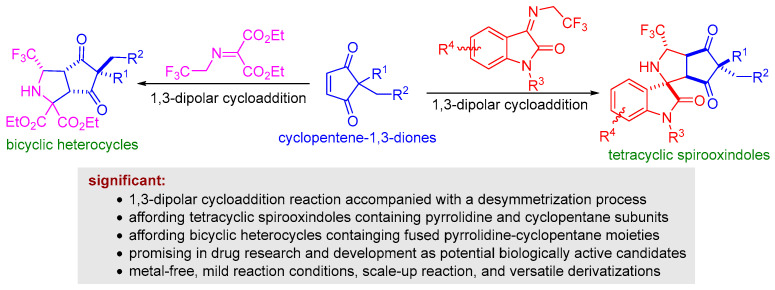
1,3-Dipolar cycloaddition reaction of trifluoroethyl amine-derived ketimines enabling the desymmetrization of cyclopentene-1,3-diones.

*N*-2,2,2-trifluoroethylisatin ketimines are one class of active azomethine-ylide precursors that are readily prepared from isatins and trifluoroethylamine by condensation reaction [39]. In recent years, a wide variety of 1,3-dipolar cycloadditions have been developed by using *N*-2,2,2-trifluoroethylisatin ketimines reacted with various dipolarophiles to synthesize CF_3_-containing spirocyclic oxindoles bearing multiple stereogenic centers [40,41]. As a continuation of our research interest in 1,3-dipolar cycloaddition for synthesis of polyheterocycles [42,43,44], we envisaged that a series of highly functional polyheterocycles fused by CF_3_-containing spirocyclic oxindole, pyrrolidine, and cyclopentane substructures could be assembled via the formal 1,3-dipolar cycloaddition reaction of *N*-2,2,2-trifluoroethylisatin ketimines and cyclopentene-1,3-diones under suitable conditions, and that this reaction would be capable of causing the desymmetrization transformation of cyclopentene-1,3-diones. (Scheme 1). Furthermore, we found that the 1,3-dipolar cycloaddition of trifluoromethyl-substituted iminomalonate and cyclopentene-1,3-diones is feasible through a desymmetrization process, leading to the formation of bicyclic heterocycles containing fused pyrrolidine-cyclopentane moieties (Scheme 1). The resulting novel tetracyclic spirooxindoles and bicyclic heterocycles were characterized by melting point, NMR, and HRMS. Herein, we report our latest results on this subject.

## 2. Results and Discussion

### 2.1. Optimization Studies

Initially, we selected the *N*-2,2,2-trifluoroethylisatin ketimine **1a** and cyclopentene-1,3-dione **2a** as model substrates for the optimization of reaction conditions. As shown in Table 1, to our delight, when using 1,1,3,3-tetramethylguanidine (TMG) as the base the reaction could proceed well in 1,2-dicholorethane (DCE) at 30 °C, providing the tetracyclic spirooxindole containing pyrrolidine and cyclopentane subunits **3aa** in 73% yield with 81:19 dr (Table 1, entry 1). By screening different organic bases (Table 1, entry 2–4) such as 1,8-diazabicyclo[5.4.0]undec-7-ene (DBU), 1,5-diazabicyclo[4.3.0]non-5-ene (DBN), and 1,5,7-triazabicyclo[4.4.0]dec-5-ene (TBD), we found that DBU was the best candidate and that the corresponding cycloaddition product **3aa** could be obtained in 87% yield with good diastereoselectivity (Table 1, entry 2). In the presence of the inorganic base CS_2_CO_3_, the reaction took place but yielded the cycloaddition product **3aa** with poor results (only 30% yield and 71:29 dr) after 96 h (Table 1, entry 5). Next, different solvents, including toluene, THF, MeCN, and MeOH, were investigated, and it was found that DCE was the optimal solvent in terms of yield and diastereoselectivity (Table 1, entry 2 vs. entries 6–10). We were pleased to find that changing the base from DBU to 1,4-diazabicyclo[2.2.2]octane (DABCO) in DCE could lead to better results (90% yield and 84:16 dr), despite increasing the reaction time to 48 h (Table 1, entry 12). Raising the reaction temperature to 50 °C delivered product **3aa** in 90% yield and 84:16 dr with a reduction of the reaction time to 24 h (Table 1, entry 13). By altering the ratio of substrates **1a**:**2a** from 1.2:1 to 1.5:1, the isolated yield of product **3aa** could be slightly improved to 92% (Table 1, entry 14). Finally, the product **3aa** was obtained in 96% yield with 85:15 dr at 50 °C for 24 h by increasing the amount of DABCO from 1.0 equivalent to 1.5 equivalents (Table 1, entry 15).

### 2.2. Substrate Scope Studies

**Scheme 2:**
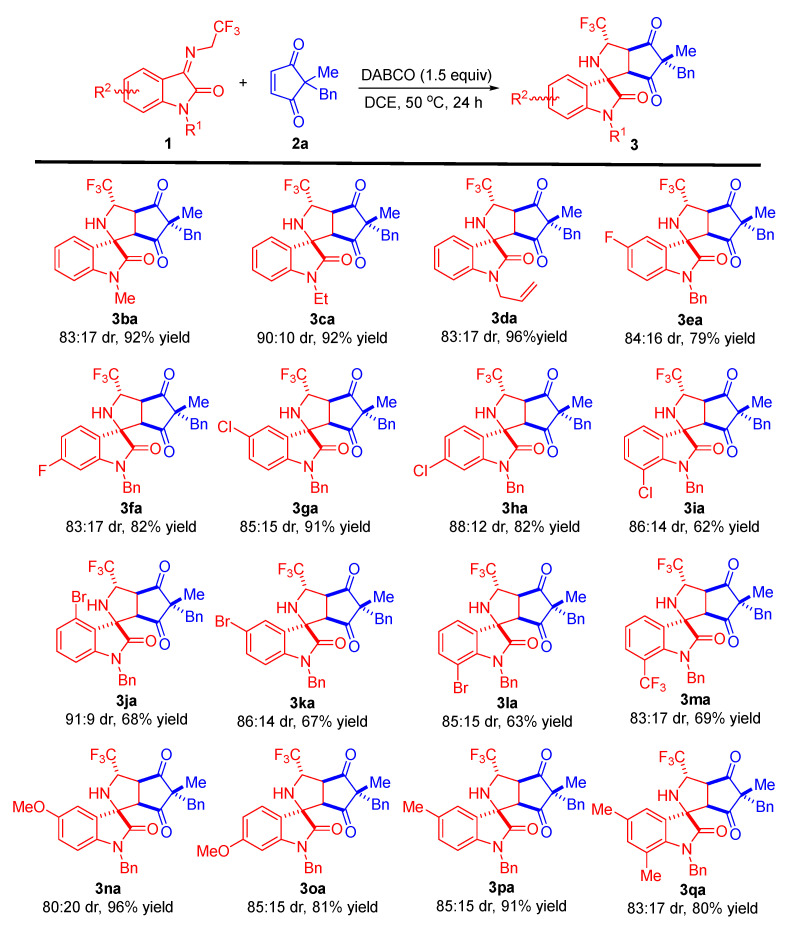
Substrate scope investigation of the metal-free diastereoselective formal 1,3-dipolar cycloaddition of different *N*-2,2,2-trifluoroethylisatin ketimines **1** and cyclopentene-1,3-dione **2a**. Reaction conditions: the reactions were carried out with **1** (0.15 mmol), **2a** (0.1 mmol), and 1.5 equivalents of DABCO in 1.0 mL of DCE at 50 °C for 24 h. The dr values were determined by ^1^H NMR analysis of the crude product. The yields refer to isolated yields as a mixture of diastereoisomers.

With the optimized reaction conditions in hand, we set out to investigate the substrate scope and generality of the metal-free diastereoselective formal 1,3-dipolar cycloaddition of different *N*-2,2,2-trifluoroethylisatin ketimines **1** and various cyclopentene-1,3-diones **2**. First, the substrate scope of *N*-2,2,2-trifluoroethylisatin ketimines was explored; the results are shown in Scheme 2. The reaction system was applicable to different substituents (Me-, Et-, and allyl-) at the *N*1 position of the isatin-derived ketimines. These substrates were able to react smoothly with cyclopentene-1,3-dione **2a** to afford the corresponding tetracyclic spirooxindoles **3ba**–**da** in high yields and good diastereoselectivities (up to 96% yield and 90:10 dr). Furthermore, the introduction of an electron-withdrawing group, such as F-, Cl-, or Br-, into the oxindole skeleton of ketimines allowed these substrates to react effectively with **2a** regardless of their different positions, resulting in the corresponding products **3ea**–**la** in yields ranging from 62–91% with acceptable dr values. Moreover, the substrate with trifluoromethyl at the C7-position of the *N*-2,2,2-trifluoroethylisatin ketimine provided cycloaddition product **3ma** in 69% yield with 83:17 dr. In addition, a variety of isatin-derived ketimines with electron-donating groups (-OMe and -Me) worked well, affording the corresponding products **3na**–**pa** with good results (91–96% yields and 80:20–85:15 dr). Disubstituted substrate, such as 5,7-dimethyl-substituted trifluoroethylisatin ketimine, was successfully employed in this transformation under the standard conditions, generating product **3qa** in 80% yield with 83:17 dr.

Next, a range of differently substituted cyclopentenediones **2** were examined by reaction with *N*-2,2,2-trifluoroethylisatin ketimine **1a** to further explore the substrate scope of the 1,3-dipolar cycloaddition reaction (Table 2). With electron-donating groups such as methyl and methoxy on the phenyl moiety of 2-benzyl-2-methylcyclopentenediones **2**, the reactions afforded cycloaddition products **3ab**–**ad** in almost quantitative yields (94–96%), with good diastereoselectivities (up to 85:15 dr) in the reaction with *N*-2,2,2-trifluoroethylisatin ketimine **1a** (Table 2, entries 1–3). Similarly, the reaction system showed good compatibility with cyclopentenediones with different electron-withdrawing groups (such as F-, Cl- and Br-) on the phenyl moiety, as demonstrated by the synthesis of cycloaddition products **3ae**–**ai** in good to excellent yields and high diastereoselectivities (Table 2, entries 4–8). Meanwhile, when the aromatic ring of the benzyl group of **2** was substituted with bulky naphthyl groups, the reactions furnished the corresponding tetracyclic spirooxindoles **3aj** and **3ak** in 98% yield with 89:11 dr and 99% yield with 86:14 dr, respectively (Table 2, entries 9 and 10). In addition, the change of the R^2^ group of cyclopentenediones from methyl to ethyl was tolerated by the reaction system, providing efficient access to products **3al**–**an** with acceptable results (up to 88% yield and 88:12 dr) (Table 2, entries 11–13).

**Scheme 3:**
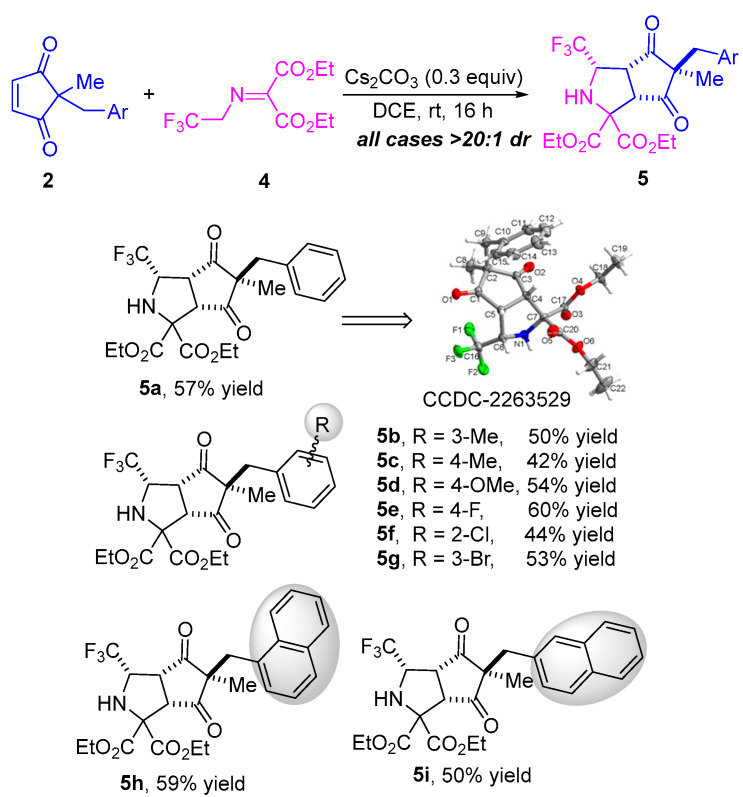
Substrate scope investigation of 1,3-dipolar cycloaddition of trifluoromethyl-substituted iminomalonate and different cyclopentenediones. The dr values were determined by ^1^H NMR analysis of the crude product. The yields refer to isolated yields as a mixture of diastereoisomers.

Having established the general scope of the metal-free diastereoselective formal 1,3-dipolar cycloaddition of *N*-2,2,2-trifluoroethylisatin ketimines **1** and diverse cyclopentene-1,3-diones **2,** we next attempted the formal 1,3-dipolar cycloaddition of trifluoromethyl-substituted iminomalonate **4** and cyclopentenediones **2** through a desymmetrization strategy. As illustrated in Scheme 3, using Cs_2_CO_3_ as a catalyst in DCE at room temperature for 16 h, the reaction of 2-benzyl-2-methylcyclopentenedione **2a** and trifluoromethyl-substituted iminomalonate **4** proceeded well, providing the desired bicyclic heterocyclic product **5a** in 57% yield with >20:1 dr. The generality of this transformation of various cyclopentene-1,3-diones **2** and trifluoromethyl-substituted iminomalonate **4** was then investigated, and it was found that all reactions resulted in the corresponding cyclization products with excellent diastereoselectivities (all cases > 20:1 dr) (Scheme 3). As cyclopentenediones bearing different electron-donating substituents such as methyl and methoxyl on the phenyl, these substrates worked well in the reaction with trifluoromethyl-substituted iminomalonate **4** under the standard conditions, providing the desired cycloaddition products **5b**–**d** in moderate yields (42–54% yield). Likewise, cyclopentenediones with various electron-withdrawing substituents (F-, Cl, and Br-) on the phenyl ring, regardless of the position, were compatible with the catalytic system in the reaction with **4**, affording the desired cycloaddition products **5e**–**g** in yields ranging from 44–60%. Cyclopentenedione substrates bearing a sterically hindered naphthyl group proceeded successfully as well, yielding the corresponding bicyclic heterocycle products **5h** and **5i** in 59% and 50% yield, respectively. The structure and the relative configuration of product **5a** was unambiguously determined by single-crystal X-ray analysis, and the configurations of all other products in Scheme 3 were assigned by analogy due to their formation through a common reaction pathway [45].

### 2.3. Scale-Up Experiment and Versatile Transformations of the Products

**Scheme 4:**
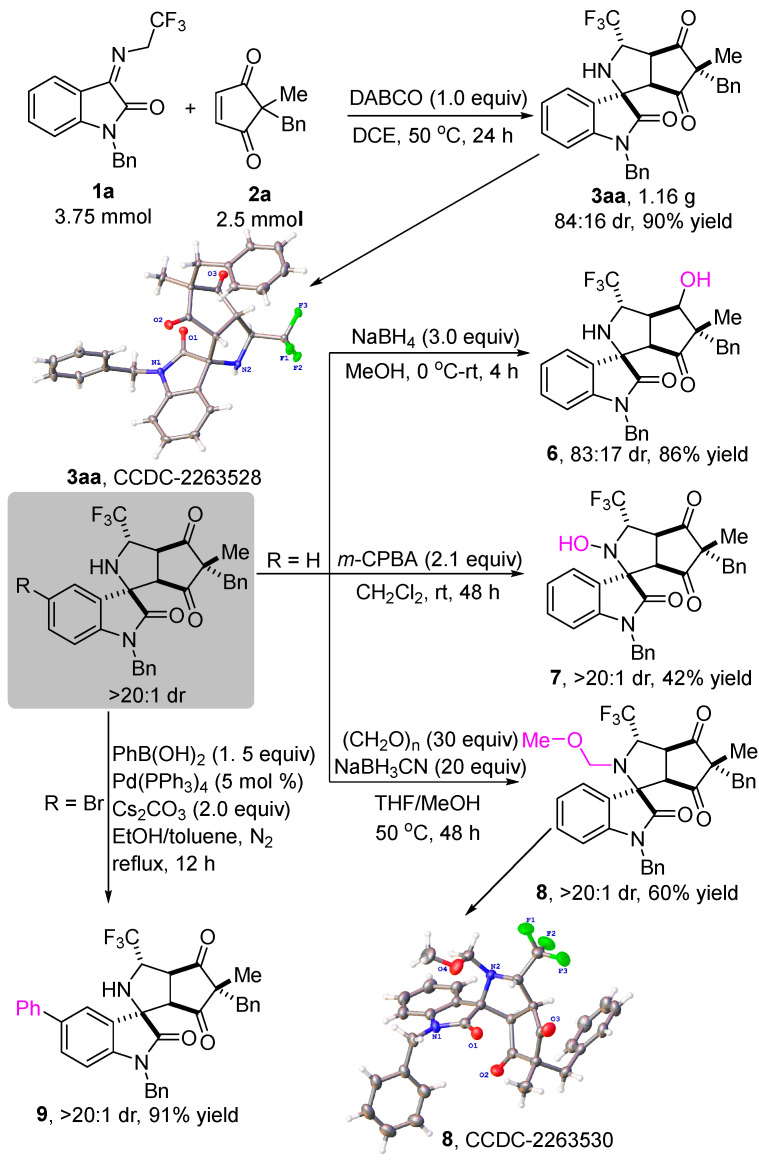
Scale-up experiment and different transformations of products.

In order to demonstrate the synthetic potential and utility of the metal-free diastereoselective formal 1,3-dipolar cycloaddition of *N*-2,2,2-trifluoroethylisatin ketimines and cyclopentene-1,3-diones, a gram-scale experiment between **1a** and **2a** was carried out under standard reaction conditions, which was 25-fold larger than the scale of the model reaction. As shown in Scheme 4, the reaction was able to proceed to completion at 50 °C after 24 h in DCE, smoothly providing product **3aa** in 90% yield with 84:16 dr. The structure and the relative configuration of the product **3aa** were unambiguously determined by single-crystal X-ray analysis. Assuming a common reaction pathway, the relative configuration of the other products in Scheme 2 and Table 2 was assigned by analogy [45]. Next, various transformations of the products to other heterocyclic compounds were investigated. When **3aa** was treated with NaBH_4_ in MeOH for 4 h, the reduction product **6** was obtained in 86% yield with 83:17 dr. The structure and relative configuration of product **6** were determined with certainty by NMR and NOESY spectra (see Supporting Information). The treatment of **3aa** with *m*-CPBA in CH_2_Cl_2_ for 48 h gave rise to the formation of the hydroxylamine product **7** in 42% yield with >20:1 dr. In addition, product **8** was obtained with acceptable results from **3aa** with (CH_2_O)_n_ and NaBH_3_CN in the mixed solvents of MeOH and THF at 50 °C for 48 h. Notably, the structure of product **8** was unambiguously determined by single-crystal X-ray analysis [45]. Ultimately, the Suzuki coupling reaction between **3ka** and phenylboronic acid was conducted with 5 mol % Pd(PPh_3_)_4_ as the catalyst to afford the product **9** in 91% yield with >20:1 dr.

### 2.4. Proposed Mechanism for the 1,3-Dipolar Cycloaddition Reaction Accompanied by Desymmetrization Process

**Scheme 5:**
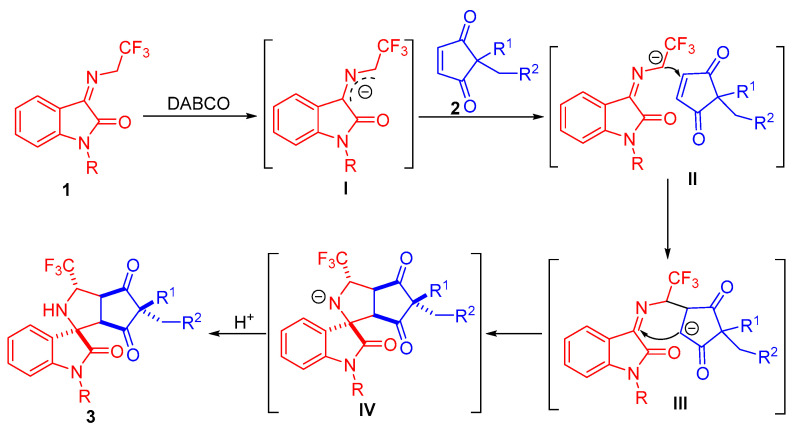
Proposed mechanism for 1,3-dipolar cycloaddition of *N*-2,2,2-trifluoroethylisatin ketimines and cyclopentene-1,3-diones.

Based on our experimental results and previous related reports [30,31,32,33], a plausible catalytic mechanism involving a stepwise reaction process was assumed to explain the stereoselectivity of the 1,3-dipolar cycloaddition of *N*-2,2,2-trifluoroethylisatin ketimines and cyclopentene-1,3-diones. As depicted in Scheme 5, the *N*-2,2,2-trifluoroethylisatin ketimine **1** is deprotonated under a basic condition to yield the 2-azaallyl anion intermediate **I**. As illustrated in transition state **II**, the *α*-carbon anion of intermediate **I** approaches the carbon–carbon double bond of cyclopentene-1,3-diones, resulting in addition of the intermediate **III**. Subsequently, intramolecular cyclization from the carbon anion to the C=N of azomethine-ylide occurs, delivering the intermediate IV, which is protonated to form products **3**.

## 3. Materials and Methods

### 3.1. General Information

Reagents were purchased from commercial sources and were used as received unless mentioned otherwise. Reactions were monitored by thin-layer chromatography (TLC). ^1^H NMR and ^13^C NMR spectra were recorded in CDCl_3_ and DMSO-*d*_6_. ^1^H NMR chemical shifts are reported in ppm relative to tetramethylsilane (TMS), with the solvent resonance employed as the internal standard (CDCl_3_ at 7.26 ppm, DMSO-*d*_6_ at 2.50 ppm). Data are reported as follows: chemical shift, multiplicity (s = singlet, br s = broad singlet, d = doublet, t = triplet, q = quartet, m = multiplet), coupling constants (Hz), and integration. ^13^C NMR chemical shifts are reported in ppm from tetramethylsilane (TMS), with the solvent resonance as the internal standard (CDCl_3_ at 77.20 ppm, DMSO-*d*_6_ at 39.52 ppm). Melting points of the products were recorded on a Büchi Melting Point B-545. The HRMS were recorded by The HRMS were recorded using an Agilent 6545 LC/Q-TOF mass spectrometer.

### 3.2. General Experimental Procedure for the 1,3-Dipolar Cycloaddition Reaction of N-2,2,2-Trifluoroethylisatin Ketimines and Cyclopentene-1,3-Diones for the Synthesis of Compounds ***3*** (Scheme 2 and Table 2)

*N*-2,2,2-trifluoroethylisatin ketimines **1** (0.3 mmol) and DABCO (33.6 mg, 0.3 mmol, 1.5 equiv.) were added to a solution of cyclopentene-1,3-diones **2** (40.2 mg, 0.2 mmol, 1.0 equiv.) in DCE (2.0 mL), then the mixture was stirred for 24 h at 50 °C. After completion, the reaction mixture was directly purified by flash chromatography on silica gel (petroleum ether/ethyl acetate, 15:1–10:1) to yield the corresponding products **3**.

**3aa**, white solid; 99.5 mg, 96% yield; 85:15 *dr*, mp 194.4–195.3 °C; ^1^H NMR (400 MHz, DMSO-*d*_6_) *δ* 7.38 (d, *J* = 7.3 Hz, 1H), 7.35–7.19 (m, 9H), 7.11 (m, 1H), 7.07–7.01 (m, 2H), 6.74 (d, *J* = 7.8 Hz, 1H), 4.85 (d, *J* = 15.9 Hz, 1H), 4.66 (d, *J* = 5.6 Hz, 1H), 4.59 (d, *J* = 15.9 Hz, 1H), 4.44 (m, 1H), 4.04 (d, *J* = 3.1 Hz, 2H), 3.01 (d, *J* = 13.3 Hz, 1H), 2.85 (d, *J* = 13.3 Hz, 1H), 0.95 (s, 3H). ^13^C{^1^H} NMR (101 MHz, DMSO-*d*_6_) δ 212.8, 210.8, 178.0, 143.1, 135.5, 134.5, 130.2, 129.6, 128.6, 128.4, 128.08, 127.4, 127.2, 127.1, 125.6 (q, *J* = 276.8 Hz, 1C), 124.1, 123.1, 109.7, 70.9, 62.0, 58.7 (q, *J* = 30.4 Hz, 1C), 57.8, 51.3, 42.7, 41.4, 14.3. HRMS (ESI) *m*/*z*: [M+H]^+^ calcd. for C_30_H_26_F_3_N_2_O_3_, 519.1890, found: 519.1895

### 3.3. General Experimental Procedure for the 1,3-Dipolar Cycloaddition Reaction of Trifluoromethyl-Substituted Iminomalonate and Cyclopentene-1,3-Diones for the Synthesis of Compounds ***5*** (Scheme 3)

First, 2-((2,2,2-trifluoroethyl)imino) malonate **4** (0.1 mmol) and Cs_2_CO_3_ (31.8 mg, 0.09 mmol) were added to a solution of cyclopentene-1,3-diones **2** (60.2mg, 0.3 mmol, 3.0 equiv.) in DCE (3.0 mL). Then, the mixture was stirred at room temperature for 24 h. After completion, the reaction mixture was directly purified by flash chromatography on silica gel (petroleum ether/ethyl acetate = 15:1–10:1) to yield the corresponding products **5**.

**5a**, white solid; 77.8 mg, 57% yield; >20:1 *dr*, mp 162.1–162.9 °C; ^1^H NMR (400 MHz, CDCl_3_) δ 7.25–7.12 (m, 3H), 6.98–6.86 (m, 2H), 4.26 (q, 2H), 4.21–4.06 (m, 2H), 3.63–3.50 (m, 1H), 3.08 (d, *J* = 10.1 Hz, 1H), 2.99 (d, *J* = 12.9 Hz, 1H), 2.86 (t, *J* = 9.7 Hz, 1H), 2.83–2.76 (m, 2H), 1.26 (t, *J* = 7.2 Hz, 3H), 1.20–1.12 (m, 6H). ^13^C{^1^H} NMR (101 MHz, CDCl_3_) δ 213.6, 212.9, 168.7, 166.2, 135.3, 129.6, 128.9 127.5, 123.6 (q, *J* = 280.5 Hz, 1C), 74.7, 63.0, 62.4, 61.4 (q, *J* = 32.6 Hz, 1C), 60.7, 56.2, 49.8, 44.2, 19.5, 13.9, 13.8. HRMS (ESI) *m*/*z*: [M+H]^+^ calcd. for C_22_H_25_F_3_NO_6_, 456.1628, found: 456.1635.

### 3.4. Procedure for the Scale-Up Experiment

*N*-2,2,2-trifluoroethylisatin ketimines **1a** (1.2 g, 3.75 mmol, 1.5 equiv.) and DABCO (0.4 g, 3.75 mmol,1.5 equiv.) were added to a solution of cyclopentene-1,3-diones **2a** (0.5 g, 2.5 mmol, 1.0 equiv.) in DCE (25.0 mL), then the mixture was stirred for 24 h at 50 °C. After completion, the reaction mixture was directly purified by flash chromatography on silica gel (petroleum ether/ethyl acetate = 15:1) to yield the corresponding product **3aa**.

### 3.5. Procedure for the Synthesis of Compound ***6***

Compound **3aa** (0.1 g, 0.2 mmol) was dissolved in MeOH (2.0 mL), then NaBH_4_ (22.7 mg, 3.0 equiv.) was added slowly at 0 °C. The reaction mixture was allowed to stir at room temperature for 4 h. The reaction system was monitored by TLC until **3aa** disappeared completely. After that, the reaction mixture was quenched by a drop of water and directly purified by column chromatography on silica gel (petroleum ether/ethyl acetate = 15:1) to provide compound **6** as a white solid (89.5 mg, 86% yield).

**6**, ^1^H NMR (300 MHz, DMSO-*d*_6_) δ 7.44–7.27 (m, 5H), 7.28–7.18 (m, 5H), 7.13 (dd, *J* = 7.5, 1.1 Hz, 1H), 7.11–7.01 (m, 2H), 6.79–6.70 (m, 1H), 5.58 (s, 1H), 4.92 (d, *J* = 15.9 Hz, 1H), 4.70 (d, *J* = 16.0 Hz, 1H), 4.63–4.50 (m, 1H), 3.95 (d, *J* = 11.6 Hz, 2H), 3.68 (ddd, *J* = 11.1, 6.3, 3.8 Hz, 2H), 2.86–2.69 (m, 2H), 0.90 (s, 3H). ^13^C NMR{^1^H} (75 MHz, CDCl_3_) δ 218.1, 180.1, 141.7, 136.2, 135.2, 132.5, 130.3, 129.1, 128.5, 127.9, 127.4, 127.3, 126.7 (q, *J* = 277.5 Hz, 1C),126.6, 123.88, 123.6, 110.0, 75.5, 70.8, 59.4, 59.3, 58.7 (q, *J* = 30.0 Hz, 1C), 44.2, 43.2, 41.2, 14.8. HRMS (ESI) *m*/*z* [M+H]^+^ calcd. for C_30_H_28_F_3_N_2_O_3_, 521.2047, found: 521.2046.

### 3.6. Procedure for the Synthesis of Compound ***7***

The solution of compound **3aa** (0.1 g, 0.20 mmol) in DCM (2.0 mL) was stirred at room temperature in a sealed tube. Subsequently, *m*-CPBA (76.1 mg, 0.44 mmol) was added to the above solution. The reaction mixture was then allowed to stir for 48 h. The reaction mixture was quenched by the addition of NaHCO_3_ aq (15 mL) and diluted with EtOAc (15 mL). The organic layer was separated and the aqueous layer was extracted twice with EtOAc (2 × 15 mL). The combined organic layers were dried over Na_2_SO_4_. Subsequently, the organic phase was concentrated under reduced pressure. The crude product was purified by flash column chromatography on silica gel (petroleum ether/ethyl acetate = 15:1) to afford the desired product **7** as a white solid (44.9 mg, 42% yield).

**7**, **^1^**H NMR (300 MHz, CDCl_3_) δ 7.37–7.24 (m, 7H), 7.24–7.14 (m, 3H), 7.07 (m, *J* = 7.6, 1.0 Hz, 1H), 7.04–6.93 (m, 2H), 6.60 (d, *J* = 7.8 Hz, 1H), 5.07 (s, 1H), 4.76 (s, 2H), 4.60 (m, *J* = 6.2 Hz, 1H), 3.00 (d, *J* = 11.9 Hz, 1H), 2.93 (d, *J* = 12.9 Hz, 1H), 2.84 (d, *J* = 12.9 Hz, 1H), 2.46 (dd, *J* = 11.9, 6.0 Hz, 1H), 1.27 (s, 3H). ^13^C NMR{^1^H} (75 MHz, CDCl_3_) δ 214.2, 211.2, 174.7, 144.3, 134.9, 134.6, 130.8, 129.5, 129.1, 128.9, 128.2, 127.8, 127.2, 126.6, 125.8, 124.9 (q, *J* = 277.2 Hz, 1C), 124.2, 123.6, 110.1, 65.6 (q, *J* = 30.5 Hz, 1C), 62.3, 54.3, 48.2, 46.4, 43.8, 18.2. HRMS (ESI) *m*/*z* [M+H]^+^ calcd. for C_30_H_26_F_3_N_2_O_4_, 535.1839, found: 535.1845.

### 3.7. Procedure for the Synthesis of Compound ***8***

Compound **3aa** (0.10 g, 0.20 mmol) and polyformaldehyde (0.5 g, 30.0 equiv.) were dissolved in the mixture solvents MeOH and THF (1:1, 4.0 mL). Then, NaBH_3_CN (0.3 g, 20.0 equiv.) was added to the mixture at room temperature for 30 min. The reaction mixture was allowed to stir for 72 h at 50 °C. After completion, the reaction mixture was quenched by the addition of 1.0 M NaOH (10.0 mL). The aqueous layer was extracted with DCM (2 × 10.0 mL). The combined organic layers were dried over anhydrous Na_2_SO_4_. After filtration, the solution was concentrated under reduced pressure and the resulting crude mixture was purified by hexane beating twice to provide compound **8** as a white solid (100.1 mg, 89% yield).

**8**, ^1^H NMR (400 MHz, CDCl_3_) δ 7.31 (m, 5H), 7.27 (d, *J* = 6.5 Hz, 3H), 7.22 (d, *J* = 7.8 Hz, 2H), 7.08 (m, 1H), 7.04–6.96 (m, 2H), 6.65 (d, *J* = 7.8 Hz, 1H), 4.72 (s, 3H), 4.06 (d, *J* = 11.0 Hz, 1H), 3.73 (d, *J* = 11.0 Hz, 1H), 3.13 (d, *J* = 10.2 Hz, 1H), 2.99 (s, 3H), 2.96 (d, *J* = 12.7 Hz, 1H), 2.84 (d, *J* = 12.7 Hz, 1H), 2.60 (dd, *J* = 12.0, 4.0 Hz, 1H), 1.28 (s, 3H). ^13^C NMR{^1^H} (101 MHz, CDCl_3_) δ 214.5, 211.0, 177.5, 144.0, 135.3, 134.9, 130.6, 129.5, 129.0, 128.9, 128.1, 127.8, 127.4, 126.1, 125.7(q, *J* = 304.0 Hz, 1C),124.4, 123.6, 110.0, 79.2, 73.8, 63.3, 60.6(q, *J* = 30.0 Hz, 1C), 58.5, 56.4, 52.1, 46.4, 43.9, 17.7. HRMS (ESI) *m*/*z* [M+H]^+^ calcd. for C_32_H_30_F_3_N_2_O_4_, 563.2152, found: 563.2163.

### 3.8. Procedure for the Synthesis of Compound ***9***

To an oven-dried Schlenk tube, **3ak** (119.2 mg, 0.20 mmol), PhB(OH)_2_ (36.6 mg, 0.30mmol), and Cs_2_CO_3_ (130.4 mg, 0.40 mmol) were combined and then the mixture solvents ethanol (0.4 mL) and toluene (2.0 mL) were added to the reaction tube. The reaction mixture was stirred at 120 °C under N_2_ atmosphere for 12 h. The heat source was an oil bath. When the mixture was cooled to room temperature, the brine (10 mL) was added to quench the reaction. The aqueous layer was extracted with DCM (2 × 10.0 mL) and the combined organic layers were dried over anhydrous Na_2_SO_4_. After filtration, the organic phase was concentrated and purified by column chromatography on silica gel (petroleum ether/ethyl acetate = 15:1) to afford product **9** as a white solid (108.1 mg, 91% yield).

**9**, ^1^H NMR (400 MHz, DMSO-*d*_6)_ δ 7.64–7.53 (m, 2H), 7.46 (m, 3H), 7.43–7.39 (m, 1H), 7.39–7.37 (m, 1H), 7.36–7.23 (m, 8H), 7.13–6.95 (m, 3H), 4.93 (d, *J* = 15.9 Hz, 1H), 4.73 (d, *J* = 15.9 Hz, 1H), 4.69 (d, *J* = 5.6 Hz, 1H), 4.52–4.38 (m, 1H), 4.06 (d, *J* = 3.9 Hz, 2H), 3.02 (d, *J* = 13.3 Hz, 1H), 2.87 (d, *J* = 13.3 Hz, 1H), 0.96 (s, 3H). ^13^C NMR{^1^H} (101 MHz, DMSO-*d*_6_) δ 212.8, 210.8, 178.2, 143.9, 142.0, 139.9, 135.6, 134.5, 130.2, 129.0, 128.4, 128.1, 127.8, 127.7, 127.3, 127.3, 127.2, 126.7, 125.6 (q, *J* = 276.4 Hz, 1C), 124.6, 121.7, 108.1, 70.8, 62.0, 58.7 (q, *J* = 31.0 Hz, 1C)57.8, 51.3, 42.7, 41.5, 14.3. HRMS (ESI) *m*/*z* [M+H]^+^ calcd. for C_36_H_30_F_3_N_2_O_3_, 595.2203 found: 595.2209

## 4. Conclusions

In conclusion, we have developed a highly diastereoselective formal 1,3-dipolar cycloaddition reaction of *N*-2,2,2-trifluoroethylisatin ketimines and cyclopentene-1,3-diones that affords a series of tetracyclic spirooxindoles with good results (up to 96% yield and 91:9 dr). This reaction can lead to the desymmetrization of cyclopentene-1,3-diones, and provides an efficient method for constructing tetracyclic spirooxindoles containing fused pyrrolidine-cyclopentane subunits, which could be useful in the development of new pharmaceuticals. In addition, this 1,3-dipolar cycloaddition reaction could be extended to trifluoromethyl-substituted iminomalonate for the synthesis of bicyclic heterocycles bearing fused pyrrolidine-cyclopentane moieties in moderate yields with >20:1 dr under mild conditions. The potential applications of the protocol have been demonstrated by performing the gram-scale reaction and versatile transformations of the products. We believe that these novel compounds based on fused pyrrolidine–cyclopentane scaffolds can provide novel therapeutic agents and useful biological tools for the research and development of new drugs. Efforts toward the development of a catalytic asymmetric version of this 1,3-dipolar cycloaddition reaction and the application of this strategy to synthesize more promising drug discovery candidates, as well as the biological evaluation of these compounds, are currently underway in our laboratory.

## Figures and Tables

**Table 1:**
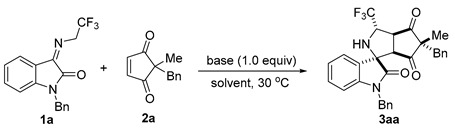
Optimization of reaction conditions [a].

Entry	Base	Solvent	Time (h)	dr (%) [b]	Yield [c]
1	TMG	DCE	12	81:19	73
2	DBU	DCE	12	77:23	87
3	DBN	DCE	12	69:31	59
4	TBD	DCE	12	77:23	66
5	CS_2_CO_3_	DCE	96	71:29	30
6	DBU	toluene	12	73:27	58
9	DBU	THF	12	74:26	42
10	DBU	MeCN	12	70:30	79
11	DBU	MeOH	12	64:36	73
12	DABCO	DCE	48	84:16	90
13 [d]	DABCO	DCE	24	84:16	90
14 [d,e]	DABCO	DCE	24	84:16	92
15 [d,e,f]	DABCO	DCE	24	85:15	96

[a] Unless otherwise noted, the reaction was carried out with **1a** (0.12 mmol), **2a** (0.1 mmol), and 1.0 equivalent of base in 1.0 mL of solvent at 30 °C. [b] Determined by ^1^H NMR analysis of the crude product. [c] Yield of isolated product as a mixture of diastereoisomers. [d] Run at 50 °C. [e] The ratio of **1a**:**2a** is 1.5:1. [f] 1.5 equivalents DABCO was used.

**Table 2:**
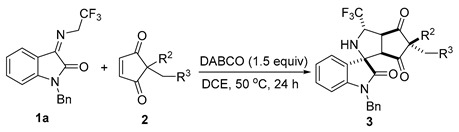
Substrate scope investigation of metal-free diastereoselective 1,3-dipolar cycloaddition of *N*-2,2,2-trifluoroethylisatin ketimines **1a** and diverse cyclopentene-1,3-diones **2** [a].

Entry	2 (R^2^/R^3^)	3	dr [b]	Yield (%) [c]
1	**2b** (Me/3-MeC_6_H_4_)	**3ab**	84:16	96
2	**2c** (Me/4-MeC_6_H_4_)	**3ac**	82:18	94
3	**2d** (Me/3-MeOC_6_H_4_)	**3ad**	85:15	94
4	**2e** (Me/4-FC_6_H_4_)	**3ae**	88:12	96
5	**2f** (Me/2-ClC_6_H_4_)	**3af**	71:29	76
6	**2g** (Me/4-ClC_6_H_4_)	**3ag**	86:14	94
7	**2h** (Me/3-BrC_6_H_4_)	**3ah**	84:16	84
8	**2i** (Me/4-BrC_6_H_4_)	**3ai**	85:15	94
9	**2j** (Me/1-naphthyl)	**3aj**	89:11	98
10	**2k** (Me/2-naphthyl)	**3ak**	86:14	99
11	**2l** (Et/C_6_H_5_)	**3al**	88:12	69
12	**2m** (Et/3-BrC_6_H_4_)	**3am**	72:28	84
13	**2n** (Et/4-BrC_6_H_4_)	**3an**	84:16	88

[a] The reactions were carried out with **1a** (0.15 mmol), **2** (0.10 mmol), and 1.5 equivalents of DABCO in 1.0 mL of DCE at 50 °C for 24 h. [b] Determined by ^1^H NMR analysis of the crude product. [c] Yield of isolated product as a mixture of diastereoisomers.

## Data Availability

All the required data are reported in the manuscript and Supplementary Materials.

## Supplementary Materials

The following supporting information can be downloaded at: https://www.mdpi.com/article/10.3390/molecules28145372/s1, Characterization data for obtained products; X-ray data for products **3aa**, **5a** and **8**; copies of ^1^H, ^13^C NMR and HRMS spectra. References [31,39,46,47] are cited in the supplementary materials.

## Sample Availability

Samples of the compounds are available from the authors.
