# Supplementary material for: Diastereoselective Formal 1,3-Dipolar Cycloaddition of Trifluoroethyl Amine-Derived Ketimines Enables the Desymmetrization of Cyclopentenediones
Source: Molecules. 2023 Jul 13;28(14):5372. doi: 10.3390/molecules28145372 (PMC10386021; doi:10.3390/molecules28145372)
Supplement: Supplementary file 1 [file molecules-28-05372-s001.zip › molecules-2493434-supplementary.pdf]

Diastereoselective Diastereoselective Formal 1,3-Dipolar Cycloaddition of Trifluoroethyl Amine-derived Ketimines Enables the Desymmetrization of Cyclopentenediones

Lin-Qiang Li[1,2,3], Jian-Qiang Zhao[1,*], Yan-Ping Zhang[1], Yong You [1], Zhen-Hua Wang[1], Zhen-Zhen Ge[3], Ming-Qiang Zhou[2,3], and Wei-Cheng Yuan[1,2,*]

1. Innovation Research Center of Chiral Drugs, Institute for Advanced Study, Chengdu University, Chengdu 610106, China
2. School of Pharmacy, Zunyi Medical University, Zunyi 563006, China
3. National Engineering Research Center of Chiral Drugs, Chengdu Institute of Organic Chemistry, Chinese Academy of Sciences, Chengdu 610041

Correspondence: zhaojianqiang@cdu.edu.cn (J.-Q. Zhao);
yuanwc@cioc.ac.cn (W.-C. Yuan)

Supporting Information

Table of Contents

1. General experimental information……S1
2. General experimental procedures for synthesis of compounds **1**……S1
3. General experimental procedures for synthesis of compound **2**……S1
4. General experimental procedures for synthesis of compound **4**……S2
5. General experimental procedures for synthesis of compounds **3**……S2
6. General experimental procedures for synthesis of compounds **5**……S16
7. Scale-up experiment……S20
8. Different transformations of products……S20
9. X-ray crystal structure of compounds **3aa**, **8** and **5a**……S22
10. ^{1}H NMR, ^{13}C NMR spectra for compounds **3**, **5**, **6**, **7**, **8**, **9**……S27
11. MS spectra for compounds **3**, **5**, **6**, **7**, **8**, **9**……S71

1. General experimental information

Reagents were purchased from commercial sources and were used as received unless mentioned otherwise. Reactions were monitored by TLC. ^{1}H NMR and ^{13}C NMR spectra were recorded in $CDCl_3$ or DMSO-d_6. 1H NMR chemical shifts are reported in ppm employed as the internal standard ($CDCl_3$ at 7.26 ppm, DMSO-d_6 at 2.50 ppm). Data are reported as follows: chemical shift, multiplicity (s = singlet, br s = broad singlet, d = doublet, t = triplet, q = quartet, m = multiplet), coupling constants (Hz) and integration. ^{13}C NMR chemical shifts are reported in ppm with the solvent resonance as the internal standard ($CDCl_3$ at 77.16 ppm, DMSO-d_6 at 39.52 ppm). Melting points products were recorded on a B üchi Melting Point B-545. The HRMS were recorded by The HRMS were recorded by Agilent 6545 LC/Q-TOF mass spectrometer.

2. General experimental procedures for synthesis of compounds 1.

The *N*-2,2,2-trifluoroethylisatin ketimines **1** are known compounds, which were prepared according to literatures. Compounds **1a-1q** were synthesized with same method**[1]**.

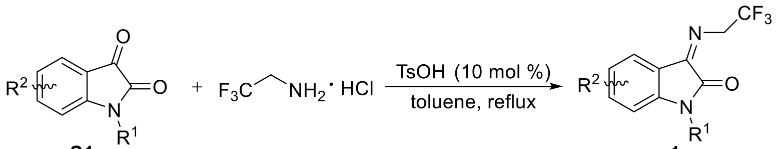

S1 **1**

In the dry reaction flask equipped with a water separator and a condenser was added the mixture of isatin **S1** (10.0 mmol), 2,2,2-trifluoroethylamine hydrochloride (15.0 mmol) and TsOH (1.0 mmol) in toluene (10.0 mL). Then, the mixture was refluxed to separate the water until complete disappearance of the isatin **S1**. Let cool slightly before evaporation of the toluene, and the crude reside was purified by flash chromatography on silica gel (petroleum ether / ethyl acetate = 15:1 to 8:1) to give the corresponding *N*-2,2,2-trifluoroethylisatin ketimines **1**.

3. General experimental procedures for synthesis of compound 2.

The 2-methyl-2-(phenylmethyl)-4-cyclopentene-1,3-dione **2** are known compounds, which were prepared according to literatures. Compounds **2a-2n** were synthesized with same method**[2]**.

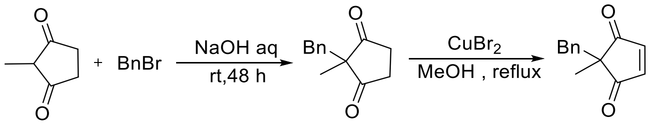

S2 **2**

2-methylcyclopentane-1,3-dione (2.2 g, 20.0 mmol, 1.0 equiv) was stirred with 1.0 M aq. NaOH solution (1.0 equiv) at room temperature for 10 min. To this suspension benzyl bromide (6.8 g, 40.0 mmol, 2.0 equiv) was added at once and the resulting bi-phasic solution was stirred vigorously. After being stirred for 48 hours, the reaction mixture was diluted with EtOAc (50.0 mL). The aqueous phase was back-extracted with EtOAc twice (50.0 mL×2). The combined organic phase was dried over anhydrous Na_2SO_4 and concentrated under reduced pressure. After an evaporation of the organic solvent, the crude residue was purified by chromatography (silica gel, hexane/ethyl acetate = 7:1). Compound **S2** (3.5 g, 87% yield) was obtained as a white solid according to the procedure mentioned above.

To a solution of 2,2-disubstituted cyclopentane-1,3-dione (1.6 g, 8.0 mmol, 1.0 equiv) in MeOH (50.0 mL) under argon atmosphere was added copper (II) bromide (3.9 g, 17.6 mmol, 2.2 equiv) and the resulting brown solution was refluxed for 1.0 hour. The reaction mixture was cooled to room temperature and then added in sequence: 10.0 mL of H_2O, 10.0 mL of HCl aq (1.0 M), and 50.0 mL of DCM. The aqueous phase was separated and extracted for 2 times with DCM. The collected organic phases were dried over $MgSO_4$, filtered and concentrated under reduced pressure. After an evaporation of the organic solvent, the crude residue was purified by chromatography (silica gel, hexane / ethyl acetate = 10:1). Compound **2** (1.5 g, 94% yield) was obtained as a yellow solid according to the procedure mentioned above.

4. General experimental procedures for synthesis of compound 4[3].

The diethyl 2-((2,2,2-trifluoroethyl)imino)malonate **4** is known compound, which was prepared according to literatures.

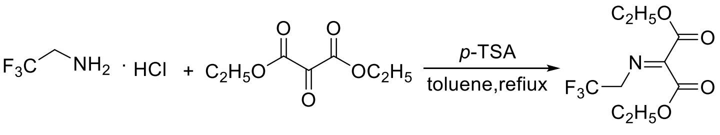

4

Diethyl ketomalonate (2.4 g, 14.0 mmol, 1.0 equiv); 2,2,2-trifluoroethyl-amine hydrochloride (3.8 g, 28.0 mmol, 2.0 equiv), and *p*-toluenesulfonic acid (0.2 g, 1.4 mmol, 0.1 equiv) were suspended in toluene (40.0 mL) in a two-neck flask with a water separator and a condenser. The mixture was then heated to separate the water until complete disappearance of the starting materials (about 30-60 minutes), after which it was cooled to room temperature, washed with a small quantity of saturated $NaHCO_3$ solution, extracted with ethyl acetate and washed with brine, and dried over anhydrous Na_2SO_4. After an evaporation of the organic solvent, the crude residue was purified by chromatography (silica gel, hexane / ethyl acetate = 5:1). Ketimine **4** (3.0 g, 84% yield) was obtained as a pale yellow liquid according to the procedure mentioned above.

5. General experimental procedures for synthesis of compounds 3.

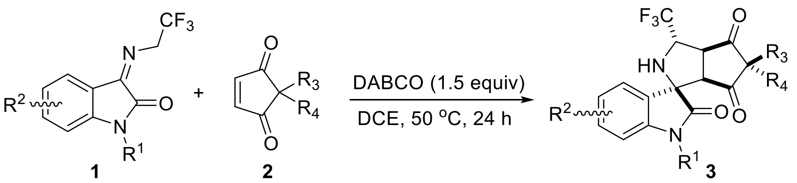

1 **2** **3**

In a dry reaction tube equipped with a magnetic stirring bar, *N*-2,2,2-trifluoroethylisatin ketimines **1** (0.3 mmol), DABCO (33.6 mg, 0.3mmol, 1.5 equiv), and **2** (40.2 mg, 0.2 mmol, 1.0 equiv), DCE (2.0 mL) were added in sequence. Then the mixture was stirred for 24 h at 50 °C，After completion, the reaction mixture was directly purified by flash chromatography on silica gel (petroleum ether / ethyl acetate, 15:1 - 10:1) to give the corresponding products **3**.

1',5-dibenzyl-5-methyl-3-(trifluoromethyl)-2,3,3a,6a-tetrahydro-4H-spiro[cyclopenta[c]pyrrole-1,3'-indoline] -2',4,6(5H)-trione (3aa)

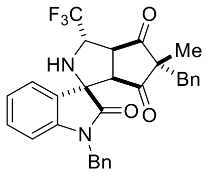

The product was purified by flash column chromatography (petroleum ether / ethyl acetate = 15:1 as the eluent);

White solid; 99.5 mg, 96% yield; 85:15 *dr*, mp 194.4-195.3 °C;

^{1}H NMR (400 MHz, DMSO-*d*$_6$) *δ* 7.38 (d, *J* = 7.3 Hz, 1H), 7.35 – 7.19 (m, 9H), 7.11 (m, 1H), 7.07 – 7.01 (m, 2H), 6.74 (d, *J* = 7.8 Hz, 1H), 4.85 (d, *J* = 15.9 Hz, 1H), 4.66 (d, *J* = 5.6 Hz, 1H), 4.59 (d, *J* = 15.9 Hz, 1H), 4.44 (m, 1H), 4.04 (d, *J* = 3.1 Hz, 2H), 3.01 (d, *J* = 13.3 Hz, 1H), 2.85 (d, *J* = 13.3 Hz, 1H), 0.95 (s, 3H).

^{13}C{^{1}H} NMR (101 MHz, DMSO-*d*$_6$) δ 212.8, 210.8, 178.0, 143.1, 135.5, 134.5, 130.2, 129.6, 128.6, 128.4, 128.08, 127.4, 127.2, 127.1, 125.6 (q, *J* = 276.8 Hz, 1C), 124.1, 123.1, 109.7, 70.9, 62.0, 58.7 (q, *J* = 30.4 Hz, 1C), 57.8, 51.3, 42.7, 41.4, 14.3.

HRMS (ESI) *m/z*: $[M+H]^+$ calcd for $C_{30}H_{26}F_3N_2O_3$, 519.1890, found: 519.1895.

5-benzyl-1',5-dimethyl-3-(trifluoromethyl)-2,3,3a,6a-tetrahydro-4H-spiro[cyclopenta[c] pyrrole-1,3'-indoline]-2',4,6(5H)-trione (3ba)

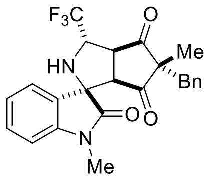

The product was purified by flash column chromatography (petroleum ether / ethyl acetate = 15:1 as the eluent);

White solid; 81.4 mg, 92% yield; 83:17 *dr*, mp 239.0-239.7 °C;

^{1}H NMR (400 MHz, DMSO-*d*$_6$) δ 7.42 – 7.31 (m, 2H), 7.30 – 7.19 (m, 3H), 7.14 (m, 1H), 7.02 (m, 3H), 4.50 (d, *J* = 5.6 Hz, 1H), 4.39 (m, 1H), 4.02 (dd, *J* = 12.0, 4.0 Hz, 1H), 3.97 (d, *J* = 12.0 Hz, 1H), 2.96 (s, 3H), 2.93 (s, 1H), 2.83 (d, *J* = 13.3 Hz, 1H), 0.92 (s, 3H).

^{13}C{^{1}H} NMR (101 MHz, DMSO-*d*$_6$) δ 212.7, 210.8, 177.8, 144.0, 134.4, 130.2, 129.7, 128.6, 128.0, 127.1,125.6 (q, *J* = 276.8 Hz, 1C), 123.9, 123.0, 108.9, 70.94, 62.0, 58.7 (q, *J* = 30.4 Hz, 1C), 57.7, 51.1, 41.3, 25.7, 14.4.

HRMS (ESI) *m/z*: $[M+H]^+$ calcd for $C_{24}H_{22}F_3N_3O_3$, 443.1577, found: 443.1572.

5-benzyl-1'-ethyl-5-methyl-3-(trifluoromethyl)-2,3,3a,6a-tetrahydro-4H-spiro[cyclopenta[c] pyrrole-1,3'-indoline]-2',4,6(5H)-trione (3ca)

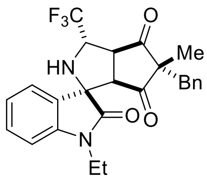

The product was purified by flash column chromatography (petroleum ether / ethyl acetate = 15:1 as the eluent);

White solid; 83.8 mg, 92% yield; 90:10 *dr*, mp 158.1-158.8 °C;

^{1}H NMR (400 MHz, $CDCl_3$) δ 7.29 – 7.16 (m, 5H), 7.03 (m, 1H), 6.94 – 6.85 (m, 2H), 6.75 (d, *J* = 7.8 Hz, 1H), 4.56 – 4.43 (m, 1H), 3.61 (m, 1H), 3.38 (m, 1H), 3.03 (d, *J* = 12.0 Hz, 1H), 2.84 (d, *J* = 12.9 Hz, 1H), 2.75 (d, *J* = 12.9 Hz, 1H), 2.61 (dd, *J* = 12.0, 4.5 Hz, 1H), 2.18 (s, 1H), 1.19 (s, 3H), 1.10 (t, *J* = 7.2 Hz, 3H).

$^{13}C\{^1H\}$ NMR (101 MHz, DMSO-d_6) δ 212.3, 210.8, 177.6, 142.9, 134.5, 130.2, 129.7, 128.8, 128.0, 127.1,125.6 (q, *J* = 279.8 Hz, 1C), 124.1, 122.9, 109.0, 70.6, 61.8, 58.8 (q, *J* = 30.3 Hz, 1C), 58.0, 51.1, 41.3, 33.8, 14.3, 12.2.

HRMS (ESI) *m/z*: $[M+H]^+$ calcd for $C_{25}H_{24}F_3N_2O_3$, 457.1734, found: 457.1736.

1'-allyl-5-benzyl-5-methyl-3-(trifluoromethyl)-2,3,3a,6a-tetrahydro-4H-spiro[cyclopenta[c]pyrrole-1,3'-indoline]-2',4,6(5H)-trione (3da)

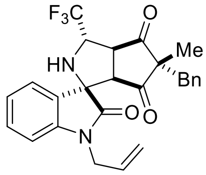

The product was purified by flash column chromatography (petroleum ether / ethyl acetate = 15:1 as the eluent);

White solid; 90.1 mg, 96% yield, 83:17 *dr*, mp 149.0-149.7 °C;

^{1}H NMR (400 MHz, DMSO-d_6) δ 7.36 (m, 2H), 7.28 (m, *J* = 3.7 Hz, 3H), 7.15 m, 1H), 7.03 (dd, *J* = 7.3, 2.3 Hz, 2H), 6.92 (d, *J* = 7.8 Hz, 1H), 5.70 (m, 1H), 5.22 (dd, *J* = 17.3, 1.7 Hz, 1H), 5.13 (dd, *J* = 10.4, 1.7 Hz, 1H), 4.58 (d, *J* = 5.6 Hz, 1H), 4.42 (m, 1H), 4.22 (m, 1H), 4.05 (dd, *J* = 9.9, 6.6 Hz, 1H), 4.01 (d, *J* = 3.8 Hz, 1H), 3.98 (d, *J* = 11.9 Hz, 1H), 2.95 (d, *J* = 13.3 Hz, 1H), 2.87 (d, *J* = 13.3 Hz, 1H), 0.92 (s, 3H).

$^{13}C\{^1H\}$ NMR (101 MHz, DMSO-d_6) δ 212.6, 210.9, 177.7, 143.1, 134.5, 131.3, 130.3, 129.6, 128.6, 128.0, 127.1, 125.6 (q, *J* = 279.1 Hz, 1C),124.1, 123.0, 116.9, 109.6, 70.8, 61.9, 58.7 (q, *J* = 30.6 Hz, 1C), 57.9, 51.1, 41.4, 41.3, 14.3.

HRMS (ESI) *m/z*: $[M+H]^+$ calcd for $C_{26}H_{24}F_3N_2O_3$, 469.1734, found: 469.1735.

1',5-dibenzyl-5'-fluoro-5-methyl-3-(trifluoromethyl)-2,3,3a,6a-tetrahydro-4H-spiro[cyclopenta[c]pyrrole-1,3'-indoline]-2',4,6(5H)-trione (3ea)

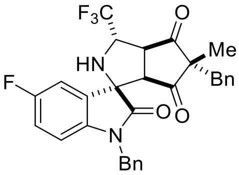

The product was purified by flash column chromatography (petroleum ether / ethyl acetate =15:1 as the eluent);

White solid; 84.7 mg, 79% yield; 84:16 *dr*, mp 209.0-209.7 °C;

^{1}H NMR (400 MHz, DMSO-d_6) δ 7.32 (d, *J* = 3.3 Hz, 4H), 7.28 (m, 4H), 7.25 (d, *J* = 2.5 Hz, 1H), 7.13 (m, 1H), 7.09 – 6.99 (m, 2H), 6.76 (dd, *J* = 8.6, 4.1 Hz, 1H), 4.86 (d, *J* = 16.0 Hz, 1H), 4.76

(d, J = 5.6 Hz, 1H), 4.61 (d, J = 16.0 Hz, 1H), 4.52 – 4.36 (m, 1H), 4.13 (d, J = 12.0 Hz, 1H), 4.05 (dd, J = 12.1, 4.4 Hz, 1H), 3.01 (d, J = 13.3 Hz, 1H), 2.88 (d, J = 13.3 Hz, 1H), 0.97 (s, 3H).

$^{13}C\{^1H\}$ NMR (101 MHz, DMSO-d_6) δ 213.2, 211.2, 178.4, 160.6, 158.2, 139.7 (d, J = 1.8 Hz, 1C), 135.7, 134.9, 130.9 (d, J = 7.9 Hz, 1C), 129.6 (d, J = 217.1 Hz, 1C),128.9, 127.9, 127.7, 127.6, 126.0 (q, J = 279.4 Hz, 1C),116.4 (d, J =23.3 Hz, 1C), 112.6 (d, J = 25.0 Hz, 1C) 111.2 (d, J = 8.0 Hz, 1C), 71.5 (d, J = 1.0 Hz, 1C), 62.5, 59.3 (q, J = 31.3 Hz, 1C), 58.2, 51.6, 43.3, 41.9, 14.8.

HRMS (ESI) *m/z*: $[M+H]^+$ calcd for $C_{30}H_{25}F_4N_2O_3$, 537.1796, found: 537.1806.

1',5-dibenzyl-6'-fluoro-5-methyl-3-(trifluoromethyl)-2,3,3a,6a-tetrahydro-4H-spiro[cyclopenta[c]pyrrole-1,3'-indoline]-2',4,6(5H)-trione (3fa)

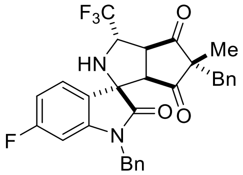

The product was purified by flash column chromatography (petroleum ether / ethyl acetate =15:1 as the eluent);

White solid; 87.9 mg, 82% yield; 87:13 *dr*, mp. 209.1-209.7 ℃;

^1H NMR (400 MHz, DMSO-d_6) δ 7.32 (dd, J = 8.3, 5.5 Hz, 1H), 7.28 – 7.11 (m, 8H), 6.97 (dd, J = 7.2, 2.3 Hz, 2H), 6.84 (m, 1H), 6.64 (dd, J = 9.4, 2.3 Hz, 1H), 4.76 (d, J = 15.9 Hz, 1H), 4.59 (d, J = 5.5 Hz, 1H), 4.53 (d, J = 15.9 Hz, 1H), 4.34 (m, J = 6.8, 6.3 Hz, 1H), 3.97 (d, J = 2.0 Hz, 2H), 2.92 (d, J = 13.3 Hz, 1H), 2.77 (d, J = 13.3 Hz, 1H), 0.86 (s, 3H).

$^{13}C\{^1H\}$ NMR (101 MHz, DMSO-d_6) δ 213.2, 211.2, 178.8, 164.8, 162.3, 145.3 (d, J = 12.1 Hz, 1C), 135.6, 134.9, 128.9, 128.1 (d, J = 218.1 Hz, 1C), 127.9, 127.8, 127.6, 126.3 (d, J = 10.2 Hz, 1C), 126.0 (q, J = 279.8 Hz, 1C), 124.8 (d, J = 2.8 Hz, 1C), 109.6 (d, J = 22.5 Hz, 1C), 98.8 (d, J = 28.1 Hz, 1C), 70.9, 62.5, 59.1(q, J = 31.0 Hz, 1C), 58.2, 51.6, 43.3, 41.9, 14.7.

HRMS (ESI) *m/z*: $[M+H]^+$ calcd for $C_{30}H_{25}F_4N_2O_3$, 537.1796, found: 537.1800.

1',5-dibenzyl-5'-chloro-5-methyl-3-(trifluoromethyl)-2,3,3a,6a-tetrahydro-4H-spiro[cyclopenta[c]pyrrole-1,3'-indoline]-2',4,6(5H)-trione (3ga)

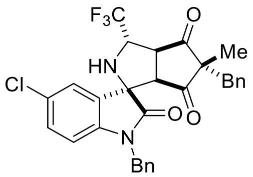

The product was purified by flash column chromatography (petroleum ether /ethyl acetate = 15:1 as the eluent);

White solid; 100.5 mg, 91% yield; 85:15 *dr*, mp 249.3-250.0 ℃;

^1H NMR (400 MHz, DMSO-d_6) δ 7.42 (d, J = 2.2 Hz, 1H), 7.38 – 7.21 (m, 9H), 7.09 – 7.02 (m, 2H), 6.77 (d, J = 8.4 Hz, 1H), 4.86 (d, J = 16.0 Hz, 1H), 4.76 (d, J = 5.6 Hz, 1H), 4.60 (d, J = 16.0 Hz, 1H), 4.44 (m, 1H), 4.19 (d, J = 12.1 Hz, 1H), 4.05 (dd, J = 12.0, 4.5 Hz, 1H), 2.99 (d, J = 13.3 Hz, 1H), 2.88 (d, J = 13.3 Hz, 1H), 0.95 (s, 3H).

^{13}C{^{1}H} NMR (101 MHz, DMSO-*d*$_6$) δ 212.7, 210.7, 177.7, 142.0, 135.1, 134.4, 130.6, 130.3, 129.5, 128.5, 128.1, 127.5, 127.3, 127.2, 127.2, 125.8 (q, *J* = 260.1 Hz, 1C), 124.5, 111.2, 70.8, 62.8, 58.7 (q, *J* = 30.6 Hz, 1C), 57.7, 51.1, 42.8, 41.5, 14.3.

HRMS (ESI) *m/z*: [M+H]$^+$ calcd for $C_{30}H_{25}{}^{35}ClF_3N_2O_3$, 553.1500, found: 553.1512; calcd for $C_{30}H_{24}{}^{37}ClF_3N_2O_3$, 555.1486, found: 555.1494.

1',5-dibenzyl-6'-chloro-5-methyl-3-(trifluoromethyl)-2,3,3a,6a-tetrahydro-4H-spiro[cyclopenta[c]pyrrole-1,3'-indoline]-2',4,6(5H)-trione (3ha)

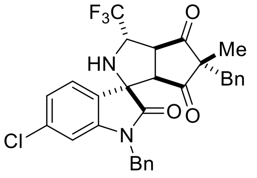

The product was purified by flash column chromatography (petroleum ether / ethyl acetate = 15:1 as the eluent);

White solid; 68.5 mg, 82% yield; 88:12 *dr*, mp 253.8-254.4 °C;

^{1}H NMR (400 MHz, DMSO-*d*$_6$) δ 7.39 (d, *J* = 8.0 Hz, 1H), 7.37 – 7.21 (m, 8H), 7.17 (dd, *J* = 7.9, 1.9 Hz, 1H), 7.05 (dd, *J* = 7.2, 2.3 Hz, 2H), 6.88 (d, *J* = 1.9 Hz, 1H), 4.86 (d, *J* = 16.0 Hz, 1H), 4.70 (d, *J* = 5.5 Hz, 1H), 4.63 (d, *J* = 16.0 Hz, 1H), 4.47 – 4.37 (m, 1H), 4.06 (d, *J* = 1.9 Hz, 2H), 3.01 (d, *J* = 13.3 Hz, 1H), 2.85 (d, *J* = 13.3 Hz, 1H), 0.94 (s, 3H).

^{13}C{^{1}H} NMR (101 MHz, DMSO-*d*$_6$) δ 212.7, 210.6, 178.1, 144.6, 135.1, 134.4, 134.0, 130.2, 128.5, 128.1, 127.49, 127.47, 127.3, 127.2, 125.7,125.6 (q, *J* = 271.2 Hz, 1C), 122.9, 110.0, 70.5, 62.1, 58.7 (q, *J* = 30.9 Hz, 1C), 57.8, 51.1, 42.8, 41.4, 14.2.

HRMS (ESI) *m/z*: [M+H]$^+$ calcd for $C_{30}H_{25}{}^{35}ClF_3N_2O_3$, 553.1500, found: 553.1511; calcd for $C_{30}H_{24}{}^{37}ClF_3N_2O_3$, 555.1486, found: 555.1493.

1',5-dibenzyl-7'-chloro-5-methyl-3-(trifluoromethyl)-2,3,3a,6a-tetrahydro-4H-spiro[cyclopenta[c]pyrrole-1,3'-indoline]-2',4,6(5H)-trione (3ia)

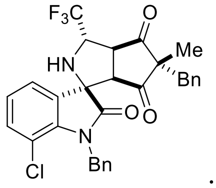

.

The product was purified by flash column chromatography (petroleum ether / ethyl acetate = 15:1 as the eluent);

White solid; 68.5 mg, 62% yield; 86:14 *dr*, mp 190.7-191.6 °C;

^{1}H NMR (400 MHz, CDCl$_3$) 7.31 – 7.18 (m, 5H), 7.19 – 7.04 (m, 5H), 7.03 – 6.81 (m, 3H), 5.09 (d, *J* = 16.2 Hz, 1H), 5.01 (d, *J* = 16.2 Hz, 1H), 4.45 (m, 1H), 3.02 (d, *J* = 12.0 Hz, 1H), 2.85 (d, *J* = 12.9 Hz, 1H), 2.73 (d, *J* = 12.9 Hz, 1H), 2.56 (dd, *J* = 12.1, 4.5 Hz, 1H), 2.24 (s, 1H), 1.07 (s, 3H).

^{13}C{^{1}H} NMR (101 MHz, CDCl$_3$) δ 214.2, 211.7, 178.6, 139.1, 136.6, 134.6, 132.8, 130.9, 129.4, 128.9, 128.6, 128.0, 127.3, 126.5, 124.9 (q, *J* = 279.5 Hz, 1C), 124.8, 123.1, 116.0, 70.5, 63.0, 59.6, 59.5 (q, *J* = 32.0 Hz, 1C), 52.7, 46.2, 45.1, 17.7.

HRMS (ESI) *m/z*: $[M+H]^+$ calcd for $C_{30}H_{25}{}^{35}ClF_3N_2O_3$, 553.1500, found: 553.1511; calcd for $C_{30}H_{24}{}^{37}ClF_3N_2O_3$, 555.1486, found: 555.1492.

1',5-dibenzyl-4'-bromo-5-methyl-3-(trifluoromethyl)-2,3,3a,6a-tetrahydro-4H-spiro[cyclopenta[c]pyrrole-1,3'-indoline]-2',4,6(5H)-trione (3ja)

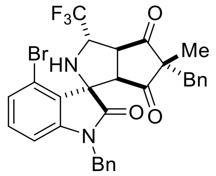

The product was purified by flash column chromatography (petroleum ether / ethyl acetate = 15:1 as the eluent);

White solid; 81.2 mg, 68% yield; 91: 9 *dr*, mp 238.1-238.8 °C;

^{1}H NMR (400 MHz, DMSO-d_6) δ 7.41 – 7.16 (m, 10H), 7.06 (dd, *J* = 7.1, 2.4 Hz, 2H), 6.78 (d, *J* = 7.6 Hz, 1H), 4.87 (d, *J* = 16.0 Hz, 1H), 4.68 – 4.54 (m, 2H), 4.41 (dd, *J* = 12.4, 6.6 Hz, 2H), 3.99 (dd, *J* = 12.3, 4.6 Hz, 1H), 3.07 (d, *J* = 13.2 Hz, 1H), 2.87 (d, *J* = 13.2 Hz, 1H), 0.98 (s, 3H).

13C{^{1}H} NMR (101 MHz, DMSO-d_6) δ 213.6, 211.0, 177.5, 145.4, 135.0, 134.5, 131.6, 130.2, 128.5, 128.1, 127.5, 127.2 127.2, 127.2, 125.9 (q, *J* = 256.4 Hz, 1C), 125.0, 119.0, 109.3, 72.2, 62.2, 58.5 (q, *J* = 30.9 Hz, 1C), 54.0, 51.1, 42.9, 41.6, 14.7.

HRMS (ESI) *m/z*: $[M+H]^+$ calcd for $C_{30}H_{25}{}^{79}BrF_3N_2O_3$, 597.0995, found: 597.1014; calcd for $C_{30}H_{24}{}^{81}BrF_3N_2O_3$, 599.0980, found: 599.0999.

1',5-dibenzyl-5'-bromo-5-methyl-3-(trifluoromethyl)-2,3,3a,6a-tetrahydro-4H-spiro[cyclopenta[c]pyrrole-1,3'-indoline]-2',4,6(5H)-trione (3ka)

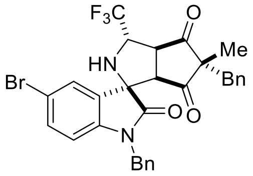

The product was purified by flash column chromatography (petroleum ether / ethyl acetate = 15:1 as the eluent);

White solid; 116.8 mg, 98% yield; 85:15 *dr*, mp 204.3-205.2 °C;

^{1}H NMR (400 MHz, DMSO-d_6) δ 7.42 – 7.30 (m, 6H), 7.28 (d, *J* = 6.7 Hz, 4H), 7.11 – 7.03 (m, 2H), 7.02 (s, 1H), 4.87 (d, *J* = 16.0 Hz, 1H), 4.72 (d, *J* = 5.6 Hz, 1H), 4.64 (d, *J* = 16.0 Hz, 1H), 4.44 (m, 1H), 4.08 (d, *J* = 1.9 Hz, 2H), 3.02 (d, *J* = 13.3 Hz, 1H), 2.87 (d, *J* = 13.3 Hz, 1H), 0.94 (s, 3H).

^{13}C{^{1}H} NMR (101 MHz, DMSO-d_6) δ 212.7, 210.6, 178.0, 144.6, 135.1, 134.4, 130.2, 128.5, 128.1, 127.9, 127.5, 127.2, 127.2, 126.0, 125.8, 125.5 (q, *J* =280.1 Hz, 1C),122.3, 112.7, 70.5, 62.1, 58.7 (q, *J* =31.2 Hz, 1C), 57.7, 51.1, 42.7, 41.3, 14.2.

HRMS (ESI) *m/z*: $[M+H]^+$ calcd for $C_{30}H_{25}{}^{79}BrF_3N_2O_3$, 597.0995, found: 597.0992; calcd for $C_{30}H_{24}{}^{81}BrF_3N_2O_3$ 599.0980, found: 599.0977.

1',5-dibenzyl-7'-bromo-5-methyl-3-(trifluoromethyl)-2,3,3a,6a-tetrahydro-4H-spiro[cyclopenta[c]pyrrole-1,3'-indoline]-2',4,6(5H)-trione (3la)

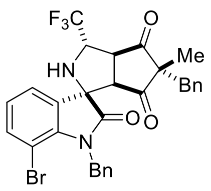

The product was purified by flash column chromatography (petroleum ether / ethyl acetate = 15:1 as the eluent);

White solid; 75.1 mg, 63% yield; 85:15 *dr*, mp 151.5-152.3 °C;

^{1}H NMR (400 MHz, DMSO-*d*$_6$) δ 7.46 (m, 2H), 7.34 – 7.25 (m, 5H), 7.25 – 7.21 (m, 1H), 7.21 – 7.16 (m, 2H), 7.11 (m, 1H), 7.08 – 7.00 (m, 2H), 5.06 (s, 2H), 4.75 (d, *J* = 5.5 Hz, 1H), 4.41 (dd, *J* = 9.8, 4.9 Hz, 1H), 4.08 (d, *J* = 2.8 Hz, 2H), 3.00 (d, *J* = 13.3 Hz, 1H), 2.86 (d, *J* = 13.3 Hz, 1H), 0.86 (s, 3H).

^{13}C{^{1}H} NMR (101 MHz, DMSO-*d*$_6$) δ 212.7, 210.5, 179.0, 140.5, 137.0, 135.2, 134.4, 132.2, 130.2, 28.2, 128.1, 127.2, 126.8, 125.9, 125.6 (q, *J* =271.4 Hz, 1C),125.1 124.0, 101.7, 70.2, 62.0, 58.8 (q, *J* =31.4 Hz, 1C), 58.1, 51.1, 44.2, 41.3, 14.1.

HRMS (ESI) *m/z*: [M+H]$^+$ calcd for $C_{30}H_{25}{}^{79}BrF_3N_2O_3$, 597.0995, found: 597.0996; calcd for $C_{30}H_{24}{}^{81}BrF_3N_2O_3$, 599.0980, found: 599.0979.

1',5-dibenzyl-5-methyl-3,7'-bis(trifluoromethyl)-2,3,3a,6a-tetrahydro-4H-spiro[cyclopenta[c]pyrrole-1,3'-indoline]-2',4,6(5H)-trione (3ma)

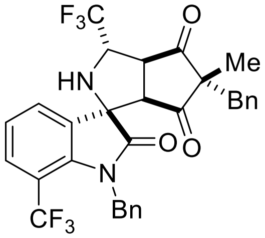

The product was purified by flash column chromatography (petroleum ether / ethyl acetate = 15:1 as the eluent);

White solid; 80.9 mg, 69% yield; 83:17 *dr,* mp 248.2-248.8 °C;

^{1}H NMR (400 MHz, DMSO-*d*$_6$) δ 7.77 (d, *J* = 7.4 Hz, 1H), 7.71 (d, *J* = 8.2 Hz, 1H), 7.40 (m, 1H), 7.26 (m, 5H), 7.19 (m, 1H), 7.12 (d, *J* = 7.6 Hz, 2H), 7.09 – 7.00 (m, 2H), 4.92 (d, *J* = 17.6 Hz, 1H), 4.82 (s, 1H), 4.78 (s, 1H), 4.41 (m, 1H), 4.17 (d, *J* = 12.0 Hz, 1H), 4.12 (dd, *J* = 12.0, 3.8 Hz, 1H), 2.99 (d, *J* = 13.3 Hz, 1H), 2.87 (d, *J* = 13.3 Hz, 1H), 0.81 (s, 3H).

^{13}C{^{1}H} NMR (101 MHz, DMSO-*d*$_6$) δ 213.2, 210.9, 180.3, 141.8, 136.3, 134.8, 132.2, 130.7, 129.5, 128.6, 128.5, 128.2, 127.6, 127.0, 126.4 (q, *J* = 273.7 Hz, 1C), 126.0 (q. *J* = 278.8 Hz), 125.7, 124.0, 112.0 (q, *J* = 33.3 Hz), 69.2, 62.5, 59.1 (q, *J* = 31.3 Hz, 1C), 58.4, 51.5, 46.3, 41.8, 14.5.

HRMS (ESI) *m/z*: [M+H]$^+$ calcd for $C_{31}H_{25}F_6N_2O_3$, 587.1764, found: 587.1772.

1',5-dibenzyl-5'-methoxy-5-methyl-3-(trifluoromethyl)-2,3,3a,6a-tetrahydro-4H-spiro[cyclopenta[c]pyrrole-1,3'-indoline]-2',4,6(5H)-trione (3na)

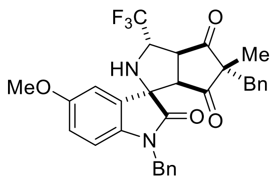

The product was purified by flash column chromatography (petroleum ether / ethyl acetate =15:1 as the eluent);

White solid; 105.3 mg, 96% yield; 80:20 *dr*, mp 188.5-189.4 °C;

^{1}H NMR (400 MHz, DMSO-*d*$_6$) δ 7.38 – 7.20 (m, 8H), 7.13 – 7.02 (m, 2H), 6.99 (d, *J* = 2.5 Hz, 1H), 6.83 (dd, *J* = 8.6, 2.6 Hz, 1H), 6.65 (d, *J* = 8.5 Hz, 1H), 4.84 (d, *J* = 15.9 Hz, 1H), 4.68 (d, *J* = 5.6 Hz, 1H), 4.58 (d, *J* = 15.9 Hz, 1H), 4.47 (m, *J* = 10.5, 5.2 Hz, 1H), 4.14 – 3.99 (m, 2H), 3.74 (s, 3H), 3.01 (d, *J* = 13.3 Hz, 1H), 2.87 (d, *J* = 13.3 Hz, 1H), 0.98 (s, 3H).

^{13}C{^{1}H} NMR (101 MHz, DMSO-*d*$_6$) δ 212.8, 210.8, 177.7, 156.0, 136.3, 135.5, 134.5, 130.3, 129.9, 128.4, 128.1, 127.3, 127.2, 127.2, 125.6 (q, *J* = 279.3 Hz, 1C), 114.1, 111.1 110.3, 71.3, 62.0, 58.8 (q, *J* = 30.9 Hz, 1C), 57.9, 55.6, 51.3, 42.8, 41.5, 14.4.

HRMS (ESI) *m/z*: $[M+H]^+$ calcd for $C_{31}H_{28}F_3N_2O_4$, 549.1996, found: 549.1998.

1',5-dibenzyl-6'-methoxy-5-methyl-3-(trifluoromethyl)-2,3,3a,6a-tetrahydro-4H-spiro[cyclopenta[c]pyrrole-1,3'-indoline]-2',4,6(5H)-trione (3oa)

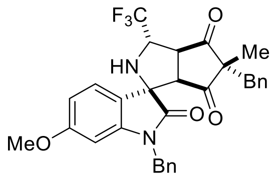

The product was purified by flash column chromatography (petroleum ether / ethyl acetate =15:1 as the eluent);

White solid; 88.8 mg, 81% yield; 85:15 *dr*, mp 205.1-205.9 °C;

^{1}H NMR (400 MHz, DMSO-*d*$_6$) δ 7.27 – 7.11 (m, 9H), 7.04 – 6.93 (m, 2H), 6.58 (dd, *J* = 8.3, 2.3 Hz, 1H), 6.28 (d, *J* = 2.3 Hz, 1H), 4.75 (d, *J* = 15.8 Hz, 1H), 4.57 – 4.43 (m, 2H), 4.33 (m, *J* = 9.7, 5.2 Hz, 1H), 3.98 – 3.78 (m, 2H), 3.62 (s, 3H), 2.93 (d, *J* = 13.4 Hz, 1H), 2.76 (d, *J* = 13.9 Hz, 1H), 0.86 (s, 3H).

^{13}C{^{1}H} NMR (101 MHz, DMSO-*d*$_6$) δ 212.8, 210.9, 178.5, 160.8, 144.4, 135.6, 134.5, 130.2, 128.4, 128.1, 127.4, 127.3, 127.2, 125.6 (q, *J* =279.8 Hz, 1C),125.0, 120.2, 106.9, 97.6, 70.7, 62.0, 58.5 (q, *J* = 31.0 Hz, 1C), 57.7, 55.4, 51.2, 42.7, 41.5, 14.4.

HRMS (ESI) *m/z*: $[M+H]^+$ calcd for $C_{31}H_{28}F_3N_2O_4$, 549.1996, found: 549.1998.

1',5-dibenzyl-5,5'-dimethyl-3-(trifluoromethyl)-2,3,3a,6a-tetrahydro-4H-spiro[cyclopenta[c]pyrrole-1,3'-indoline]-2',4,6(5H)-trione (3pa)

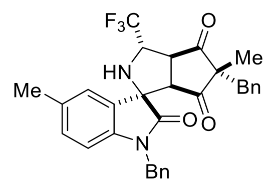

The product was purified by flash column chromatography (petroleum ether / ethyl acetate =15:1

as the eluent);

White solid; 101.1 mg, 95% yield; 89:11 *dr*, mp 231.8-232.4 °C;

^{1}H NMR (400 MHz, DMSO-*d*$_6$) δ 7.50 – 7.22 (m, 8H), 7.20 (s, 1H), 7.13 – 6.94 (m, 3H), 6.62 (d, *J* = 7.9 Hz, 1H), 4.83 (d, *J* = 15.9 Hz, 1H), 4.64 (d, *J* = 5.6 Hz, 1H), 4.57 (d, *J* = 15.9 Hz, 1H), 4.44 (m 1H), 3.99 (m, 2H), 3.01 (d, *J* = 13.3 Hz, 1H), 2.86 (d, *J* = 13.3 Hz, 1H), 2.30 (s, 3H), 0.96 (s, 3H).

^{13}C{^{1}H} NMR (101 MHz, DMSO-*d*$_6$) δ 212.8, 210.8, 177.9, 140.7, 135.5, 134.5, 132.3, 130.2, 129.7, 128.5, 128.4 128.1, 127.3, 127.2, 127.1, 125.8 (q, *J* = 287.9 Hz, 1C), 124.8, 109.5, 71.0, 62.0, 58.7 (q, *J* = 30.7 Hz, 1C), 57.8, 51.3, 42.7, 41.6, 20.6, 14.4.

HRMS (ESI) *m/z*: $[M+H]^+$ calcd for $C_{31}H_{28}F_3N_2O_3$, 533.2047, found: 533.2048.

1',5-dibenzyl-5,5',7'-trimethyl-3-(trifluoromethyl)-2,3,3a,6a-tetrahydro-4H-spiro[cyclopenta[c]pyr role-1,3'-indoline]-2',4,6(5H)-trione (3qa)

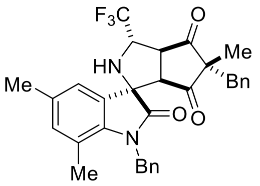

The product was purified by flash column chromatography (petroleum ether / ethyl acetate = 15:1 as the eluent);

White solid; 87.4 mg, 80% yield; 83:17 *dr*, mp.190.7-191.6 °C;

^{1}H NMR (400 MHz, CDCl$_3$) δ 7.32 – 7.19 (m, 5H), 7.16 (m, 1H), 7.08 – 6.99 (m, 2H), 6.93 (dd, *J* = 6.4, 2.9 Hz, 2H), 6.90 (d, *J* = 1.9 Hz, 1H), 6.72 (d, *J* = 1.9 Hz, 1H), 5.00 (d, *J* = 16.9 Hz, 1H), 4.73 (d, *J* = 16.9 Hz, 1H), 4.58 – 4.34 (m, 1H), 3.04 (d, *J* = 12.0 Hz, 1H), 2.86 (d, *J* = 12.9 Hz, 1H), 2.74 (d, *J* = 12.9 Hz, 1H), 2.56 (dd, *J* = 12.0, 4.6 Hz, 1H), 2.19 (s, 4H), 2.05 (s, 3H), 1.09 (s, 3H).

^{13}C{^{1}H} NMR (101 MHz, CDCl$_3$) δ 214.8, 212.1, 179.0, 138.7, 136.9, 134.9, 134.8, 133.7, 129.5, 129.0, 129.0, 128.5, 128.0, 127.4, 125.7, 125.1 (q, *J* = 279.8Hz, 1C), 123.0, 120.4, 70.8, 63.1,59.6 (q, *J* = 31.8Hz, 1C), 59.5, 52.9, 46.3, 45.2, 20.8, 18.4, 17.9.

HRMS (ESI) *m/z*: $[M+H]^+$ calcd for $C_{32}H_{30}F_3N_2O_3$, 547.2203, found: 547.2199.

1'-benzyl-5-methyl-5-(3-methylbenzyl)-3-(trifluoromethyl)-2,3,3a,6a-tetrahydro-4H-spiro[cyclopenta[c]pyrrole-1,3'-indoline]-2',4,6(5H)-trione (3ab)

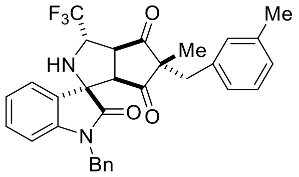

The product was purified by flash column chromatography (petroleum ether /ethyl acetate =15:1 as the eluent);

White solid; 102.1 mg, 96% yield; 84:16 *dr*, mp 190.6-191.4 °C;

^{1}H NMR (400 MHz, CDCl$_3$) δ 7.38 (dd, *J* = 7.4, 1.3 Hz, 1H), 7.31 (d, *J* = 4.3 Hz, 4H), 7.29 – 7.21 (m, 2H), 7.17 (m, 1H), 7.11 (m, 1H), 7.08 (d, *J* = 7.4 Hz, 1H), 6.86 (s, 1H), 6.82 (d, *J* = 7.6 Hz, 1H), 6.75 (d, *J* = 7.8 Hz, 1H), 4.85 (d, *J* = 16.0 Hz, 1H), 4.65 (d, *J* = 5.6 Hz, 1H), 4.59 (d, *J* =

16.0 Hz, 1H), 4.43 (q, J = 6.9 Hz, 1H), 3.97 (d, J = 2.3 Hz, 2H), 2.96 (d, J = 13.3 Hz, 1H), 2.80 (d, J = 13.3 Hz, 1H), 2.25 (s, 3H), 0.96 (s, 3H).

$^{13}C\{^1H\}$ NMR (101 MHz, CDCl$_3$) δ 213.8, 211.8, 178.9, 144.1, 138.1 136.4 135.4, 131.8, 130.6, 129.6, 129.4, 129.0, 128.8 128.3, 128.2, 128.2,126.6 (q, J = 279.8 Hz, 1C), 125.1, 124.1, 110.7, 71.9, 63.0, 59.7 (q, J = 30.6 Hz, 1C), 58.8, 52.3, 43.7, 42.6, 21.9, 15.5.

HRMS (ESI) *m/z*: $[M+H]^+$ calcd for $C_{31}H_{28}F_3N_2O_3$, 533.2047, found: 533.2046.

1'-benzyl-5-methyl-5-(4-methylbenzyl)-3-(trifluoromethyl)-2,3,3a,6a-tetrahydro-4H-spiro[cyclopenta[c]pyrrole-1,3'-indoline]-2',4,6(5H)-trione (3ac)

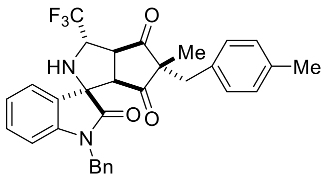

The product was purified by flash column chromatography (petroleum ether / ethyl acetate =15:1 as the eluent);

White solid; 100.0 mg, 94% yield; 82:18 *dr*, mp 131.2-132.0 °C;

^1H NMR (400 MHz, DMSO-d_6)δ 7.31 – 7.21 (m, 3H), 7.21 – 7.14 (m, 3H), 7.11 (m, 1H), 7.04 – 6.94 (m, 3H), 6.80 (d, J = 7.8 Hz, 2H), 6.55 (d, J = 7.8 Hz, 1H), 4.84 (d, J = 15.7 Hz, 1H), 4.54 – 4.46 (m, 1H), 4.43 (d, J = 15.8 Hz, 1H),3.10 (d, J = 12.0 Hz, 1H), 2.83 (d, J = 13.0 Hz, 1H), 2.72 (d, J = 13.0 Hz, 1H), 2.64 (dd, J = 12.0, 4.6 Hz, 1H), 2.25 (s, 3H), 2.21 (s, 1H), 1.15 (s, 3H).

$^{13}C\{^1H\}$ NMR (101 MHz, DMSO-d_6)/ δ 214.7, 212.2, 178.1, 143.0, 137.8, 134.9, 131.5, 130.3, 129.6, 129.4, 128.9, 128.1, 127.9, 127.3,125.1 (q, J = 276.7 Hz, 1C) ,123.9, 123.7, 110.0, 71.3, 63.2, 59.7 (q, J = 31.5 Hz, 1C), 59.2, 52.8, 45.9 43.9, 21.2, 17.8.

HRMS (ESI) *m/z*: $[M+H]^+$ calcd for $C_{31}H_{28}F_3N_2O_3$, 533.2047, found: 533.2043.

1'-benzyl-5-(3-methoxybenzyl)-5-methyl-3-(trifluoromethyl)-2,3,3a,6a-tetrahydro-4H-spiro[cyclopenta[c]pyrrole-1,3'-indoline]-2',4,6(5H)-trione (3ad)

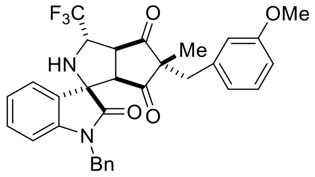

The product was purified by flash column chromatography (petroleum ether / ethyl acetate =15:1 as the eluent);

White solid; 103.1 mg, 94% yield; 85:15 *dr*, mp 182.6-183.4 °C;

^1H NMR (400 MHz, CDCl$_3$) δ 7.26 (dd, J = 8.0, 6.5 Hz, 2H), 7.23 – 7.17 (m, 2H), 7.17 – 7.14 (m, 2H), 7.11 (m, 2H), 6.98 (m, 1H), 6.79 – 6.69 (m, 1H), 6.55 (d, J = 7.8 Hz, 1H), 6.49 (m, 1H), 6.45 (t, J = 2.1 Hz, 1H), 4.84 (d, J = 15.7 Hz, 1H), 4.54 – 4.45 (m, 1H), 4.42 (d, J = 15.7 Hz, 1H), 3.65 (s, 3H), 3.11 (d, J = 12.1 Hz, 1H), 2.84 (d, J = 12.8 Hz, 1H), 2.72 (d, J = 12.8 Hz, 1H), 2.66 (dd, J = 12.1, 4.5 Hz, 1H), 2.19 (s, 1H), 1.17 (s, 3H).

$^{13}C\{^1H\}$ NMR (101 MHz, CDCl$_3$) δ 214.5, 212.1, 178.1, 159.9, 143.0, 136.2, 134.9, 130.3, 130.1 128.9, 128.0, 127.9, 127.3,125.1 (q, J = 276.8 Hz, 1C), 124.3, 123.9, 121.7, 114.8, 113.8, 110.0, 71.3, 63.0, 59.6 (q, J = 31.5 Hz, 1C), 59.4, 55.4, 53.0, 46.4, 43.9, 18.0.

HRMS (ESI) *m/z*: $[M+H]^+$ calcd for $C_{31}H_{28}F_3N_2O_4$,549.1996, found: 549.2004.

1'-benzyl-5-(4-fluorobenzyl)-5-methyl-3-(trifluoromethyl)-2,3,3a,6a-tetrahydro-4H-spiro[cyclopenta[c]pyrrole-1,3'-indoline]-2',4,6(5H)-trione (3ae)

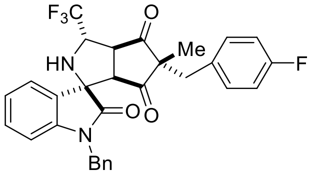

The product was purified by flash column chromatography (petroleum ether / ethyl acetate =15:1 as the eluent);

White solid; 102.9 mg, 96% yield; 88:12 *dr*, mp 172.9-173.7 °C;

^{1}H NMR (400 MHz, $CDCl_3$) δ 7.33 – 7.22 (m, 3H), 7.22 – 7.07 (m, 4H), 7.04 – 6.96 (m, 1H), 6.95 – 6.81 (m, 4H), 6.56 (d, *J* = 7.8 Hz, 1H), 4.85 (d, *J* = 15.7 Hz, 1H), 4.52 (m, 1H), 4.44 (d, *J* = 15.7 Hz, 1H), 3.15 (d, *J* = 12.1 Hz, 1H), 2.87 – 2.81 (m, 1H), 2.81 – 2.76 (m, 1H), 2.74 (d, *J* = 13.2 Hz, 1H), 2.25 (s, 1H), 1.14 (s, 3H).

^{13}C{^{1}H} NMR (101 MHz, $CDCl_3$) δ 214.3, 211.8, 178.1, 162.4 (d, *J* = 248.3 Hz, 1C), 143.0, 134.8, 131.2 (d, *J* = 8.0 Hz, 1C), 130.4 (d, *J* = 3.0 Hz, 1C), 129.0, 127.9, 127.3, 125.0 (q, *J* = 279.4 Hz, 1C), 124.4, 124.0, 115.9 (d, *J* = 21.3 Hz, 1C), 110.1, 71.4, 62.9, 62.8, 59.8 (q, *J* = 31.9 Hz, 1C), 59.2, 52.7, 44.8, 43.9, 17.6.

HRMS (ESI) *m/z*: $[M+H]^+$ calcd for $C_{30}H_{25}F_4N_2O_3$, 537.1796, found: 537.1802.

1'-benzyl-5-(2-chlorobenzyl)-5-methyl-3-(trifluoromethyl)-2,3,3a,6a-tetrahydro-4H-spiro[cyclopenta[c]pyrrole-1,3'-indoline]-2',4,6(5H)-trione(3af)

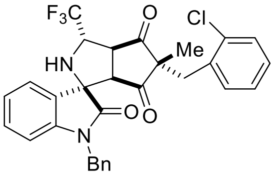

The product was purified by flash column chromatography (petroleum ether / ethyl acetate =15:1 as the eluent);

White solid; 83.9 mg, 76% yield; 71:29 *dr*, mp 174.8-175.6 °C;

^{1}H NMR (400 MHz, $CDCl_3$) δ 7.31 – 7.22 (m, 3H), 7.16 (m, 4H), 7.05 – 6.96 (m, 1H), 6.90 (d, *J* = 2.4 Hz, 2H), 6.89 (s, 2H), 6.56 (d, *J* = 7.8 Hz, 1H), 4.85 (d, *J* = 15.7 Hz, 1H), 4.52 (m, 1H), 4.44 (d, *J* = 15.7 Hz, 1H), 3.15 (d, *J* = 12.1 Hz, 1H), 2.96 – 2.62 (m, 3H), 2.25 (s, 1H), 1.15 (s, 3H).

^{13}C{^{1}H} NMR (101 MHz, $CDCl_3$) δ 214.3, 211.9, 178.1, 163.7, 161.2, 143.1, 134.9, 131.3, 131.2, 130.4, 130.4, 129.07, 127.9, 127.3, 125.0 (q, *J* = 279.4 Hz, 1C), 124.4, 124.0, 116.0, 115.8, 110.1, 71.4, 62.9, 59.8 (q, *J* = 31.9 Hz, 1C), 59.2, 52.7, 44.8, 44.0, 17.6.

HRMS (ESI) *m/z*: $[M+H]^+$ calcd for $C_{30}H_{25}{}^{35}Cl\,F_3N_2O_3$, 553.1500, found: 553.1500; calcd for $C_{30}H_{24}{}^{37}Cl\,F_3N_2O_3$, 555.1486, found: 555.1485.

1'-benzyl-5-(4-chlorobenzyl)-5-methyl-3-(trifluoromethyl)-2,3,3a,6a-tetrahydro-4H-spiro[cyclopenta[c]pyrrole-1,3'-indoline]-2',4,6(5H)-trione (3ag)

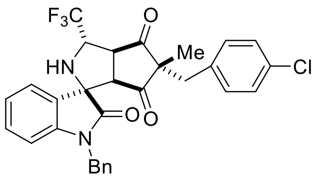

The product was purified by flash column chromatography (petroleum ether / ethyl acetate =15:1 as the eluent);

White solid; 103.8 mg, 94% yield; 86:14 *dr*, mp 198.1-198.8 °C;

^{1}H NMR (400 MHz, CDCl$_3$) δ 7.26 (dd, *J* = 7.9, 6.5 Hz, 3H), 7.23 – 7.07 (m, 6H), 7.00 (m, 1H), 6.90 – 6.79 (m, 2H), 6.56 (d, *J* = 7.8 Hz, 1H), 4.84 (d, *J* = 15.7 Hz, 1H), 4.65 – 4.49 (m, 1H), 4.44 (d, *J* = 15.7 Hz, 1H), 3.20 (d, *J* = 12.1 Hz, 1H), 2.86 (dd, *J* = 12.1, 4.6 Hz, 1H), 2.83 – 2.64 (m, 2H), 2.27 (s, 1H), 1.14 (s, 3H).

^{13}C{^{1}H}NMR (101 MHz, CDCl$_3$) δ 214.0, 211.6, 178.1, 143.0 134.8, 134.0, 133.0, 131.0, 130.4, 129.0 129.0, 127.9 127.9, 127.3,125.0 (q, *J* = 276.7 Hz, 1C) 124.4, 124.0, 110.1, 71.4, 62.7, 59.8 (q, *J* = 31.0 Hz, 1C), 59.1, 52.6 44.7, 43.9, 17.5.

HRMS (ESI) *m/z*: [M+H]$^+$ calcd for $C_{30}H_{25}{}^{35}Cl\ F_3N_2O_3$, 553.1500, found: 553.1502; calcd for $C_{30}H_{24}{}^{37}Cl\ F_3N_2O_3$, 555.1486, found: 555.1486.

1'-benzyl-5-(3-bromobenzyl)-5-methyl-3-(trifluoromethyl)-2,3,3a,6a-tetrahydro-4H-spiro[cyclopenta[c]pyrrole-1,3'-indoline]-2',4,6(5H)-trione (3ah)

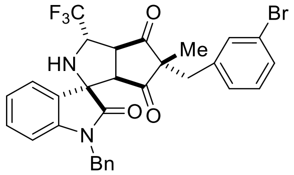

The product was purified by flash column chromatography (petroleum ether / ethyl acetate =15:1 as the eluent);

White solid; 100.1 mg, 84% yield; 84:16 *dr*, mp 175.0-175.6 °C;

^{1}H NMR (400 MHz, CDCl$_3$) δ 7.40 – 7.32 (m, 1H), 7.31 – 7.23 (m, 3H), 7.23 – 7.11 (m, 4H), 7.11 – 7.04 (m, 2H), 7.01 (m, 1H), 6.84 (d, *J* = 7.9 Hz, 1H), 6.57 (d, *J* = 7.7 Hz, 1H), 4.85 (d, *J* = 15.7 Hz, 1H), 4.53 (m, 1H), 4.44 (d, *J* = 15.7 Hz, 1H), 3.21 (d, *J* = 12.0 Hz, 1H), 2.88 – 2.76 (m, 2H), 2.71 (d, *J* = 13.0 Hz, 1H), 2.43 – 2.01 (s, 1H), 1.15 (s, 3H).

^{13}C{^{1}H} NMR (101 MHz, CDCl$_3$) δ 214.3, 211.9, 178.1, 163.7, 161.2, 143.1, 134.9, 131.3, 131.2, 130.4, 130.4, 129.0, 127.9, 127.3, 125.0 (q, *J* = 276.6 Hz, 1C), 124.4, 124.0, 116.0, 115.8, 110.1, 71.4, 62.9, 59.8 (q, *J* = 31.6 Hz, 1C), 59.2, 52.7, 44.8, 44.0, 17.6.

HRMS (ESI) *m/z*: [M+H]$^+$ calcd for $C_{30}H_{25}{}^{79}BrF_3N_2O_3$, 597.0995, found, 597.0995, calcd for $C_{30}H_{24}{}^{81}BrF_3N_2O_3$, 599.0980, found: 599.0981.

1'-benzyl-5-(4-bromobenzyl)-5-methyl-3-(trifluoromethyl)-2,3,3a,6a-tetrahydro-4H-spiro[cyclopenta[c]pyrrole-1,3'-indoline]-2',4,6(5H)-trione (3ai)

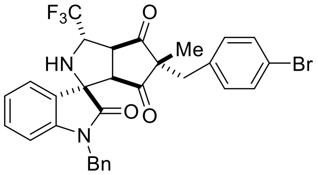

The product was purified by flash column chromatography (petroleum ether / ethyl acetate =15:1 as the eluent);

White solid; 112.1 mg, 94% yield; 85:15 *dr*, mp 176.1-176.9 °C;

^{1}H NMR (400 MHz, DMSO-d_6) δ 7.47 (d, *J* = 8.0 Hz, 2H), 7.41 (d, *J* = 7.3 Hz, 1H), 7.31 (d, *J* = 4.3 Hz, 4H), 7.26 (m, 2H), 7.12 (m, 1H), 7.03 (d, *J* = 8.0 Hz, 2H), 6.75 (d, *J* = 7.8 Hz, 1H), 4.84 (d, *J* = 15.9 Hz, 1H), 4.68 (d, *J* = 5.5 Hz, 1H), 4.60 (d, *J* = 15.9 Hz, 1H), 4.45 (m, 1H), 4.29 (dd, *J* = 12.1, 4.4 Hz, 1H), 4.15 (d, *J* = 12.0 Hz, 1H), 3.08 – 2.78 (m, 2H), 0.92 (s, 3H).

^{13}C{^{1}H} NMR (101 MHz, DMSO-d_6) δ 212.4, 210.6, 178.0, 143.1, 135.5, 133.9, 132.6, 130.9, 129.6, 128.6, 128.4, 127.4, 127.2,125.7 (q, *J* = 272.1 Hz, 1C), 124.2, 123.2, 120.5, 109.7, 71.0, 61.6, 58.7 (q, *J* = 30.5 Hz, 1C), 57.7, 51.0, 42.8, 39.9, 13.8.

HRMS (ESI) *m/z*: $[M+H]^+$ calcd for $C_{30}H_{25}{}^{79}BrF_3N_2O_3$, 597.0995, found, 597.0995, calcd for $C_{30}H_{24}{}^{81}BrF_3N_2O_3$, 599.0980, found: 599.0978.

1'-benzyl-5-methyl-5-(naphthalen-1-ylmethyl)-3-(trifluoromethyl)-2,3,3a,6a-tetrahydro-4H-spiro[cyclopenta[c]pyrrole-1,3'-indoline]-2',4,6(5H)-trione (3aj)

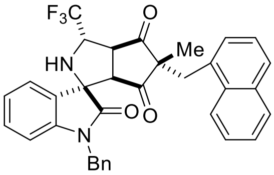

The product was purified by flash column chromatography (petroleum ether / ethyl acetate =15:1 as the eluent);

White solid; 111.3 mg, 98% yield; 89:11 *dr*, mp 190.2-191.1 °C;

^{1}H NMR (400 MHz, DMSO-d_6) δ 8.07 – 8.00 (m, 1H), 7.97 – 7.89 (m, 1H), 7.88 (d, *J* = 8.2 Hz, 1H), 7.51 (dd, *J* = 6.5, 3.3 Hz, 2H), 7.45 (m, 1H), 7.37 – 7.20 (m, 8H), 7.10 (m, 1H), 6.75 (d, *J* = 7.7 Hz, 1H), 4.86 (d, *J* = 16.0 Hz, 1H), 4.65 (d, *J* = 5.5 Hz, 1H), 4.59 (d, *J* = 16.0 Hz, 1H), 4.44 (t, *J* = 7.0 Hz, 1H), 3.81 (s, 2H), 3.60 – 3.44 (m, 2H), 1.08 (s, 3H).

^{13}C{^{1}H} NMR (101 MHz, DMSO-d_6) δ 213.2, 211.2, 178.6, 143.1, 135.4, 133.3, 132.0, 131.0, 129.6, 129.1, 128.6, 128.6, 128.4, 128.0, 127.4, 127.2, 126.0, 125.8, 125.5 (q, *J* = 276.6 Hz, 1C), 125.1, 124.0, 123.9, 123.2, 109.7, 71.0, 62.3, 58.7 (q, *J* = 30.6 Hz, 1C), 58.3, 51.7, 42.8, 37.8, 15.5.

HRMS (ESI) *m/z*: $[M+H]^+$ calcd for $C_{34}H_{28}F_3N_2O_3$, 569. 2047, found: 569.2072.

1'-benzyl-5-methyl-5-(naphthalen-2-ylmethyl)-3-(trifluoromethyl)-2,3,3a,6a-tetrahydro-4H-spiro[cyclopenta[c]pyrrole-1,3'-indoline]-2',4,6(5H)-trione (3ak)

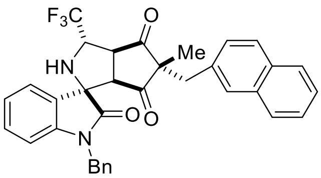

The product was purified by flash column chromatography (petroleum ether / ethyl acetate =15:1 as the eluent);

White solid; 112.6 mg, 99% yield; 86:14 *dr*, mp 138.2-138.9 °C

^{1}H NMR (400 MHz, $CDCl_3$) δ 7.78 – 7.69 (m, 1H), 7.66 (dd, *J* = 9.0, 5.4 Hz, 2H), 7.39 (dd, *J* = 6.3, 2.9 Hz, 3H), 7.25 (dd, *J* = 8.1, 6.5 Hz, 2H), 7.21 – 7.12 (m, 4H), 7.09 (m, 1H), 7.00 (dd, *J* = 8.4, 1.8 Hz, 1H), 6.93 (m, 1H), 6.53 (d, *J* = 7.8 Hz, 1H), 4.84 (d, *J* = 15.7 Hz, 1H), 4.50 (m, *J* = 11.3, 5.6 Hz, 1H), 4.41 (d, *J* = 15.7 Hz, 1H), 3.08 (d, *J* = 12.0 Hz, 1H), 3.00 (d, *J* = 13.0 Hz, 1H), 2.92 (d, *J* = 13.0 Hz, 1H), 2.75 (dd, *J* = 12.1, 4.7 Hz, 1H), 2.12 (s, 1H), 1.21 (s, 3H).

$^{13}C\{^1H\}$ NMR (101 MHz, $CDCl_3$) δ 214.5, 212.1, 178.1, 143.0, 134.9, 133.3, 132.7, 132.1, 130.3, 128.9, 128.7, 128.5, 127.9, 127.8, 127.8, 127.4, 127.3, 126.7, 126.4, 125.0 (q, *J* = 276.7 Hz, 1C), 123.9, 123.6, 110.0, 71.3, 63.2, 59.7 (q, *J* = 29.1 Hz, 1C), 59.2, 52.8 46.1, 43.9, 17.9.

HRMS (ESI) *m/z*: $[M+H]^+$ calcd for $C_{34}H_{28}F_3N_2O_3$, 569. 2047, found: 569.2049.

1',5-dibenzyl-5-ethyl-3-(trifluoromethyl)-2,3,3a,6a-tetrahydro-4H-spiro[cyclopenta[c] pyrrole-1,3'-indoline]-2',4,6(5H)-trione (3aI)

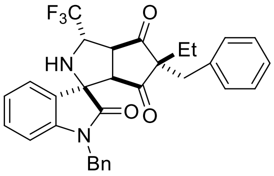

The product was purified by flash column chromatography (petroleum ether / ethyl acetate =15:1 as the eluent);

White solid; 73.4 mg, 69% yield; 88:12 *dr*, mp 171.2-171.8 °C;

^{1}H NMR (400 MHz, DMSO-*d*$_6$) δ 7.34 (m , 5H), 7.30 – 7.20 (m, 5H), 7.11 (m, 1H), 7.08 – 6.98 (m, 2H), 6.73 (d, *J* = 7.8 Hz, 1H), 4.91 (d, *J* = 16.1 Hz, 1H), 4.64 (d, *J* = 5.2 Hz, 1H), 4.59 (d, *J* = 15.8 Hz, 1H), 4.47 (m, 1H), 4.01 (d, *J* = 12.4 Hz, 1H), 3.87 (dd, *J* = 12.5, 5.2 Hz, 1H), 3.09 (d, *J* = 13.5 Hz, 1H), 2.99 (d, *J* = 13.5 Hz, 1H), 1.54 (m, *J* = 7.5 Hz, 2H), 0.87 (t, *J* = 7.6 Hz, 3H).

$^{13}C\{^1H\}$ NMR (101 MHz, DMSO-*d*$_6$) δ 212.3, 210.5, 177.7, 143.2, 135.6, 134.5, 130.1, 129.6, 128.9, 128.4, 128.2, 127.3, 127.2, 127.2, 125.5 (q, *J* = 276.8 Hz, 1C), 124.1, 123.0, 109.6, 70.1, 66.2, 58.6 (q, *J* = 30.5 Hz, 1C), 57.8, 51.3, 42.8, 40.3 22.1, 8.1.

HRMS (ESI) *m/z*: $[M+H]^+$ calcd for $C_{31}H_{28}F_3N_2O_3$, 533. 2047, found: 533.2048.

1'-benzyl-5-(3-bromobenzyl)-5-ethyl-3-(trifluoromethyl)-2,3,3a,6a-tetrahydro-4H-spiro[cyclo penta[c]pyrrole-1,3'-indoline]-2',4,6(5H)-trione(3am)

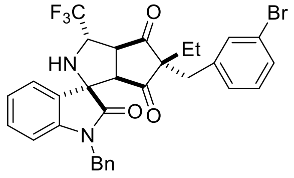

The product was purified by flash column chromatography (petroleum ether / ethyl acetate =15:1 as the eluent);

White solid; 102.5 mg, 84% yield; 72:28 *dr*, mp 166.5-167.4 °C;

^{1}H NMR (400 MHz, DMSO-*d*$_6$) δ 7.46 (dd, *J* = 7.9, 2.0 Hz, 1H), 7.42 – 7.37 (m, 1H), 7.37 – 7.28 (m, 5H), 7.25 (dd, *J* = 8.9, 7.2 Hz, 3H), 7.12 (m, 1H), 7.07 (d, *J* = 7.7 Hz, 1H), 6.73 (d, *J* = 7.8 Hz, 1H), 4.90 (d, *J* = 16.0 Hz, 1H), 4.65 (d, *J* = 5.3 Hz, 1H), 4.59 (d, *J* = 16.0 Hz, 1H), 4.54 – 4.40 (m, 1H), 4.30 – 4.01 (m, 2H), 3.10 (d, *J* = 13.6 Hz, 1H), 3.01 (d, *J* = 13.6 Hz, 1H), 1.49 (m, *J* = 9.8, 5.0 Hz, 2H), 0.84 (t, *J* = 7.5 Hz, 3H).

^{13}C{^{1}H} NMR (101 MHz, DMSO-*d*$_6$) δ 213.8, 211.3, 178.0, 143.0, 136.9, 134.8, 132.6, 131.2, 130.5, 130.4, 129.0, 128.3, 127.9, 127.9, 127.3, 125.0 (q, *J* = 279.4 Hz, 1C), 124.4, 124.0, 122.9, 110.2, 77.5, 77.2, 76.8, 71.4, 62.6, 59.7 (q, *J* = 31.7 Hz, 1C), 59.1, 52.7, 44.8, 44.0, 17.5.

HRMS (ESI) *m/z*: [M+H]$^+$ calcd for $C_{31}H_{27}{}^{79}BrF_3N_2O_3$, 611.1152, found: 611.1149; calcd for $C_{31}H_{26}{}^{81}BrF_3N_2O_3$, 613.1136, found: 613.1139.

1'-benzyl-5-(4-bromobenzyl)-5-ethyl-3-(trifluoromethyl)-2,3,3a,6a-tetrahydro-4H-spiro[cyclopenta[c]pyrrole-1,3'-indoline]-2',4,6(5H)-trione(3an)

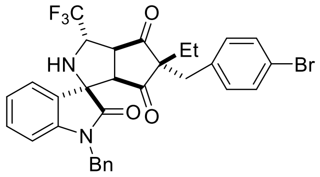

The product was purified by flash column chromatography (petroleum ether / ethyl acetate =15:1 as the eluent);

White solid; 107.3 mg, 88% yield; 84:16 *dr*, mp. 120.1-120.7 °C;

^{1}H NMR (400 MHz, DMSO-*d*$_6$) δ^{1}H NMR (400 MHz, DMSO-*d*$_6$) δ 7.48 (d, *J* = 8.0 Hz, 2H), 7.40 (d, *J* = 7.3 Hz, 1H), 7.39 – 7.28 (m, 4H), 7.25 (m, 2H), 7.11 (m, 1H), 7.02 (d, *J* = 8.1 Hz, 2H), 6.73 (d, *J* = 7.8 Hz, 1H), 4.90 (d, *J* = 16.0 Hz, 1H), 4.65 (d, *J* = 5.3 Hz, 1H), 4.58 (d, *J* = 16.0 Hz, 1H), 4.48 (m, 1H), 4.19 (d, *J* = 12.5 Hz, 1H), 4.14 (dd, *J* = 12.5, 4.7 Hz, 1H), 3.07 (d, *J* = 13.6 Hz, 1H), 3.00 (d, *J* = 13.6 Hz, 1H), 1.48 (dt, *J* = 11.7, 7.0 Hz, 2H), 0.83 (t, *J* = 7.5 Hz, 3H).

^{13}C{^{1}H} NMR (101 MHz, DMSO-*d*$_6$) δ 211.8, 210.1, 177.7, 143.2, 135.6, 133.9, 132.4, 131.0, 129.6, 128.8, 128.4, 127.3, 127.1,125.5 (q, *J* = 276.9 Hz, 1C) 124.2, 123.0, 120.6, 109.6, 70.0, 66.0, 58.6 (q, *J* = 30.8 Hz, 1C), 57.6, 51.0, 42.8, 38.9, 21.5, 8.1.

HRMS (ESI) *m/z*: [M+H]$^+$ calcd for $C_{31}H_{27}{}^{79}BrF_3N_2O_3$, 611. 1152, found: 611.1153; calcd for $C_{31}H_{26}{}^{81}BrF_3N_2O_3$, 613.1136, found: 613.1138.

6. General experimental procedures for synthesis of compounds 5.

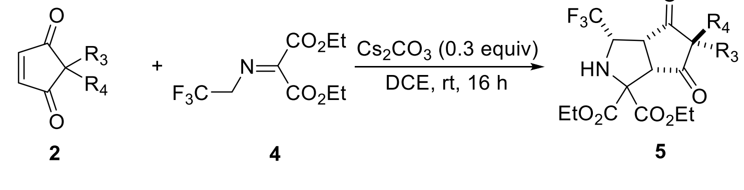

In a dry reaction tube equipped with a magnetic stirring bar, **2** (60.2 mg, 0.3 mmol, 3.0 equiv), and 2-((2,2,2-trifluoroethyl)imino) malonate **4** (0.1 mmol), Cs_2CO_3 (31.8 mg, 0.09 mmol), DCE (3.0 mL) were added in sequence, Then the mixture was stirred at room temperature for 24 h.

After completion, the reaction mixture was directly purified by flash chromatography on silica gel (petroleum ether / ethyl acetate = 15:1 - 10:1) to give the corresponding products **5**.

Diethyl 5-benzyl-5-methyl-4,6-dioxo-3-(trifluoromethyl)hexahydrocyclopenta[c]pyrrole-1,1(2H)-dicarboxylate(5a)

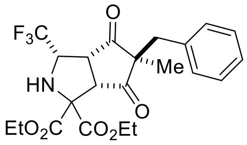

The product was purified by flash column chromatography (petroleum ether / ethyl acetate =10:1 as the eluent);

White solid; 77.8 mg, 57% yield; > 20:1 *dr*, mp 162.1-162.9 °C;

^{1}H NMR (400 MHz, $CDCl_3$) δ 7.25 – 7.12 (m, 3H), 6.98 – 6.86 (m, 2H), 4.26 (q, 2H), 4.21 – 4.06 (m, 2H), 3.63 – 3.50 (m, 1H), 3.08 (d, *J* = 10.1 Hz, 1H), 2.99 (d, *J* = 12.9 Hz, 1H), 2.86 (t, *J* = 9.7 Hz, 1H), 2.83 – 2.76 (m, 2H), 1.26 (t, *J* = 7.2 Hz, 3H), 1.20 – 1.12 (m, 6H).

^{13}C{^{1}H} NMR (101 MHz, $CDCl_3$) δ 213.6, 212.9, 168.7, 166.2, 135.3, 129.6, 128.9 127.5, 123.6 (q, *J* = 280.5 Hz, 1C), 74.7, 63.0, 62.4, 61.4 (q, *J* = 32.6 Hz, 1C), 60.7, 56.2, 49.8, 44.2, 19.5, 13.9, 13.8.

HRMS (ESI) *m/z*: $[M+H]^+$ calcd for $C_{22}H_{25}F_3NO_6$, 456.1628, found: 456.1635.

Diethyl 5-methyl-5-(3-methylbenzyl)-4,6-dioxo-3-(trifluoromethyl)hexahydrocyclopenta[c] pyrrole-1,1(2H)-dicarboxylate(5b)

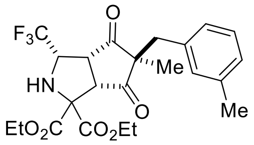

The product was purified by flash column chromatography (petroleum ether / ethyl acetate =10:1 as the eluent);

White solid; 70.4 mg, 50% yield; > 20:1 *dr*, mp 136.9-137.7 °C;

^{1}H NMR (400 MHz, $CDCl_3$) δ 7.13 (m, 1H), 7.04 (d, *J* = 7.6 Hz, 1H), 6.86 – 6.72 (m, 2H), 4.38 – 4.27 (m, 2H), 4.27 – 4.05 (m, 2H), 3.71 – 3.52 (m, 1H), 3.12 (d, *J* = 10.1 Hz, 1H), 3.01 (d, *J* = 12.9 Hz, 1H), 2.94 (t, *J* = 9.9 Hz, 1H), 2.87 (d, *J* = 4.1 Hz, 1H), 2.82 (d, *J* = 12.9 Hz, 1H), 2.28 (s, 3H), 1.33 (t, *J* = 7.1 Hz, 3H), 1.28 – 1.13 (m, 6H).

^{13}C{^{1}H} NMR (101 MHz, $CDCl_3$) δ 213.6, 212.9, 168.7, 166.3, 138.5, 135.2, 130.3, 128.7, 128.3, 126.5, 123.6 (q, *J* = 280.5 Hz, 1C), 74.6, 63.0, 62.4, 61.3 (q, *J* = 32.5 Hz, 1C), 60.7, 56.3, 49.8, 44.4, 21.3, 19.4, 13.9 (2C).

HRMS (ESI) *m/z*: $[M+H]^+$ calcd for $C_{23}H_{27}F_3NO_6$, 470.1785, found: 470.1790.

Diethyl 5-methyl-5-(4-methylbenzyl)-4,6-dioxo-3-(trifluoromethyl)hexahydrocyclopenta[c] pyrrole-1,1(2H)-dicarboxylate(5c)

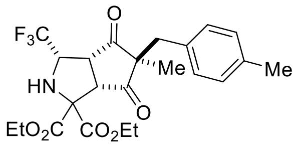

The product was purified by flash column chromatography (petroleum ether / ethyl acetate =10:1 as the eluent);

White solid; 59.1 mg, 42% yield; > 20:1 *dr*, mp 113.3-114.2 ℃;

^{1}H NMR (400 MHz, $CDCl_3$) δ 7.04 (d, *J* = 7.8 Hz, 2H), 6.96 – 6.74 (m, 2H), 4.33 (m, 2H), 4.28 – 4.09 (m, 2H), 3.63 (m, 1H), 3.20 (d, *J* = 10.2 Hz, 1H), 3.00 (d, *J* = 12.9 Hz, 1H), 2.93 (t, *J* = 9.8 Hz, 1H), 2.88 (s, 1H), 2.83 (d, *J* = 13.0 Hz, 1H), 2.29 (s, 3H), 1.33 (t, *J* = 7.2 Hz, 3H), 1.29 – 1.13 (m, 6H).

^{13}C{^{1}H} NMR (101 MHz, $CDCl_3$) δ 213.8, 213.0, 168.7, 166.3, 137.2, 132.2, 129.5, 129.4, 123.7 (q, *J* = 278.1 Hz, 1C), 74.8, 63.0, 62.4,61.4 (q, *J* = 32.3 Hz, 1C), 60.7, 56.2, 49.8, 43.8, 21.1, 19.4, 13.9 (2C).

HRMS (ESI) *m/z*: $[M+H]^+$ calcd for $C_{23}H_{27}F_3NO_6$, 470.1785, found: 470.1788.

Diethyl 5-(3-methoxybenzyl)-5-methyl-4,6-dioxo-3-(trifluoromethyl)hexahydrocyclopenta[c] pyrrole-1,1(2H)-dicarboxylate(5d)

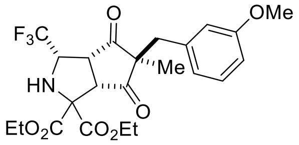

The product was purified by flash column chromatography (petroleum ether / ethyl acetate =10:1 as the eluent);

White solid; 78.6 mg, 54% yield; > 20:1 *dr*, mp 110.8-111.4 ℃;

^{1}H NMR (400 MHz, $CDCl_3$) δ 7.15 (m, 1H), 6.77 (dd, *J* = 8.2, 2.6 Hz, 1H), 6.57 (d, *J* = 7.5 Hz, 1H), 6.53 (m, 1H), 4.32 (m, 2H), 4.28 – 4.12 (m, 2H), 3.75 (d, *J* = 1.9 Hz, 3H), 3.63 (m, 1H), 3.19 (d, *J* = 10.2 Hz, 1H), 3.03 (d, *J* = 12.7 Hz, 1H), 2.97 (t, *J* = 9.9 Hz, 1H), 2.85 (d, *J* = 12.8 Hz, 2H), 1.32 (m, 3H), 1.27 – 1.18 (m, 6H).

^{13}C{^{1}H} NMR (101 MHz, $CDCl_3$) δ 213.8, 212.9, 168.8, 166.4, 159.8, 137.0, 129.9, 123.7 (q, *J* = 280.5 Hz, 1C), 121.9, 115.2, 113.3, 74.8, 63.1, 62.5, 61.5 (q, *J* = 32.6Hz, 1C), 60.7, 56.4, 55.3, 50.0, 44.4, 19.7, 14.1, 14.0.

HRMS (ESI) *m/z*: $[M+H]^+$ calcd for $C_{23}H_{27}F_3NO_7$, 486.1734, found: 486.1714.

Diethyl 5-(4-fluorobenzyl)-5-methyl-4,6-dioxo-3-(trifluoromethyl)hexahydrocyclopenta [c] pyrrole-1,1(2H)-dicarboxylate(5e)

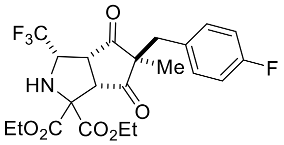

The product was purified by flash column chromatography (petroleum ether / ethyl acetate =10:1 as the eluent);

White solid; 85.2 mg, 60% yield; > 20:1 *dr*, mp 152.6-153.4 ℃;

^{1}H NMR (400 MHz, $CDCl_3$) δ 7.05 – 6.82 (m, 4H), 4.32 (m, 2H), 4.28 – 4.11 (m, 2H), 3.68 – 3.59 (m, 1H), 3.25 (d, *J* = 10.1 Hz, 1H), 3.01 (d, *J* = 13.1 Hz, 1H), 2.96 (d, *J* = 9.8 Hz, 1H), 2.93 – 2.89 (m, 1H), 2.86 (d, *J* = 13.2 Hz, 1H), 1.32 (t, *J* = 7.2 Hz, 3H), 1.29 – 1.18 (m, 6H).

^{13}C{^{1}H} NMR (101 MHz, $CDCl_3$) δ 213.7, 212.9, 168.8, 166.3, 162.2 (d, *J* = 247.6 Hz, 1C), 131.4 (d, *J* = 8.0 Hz, 1C), 131.3 (d, *J* = 3.3 Hz, 1C), 123.7 (q, *J* = 280.4 Hz, 1C), 115.7 (d, *J* = 21.3 Hz, 1C), 75.0, 63.2, 62.6, 61.6 (q, *J* = 32.5 Hz, 1C), 60.7, 60.7, 56.3, 49.8, 42.7, 19.8, 14.0.
HRMS (ESI) *m/z*: $[M+H]^+$ calcd for $C_{22}H_{24}F_4NO_6$, 474.1534, found: 474.1534.

Diethyl 5-(2-chlorobenzyl)-5-methyl-4,6-dioxo-3-(trifluoromethyl)hexahydrocyclopenta[c] pyrrole-1,1(2H)-dicarboxylate(5f)

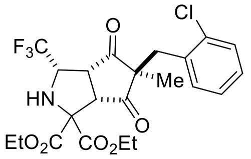

The product was purified by flash column chromatography (petroleum ether / ethyl acetate =10:1 as the eluent);
White solid; 64.6 mg, 44% yield; > 20:1 *dr*, mp 159.1-159.8°C;
^{1}H NMR (400 MHz, $CDCl_3$) δ 7.38 – 7.31 (m, 1H), 7.26 – 7.23 (m, 2H), 7.08 (dd, *J* = 6.8, 2.5 Hz, 1H), 4.42 – 4.28 (m, 2H), 4.27 – 4.10 (m, 2H), 3.77 (d, *J* = 10.2 Hz, 2H), 3.37 (t, *J* = 10.1 Hz, 1H), 3.10 (d, *J* = 2.9 Hz, 2H), 2.90 (d, *J* = 4.2 Hz, 1H), 1.31 (t, *J* = 7.2 Hz, 3H), 1.25 (t, *J* = 7.1 Hz, 3H), 1.20 (s, 3H).
^{113}C{^{1}H}NMR (101 MHz, $CDCl_3$) δ 211.0 (2C), 168.7, 166.3, 134.6, 132.7, 132.1, 130.0, 129.2, 127.0, 123.6 (q, *J* = 280.5Hz, 1C),74.0, 63.0, 62.3, 61.0 (q, *J* = 32.4 Hz, 1C), 59.3, 55.6, 49.9, 40.3, 17.8, 14.0, 13.9.
HRMS (ESI) *m/z*: $[M+H]^+$ calcd for $C_{22}H_{24}{}^{35}ClF_3NO_6$, 490.1239, found: 490.1242; calcd for $C_{22}H_{23}{}^{37}ClF_3NO_6$, 492.1225, found: 492.1225.

Diethyl 5-(4-bromobenzyl)-5-methyl-4,6-dioxo-3-(trifluoromethyl)hexahydrocyclopenta[c] pyrrole-1,1(2H)-dicarboxylate(5g)

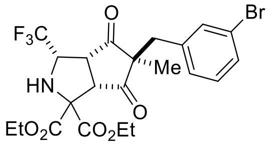

The product was purified by flash column chromatography (petroleum ether / ethyl acetate =10:1 as the eluent);
White solid; 84.8 mg, 53% yield; > 20:1 *dr*, mp 149.1-150.0 °C;
^{1}H NMR (400 MHz, $CDCl_3$) δ 7.36 (dd, *J* = 8.0, 1.9 Hz, 1H), 7.12 (dd, *J* = 14.4, 6.6 Hz, 2H), 6.92 (d, *J* = 7.6 Hz, 1H), 4.32 (m, 2H), 4.29 – 4.13 (m, 2H), 3.66 (m, 1H), 3.29 (d, *J* = 10.1 Hz, 1H), 3.08 – 2.96 (m, 2H), 2.92 (d, *J* = 3.9 Hz, 1H), 2.83 (d, *J* = 13.1 Hz, 1H), 1.33 (t, *J* = 7.2 Hz, 3H), 1.29 – 1.20 (m, 6H).
^{13}C{^{1}H} NMR (101 MHz, $CDCl_3$) δ 213.1, 212.3 168.7, 166.1, 137.8 132.7, 130.7, 130.3, 128.4, 123.5 (q, *J* = 280.3 Hz, 1C),122.7, 74.9, 63.1, 62.5, 61.5 (q, *J* = 32.4 Hz, 1C), 60.4, 56.1, 49.7, 42.7, 19.8, 14.0, 13.9.
HRMS (ESI) *m/z*: $[M+H]^+$ calcd for $C_{22}H_{24}{}^{79}BrF_3NO_6$, 534.0734, found: 534.0732; calcd for $C_{22}H_{23}{}^{81}BrF_3NO_{6,}$, 536.0716, found: 536.0717.

Diethyl 5-methyl-5-(naphthalen-1-ylmethyl)-4,6-dioxo-3-(trifluoromethyl) hexahy drocyclopenta[c]pyrrole-1,1(2H)-dicarboxylate(5h)

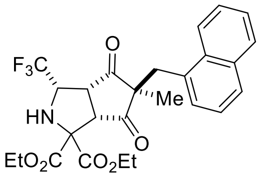

The product was purified by flash column chromatography (petroleum ether / ethyl acetate =10:1 as the eluent);

White solid; 89.4 mg, 59% yield; > 20:1 *dr*, mp 158.3-159.1 °C;

^{1}H NMR (400 MHz, $CDCl_3$) δ 7.98 – 7.91 (m, 1H), 7.87 – 7.80 (m, 1H), 7.78 (d, *J* = 8.2 Hz, 1H), 7.49 (m, 2H), 7.37 (m, 1H), 7.18 (d, *J* = 7.0 Hz, 1H), 4.37 – 4.15 (m, 2H), 4.15 – 3.97 (m, 2H), 3.56 (m, 6.8 Hz, 1H), 3.48 (s, 2H), 2.88 – 2.72 (m, 2H), 2.64 (d, *J* = 10.4 Hz, 1H), 1.34 (s, 3H), 1.23 (t, *J* = 7.2 Hz, 3H), 1.13 (t, *J* = 7.1 Hz, 3H).

^{13}C{^{1}H} NMR (101 MHz, $CDCl_3$) δ 213.2, 212.8, 168.5, 166.2, 133.9, 131.7, 131.6, 128.9, 128.7, 128.4, 126.5, 126.1, 125.3, 124.1, 123.7 (q, *J* = 280.4 Hz, 1C), 74.0, 62.9, 62.2, 61.0 (q, *J* = 32.4 Hz, 1C), 60.6, 56.5, 50.2, 41.0, 19.5, 13.9 (2C).

HRMS (ESI) *m/z*: $[M+H]^+$ calcd for $C_{26}H_{27}F_3NO_6$, 506.1785, found: 506.1789.

Diethyl 5-methyl-5-(naphthalen-2-ylmethyl)-4,6-dioxo-3-(trifluoromethyl) hexahy drocyclopenta[c]pyrrole-1,1(2H)-dicarboxylate(5i)

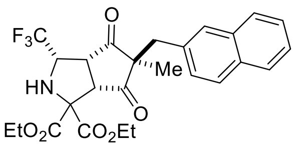

The product was purified by flash column chromatography (petroleum ether / ethyl acetate =10:1 as the eluent);

White solid; 75.8 mg, 50% yield; > 20:1 *dr*, mp 122.5-123.4 °C;

^{1}H NMR (400 MHz, $CDCl_3$) δ 7.82 – 7.74 (m, 2H), 7.72 (d, *J* = 8.5 Hz, 1H), 7.51 – 7.41 (m, 3H), 7.11 (dd, *J* = 8.4, 1.7 Hz, 1H), 4.33 (m, *J* = 7.1 Hz, 2H), 4.22 – 4.05 (m, 2H), 3.59 (m, 3.8 Hz, 1H), 3.21 (t, *J* = 11.8 Hz, 2H), 3.06 (d, *J* = 13.0 Hz, 1H), 2.95 – 2.83 (m, 2H), 1.39 – 1.27 (m, 6H), 1.15 (t, *J* = 7.1 Hz, 3H).

^{13}C{^{1}H} NMR (101 MHz, $CDCl_3$) δ 213.8, 212.9, 168.8, 166.3, 133.4, 133.0, 132.5, 128.7, 128.5, 127.9, 127.7, 127.7, 126.5, 126.2, 123.7 (q, *J* = 280.4 Hz, 1C), 74.9, 63.1, 62.5, 61.6 (q, *J* = 32.6 Hz, 1C), 60.9, 56.3, 49.8, 44.0, 19.9, 14.0, 13.9.

HRMS (ESI) *m/z*: $[M+H]^+$ calcd for $C_{26}H_{27}F_3NO_6$, 506.1785, found: 506.1790.

7. Scale-up experiment.

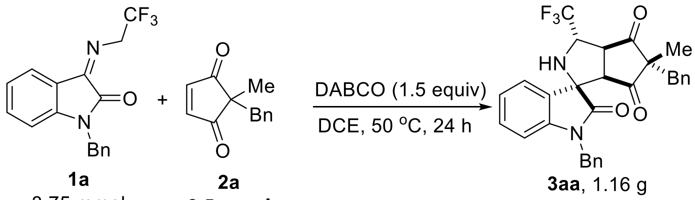

In a dry 100 mL round-bottom flask equipped with a magnetic stirring bar, *N*-2,2,2-trifluoroethylisatin ketimines **1a** (1.2 g, 3.75 mmol, 1.5 equiv), DABCO (0.4 g, 3.75 mmol,1.5 equiv), and **2a** (0.5 g, 2.5 mmol, 1.0 equiv), DCE (25.0 mL) were added in sequence. Then the mixture was stirred for 24 h at 50 °C. After completion, the reaction mixture was directly purified by flash chromatography on silica gel (petroleum ether / ethyl acetate = 15:1) to give the corresponding product **3aa**.

8. Different transformations of products.

8.1. Procedure for the synthesis of 6.

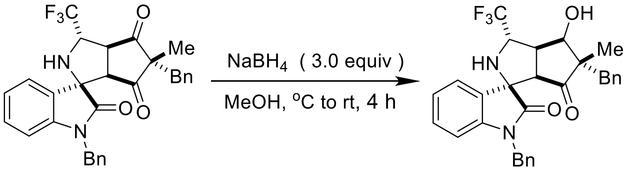

3aa **6**

Compound **3aa** (0.1 g, 0.2 mmol) was dissolved in MeOH (2.0 mL), and then $NaBH_4$ (22.7 mg, 3.0 equiv) was added slowly at 0 ℃. The reaction mixture was then allowed to stir at room temperature for 4 h. The reaction system was monitored by TLC until **3aa** disappeared completely. After that, the reaction mixture was quenched by a drop of water and directly purified by column chromatography on silica gel (petroleum ether / ethyl acetate = 15:1) to provide compound **6** as a white solid (89.5 mg, 86% yield).

White solid; 89.5 mg, 86% yield; 83:17 *dr*, mp 188.2-188.9 °C;

^{1}H NMR (300 MHz, DMSO-d_6) δ 7.44 – 7.27 (m, 5H), 7.28 – 7.18 (m, 5H), 7.13 (dd, *J* = 7.5, 1.1 Hz, 1H), 7.11 – 7.01 (m, 2H), 6.79 – 6.70 (m, 1H), 5.58 (s, 1H), 4.92 (d, *J* = 15.9 Hz, 1H), 4.70 (d, *J* = 16.0 Hz, 1H), 4.63 – 4.50 (m, 1H), 3.95 (d, *J* = 11.6 Hz, 2H), 3.68 (ddd, *J* = 11.1, 6.3, 3.8 Hz, 2H), 2.86 – 2.69 (m, 2H), 0.90 (s, 3H).

^{13}C NMR{^{1}H} (75 MHz, $CDCl_3$) δ 218.1, 180.1, 141.7, 136.2, 135.2, 132.5, 130.3, 129.1, 128.5, 127.9, 127.4, 127.3, 126.7 (q, *J* = 277.5 Hz, 1C),126.6, 123.88, 123.6, 110.0, 75.5, 70.8, 59.4, 59.3, 58.7 (q, *J* = 30.0 Hz, 1C), 44.2, 43.2, 41.2, 14.8**.**

HRMS (ESI) *m/z* $[M+H]^+$ calcd for $C_{30}H_{28}F_3N_2O_3$, 521.2047, found: 521.2046.

8.2 Procedure for the synthesis of 7.

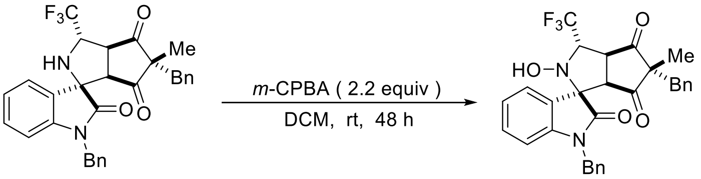

3aa **7**

The solution of compound **3aa** (0.1 g, 0.20 mmol) in DCM (2.0 mL) was stirred at room temperature in a sealed tube. Subsequently, *m*-CPBA (76.1 mg, 0.44mmol) was added to the above solution. The reaction mixture was then allowed to stir 48 h. The reaction mixture was quenched by the addition of $NaHCO_3$ aq (15 mL), and diluted with EtOAc (15 mL). The organic layer was separated, and the aqueous layer was extracted twice with EtOAc (2 × 15 mL). The combined organic layers were dried over Na_2SO_4, Subsequently, the organic phase was concentrated under reduced pressure. The crude product was purified by flash column

chromatography on silica gel (petroleum ether / ethyl acetate = 15:1) to afford the desired product **7** as a white solid (44.9 mg, 42% yield).

White solid; 44.9 mg, 42% yield; 86:14 *dr*, mp 135.8-136.6 °C;

^{1}H NMR (300 MHz, $CDCl_3$) δ 7.37 – 7.24 (m, 7H), 7.24 – 7.14 (m, 3H), 7.07 (m, *J* = 7.6, 1.0 Hz, 1H), 7.04 – 6.93 (m, 2H), 6.60 (d, *J* = 7.8 Hz, 1H), 5.07 (s, 1H), 4.76 (s, 2H), 4.60 (m, *J* = 6.2 Hz, 1H), 3.00 (d, *J* = 11.9 Hz, 1H), 2.93 (d, *J* = 12.9 Hz, 1H), 2.84 (d, *J* = 12.9 Hz, 1H), 2.46 (dd, *J* = 11.9, 6.0 Hz, 1H), 1.27 (s, 3H).

^{13}C NMR{^{1}H} (75 MHz, $CDCl_3$) δ 214.2, 211.2, 174.7, 144.3, 134.9, 134.6, 130.8, 129.5, 129.1, 128.9, 128.2, 127.8, 127.2, 126.6, 125.8, 124.9 (q, *J* = 277.2 Hz, 1C), 124.2, 123.6, 110.1, 65.6 (q, *J* = 30.5 Hz, 1C), 62.3, 54.3, 48.2, 46.4, 43.8, 18.2.

HRMS (ESI) *m/z* $[M+H]^+$ calcd for $C_{30}H_{26}F_3N_2O_4$, 535.1839, found: 535.1845.

8.3 Procedure for the synthesis of 8.

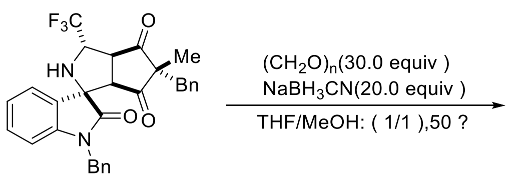

3aa **8**

Compound **3aa** (0.10 g, 0.20 mmol) and polyformaldehyde (0.5 g, 30.0 equiv) was dissolved in the mixture solvents of MeOH /THF (1:1, 4.0 mL). Then the $NaBH_3CN$ (0.3 g, 20.0 equiv) was added into the mixture at room temperature for 30 minutes. The reaction mixture was then allowed to stir 72 h at 50 ℃. After completion, the reaction mixture was quenched by addition of 1.0 M NaOH (10.0 mL). The aqueous layer was extracted with DCM (2×10.0 mL). The combined organic layers were dried over anhydrous Na_2SO_4. After filtration, the solution was concentrated under reduced pressure and the resulting crude mixture was purified by hexane beating twice to provide compound **8** as a white solid (100.1 mg, 89% yield).

White solid; 100.1 mg, 89% yield; 86:14 *dr*, mp 192.3-193.1 °C;

^{1}H NMR (400 MHz, $CDCl_3$) δ 7.31 (m, 5H), 7.27 (d, *J* = 6.5 Hz, 3H), 7.22 (d, *J* = 7.8 Hz, 2H), 7.08 (m, 1H), 7.04 – 6.96 (m, 2H), 6.65 (d, *J* = 7.8 Hz, 1H), 4.72 (s, 3H), 4.06 (d, *J* = 11.0 Hz, 1H), 3.73 (d, *J* = 11.0 Hz, 1H), 3.13 (d, *J* = 10.2 Hz, 1H), 2.99 (s, 3H), 2.96 (d, *J* = 12.7 Hz, 1H), 2.84 (d, *J* = 12.7 Hz, 1H), 2.60 (dd, *J* = 12.0, 4.0 Hz, 1H), 1.28 (s, 3H).

^{13}C NMR{^{1}H} (101 MHz, $CDCl_3$) δ 214.5, 211.0, 177.5, 144.0, 135.3, 134.9, 130.6, 129.5, 129.0, 128.9, 128.1, 127.8, 127.4, 126.1, 125.7(q, *J* = 304.0 Hz, 1C),124.4, 123.6, 110.0, 79.2, 73.8, 63.3, 60.6(q, *J* = 30.0 Hz, 1C), 58.5, 56.4, 52.1, 46.4, 43.9, 17.7.

HRMS (ESI) *m/z* $[M+H]^+$ calcd for $C_{32}H_{30}F_3N_2O_4$, 563.2152, found: 563.2163.

8.4 Procedure for the synthesis of 9.

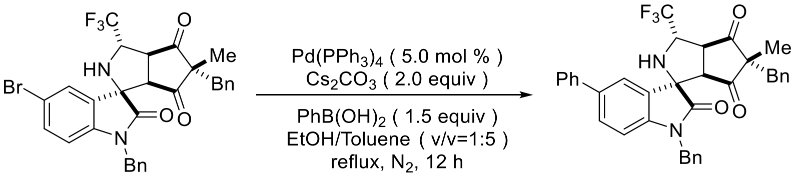

3ka **9**

To an oven-dried schlenk tube, **3ak** (119.2 mg, 0.20 mmol), $PhB(OH)_2$ (36.6 mg, 0.30mmol), Cs_2CO_3 (130.4 mg, 0.40 mmol) were added and then the mixture solvents of ethanol (0.4 mL) and toluene (2.0 mL) was added into reaction tube. The reaction mixture was stirred at 120 ℃ under N_2 atmosphere for 12 hours. The heat source is an oil bath. When the mixture was cooled to room temperature, the brine (10 mL) was added to quench the reaction. The aqueous layer was extracted with DCM (2×10.0 mL), and the combined organic layers were dried over anhydrous Na_2SO_4. After filtration, the organic phase was concentrated and purified by column chromatography on silica gel (petroleum ether / ethyl acetate = 15:1) to afford product **9** as a white solid (108.1 mg, 91% yield).

White solid; 108.1 mg, 91% yield; 86:14 dr, mp.140.0-140.7 °C;

^{1}H NMR (400 MHz, DMSO-*d*$_6$) δ 7.64 – 7.53 (m, 2H), 7.46 (m, 3H), 7.43 – 7.39 (m, 1H), 7.39 – 7.37 (m, 1H), 7.36 – 7.23 (m, 8H), 7.13 – 6.95 (m, 3H), 4.93 (d, *J* = 15.9 Hz, 1H), 4.73 (d, *J* = 15.9 Hz, 1H), 4.69 (d, *J* = 5.6 Hz, 1H), 4.52 – 4.38 (m, 1H), 4.06 (d, *J* = 3.9 Hz, 2H), 3.02 (d, *J* = 13.3 Hz, 1H), 2.87 (d, *J* = 13.3 Hz, 1H), 0.96 (s, 3H).

^{13}C NMR{^{1}H} (101 MHz, DMSO-*d*$_6$) δ 212.8, 210.8, 178.2, 143.9, 142.0, 139.9, 135.6, 134.5, 130.2, 129.0, 128.4, 128.1, 127.8, 127.7, 127.3, 127.3, 127.2, 126.7,125.6 (q, *J* = 276.4 Hz, 1C), 124.6, 121.7, 108.1, 70.8, 62.0, 58.7 (q, *J* = 31.0 Hz, 1C)57.8, 51.3, 42.7, 41.5, 14.3.

HRMS (ESI) *m/z* $[M+H]^+$ calcd for $C_{36}H_{30}F_3N_2O_3$, 595.2203 found: 595.2209

9. X-ray crystal structure of compounds 3aa, 8 and 5a.

Single crystal of compound **3aa** was prepared from the mixture solvent of MeOH/ Dichloromethane (V : V = 6/1) at room temperature by slow evaporation of solvent. A suitable crystal was selected for structure determination on a Xcalibur, Eos, Gemini diffractometer. The crystal was kept at 293(2) K during data collection. Using Olex2[1], the structure was solved with the ShelXS[2] structure solution program using Direct Methods and refined with the ShelXL[3] refinement package using Least Squares minimisation.

[1] Dolomanov, O. V.; Bourhis, L. J.; Gildea, R. J, Howard, J. A. K.; Puschmann, H. *J. Appl. Cryst*. **2009**, *42*, 339-341.

[2]Sheldrick, G. M. *Acta Cryst.* **2008**, A64, 112-122.

[3]Sheldrick, G. M. *Acta Cryst*. **2015**, C71, 3-8.

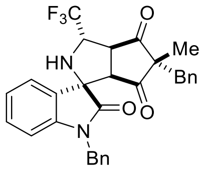

3aa

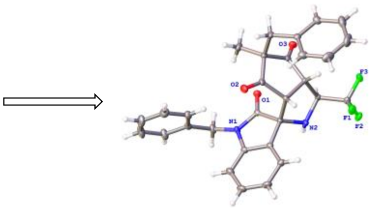

(CCDC- 2263528)

ORTEP of **3aa** (at 50% level)

Crystal data and structure refinement for 3aa.

Identification code	**3aa**
Empirical formula	$C_{30}H_{25}F_3N_2O_3$
Formula weight	518.52
Temperature/K	179.99(10)
Crystal system	triclinic
Space group	P-1

a/Å	8.6814(4)
b/Å	10.3082(6)
c/Å	14.7740(8)
α/°	88.935(5)
β/°	81.813(4)
γ/°	71.234(5)
Volume/Å3	1238.55(12)
Z	2
ρcalcg/cm3	1.390
μ/mm-1	0.105
F(000)	540.0
Crystal size/mm3	0.15 × 0.13 × 0.11
Radiation	Mo Kα (λ = 0.71073)
2Θ range for data collection/°	4.174 to 49.998
Index ranges	-8 ≤ h ≤ 10, -8 ≤ k ≤ 12, -17 ≤ l ≤ 17
Reflections collected	8048
Independent reflections	4362 [Rint = 0.0190, Rsigma = 0.0344]
Data/restraints/parameters	4362/0/348
Goodness-of-fit on F2	1.082
Final R indexes [I>=2σ (I)]	R1 = 0.0424, wR2 = 0.0968
Final R indexes [all data]	R1 = 0.0508, wR2 = 0.1027
Largest diff. peak/hole / e Å-3	0.18/-0.32

Single crystal of compound **5a** was prepared from the mixture solvent of Hexane/Ethyl acetate (*V* : *V* = 8/1) at room temperature by slow evaporation of solvent.

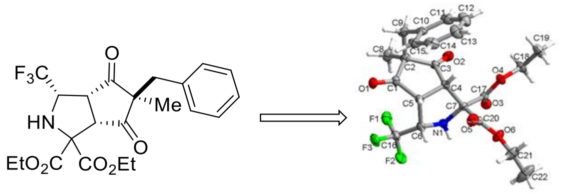

5a (CCDC-2263529)

ORTEP of **5a** (at 50% level)

Crystal data and structure refinement for 5a

Identification code	**5a**
Empirical formula	$C_{22}H_{24}F_3NO_6$
Formula weight	455.42
Temperature/K	293(2)
Crystal system	triclinic
Space group	P-1
a/Å	10.1328(11)
b/Å	11.3561(14)
c/Å	11.5752(17)
α/°	67.509(13)
β/°	64.451(13)

γ/°	81.505(10)
Volume/Å3	1110.1(3)
Z	2
ρcalcg/cm3	1.363
μ/mm-1	0.988
F(000)	476.0
Crystal size/mm3	0.15 × 0.11 × 0.1
Radiation	CuKα (λ = 1.54184)
2Θ range for data collection/°	8.428 to 134.114
Index ranges	-9 ≤ h ≤ 12, -13 ≤ k ≤ 12, -13 ≤ l ≤ 13
Reflections collected	7940
Independent reflections	3935 [Rint = 0.0248, Rsigma = 0.0330]
Data/restraints/parameters	3935/0/296
Goodness-of-fit on F2	1.049
Final R indexes [I>=2σ (I)]	R1 = 0.0502, wR2 = 0.1356
Final R indexes [all data]	R1 = 0.0612, wR2 = 0.1496
Largest diff. peak/hole / e Å-3	0.27/-0.23

Single crystal of compound **8** was prepared from the mixture solvent of MeOH/Ethyl acetate (V : V = 5/1) at room temperature by slow evaporation of solvent.

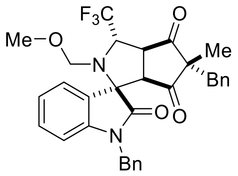

8

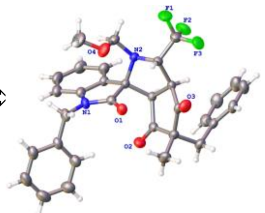

(CCDC-2263530)

ORTEP of **8** (at 50% level)

Crystal data and structure refinement for 8.

Identification code	**8**
Empirical formula	$C_{32}H_{29}F_3N_2O4$
Formula weight	562.57
Temperature/K	296.15
Crystal system	triclinic
Space group	P-1
a/Å	10.034(10)
b/Å	12.228(12)
c/Å	12.997(13)
α/°	69.56(2)
β/°	86.77(2)
γ/°	87.29(2)
Volume/Å3	1491(3)
Z	2
ρcalcg/cm3	1.253

μ/mm-1	0.095
F(000)	588.0
Crystal size/mm3	0.15 × 0.12 × 0.11
Radiation	MoKα (λ = 0.71073)
2Θ range for data collection/°	5.158 to 50.416
Index ranges	-11 ≤ h ≤ 11, -14 ≤ k ≤ 14, -15 ≤ l ≤ 15
Reflections collected	40902
Independent reflections	5302 [Rint = 0.0772, Rsigma = 0.0602]
Data/restraints/parameters	5302/0/376
Goodness-of-fit on F2	1.037
Final R indexes [I>=2σ (I)]	R1 = 0.0622, wR2 = 0.1240
Final R indexes [all data]	R1 = 0.1239, wR2 = 0.1531
Largest diff. peak/hole / e Å-3	0.18/-0.23

References:

[1] (a) Ma, M.; Zhu, Y.; Sun, Q.; Li, X.; Su, J; Zhao, L.; Zhao, Y.; Qiu, S.; Yan, W.; Wang, K.; Wang, R. The asymmetric synthesis of CF3-containing spiro[pyrrolidin-3,2′-oxindole] through the organocatalytic 1,3-dipolar cycloaddition reaction. *Chem. Commun.*, **2015**, 51, 8789. (b) Ryu, H.; Seo, J.; Ko H. M. Synthesis of Spiro[oxindole-3,2′-pyrrolidine] Derivatives from Benzynes and Azomethine Ylides through 1,3-Dipolar Cycloaddition Reactions. *J. Org. Chem.* **2018**, *83*, 14102.

[2] Das, T.; Saha, P.; Singh, V. K. Silver(I)-Ferrophox Catalyzed Enantioselective Desymmetrization of Cyclopentenedione: Synthesis of Highly Substituted Bicyclic Pyrrolidines *Org. Lett.*, **2015**, 17, 5088.

[3] Zhi Y., Zhao, K., Liu, Q.; Wang, A.; Enders, D. Asymmetric synthesis of functionalized trifluoromethyl-substituted pyrrolidines: Via an organocatalytic domino Michael/Mannich [3+2] cycloaddition. *Chem. Commun.* **2016**, *52*, 14011.

10. ^{1}H NMR, ^{13}C{^{1}H } NMR spectra for compounds 3, 5, 6, 7, 8, 9.

^{1}H NMR (400 MHz, DMSO-d_6) of **3aa**

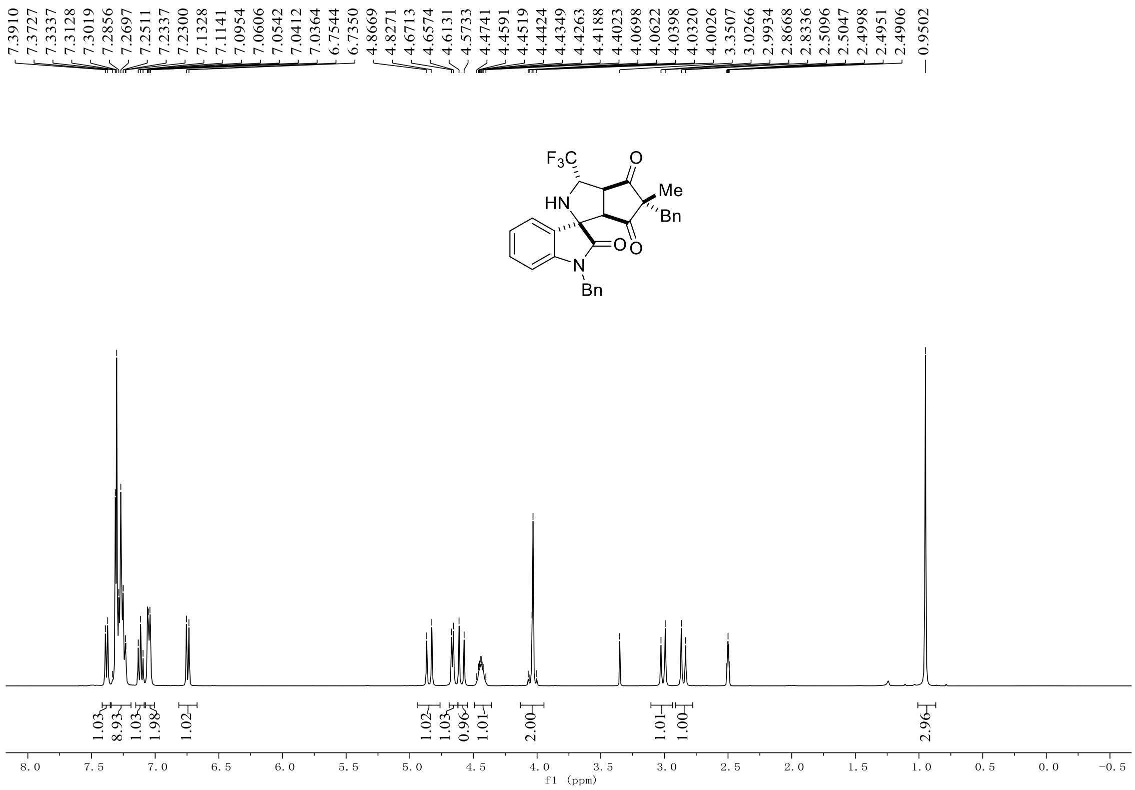

^{13}C{^{1}H} NMR (101 MHz, DMSO-d_6) of **3aa**

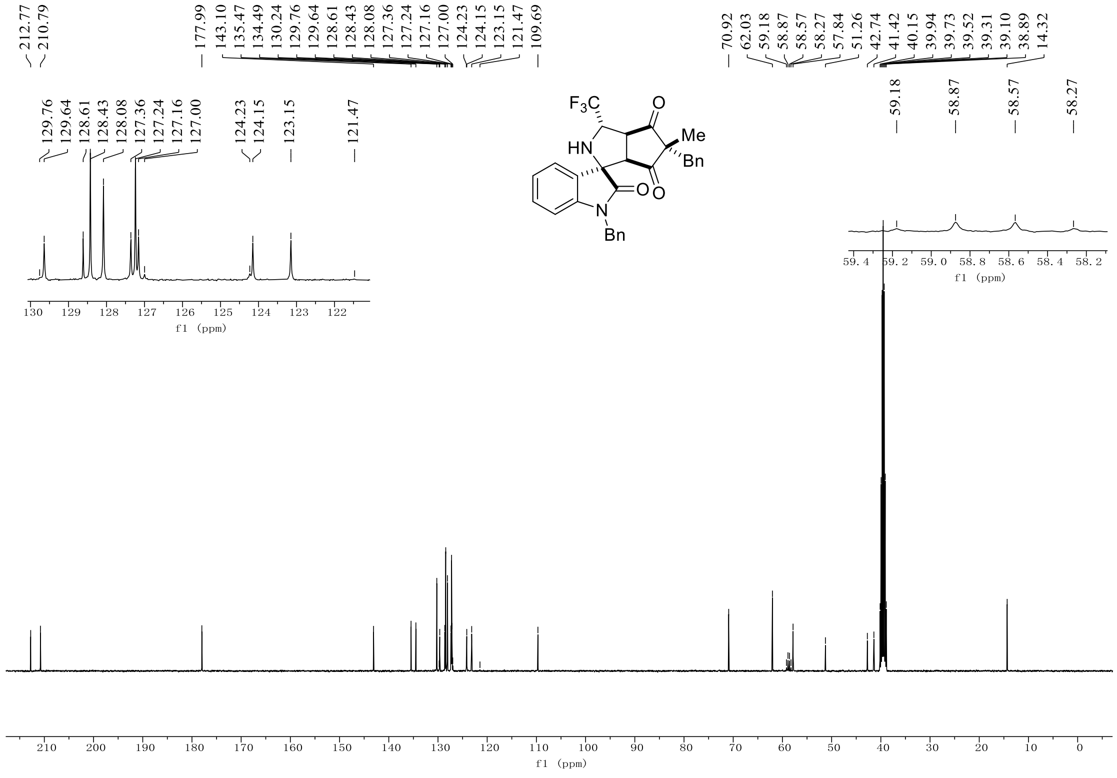

^{1}H NMR (400 MHz, DMSO-d_6) of **3ba**

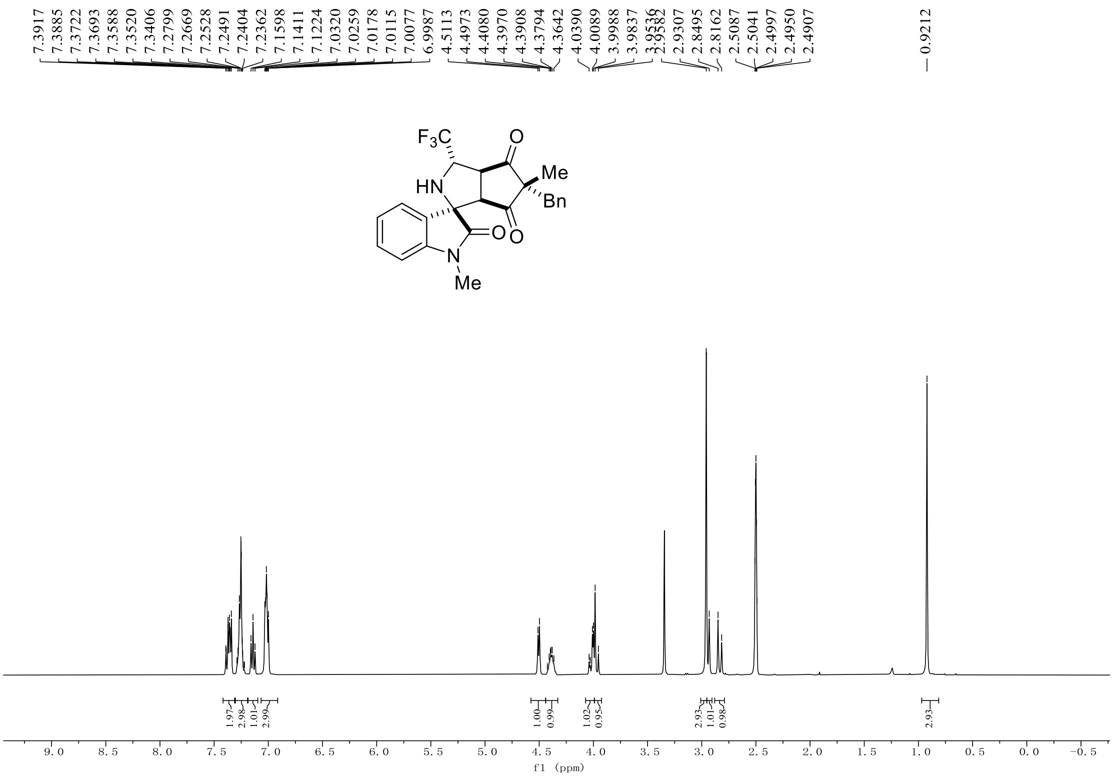

$^{13}C\{^{1}H\}$ NMR (101 MHz, DMSO-d_6) of **3ba**

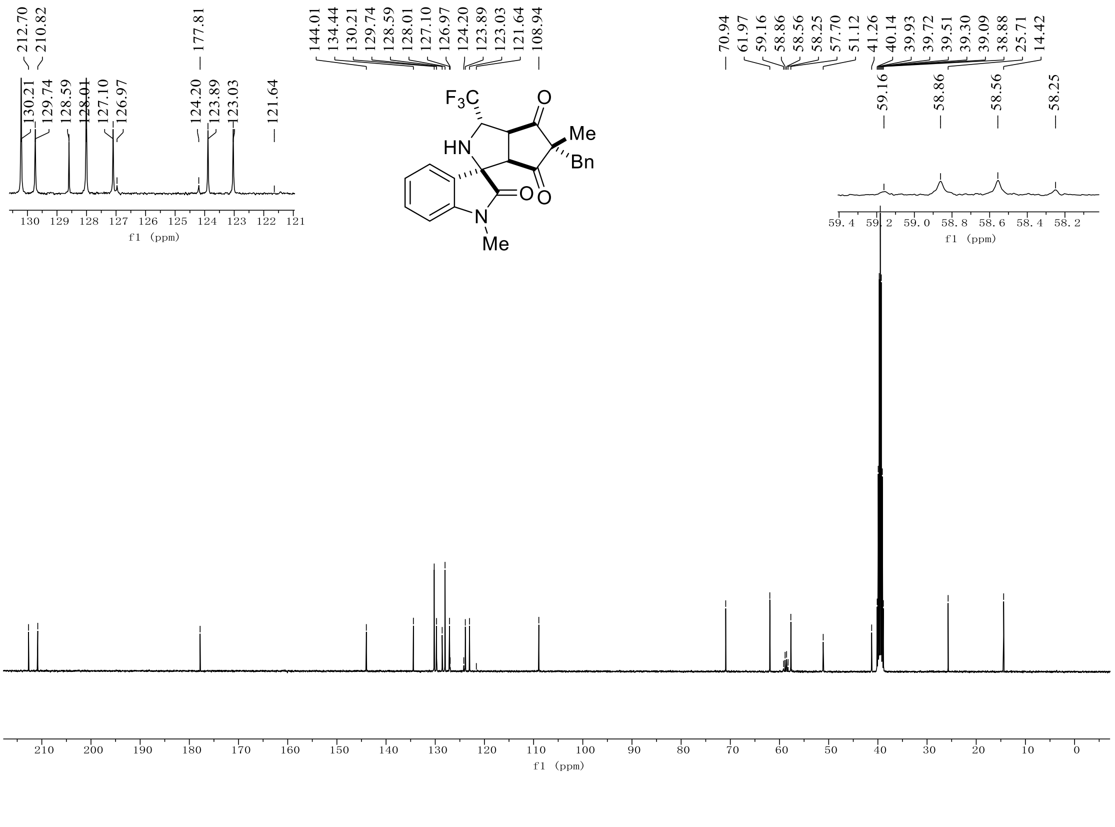

^{1}H NMR (400 MHz, $CDCl_3$) of **3ca**

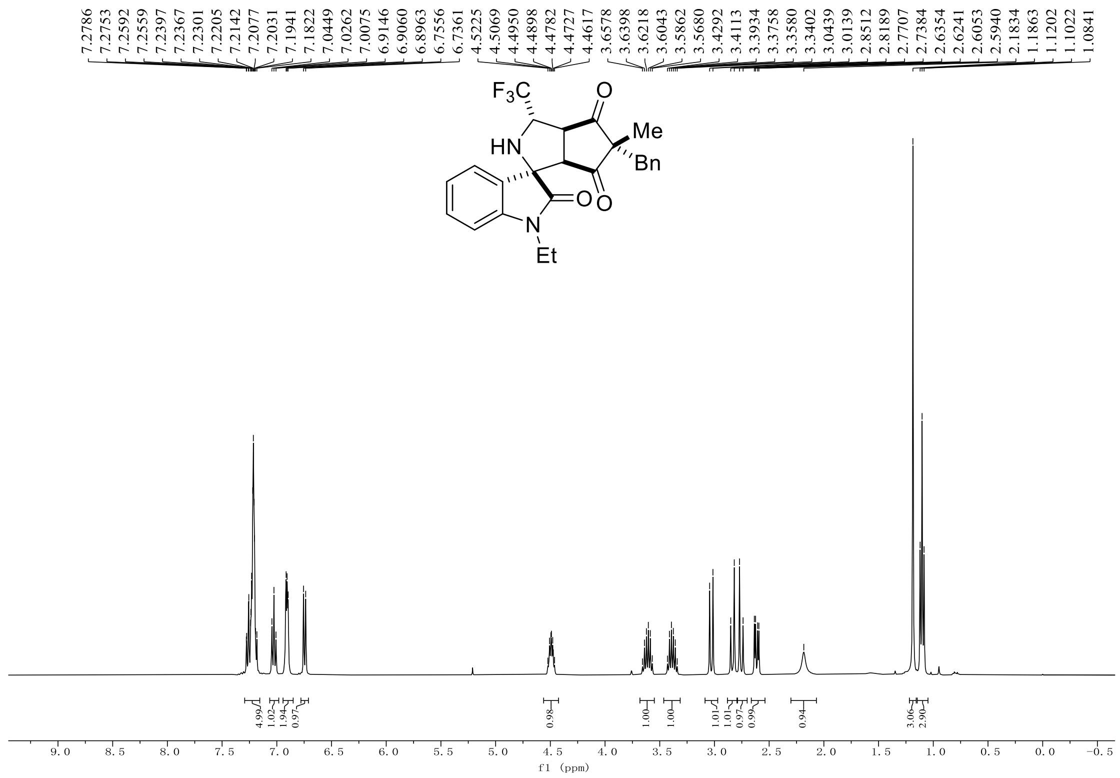

^{13}C{^{1}H} NMR (101 MHz, DMSO-d_6) of **3ca**

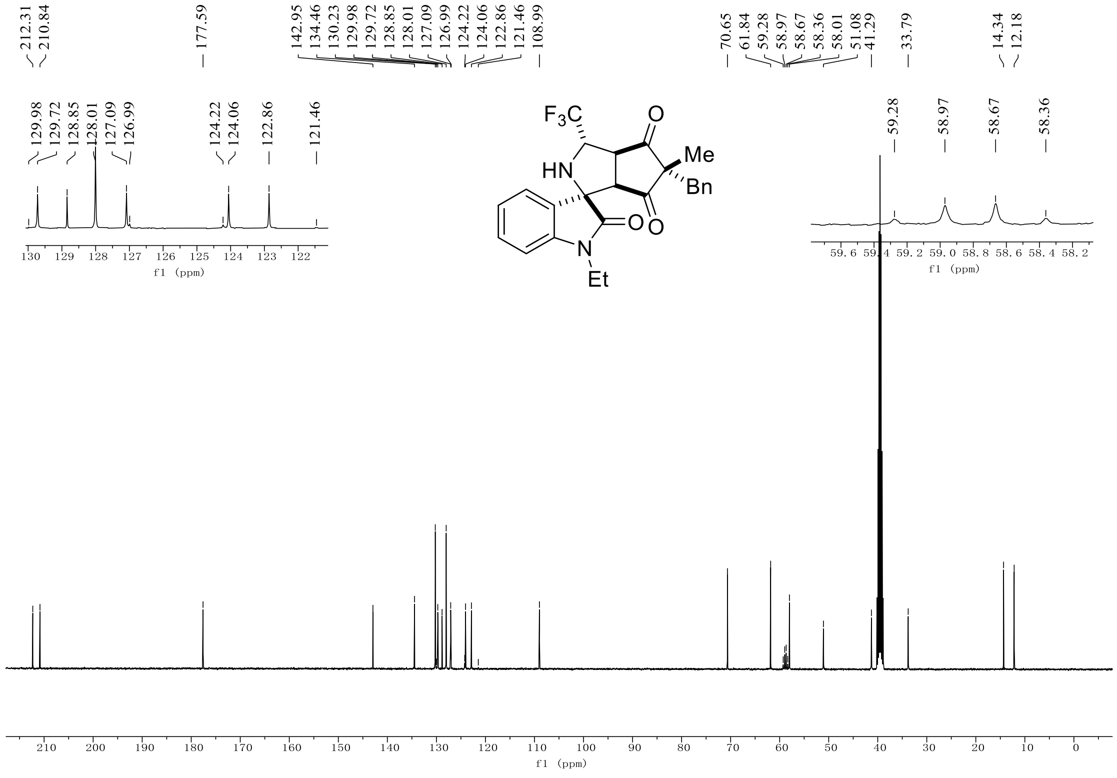

^{1}H NMR (400 MHz, DMSO-d_6) of 3**da**

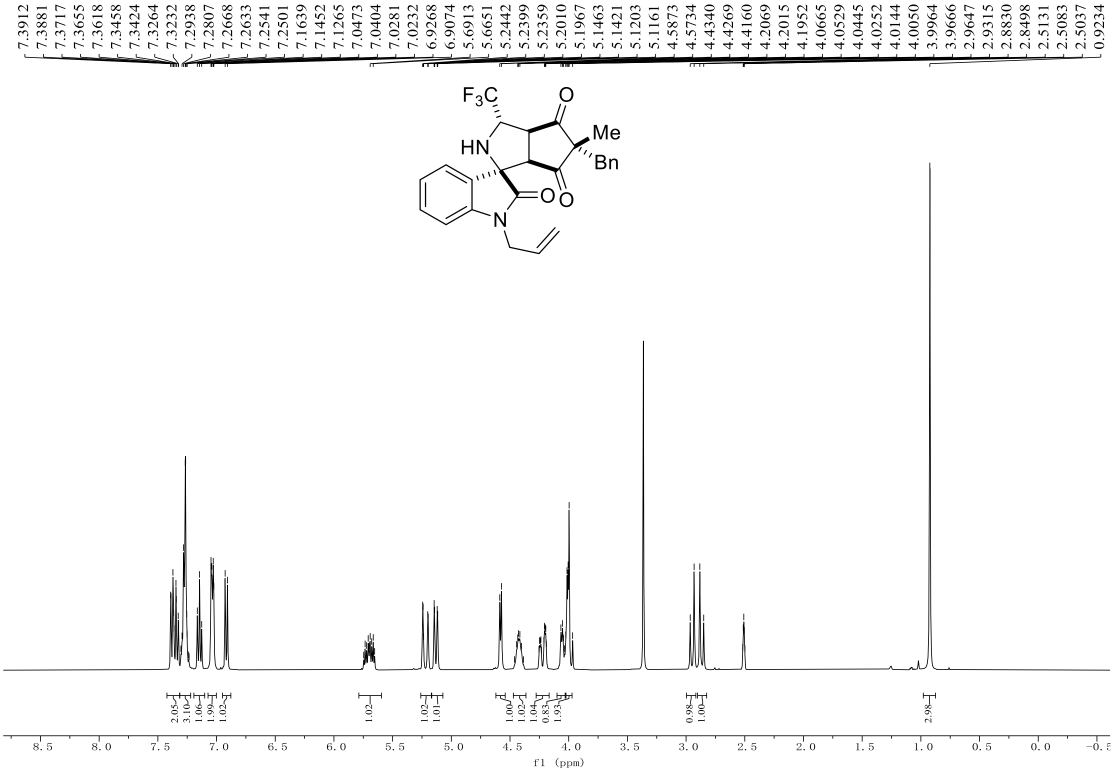

$^{13}C\{^{1}H\}$ NMR (101 MHz, DMSO-d_6) of **3da**

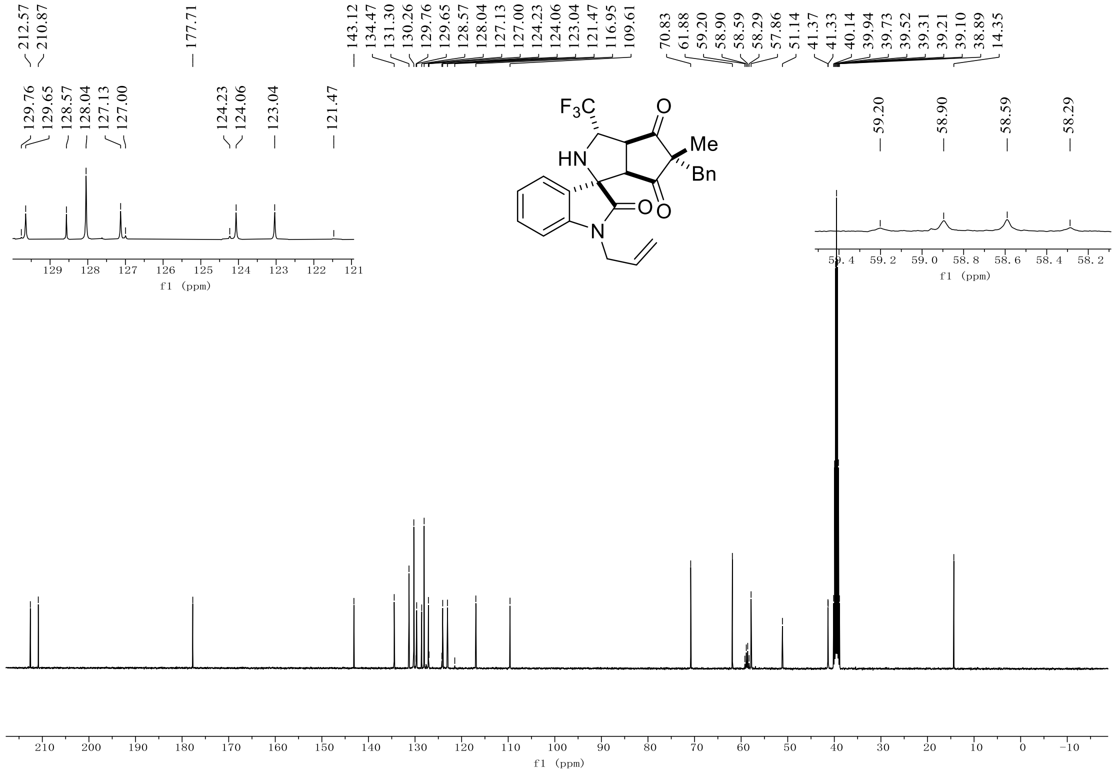

^{1}H NMR (400 MHz, DMSO-d_6) of **3ea**

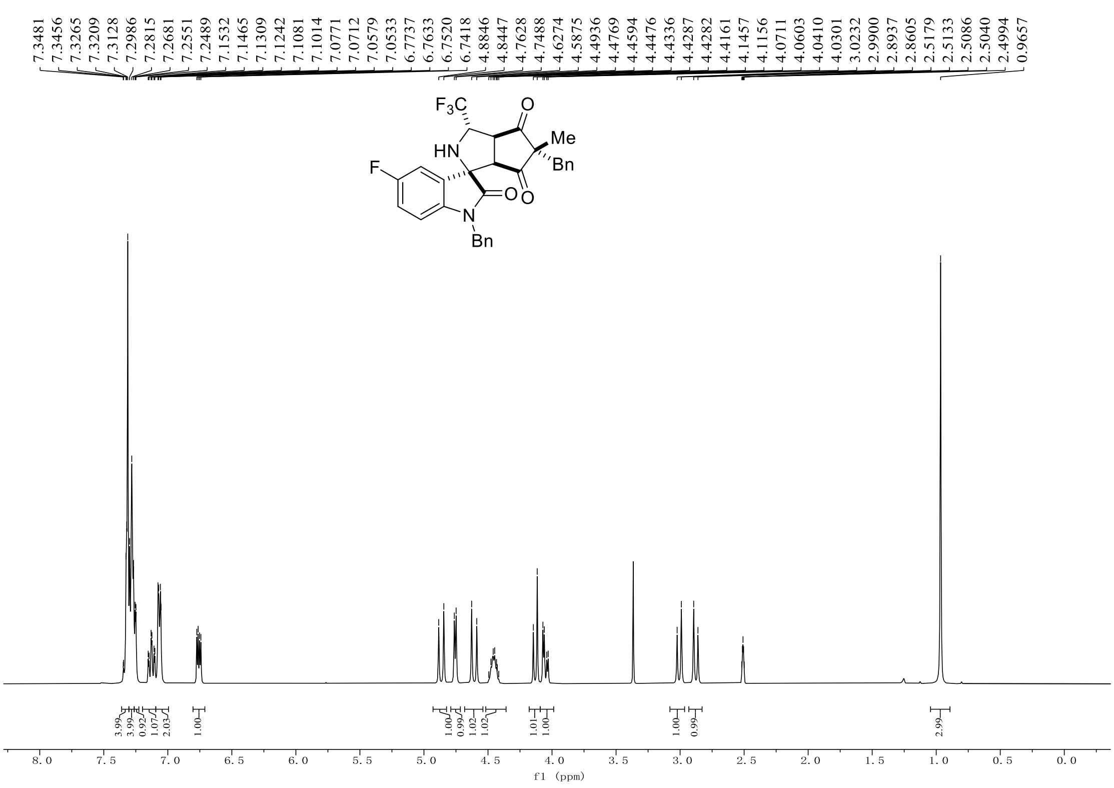

^{13}C{^{1}H} NMR (101 MHz, DMSO-d_6) of **3ea**

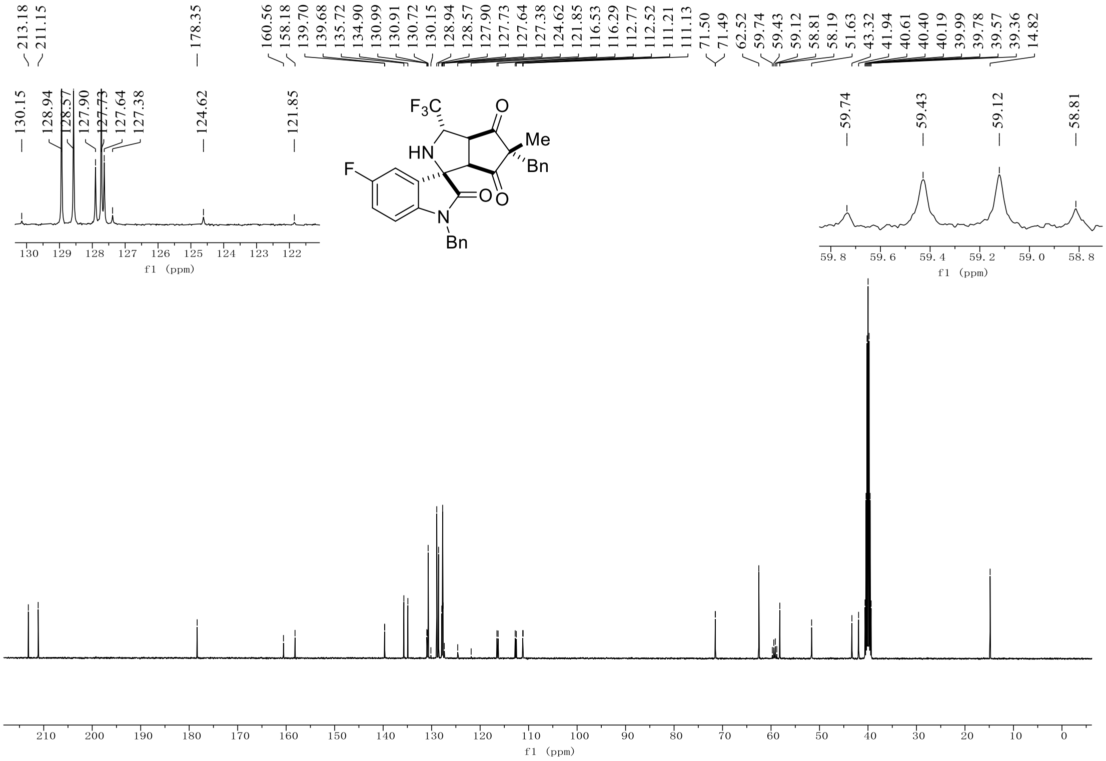

^{1}H NMR (400 MHz, DMSO-d_6) of **3fa**

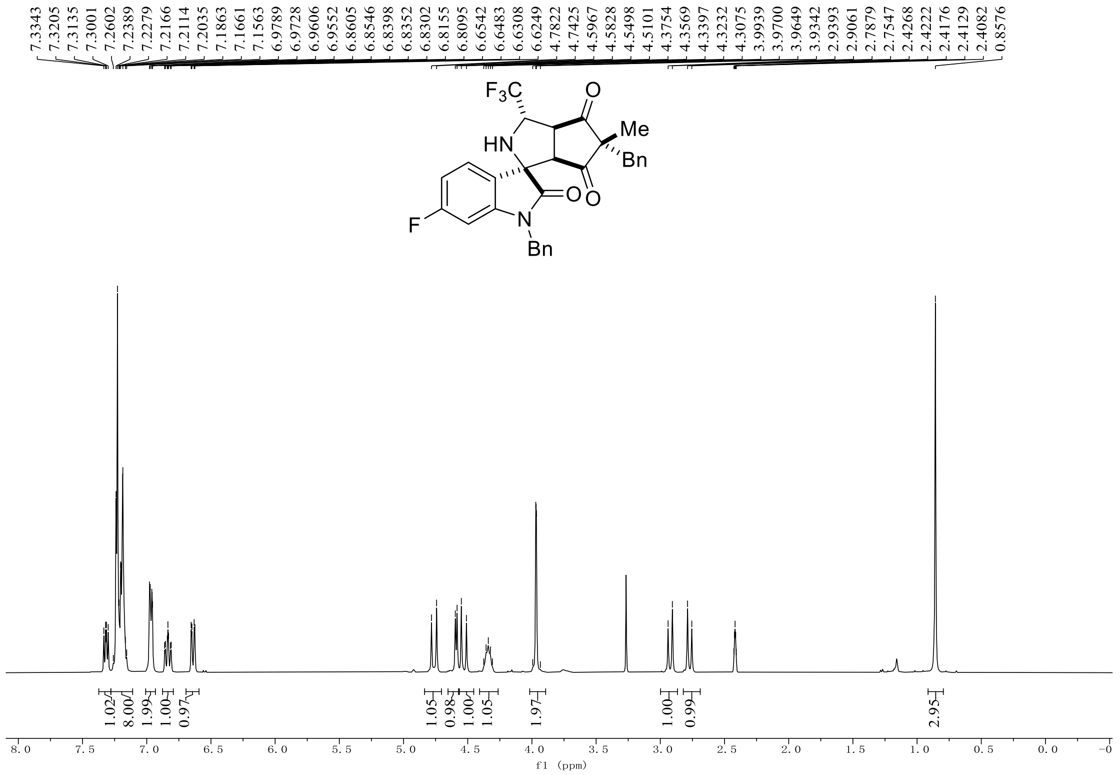

^{13}C{^{1}H} NMR (101 MHz, DMSO-d_6) of **3fa**

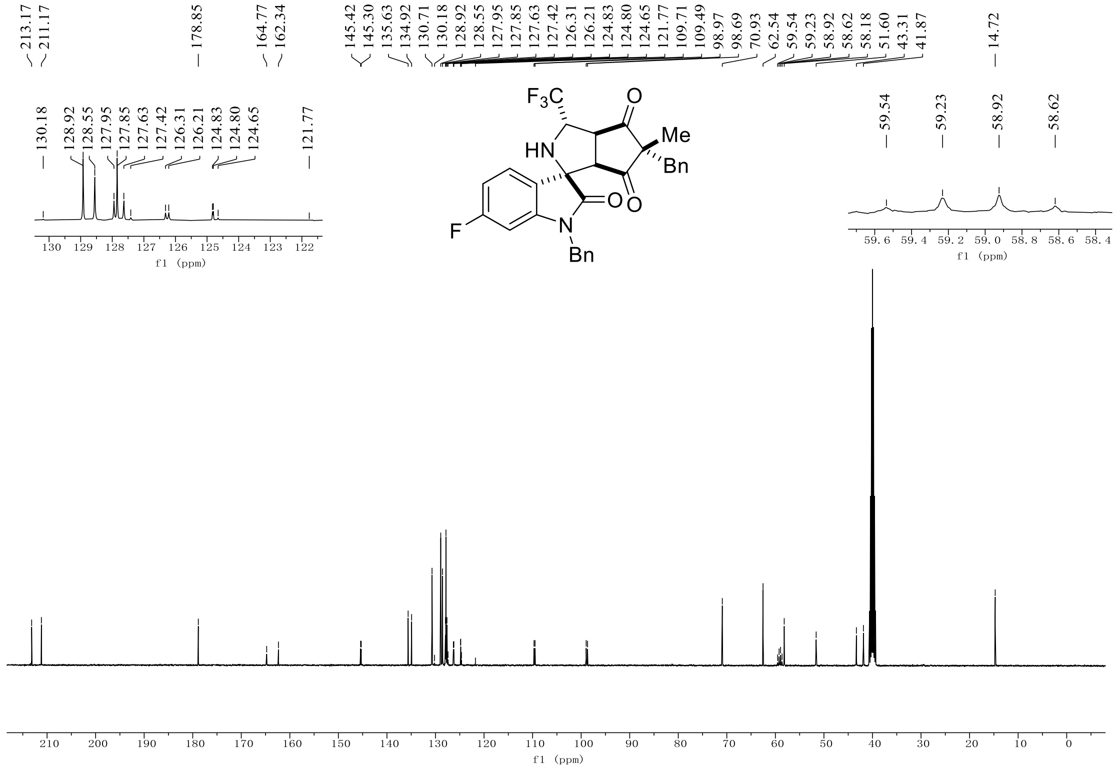

^{1}H NMR (400 MHz, DMSO-d_6) of **3ga**

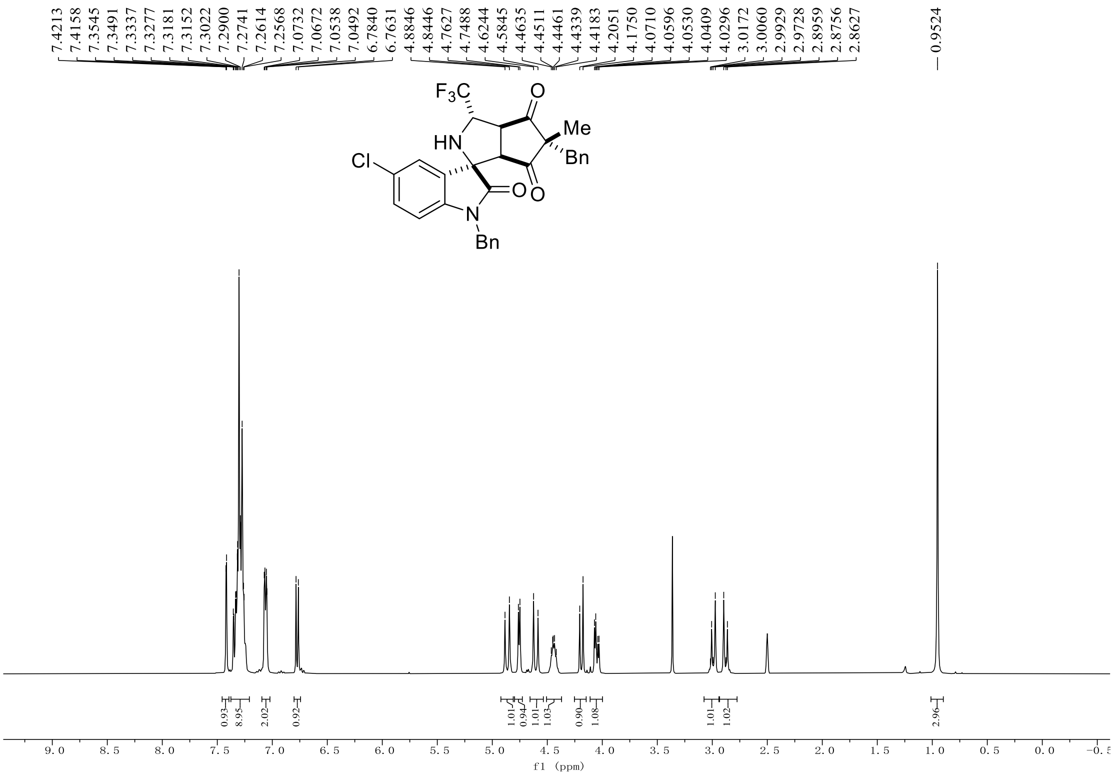

^{13}C{^{1}H} NMR (101 MHz, DMSO-d_6) of **3ga**

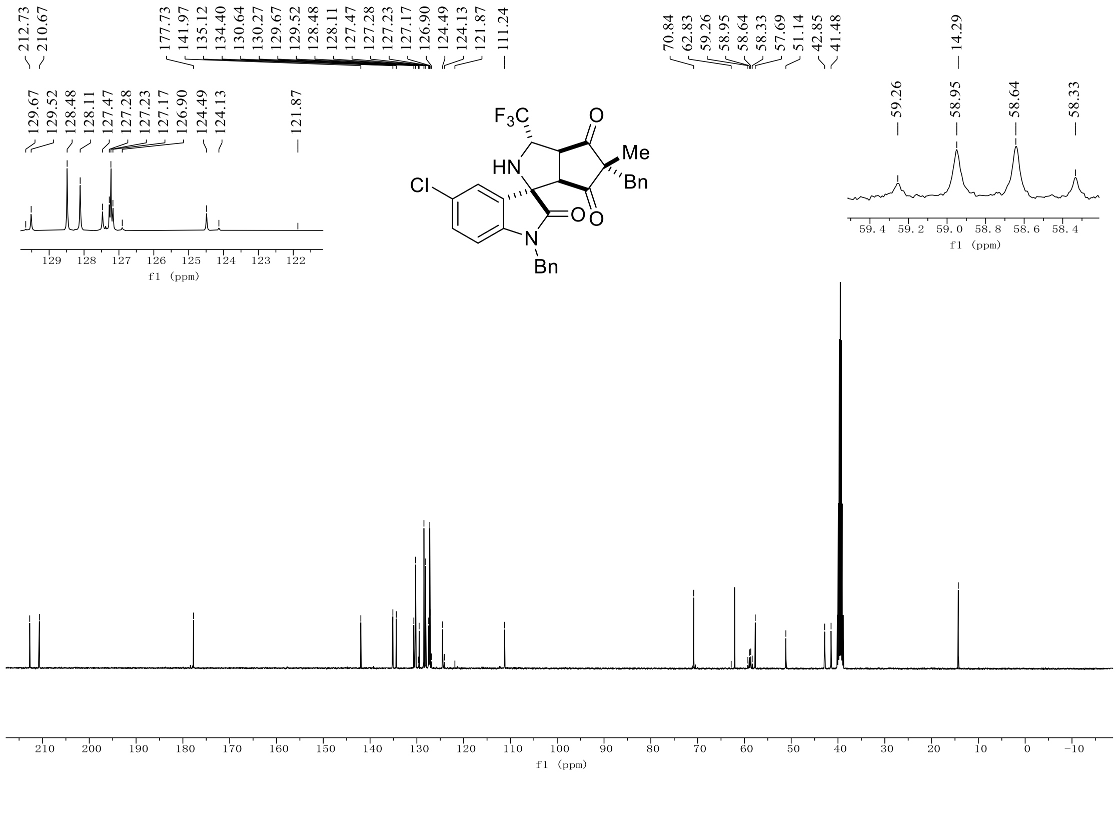

^{1}H NMR (400 MHz, DMSO-d_6) of **3ha**

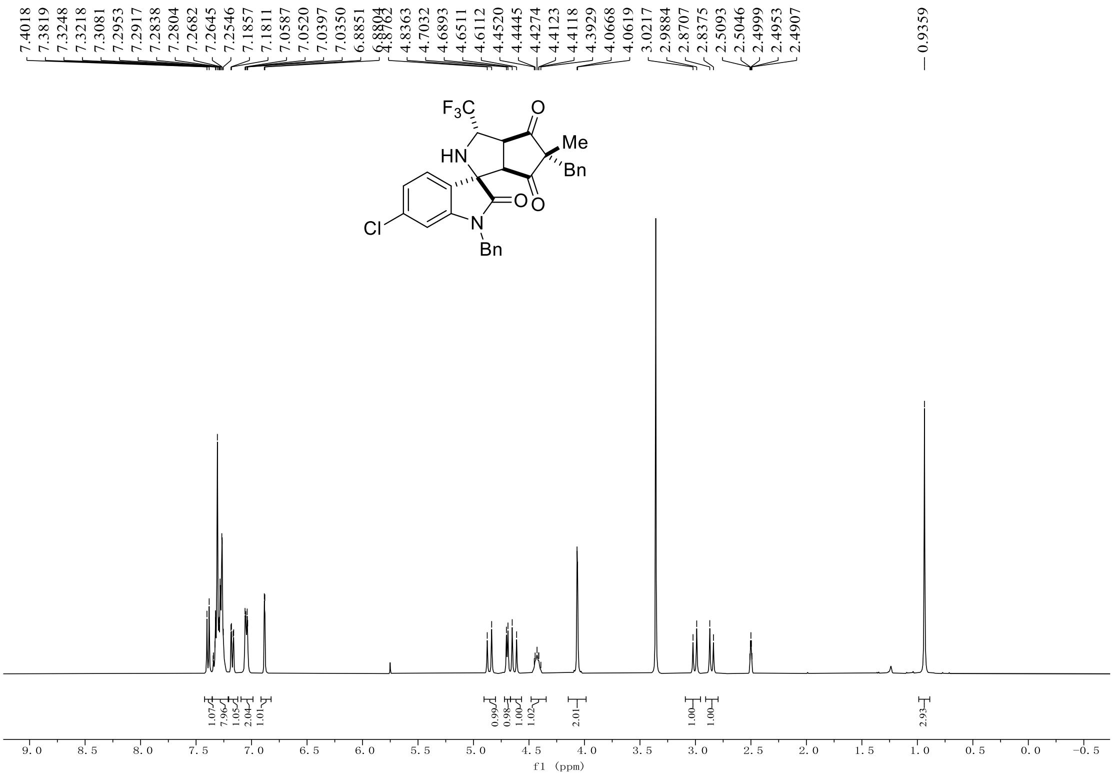

^{13}C{^{1}H} NMR (101 MHz, DMSO-d_6) of **3ha**

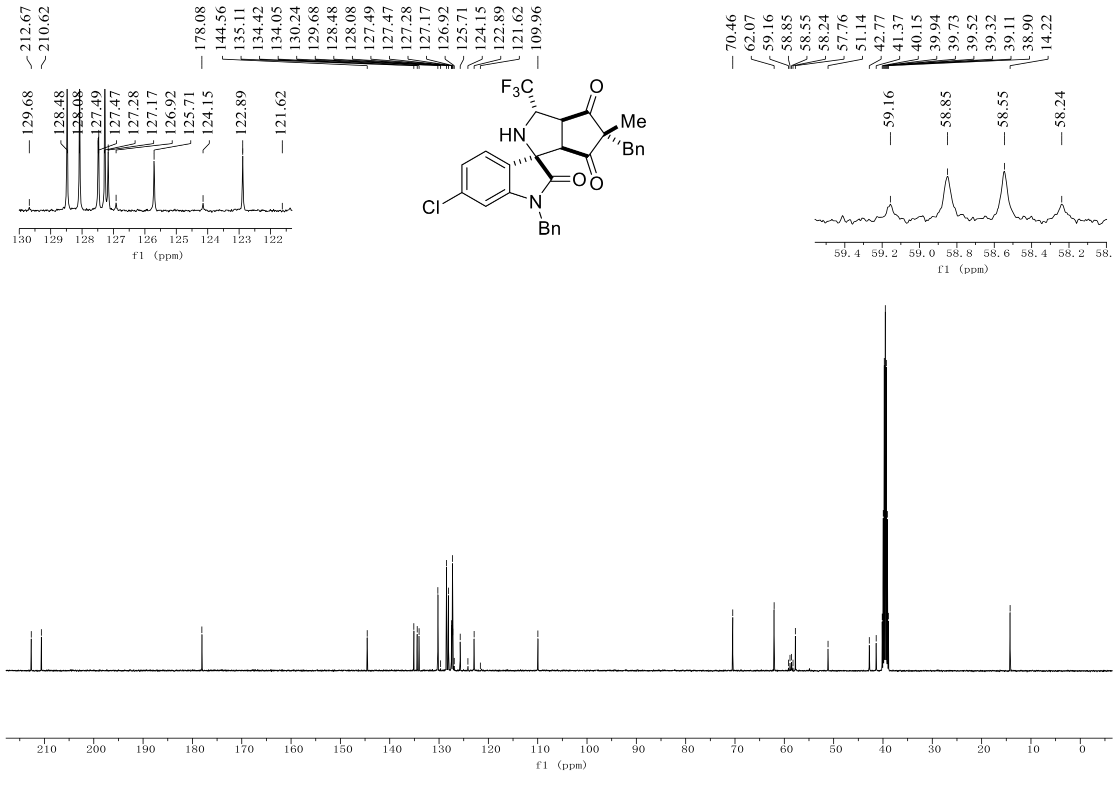

^{1}H NMR (400 MHz, $CDCl_3$) of **3ia**

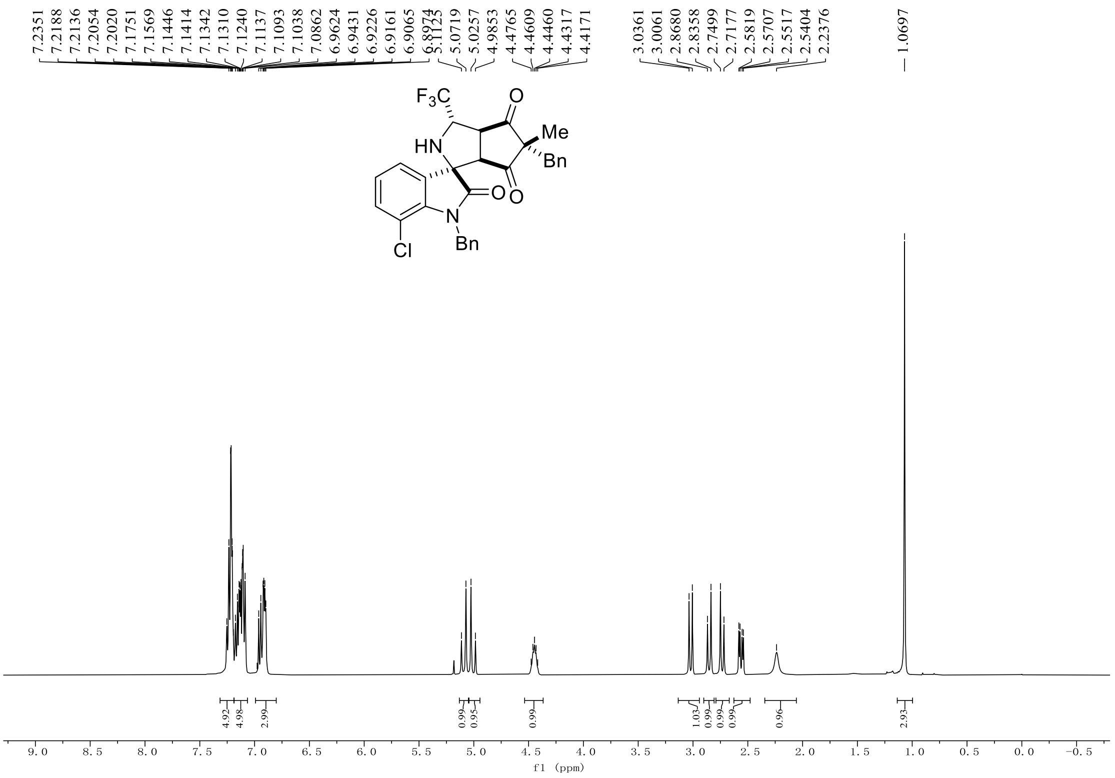

^{13}C{^{1}H} NMR (101 MHz, $CDCl_3$) of **3ia**

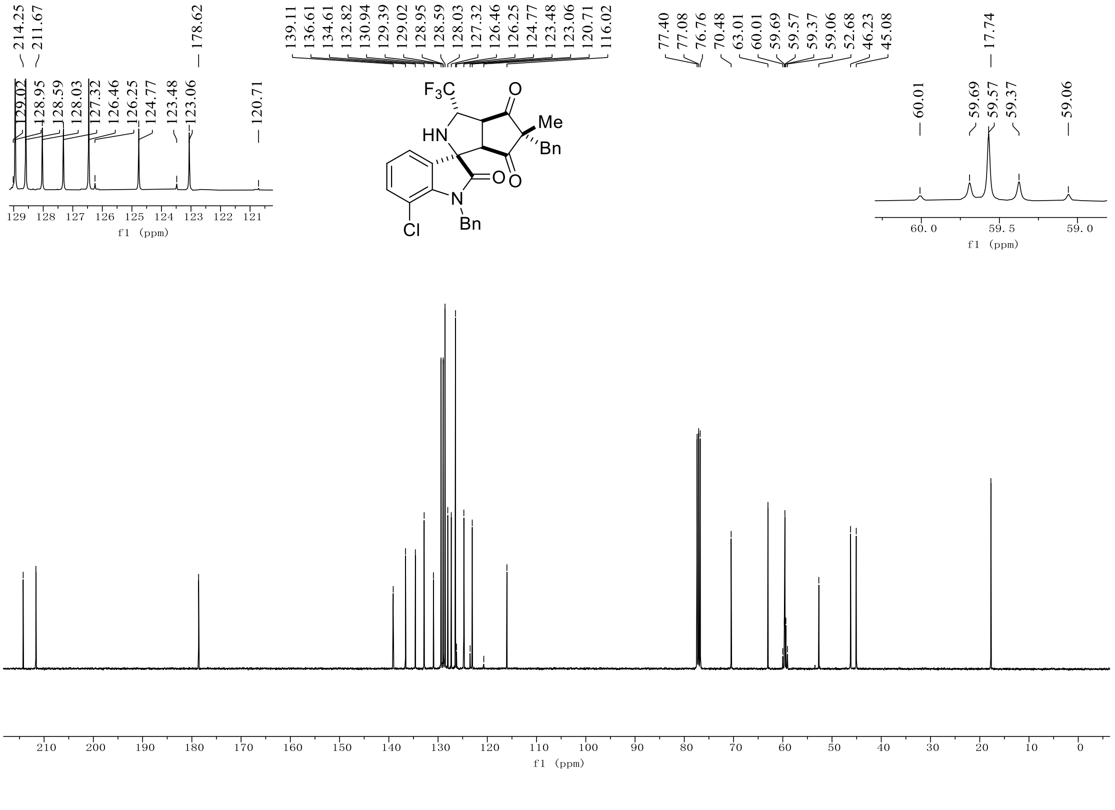

^{1}H NMR (400 MHz, DMSO-d_6) of **3ja**

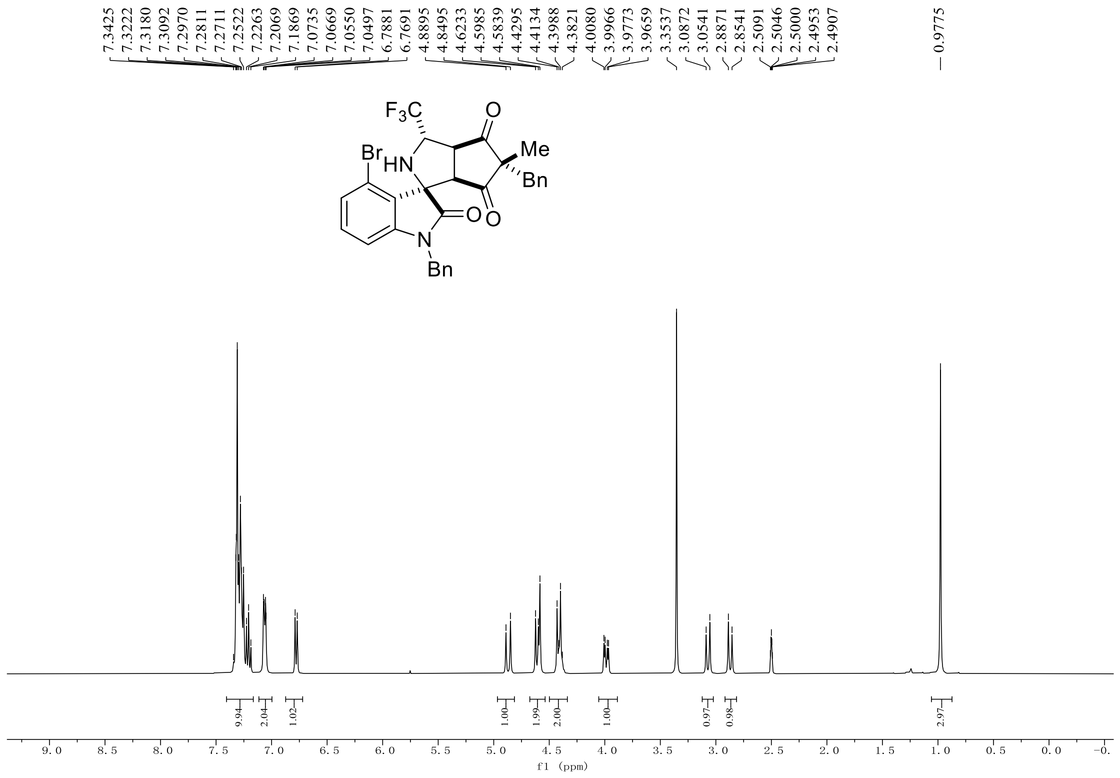

$^{13}C\{^{1}H\}$ NMR (101 MHz, DMSO-d_6) of **3ja**

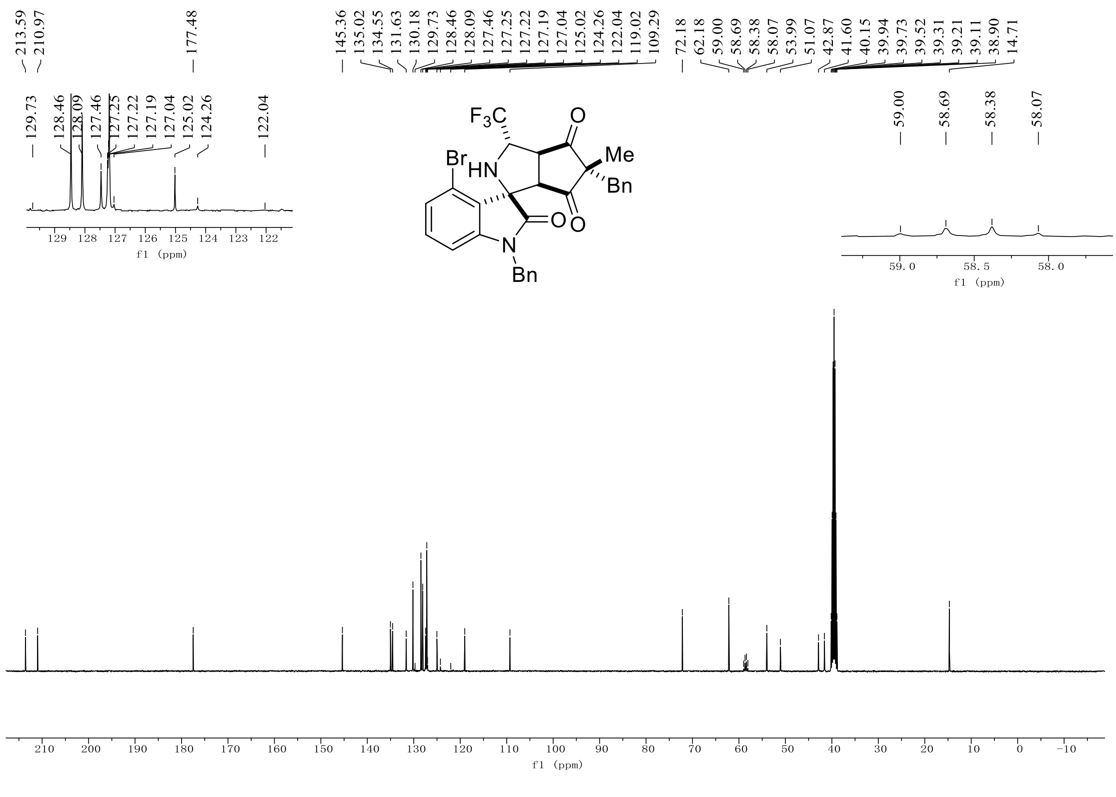

^{1}H NMR (400 MHz, DMSO-d_6) of **3ka**

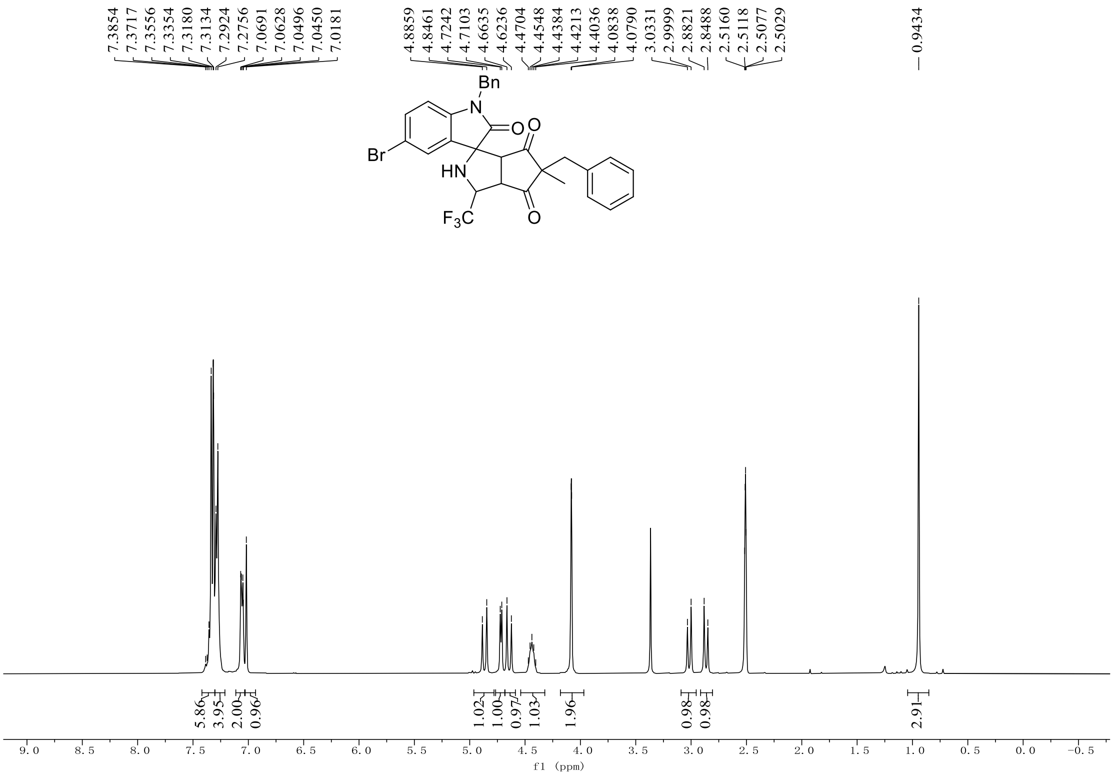

^{13}C{^{1}H} NMR (101 MHz, DMSO-d_6) of **3ka**

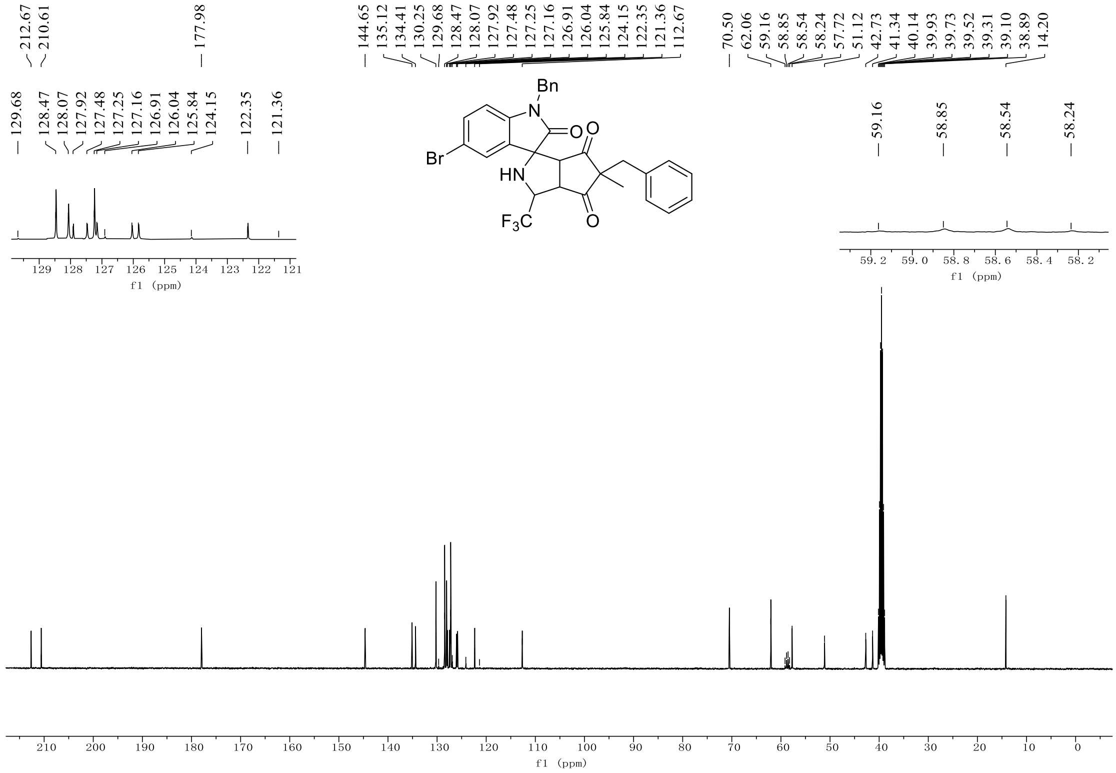

^{1}H NMR (400 MHz, DMSO-d_6) of **3la**

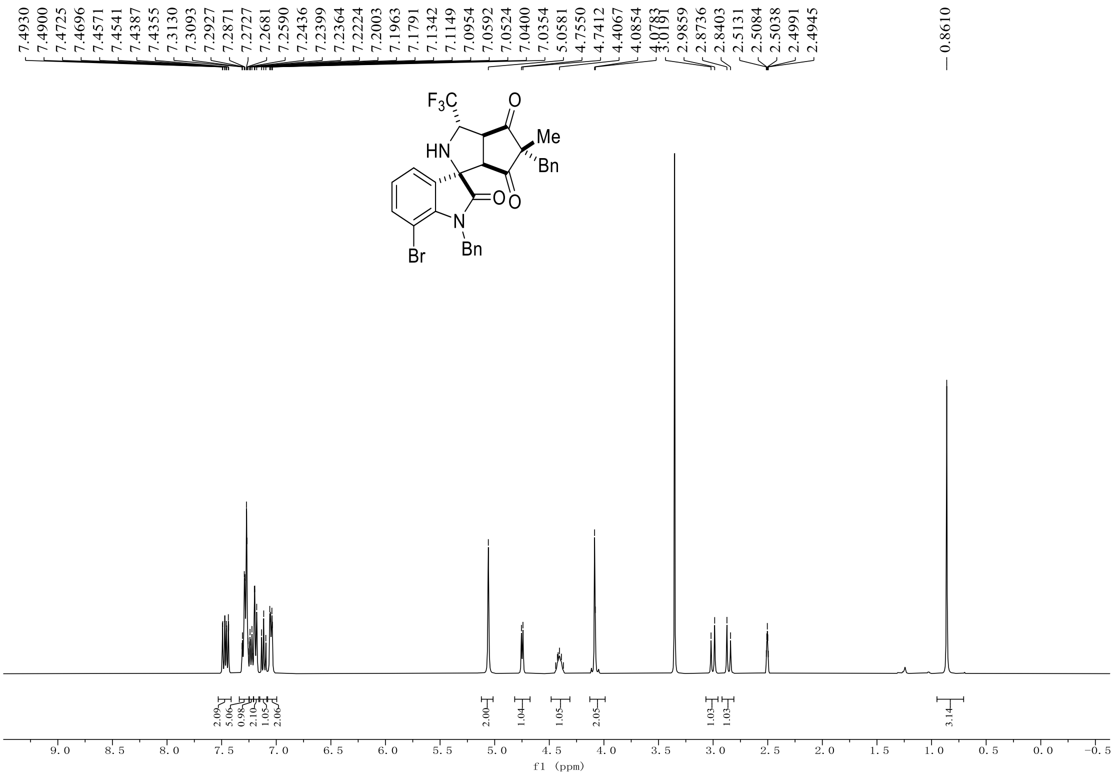

$^{13}C\{^{1}H\}$ NMR (101 MHz, DMSO-d_6) of **3la**

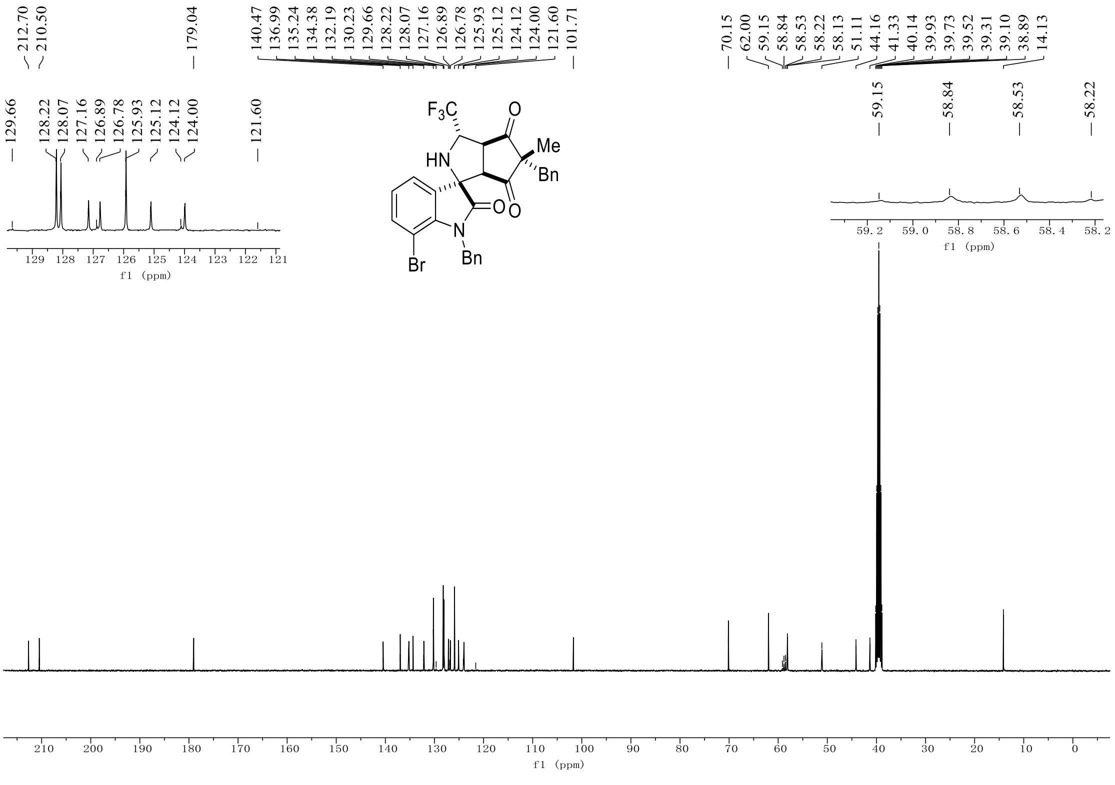

^{1}H NMR (400 MHz, DMSO-d_6) of **3ma**

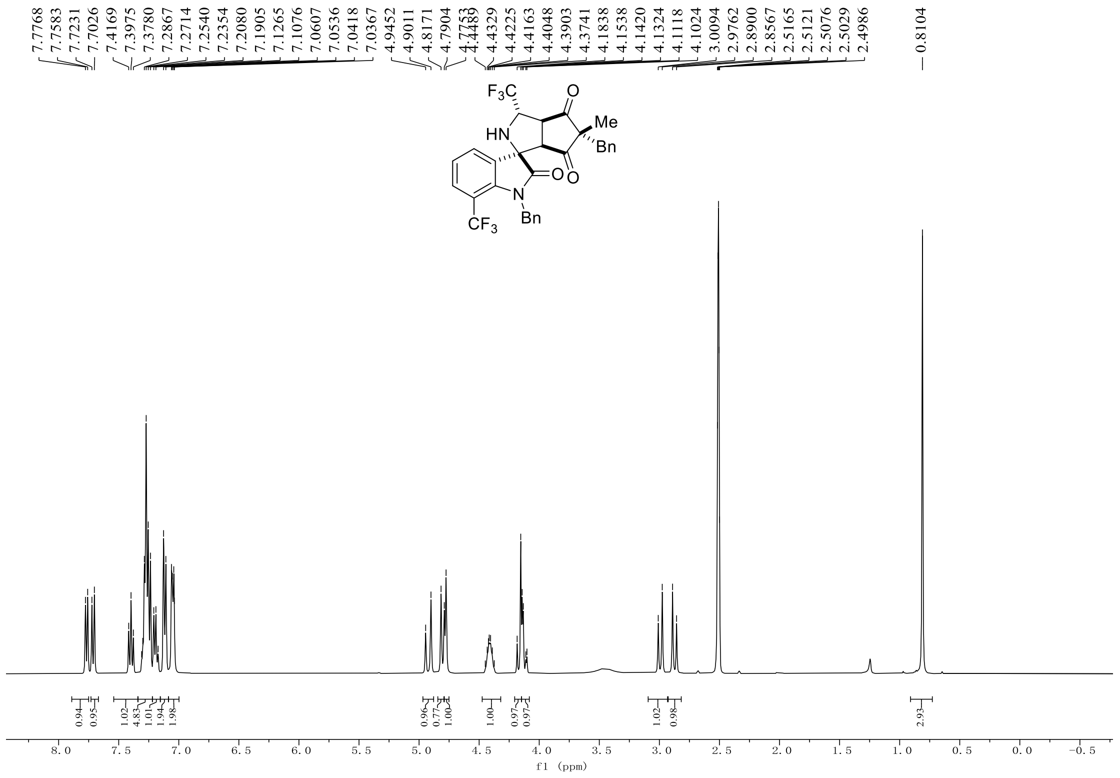

^{13}C{^{1}H} NMR (101 MHz, DMSO-d_6) of **3ma**

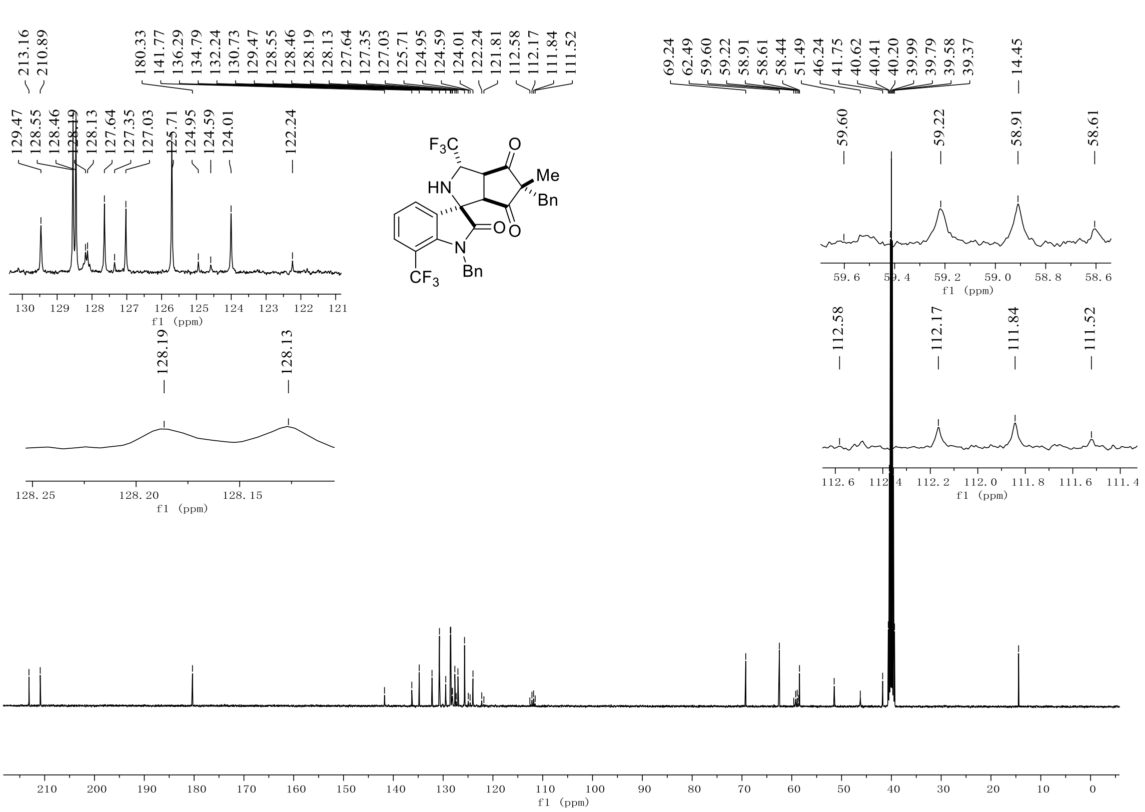

^{1}H NMR (400 MHz, DMSO-d_6) of **3na**

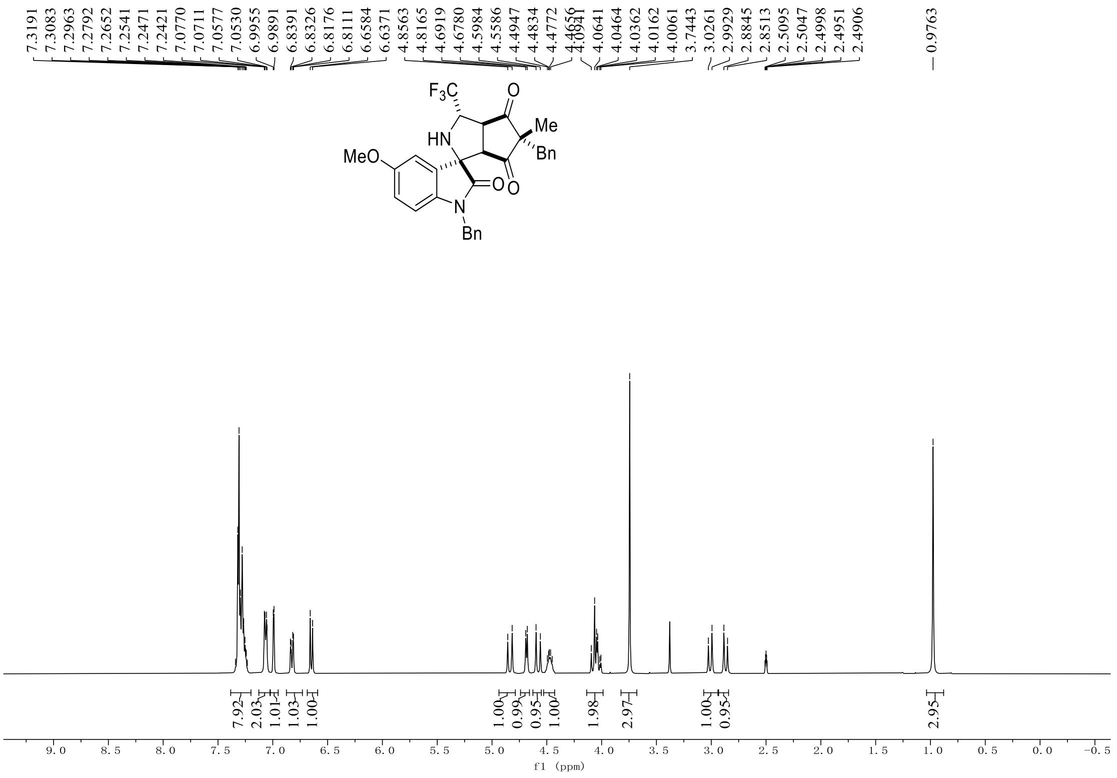

^{13}C{^{1}H} NMR (101 MHz, DMSO-d_6) of **3na**

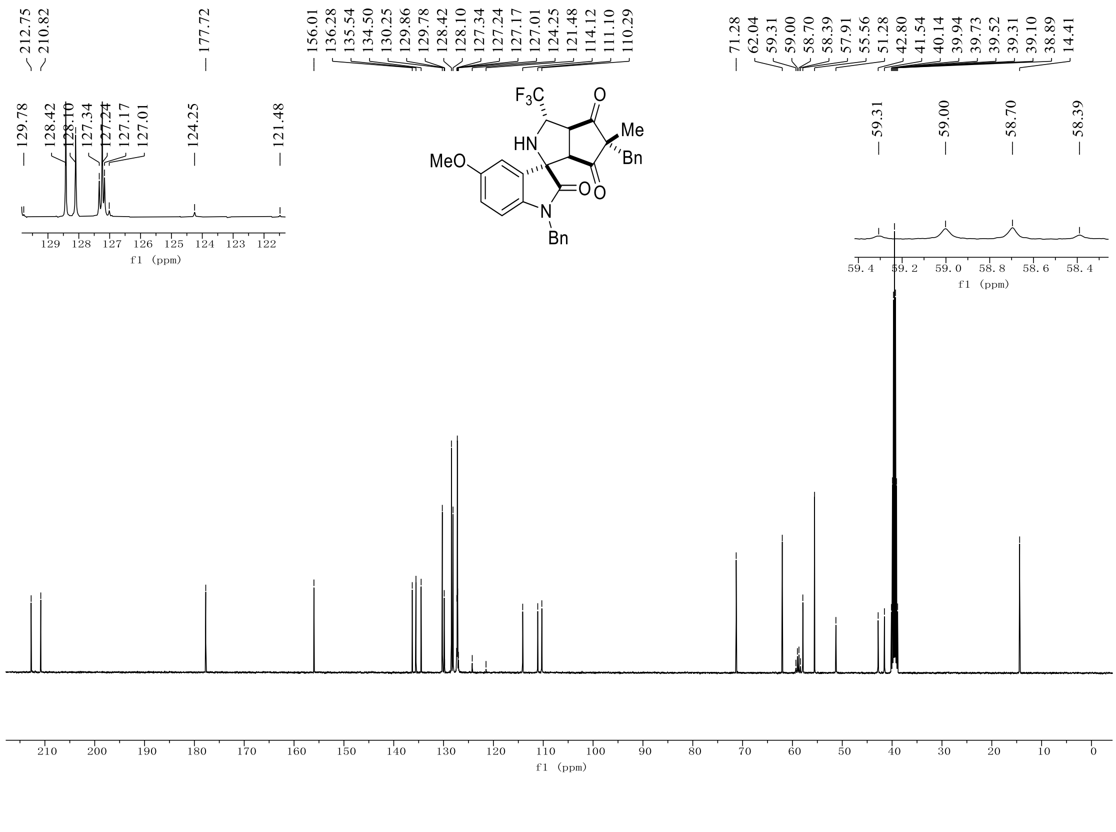

^{1}H NMR (400 MHz, DMSO-d_6) of **3oa**

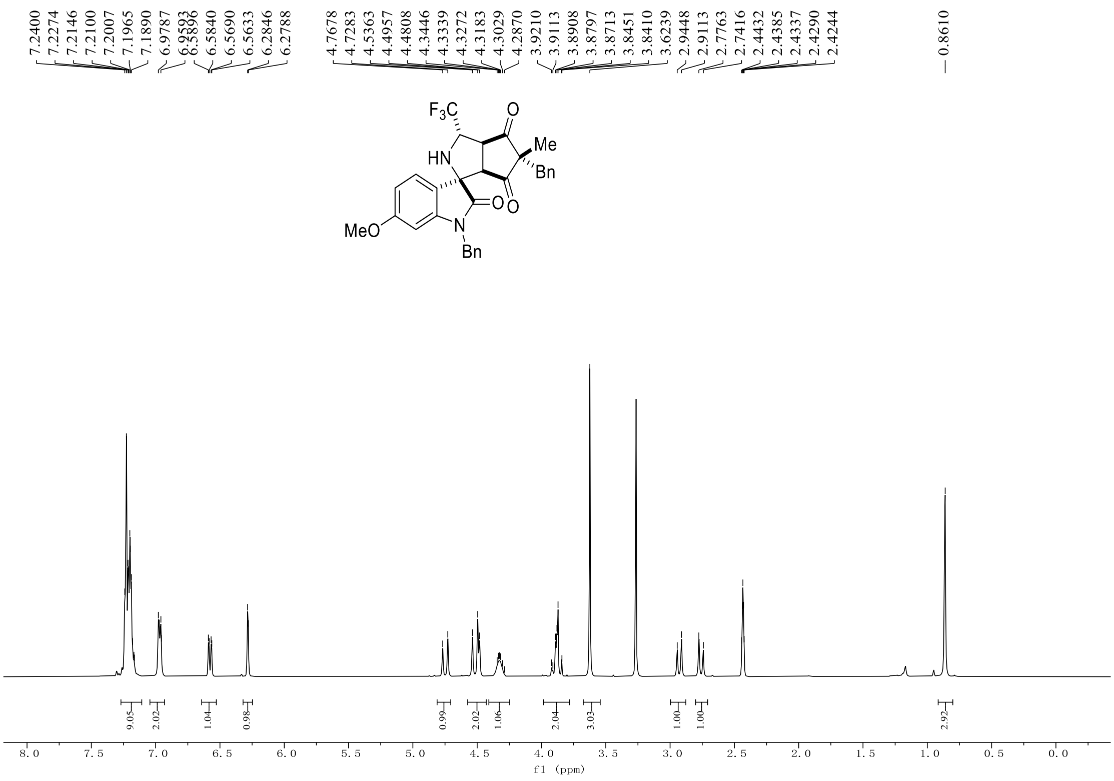

^{13}C{^{1}H} NMR (101 MHz, DMSO-d_6) of **3oa**

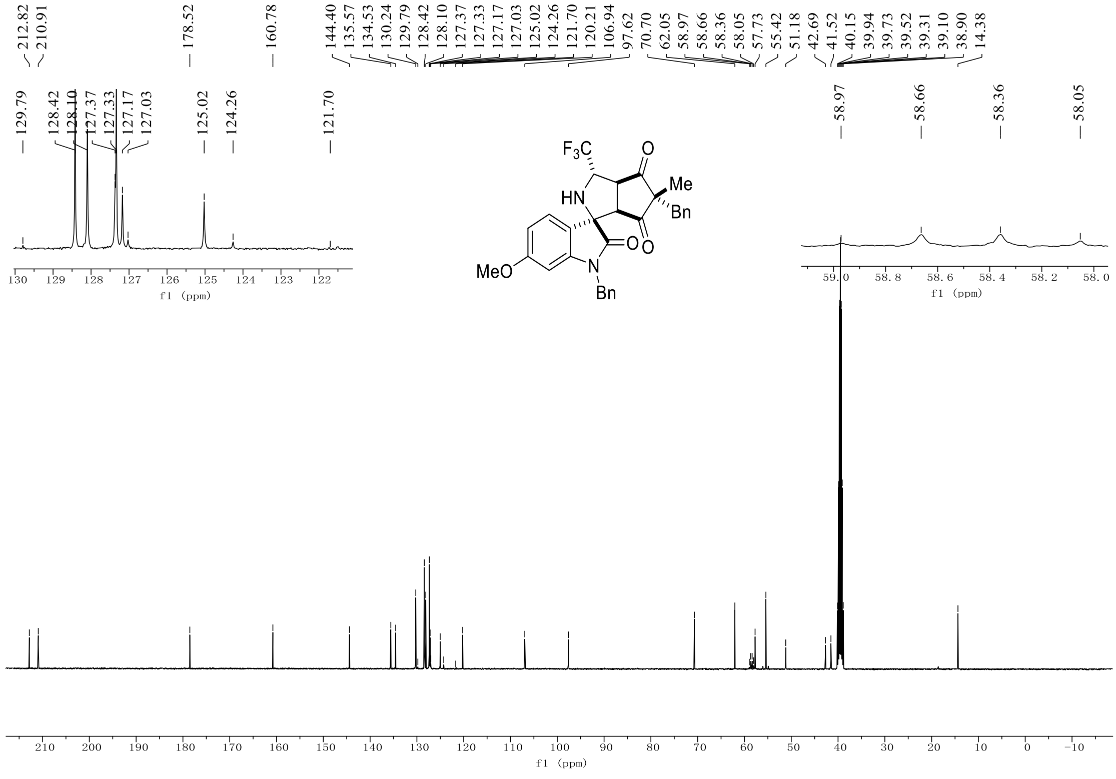

^{1}H NMR (400 MHz, DMSO-d_6) of **3pa**

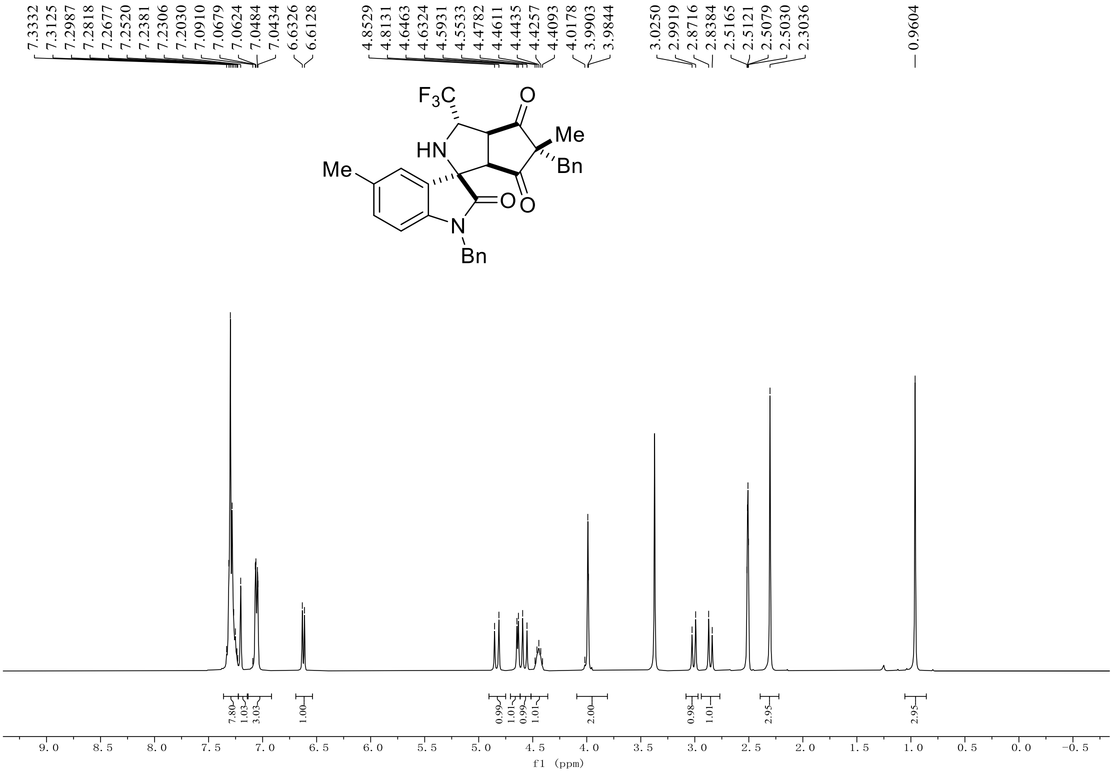

^{13}C{^{1}H} NMR (101 MHz, DMSO-d_6) of **3pa**

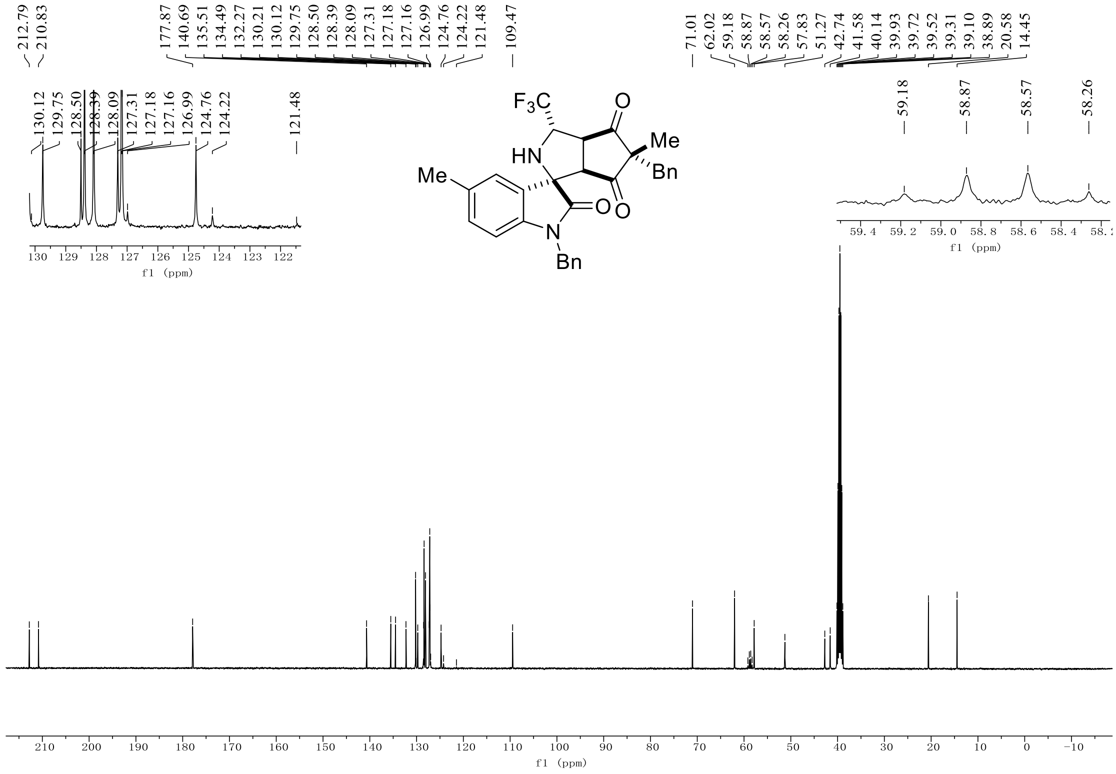

^{1}H NMR (400 MHz, $CDCl_3$) of **3qa**

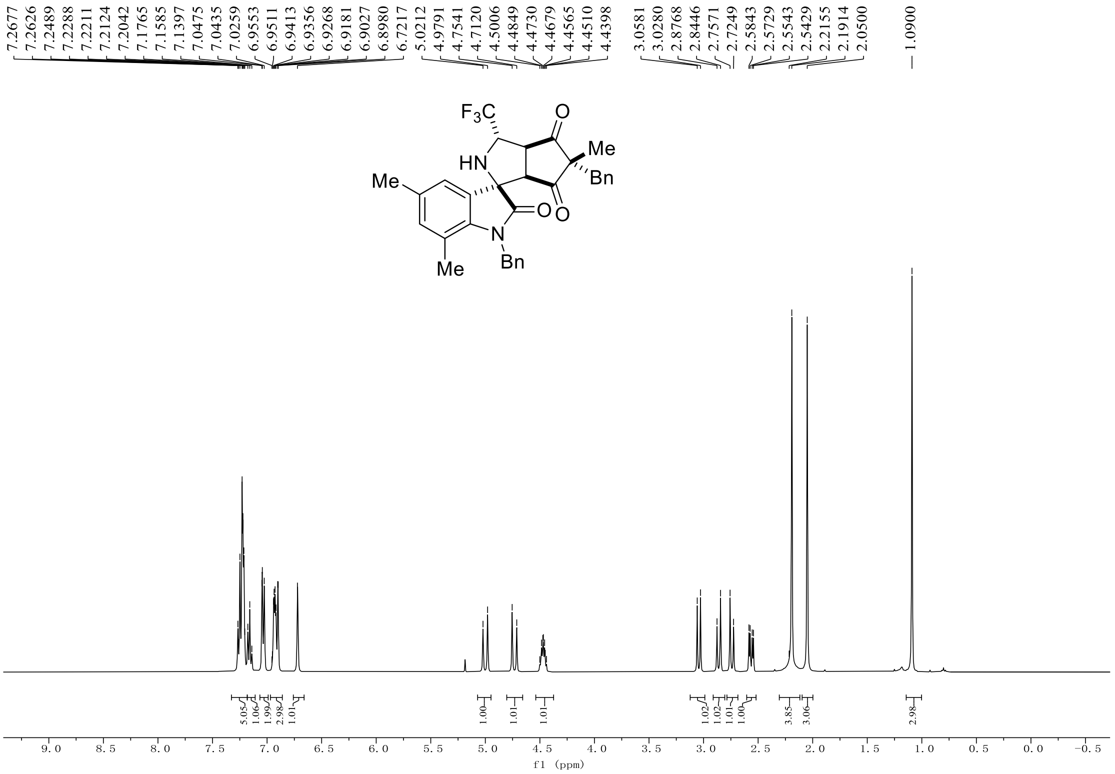

$^{13}C\{^{1}H\}$ NMR (101 MHz, $CDCl_3$) of **3qa**

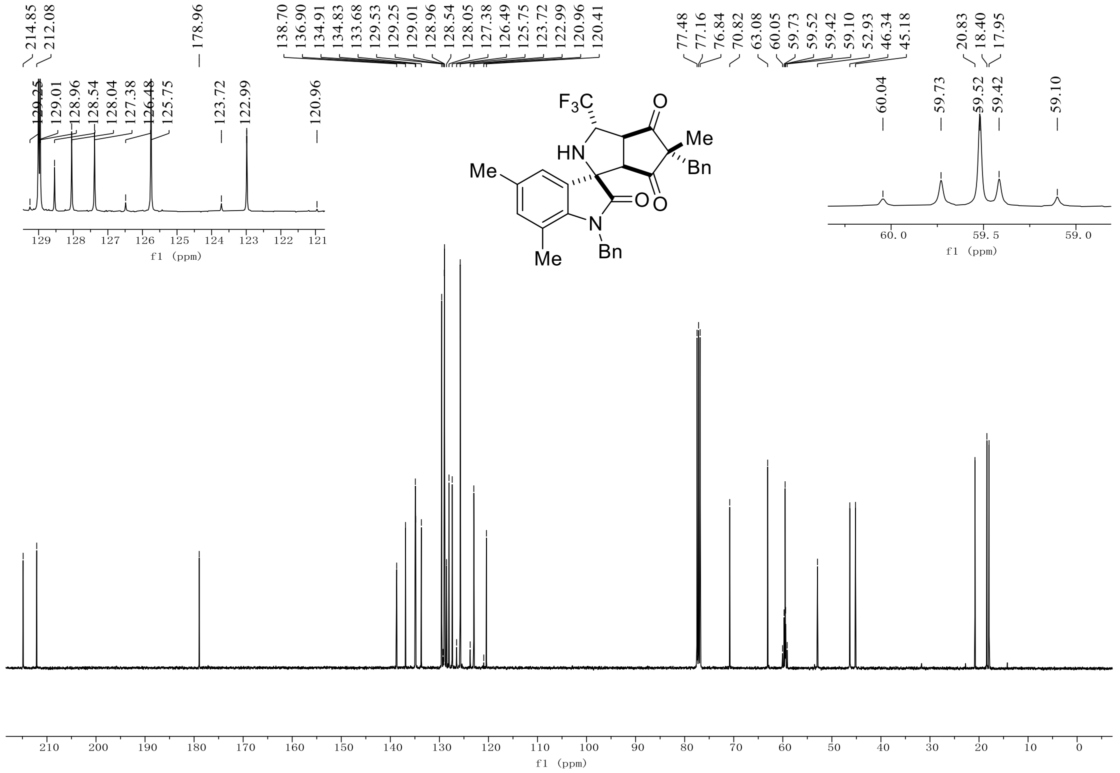

^1H NMR (400 MHz, DMSO-d_6) of **3ab**

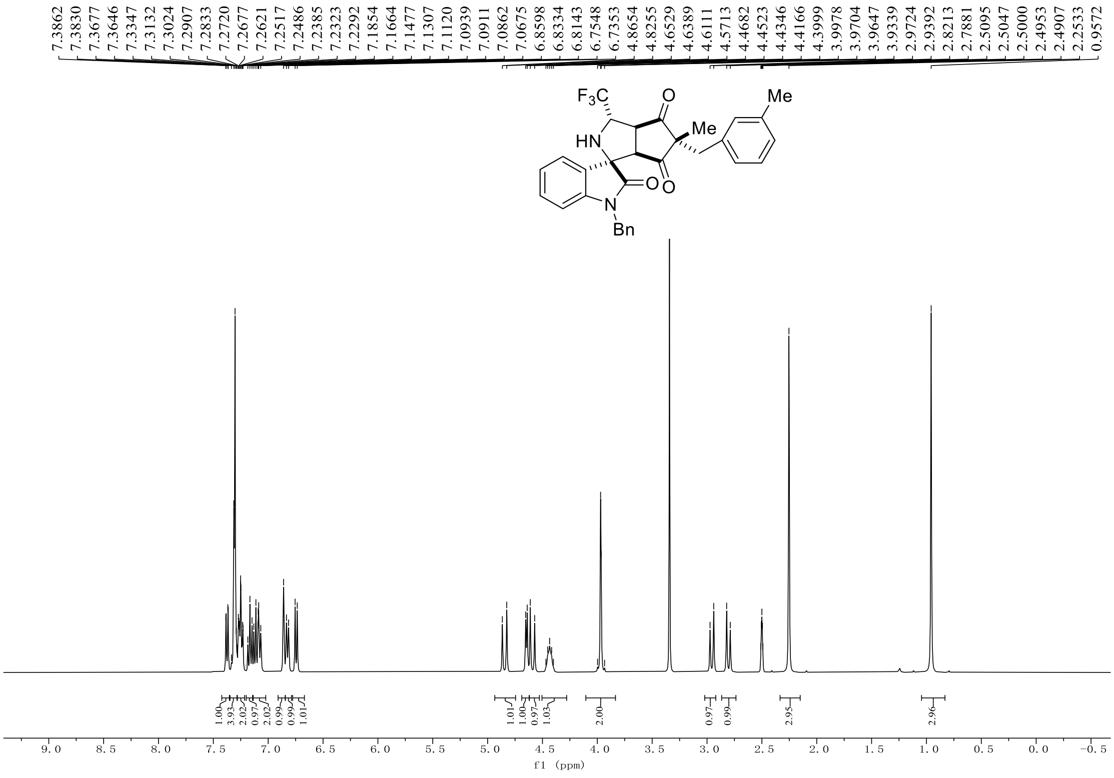

$^{13}C\{^1H\}$ NMR (101 MHz, DMSO-d_6) of **3ab**

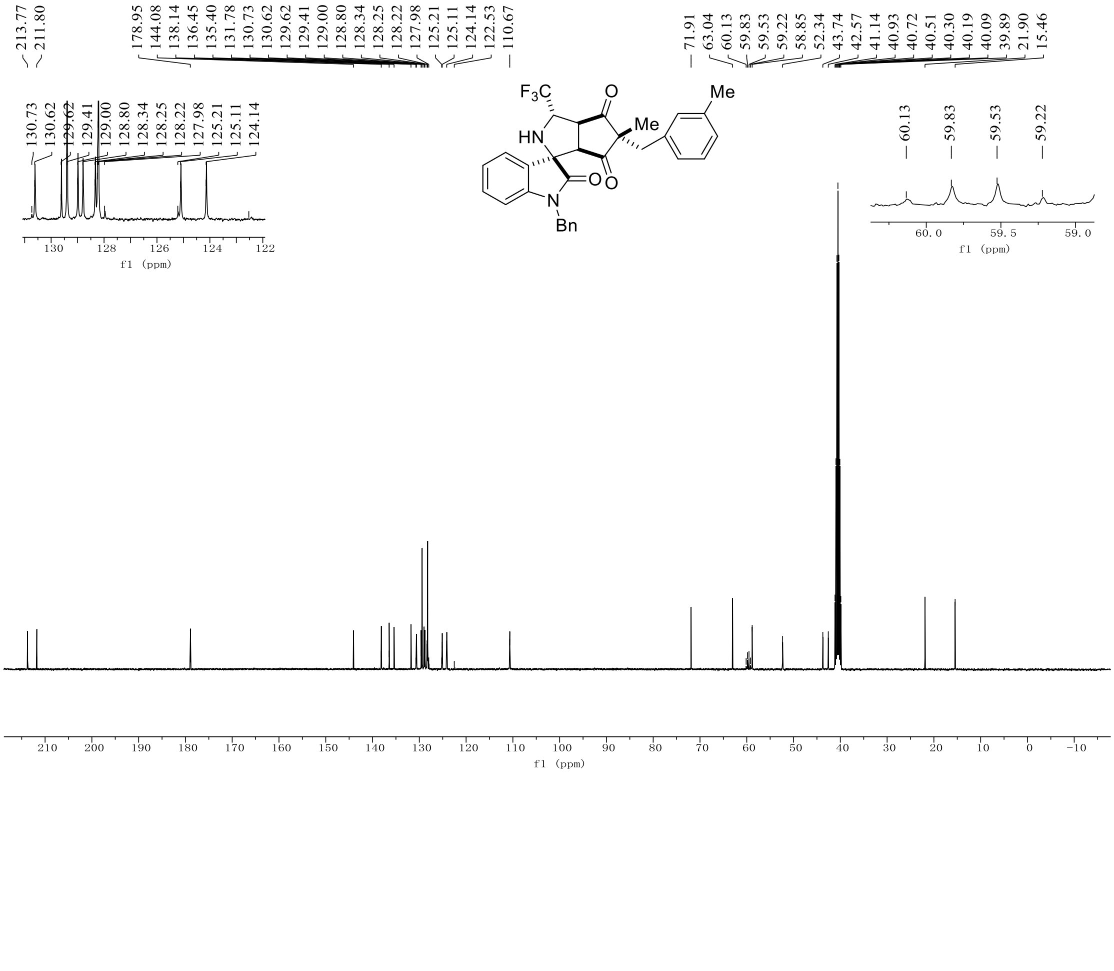

^{1}H NMR (400 MHz, $CDCl_3$) of **3ac**

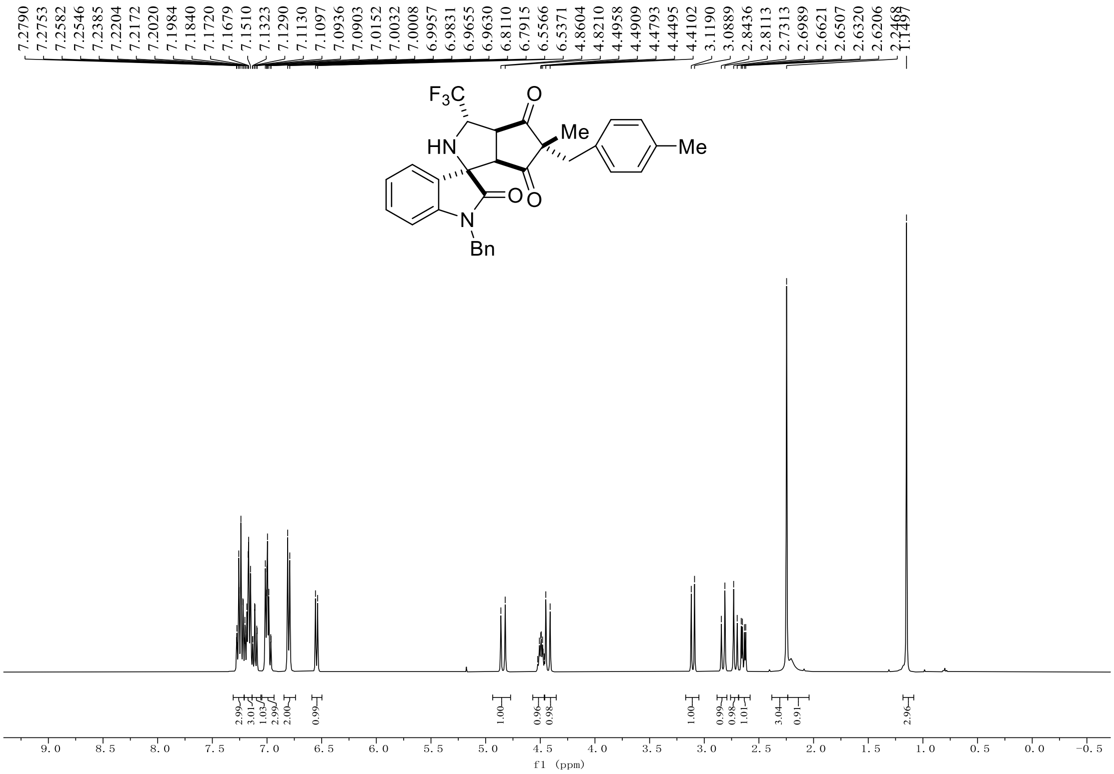

$^{13}C\{^{1}H\}$ NMR (101 MHz, $CDCl_3$) of **3ac**

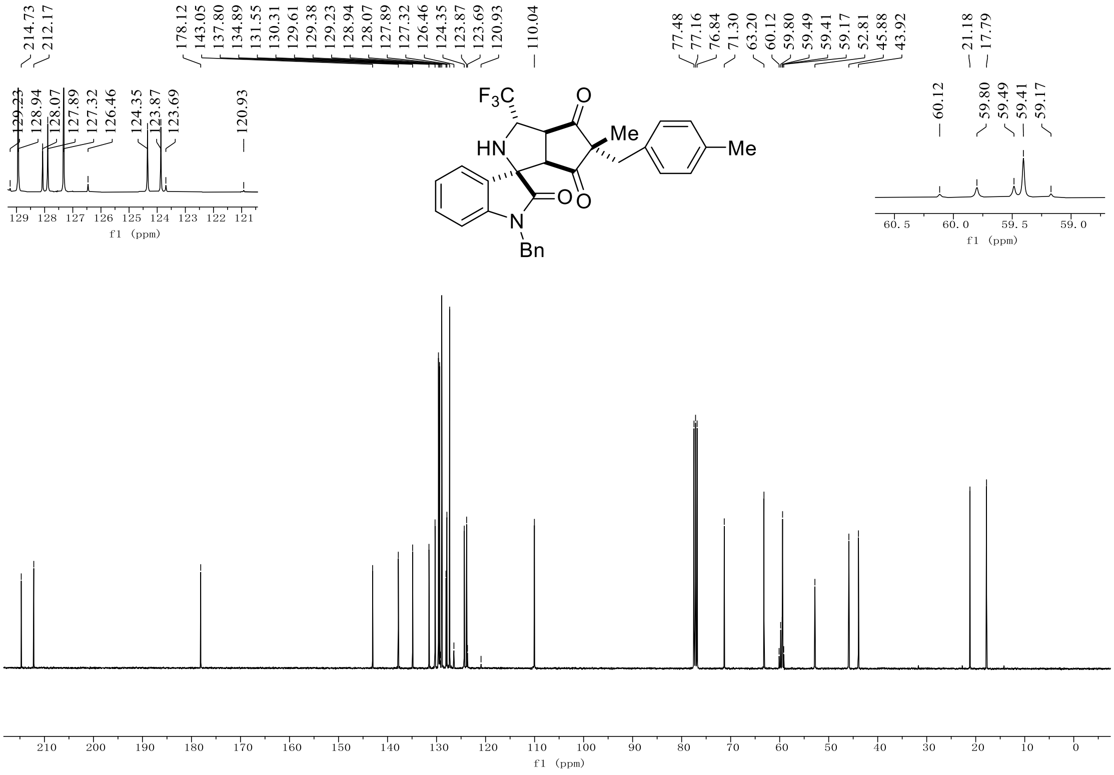

^{1}H NMR (400 MHz, $CDCl_3$) of **3ad**

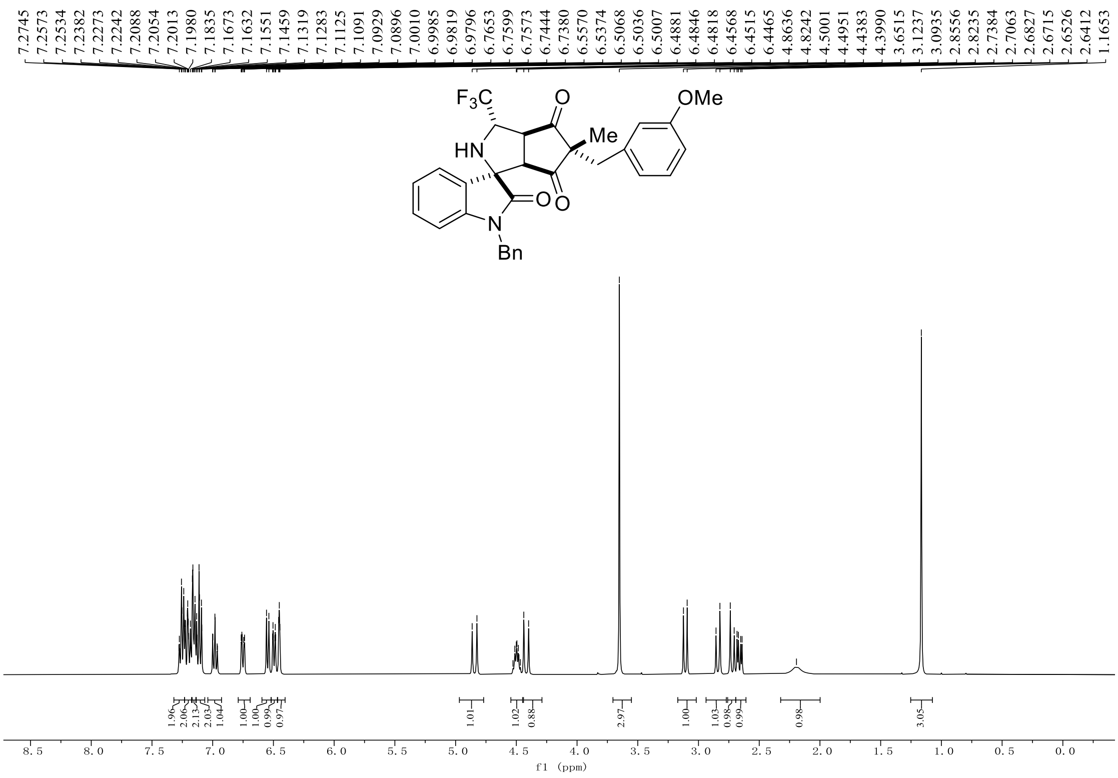

^{13}C{^{1}H} NMR (101 MHz, $CDCl_3$) of **3ad**

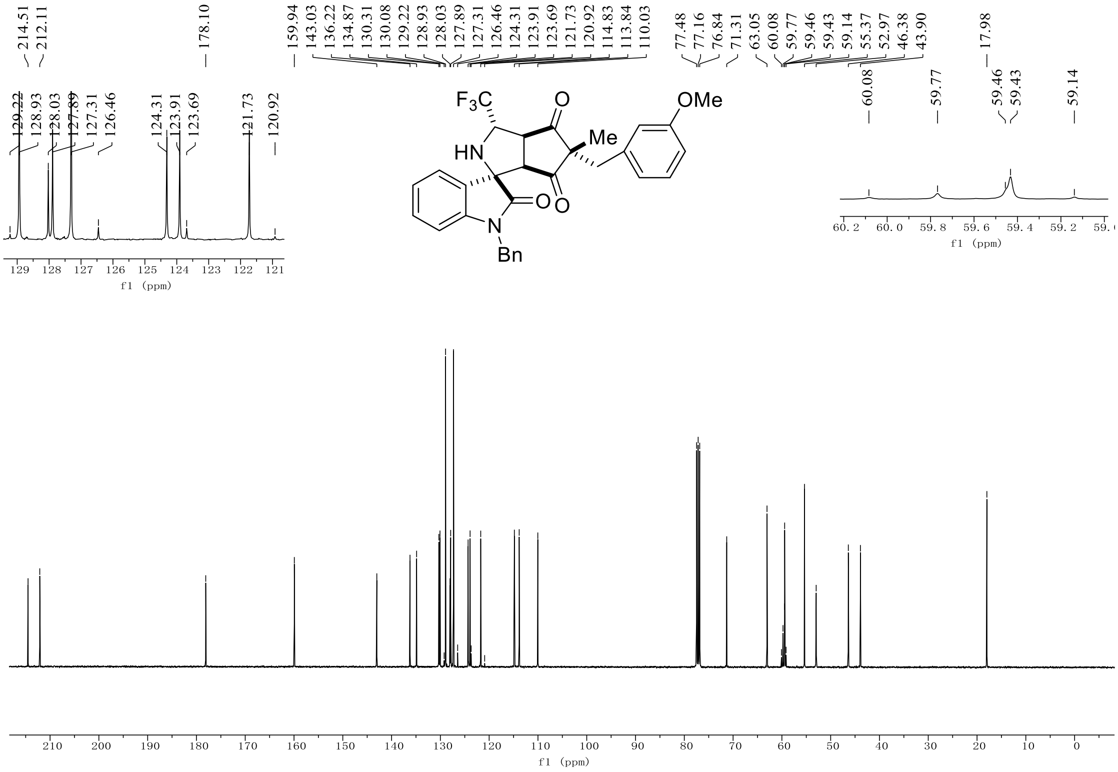

^{1}H NMR (400 MHz, $CDCl_3$) of **3ae**

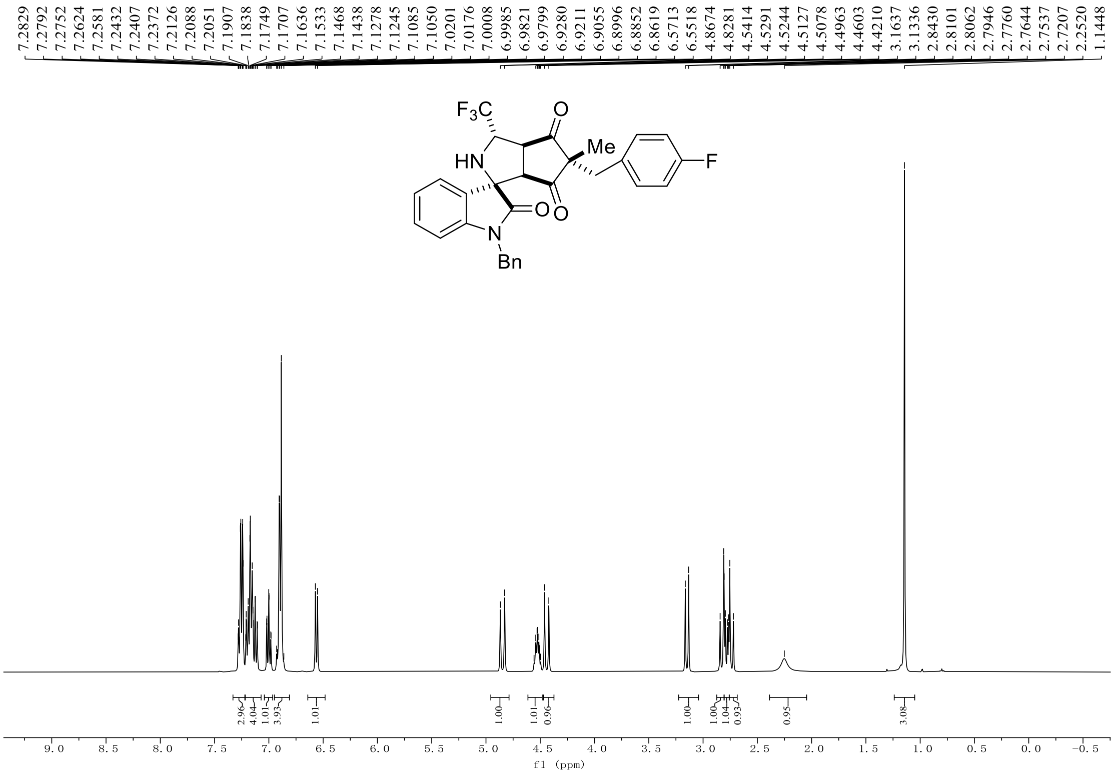

^{13}C{^{1}H} NMR (101 MHz, $CDCl_3$) of **3ae**

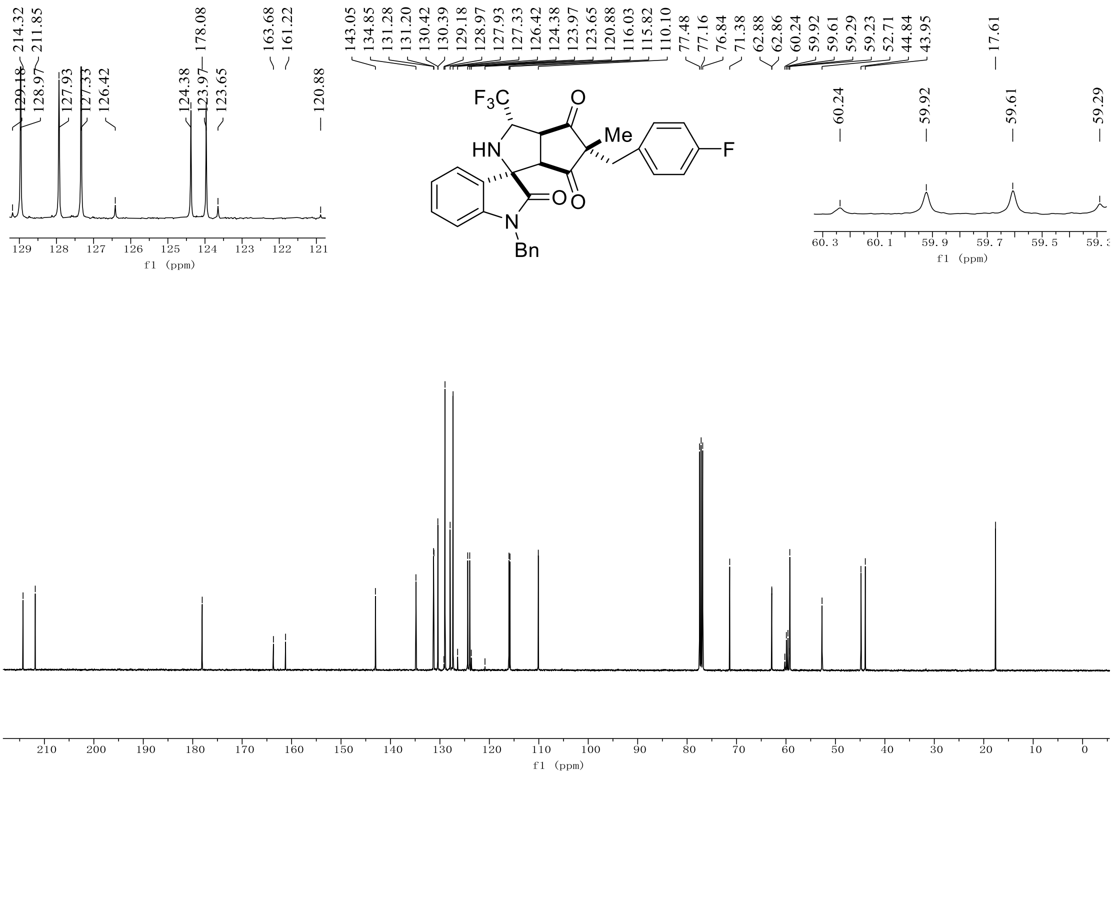

^{1}H NMR (400 MHz, $CDCl_3$) of **3af**

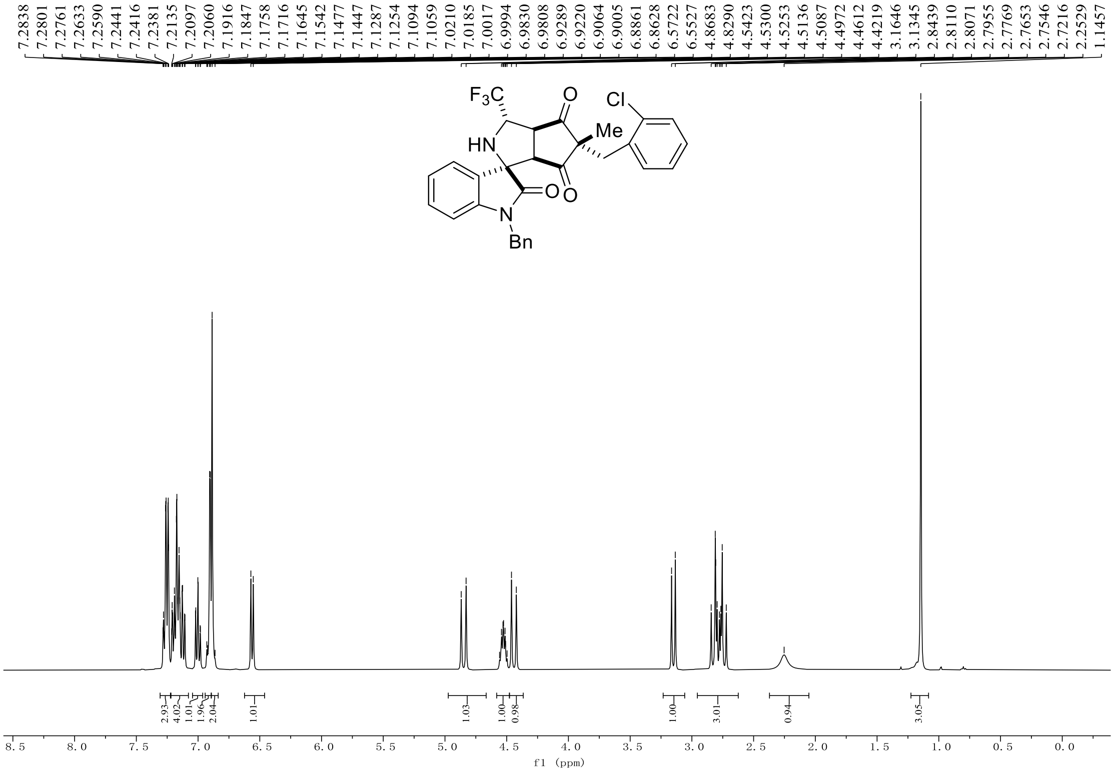

^{13}C{^{1}H} NMR (101 MHz, $CDCl_3$) of **3af**

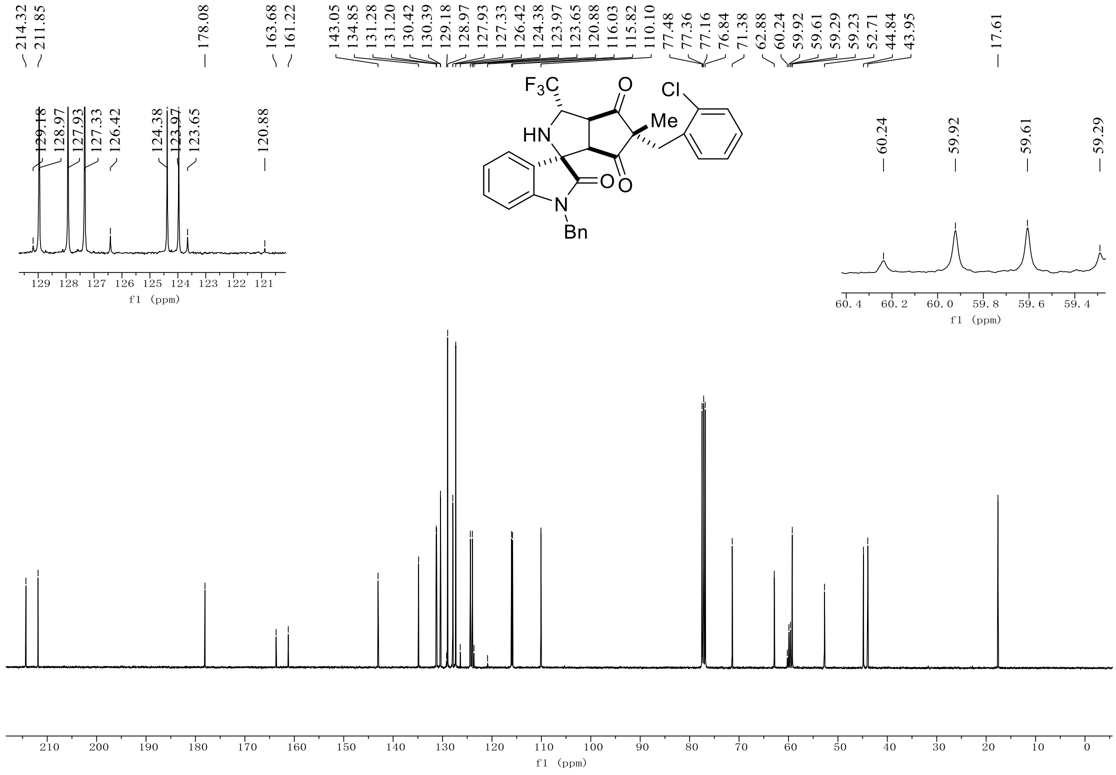

^{1}H NMR (400 MHz, $CDCl_3$) of **3ag**

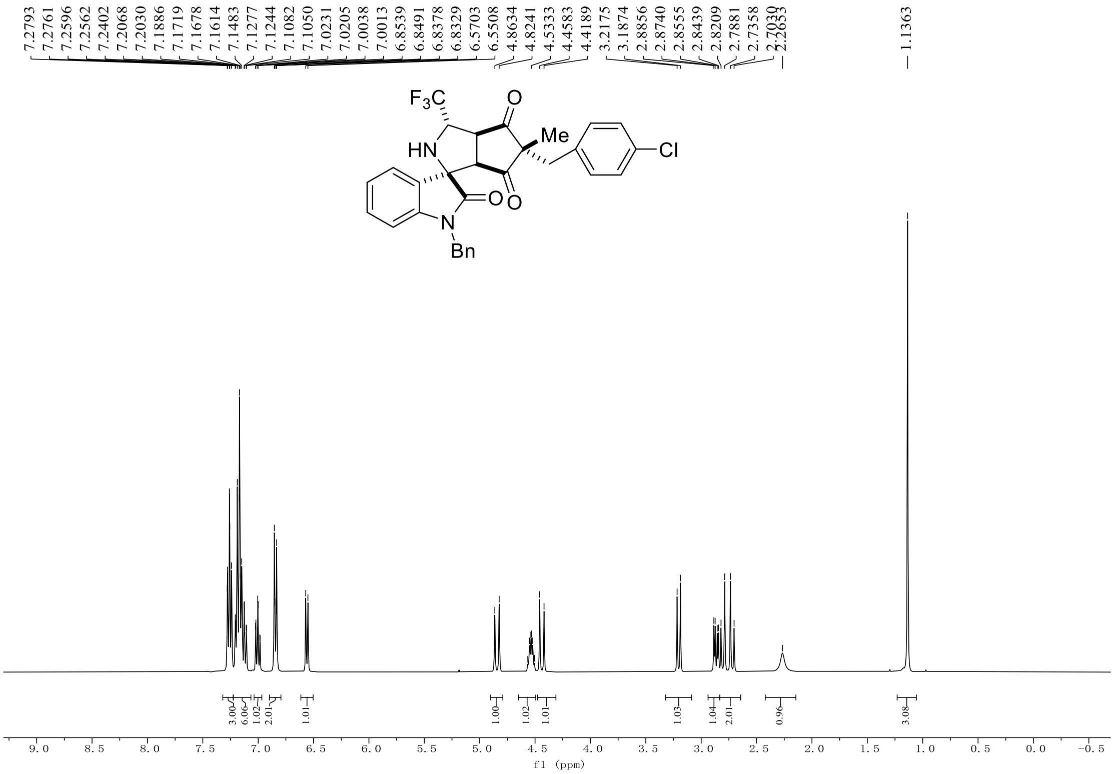

^{13}C{^{1}H} NMR (101 MHz, $CDCl_3$) of **3ag**

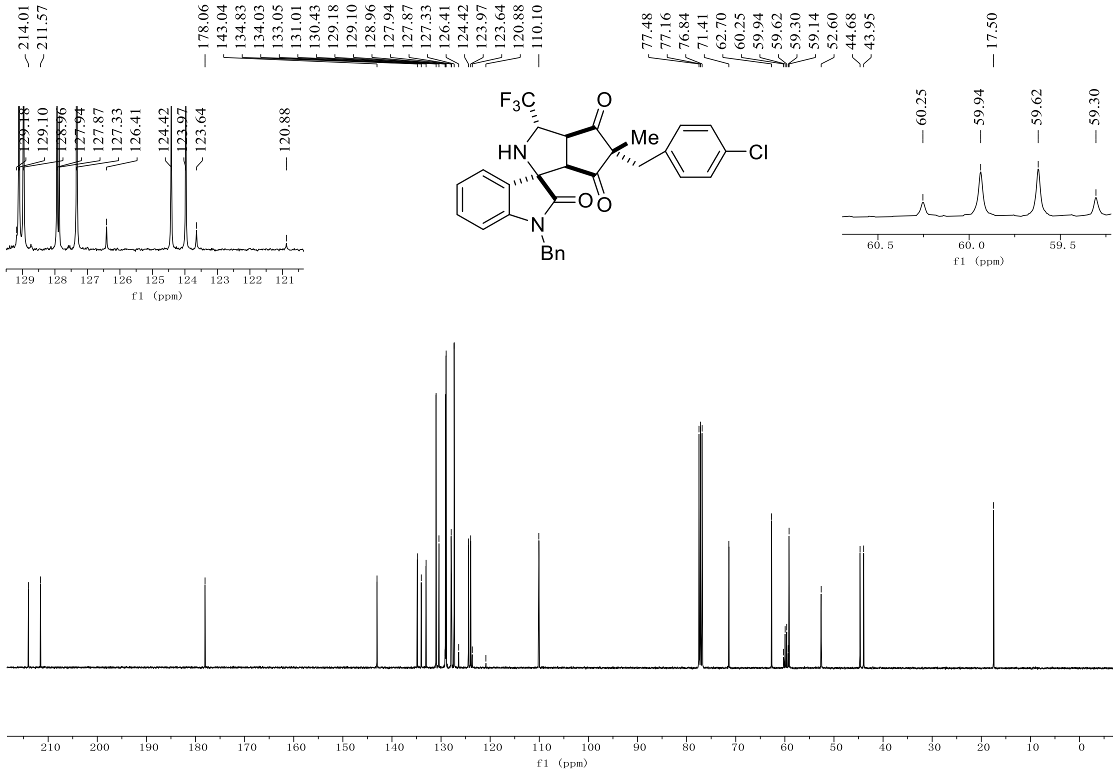

^{1}H NMR (400 MHz, $CDCl_3$) of **3ah**

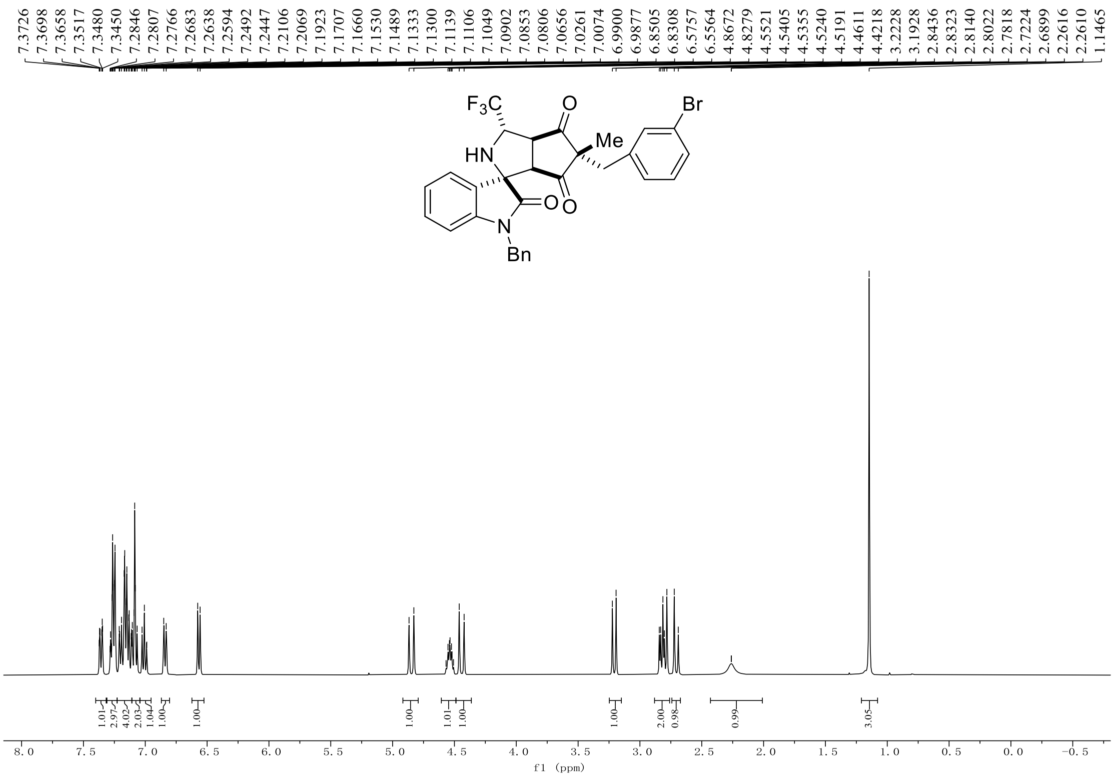

$^{13}C\{^{1}H\}$ NMR (101 MHz, $CDCl_3$) of **3ah**

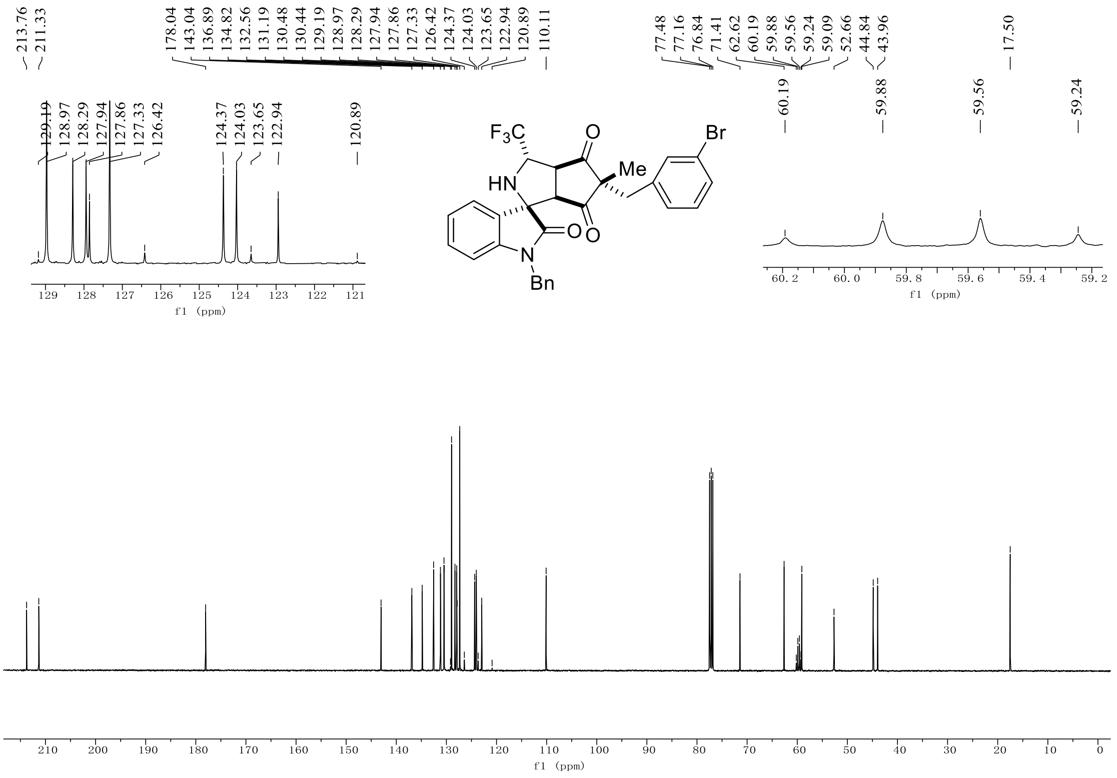

^{1}H NMR (400 MHz, DMSO-d_6) of **3ai**

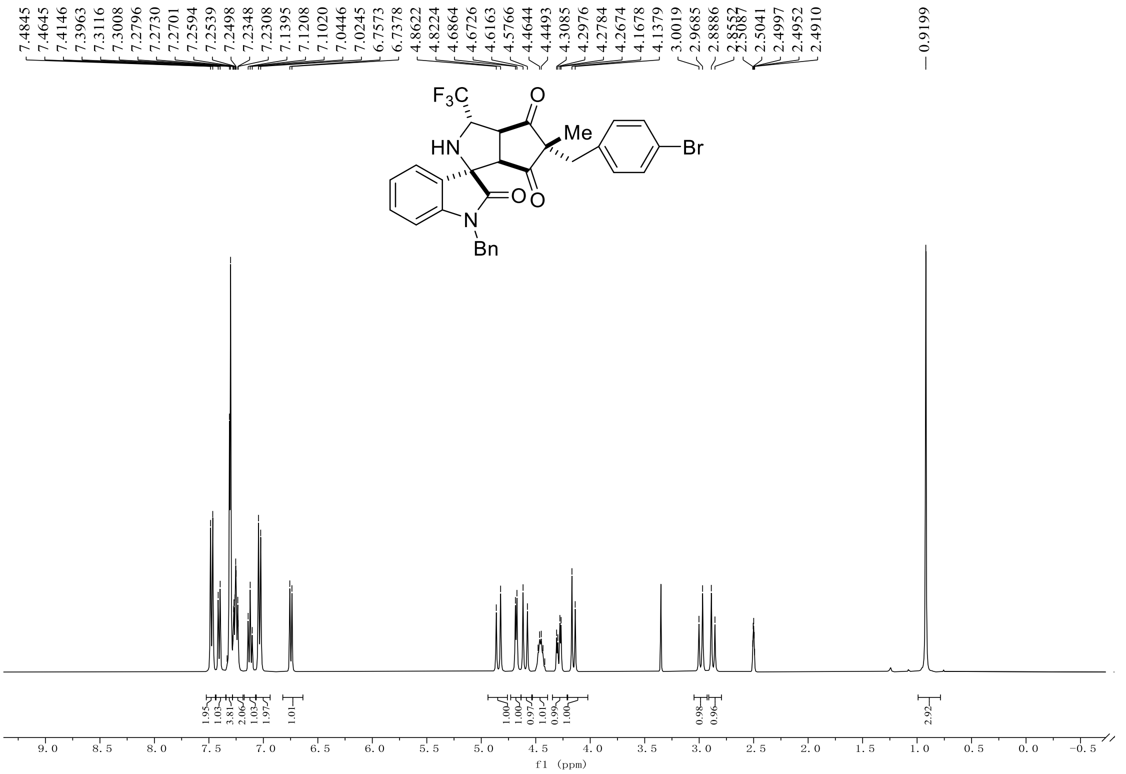

^{13}C{^{1}H} NMR (101 MHz, DMSO-d_6) of **3gi**

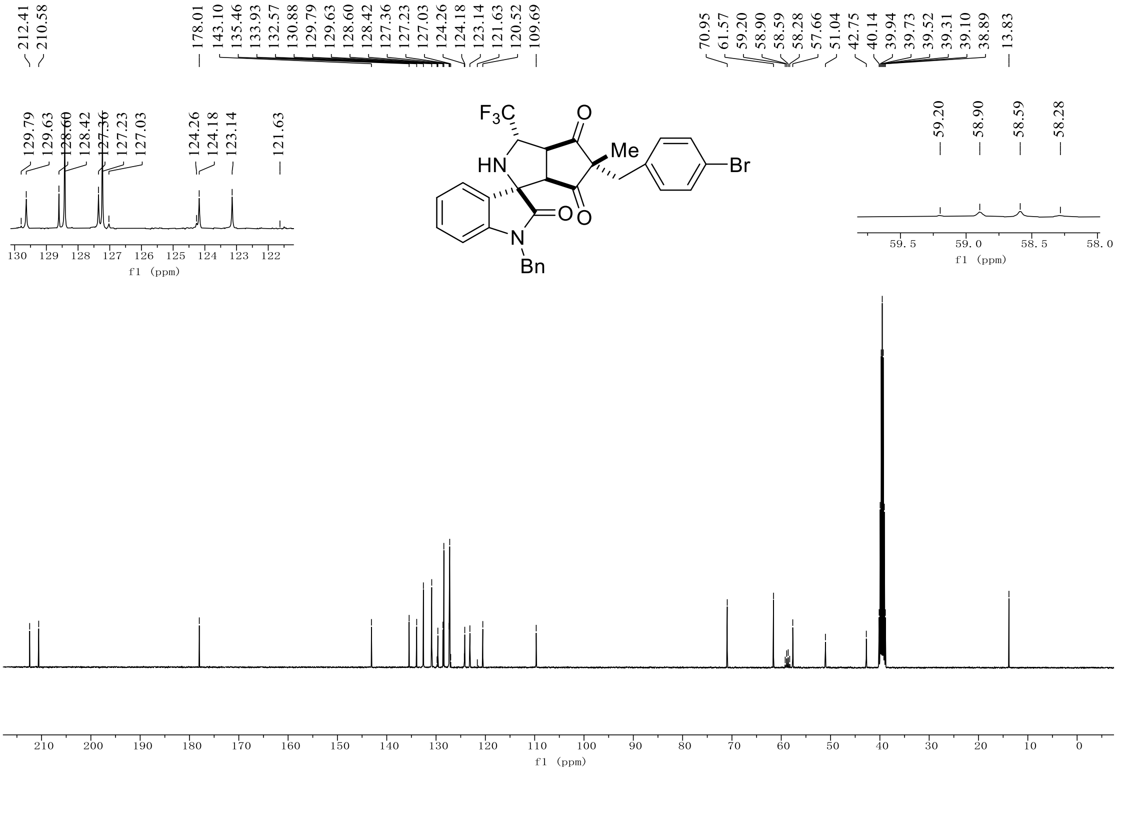

^{1}H NMR (400 MHz, DMSO-d_6) of **3aj**

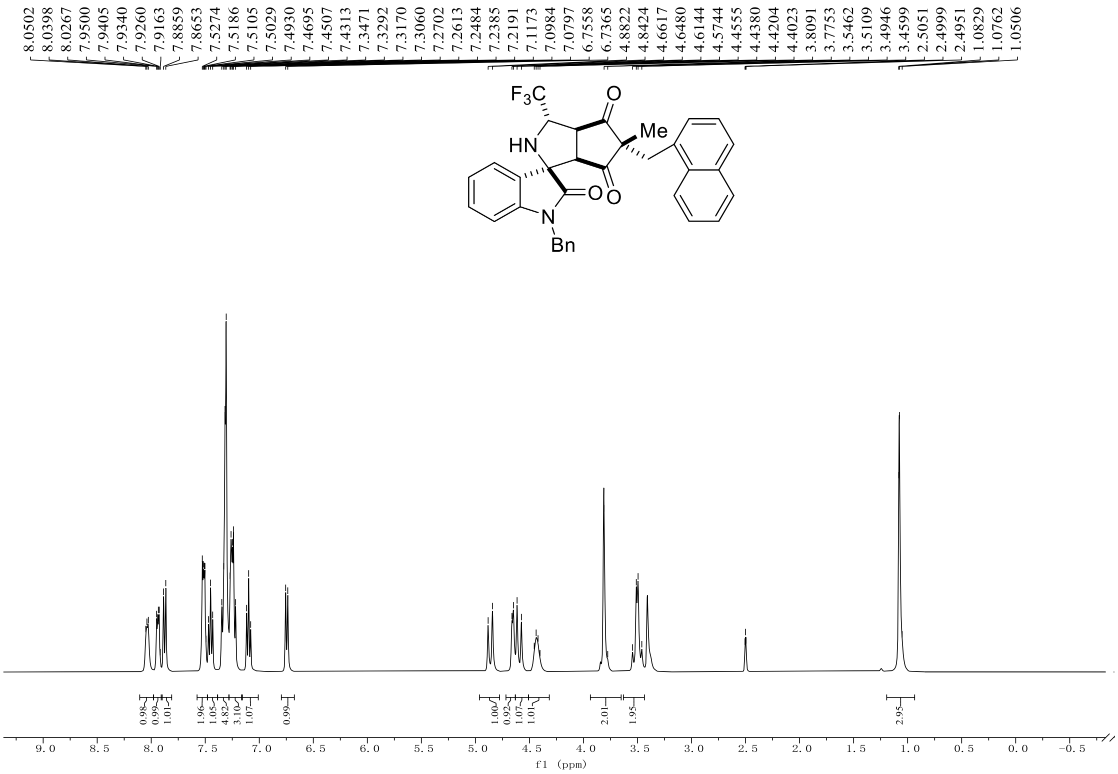

^{13}C{^{1}H} NMR (101 MHz, DMSO-d_6) of **3aj**

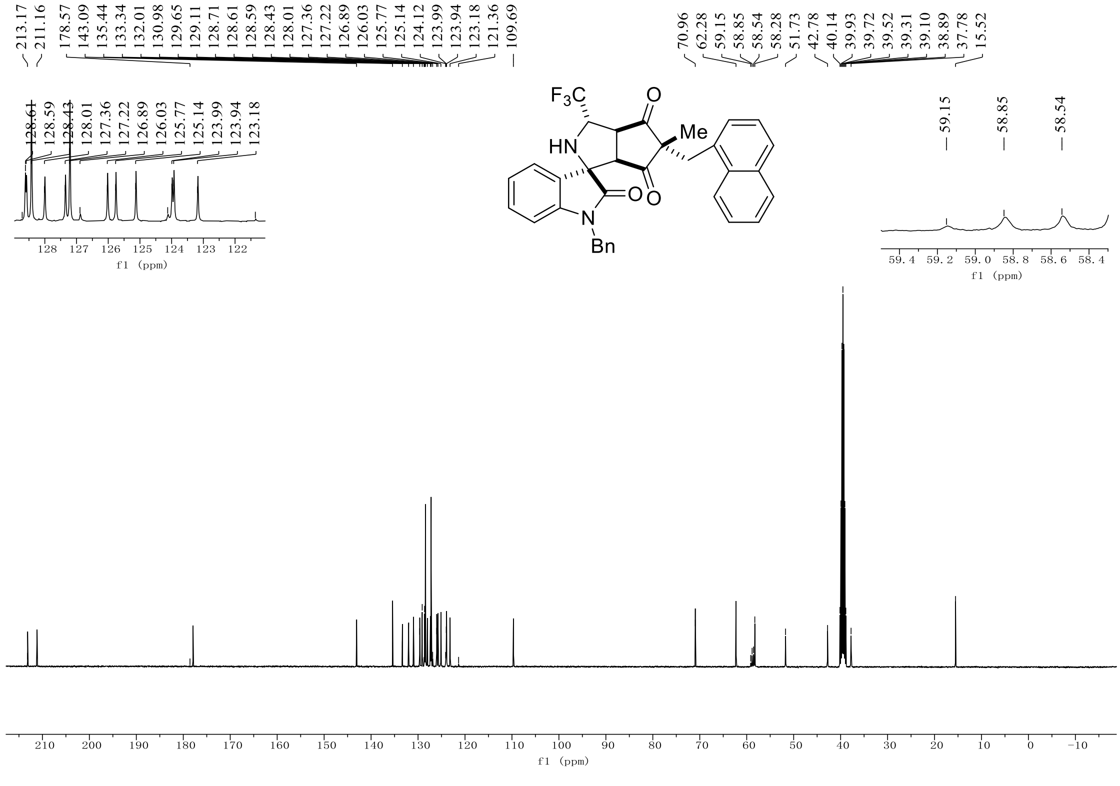

^{1}H NMR (400 MHz, $CDCl_3$) of **3ak**

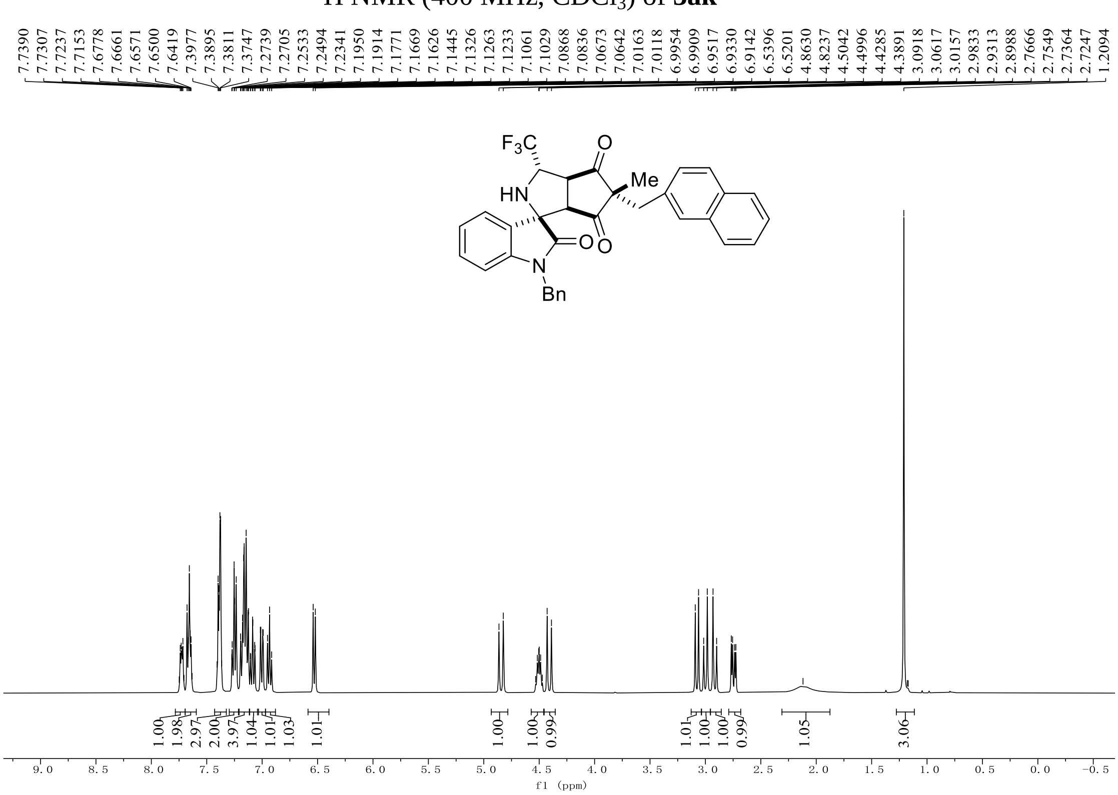

^{13}C{^{1}H} NMR (101 MHz, $CDCl_3$) of **3ak**

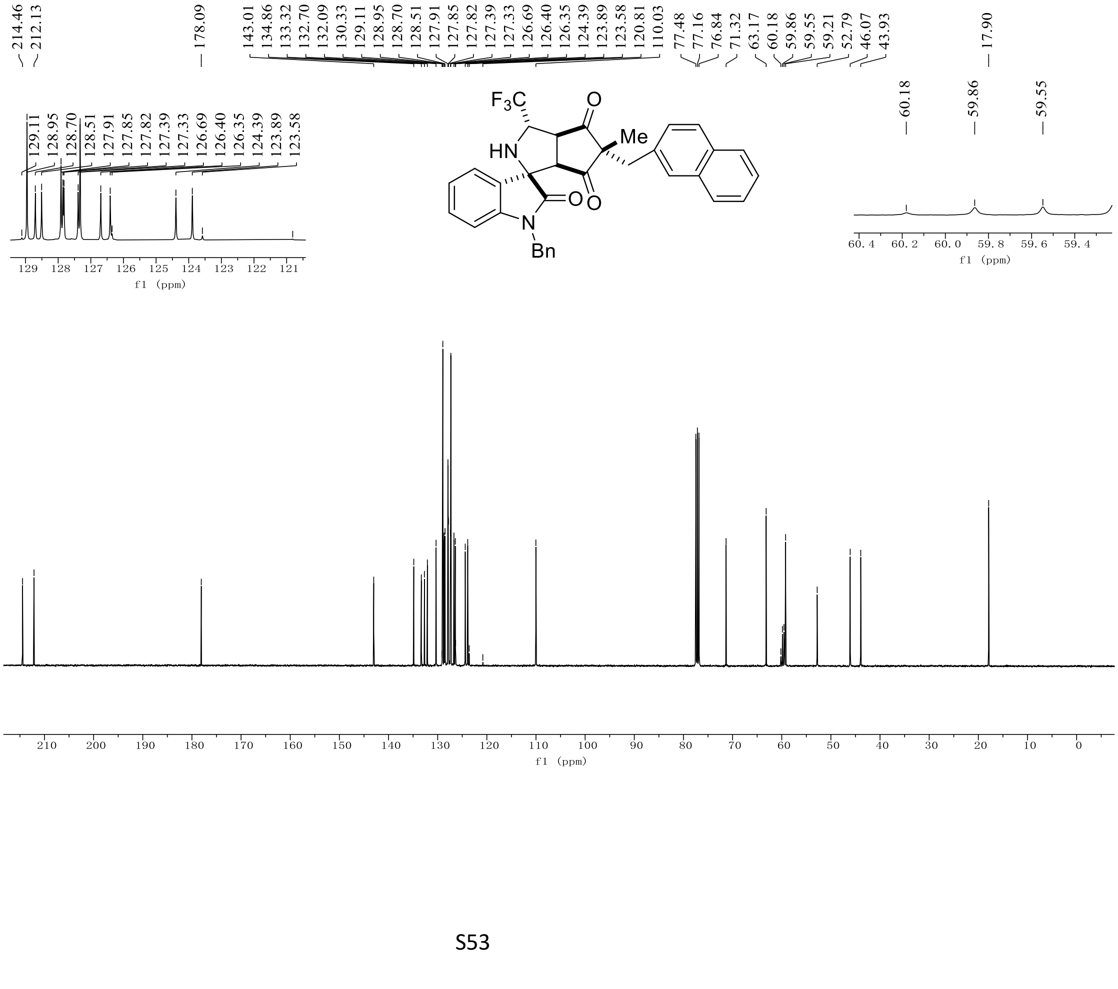

^{1}H NMR (400 MHz, DMSO-d_6) of **3al**

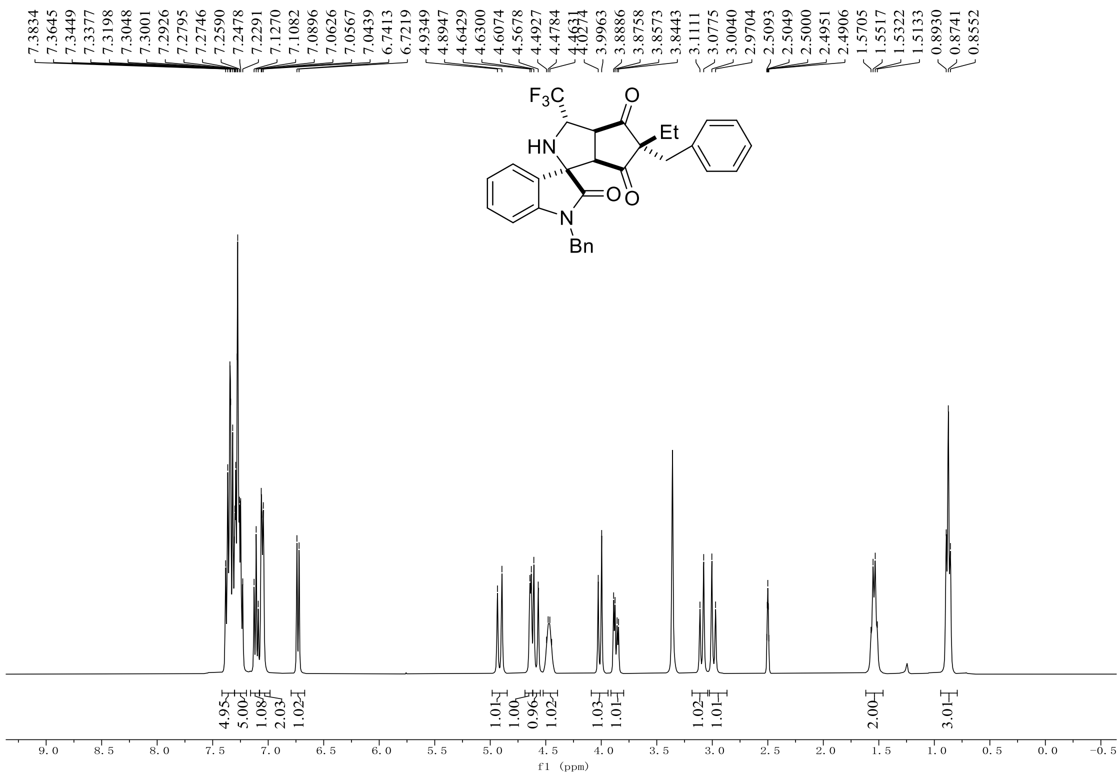

^{13}C{^{1}H} NMR (101 MHz, DMSO-d_6) of **3al**

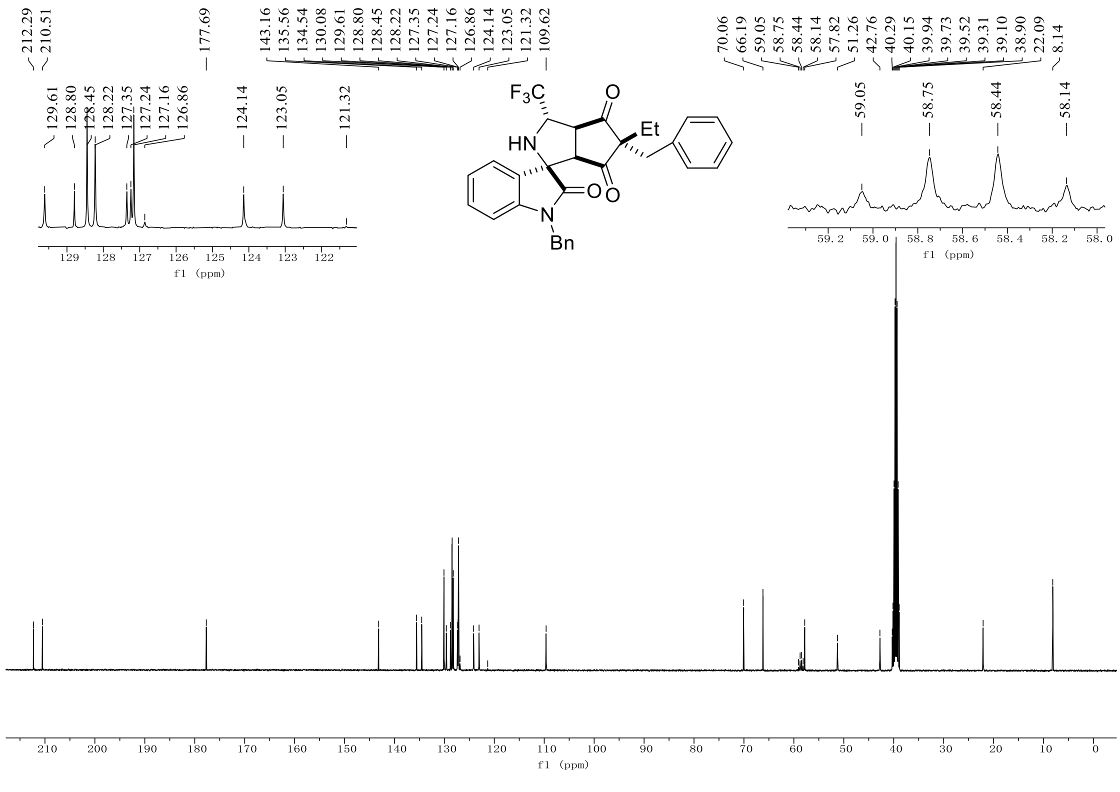

^{1}H NMR (400 MHz, DMSO-d_6) of **3am**

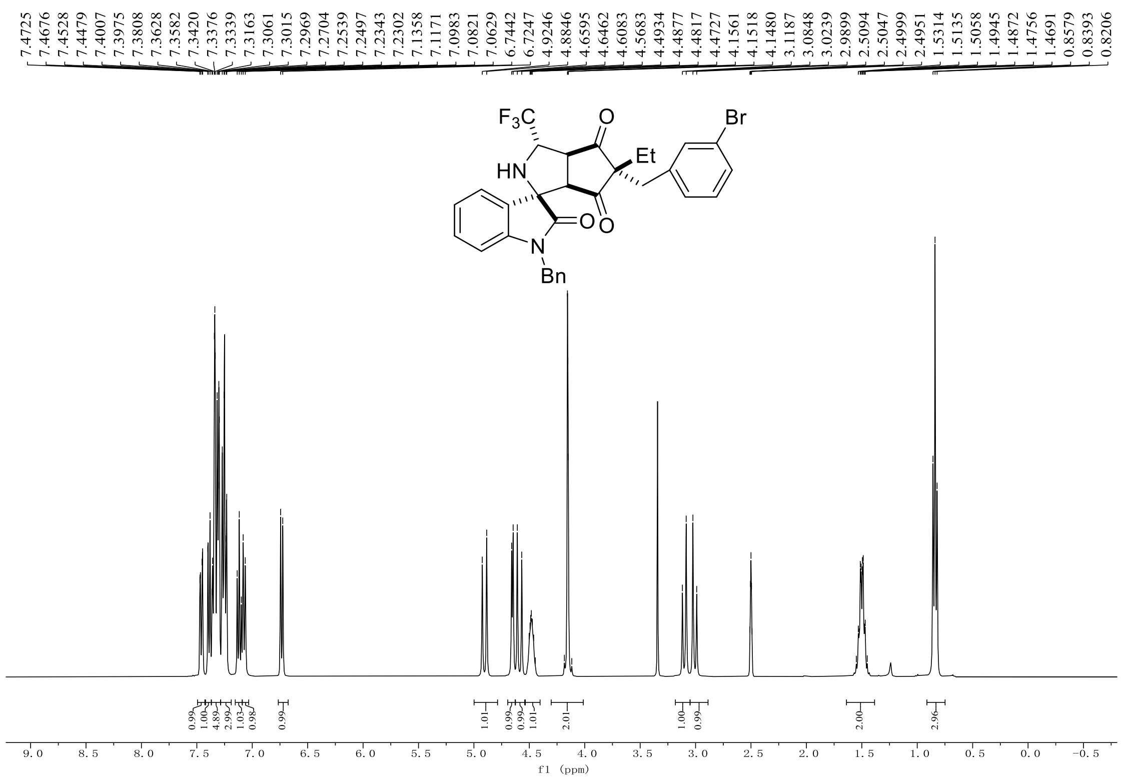

^{13}C{^{1}H} NMR (101 MHz, DMSO-d_6) of **3am**

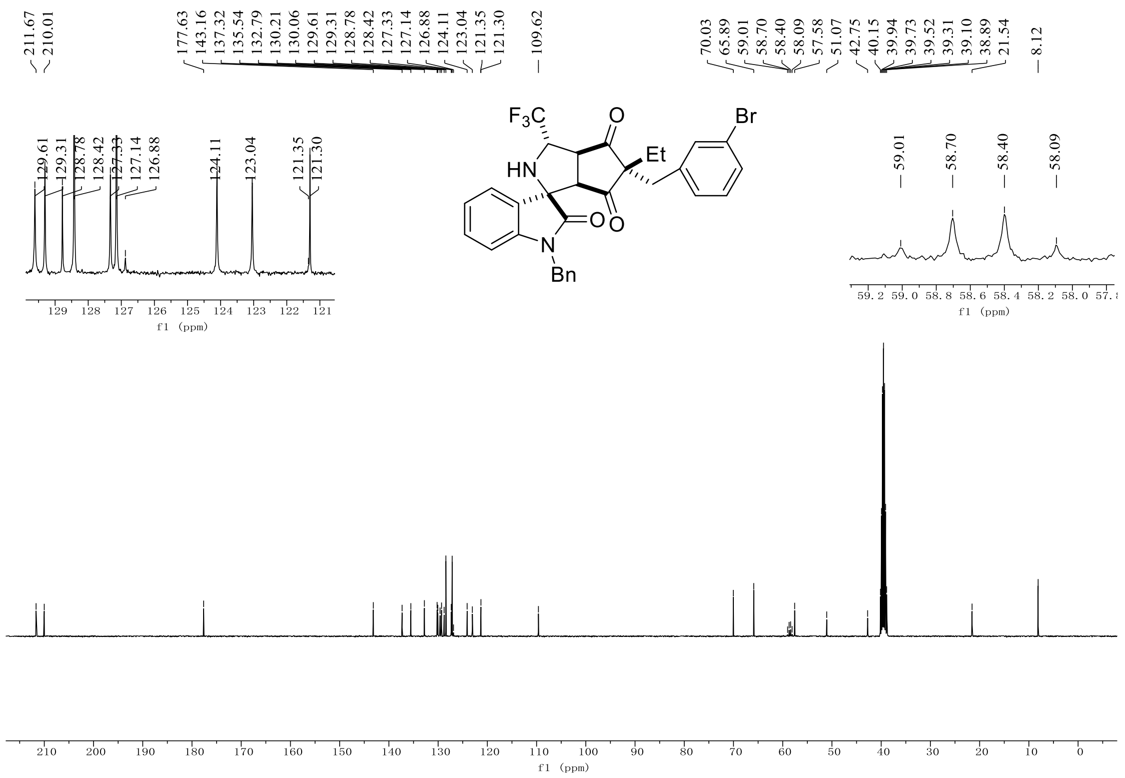

^{1}H NMR (400 MHz, DMSO-d_6) of **3an**

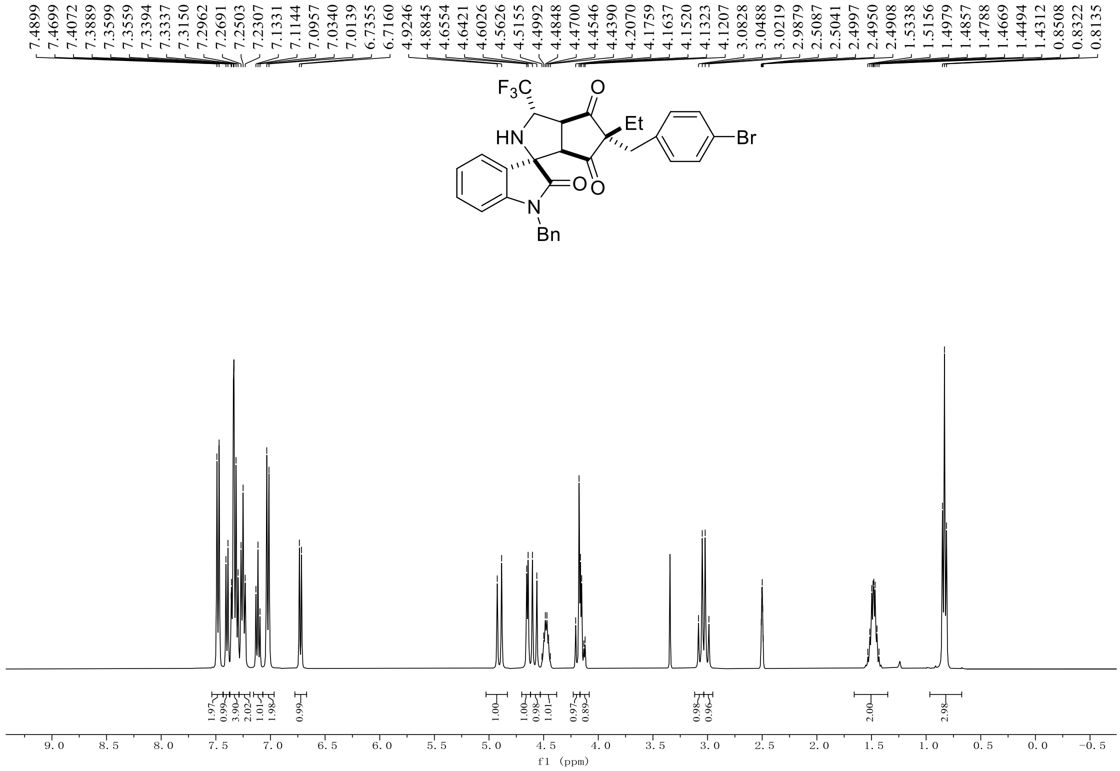

^{13}C{^{1}H} NMR (101 MHz, DMSO-d_6) of **3an**

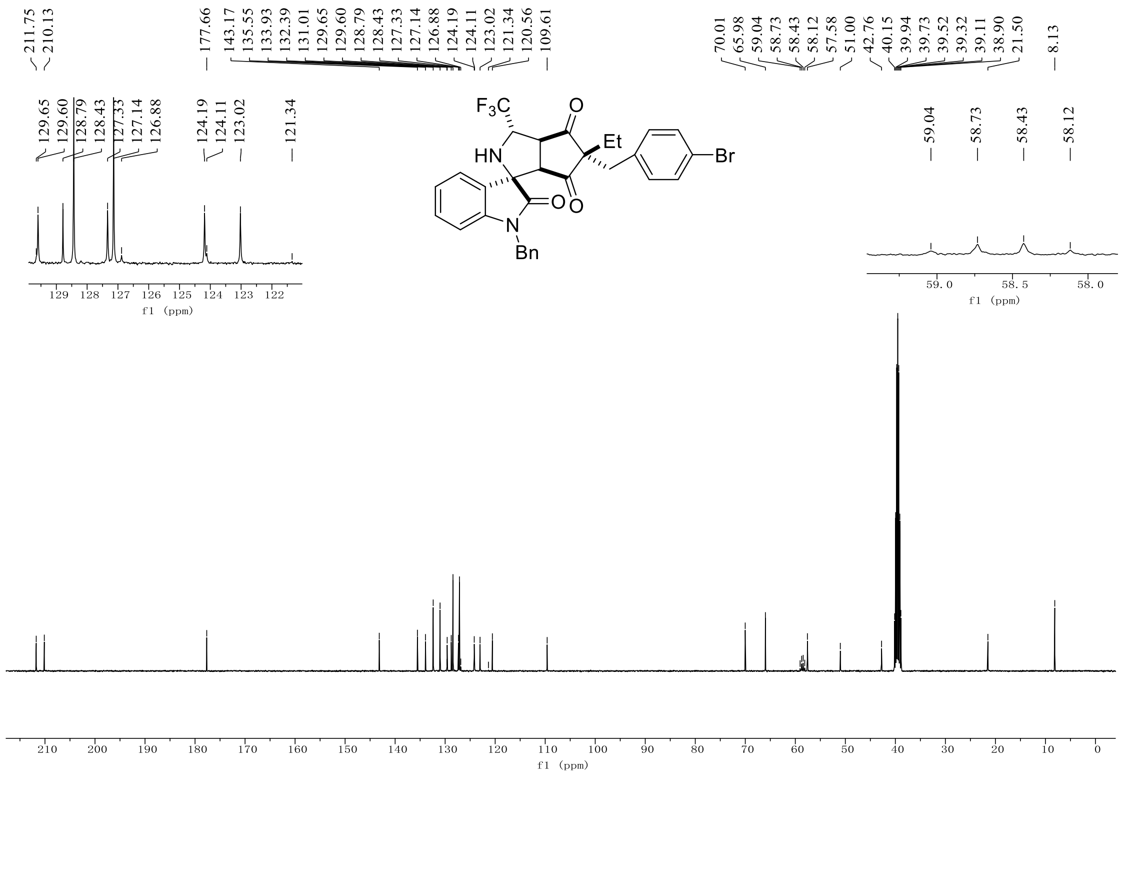

^{1}H NMR (400 MHz, $CDCl_3$) of **5a**

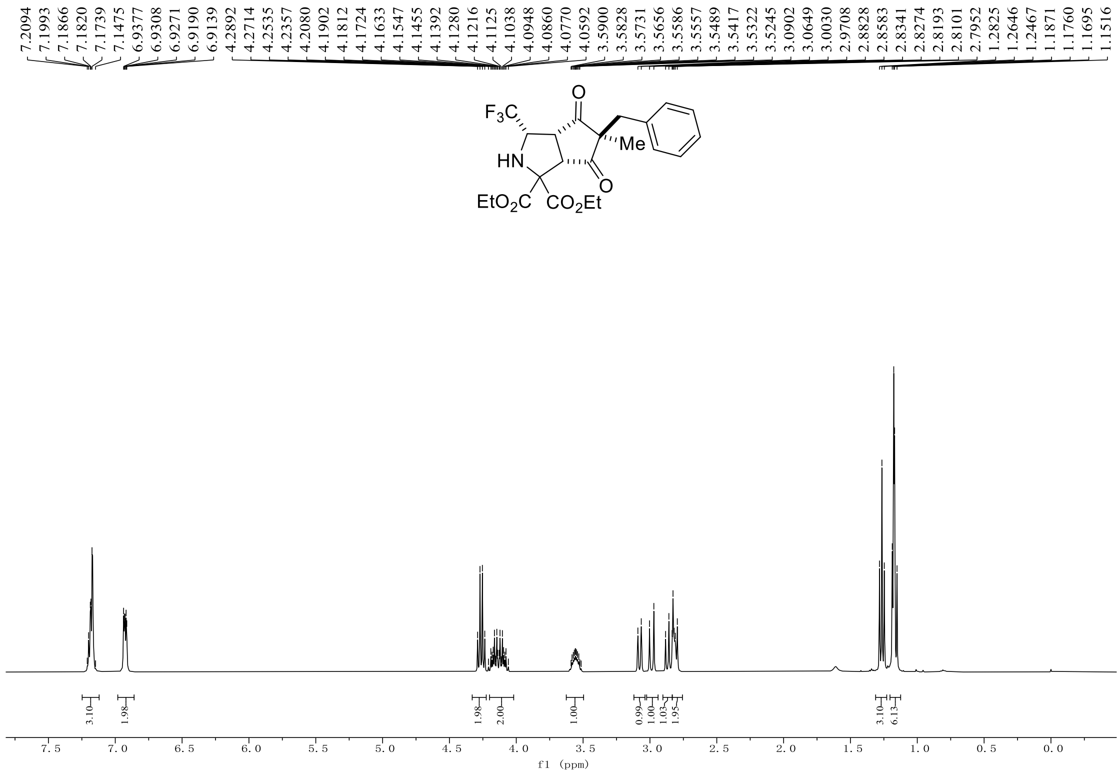

^{13}C{^{1}H} NMR (101 MHz, $CDCl_3$) of **5a**

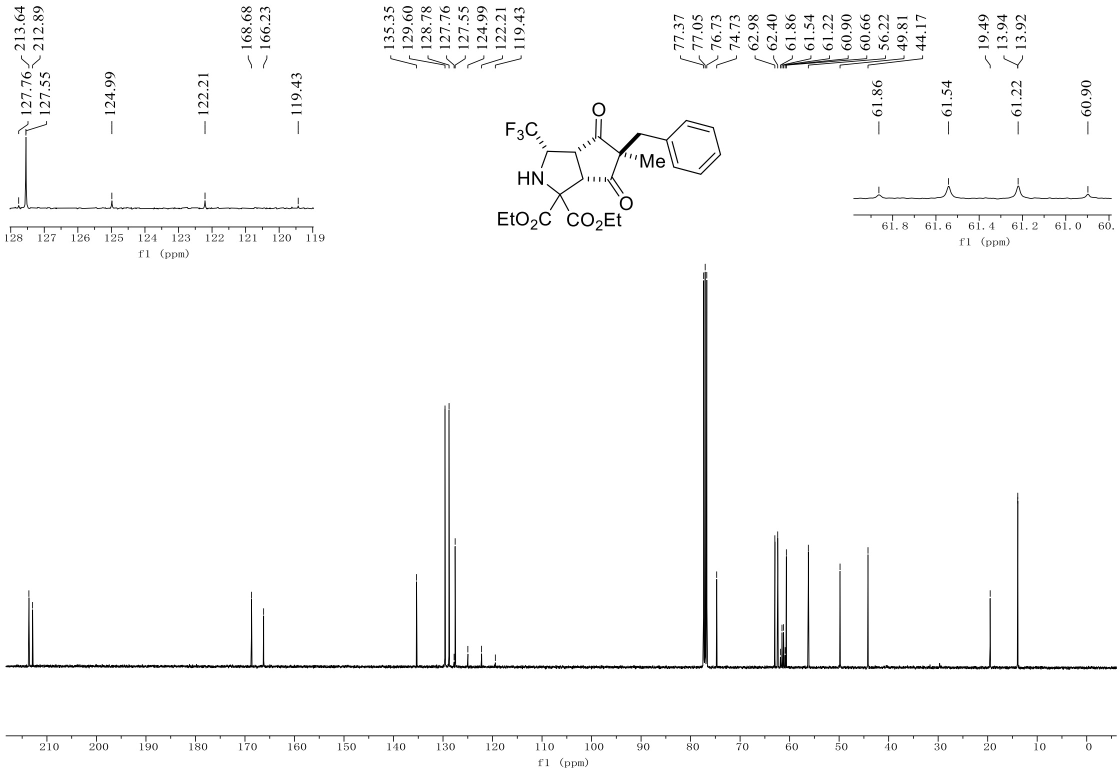

^{1}H NMR (400 MHz, $CDCl_3$) of **5b**

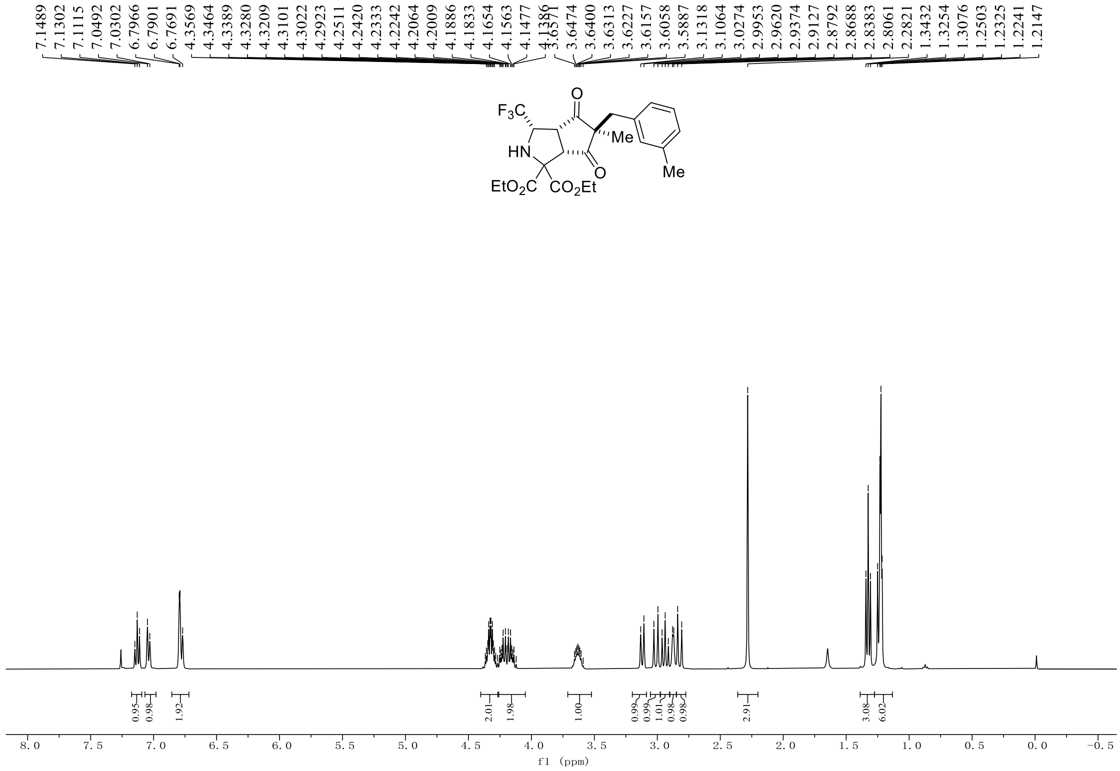

$^{13}C\{^{1}H\}$ NMR (101 MHz, $CDCl_3$) of **5b**

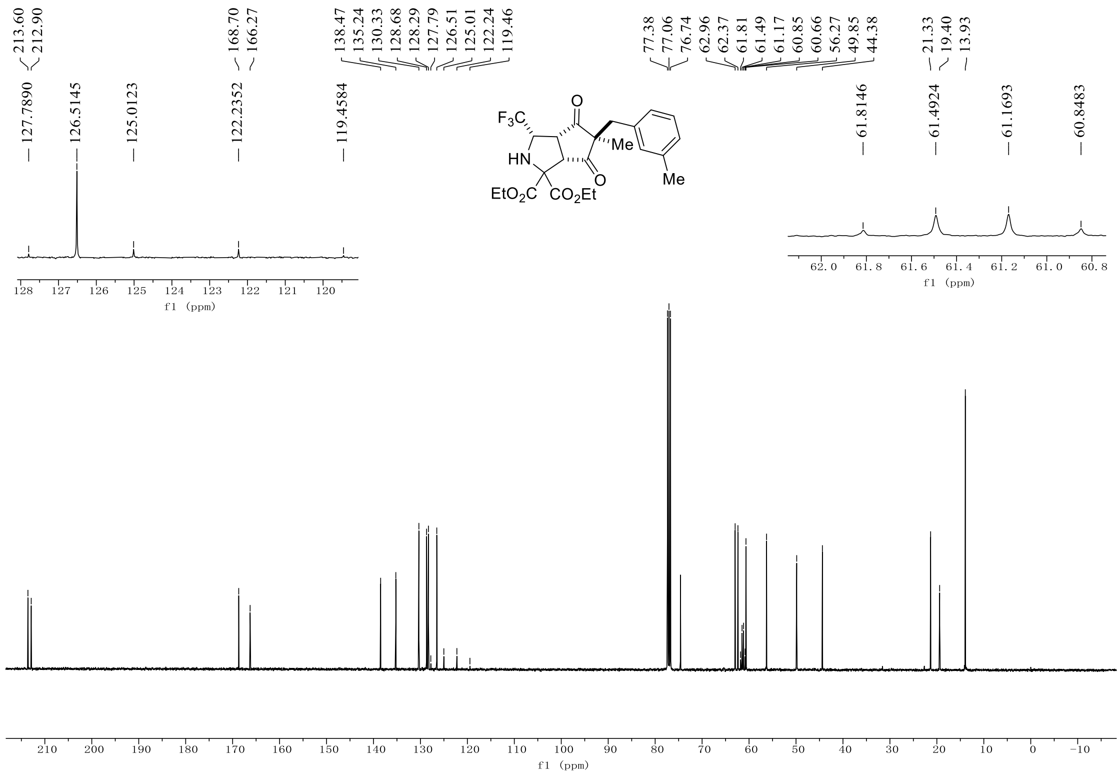

^{1}H NMR (400 MHz, $CDCl_3$) of **5c**

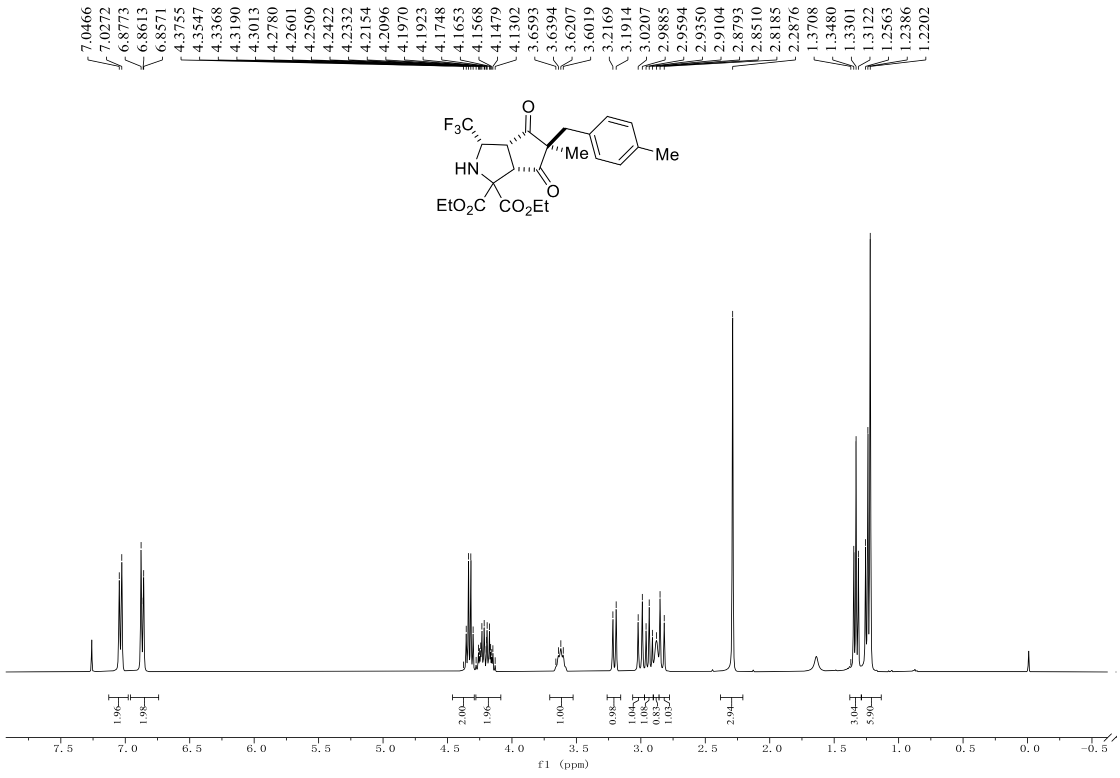

$^{13}C\{^{1}H\}$ NMR (101 MHz, $CDCl_3$) of **5c**

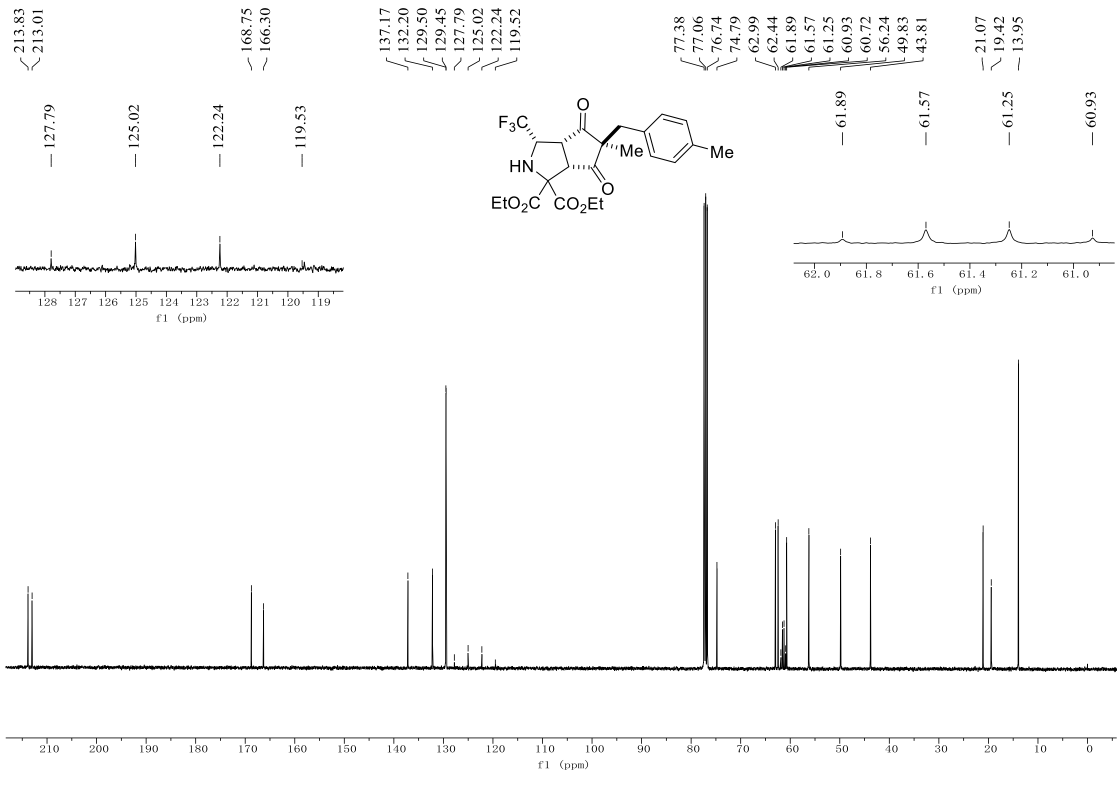

^{1}H NMR (400 MHz, CDCl$_3$) of **5d**

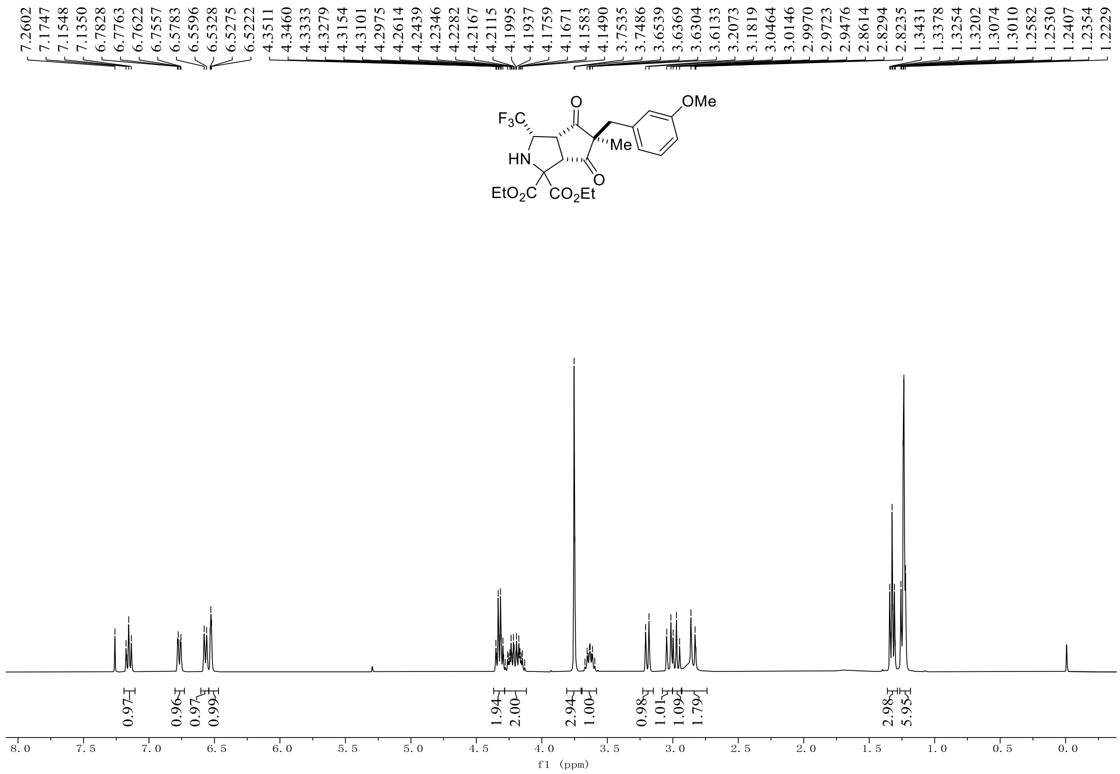

^{13}C{^{1}H} NMR (101 MHz, CDCl$_3$) of **5d**

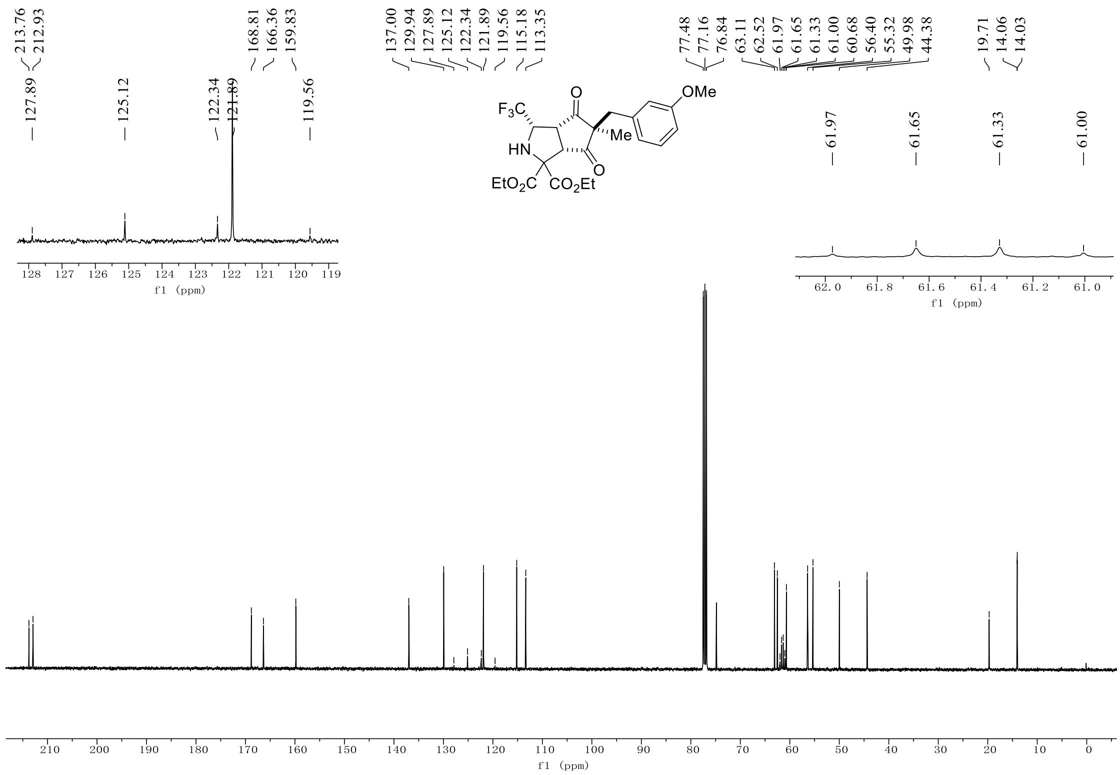

^{1}H NMR (400 MHz, CDCl$_3$) of **5e**

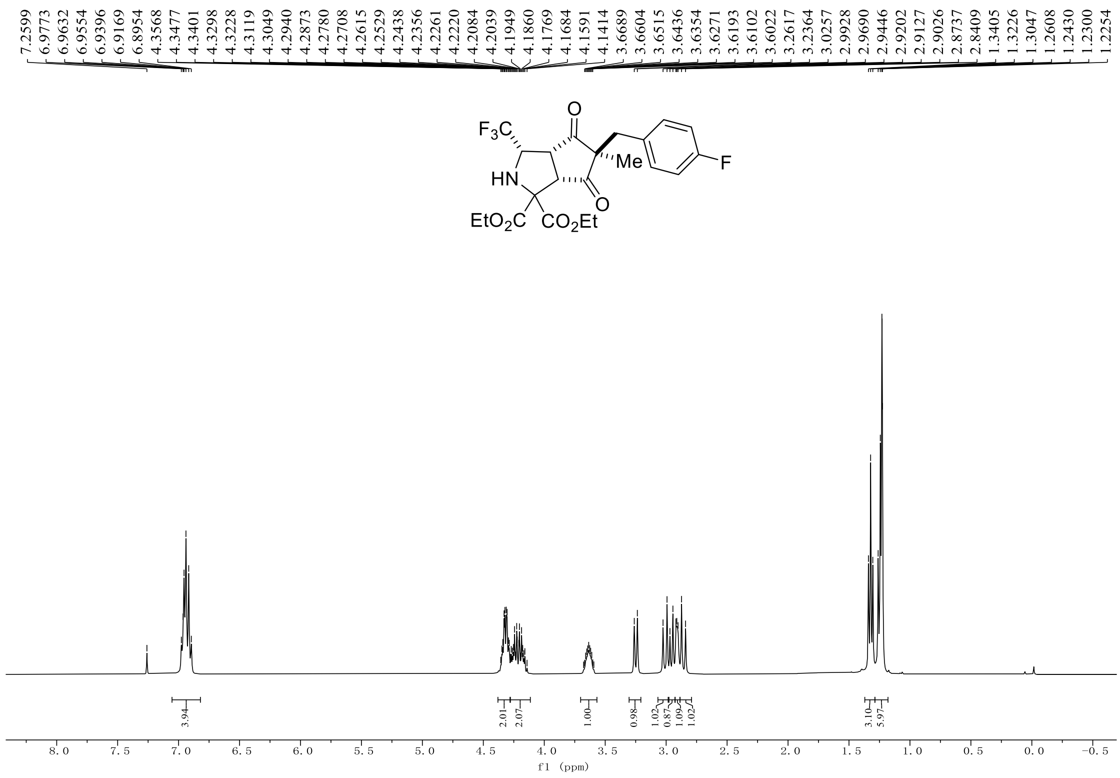

$^{13}C\{^{1}H\}$ NMR (101 MHz, $CDCl_3$) of **5e**

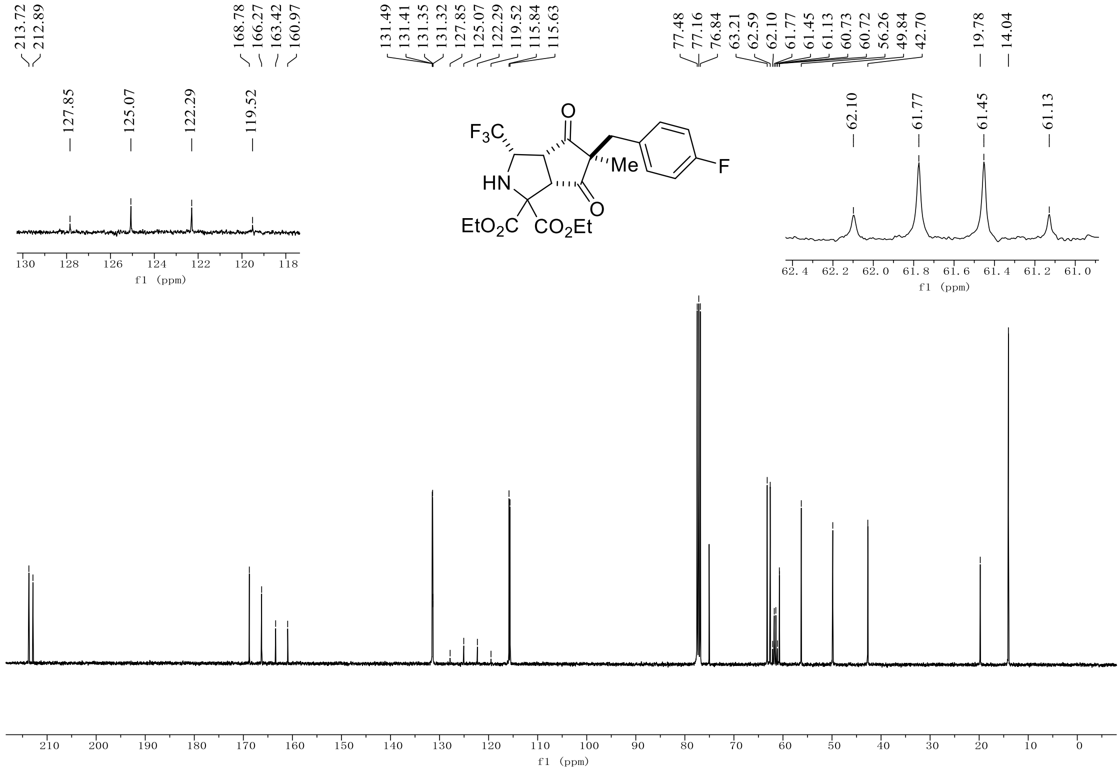

^{1}H NMR (400 MHz, $CDCl_3$) of **5f**

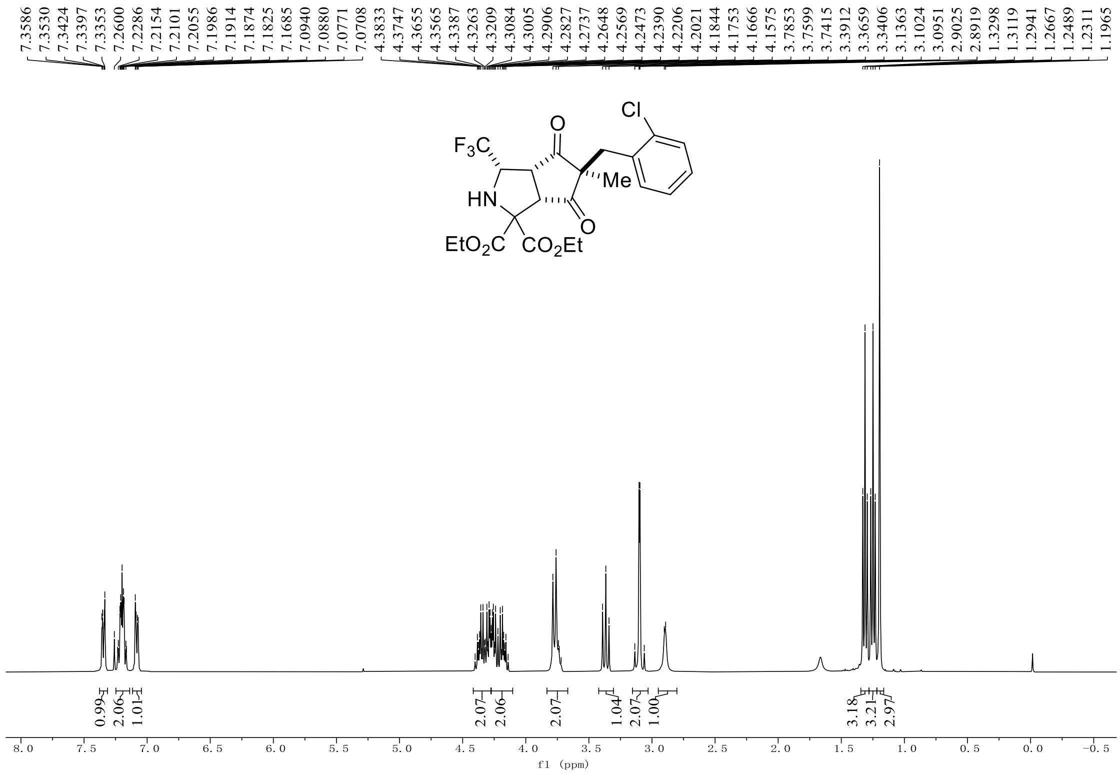

^{13}C{^{1}H} NMR (101 MHz, $CDCl_3$) of **5f**

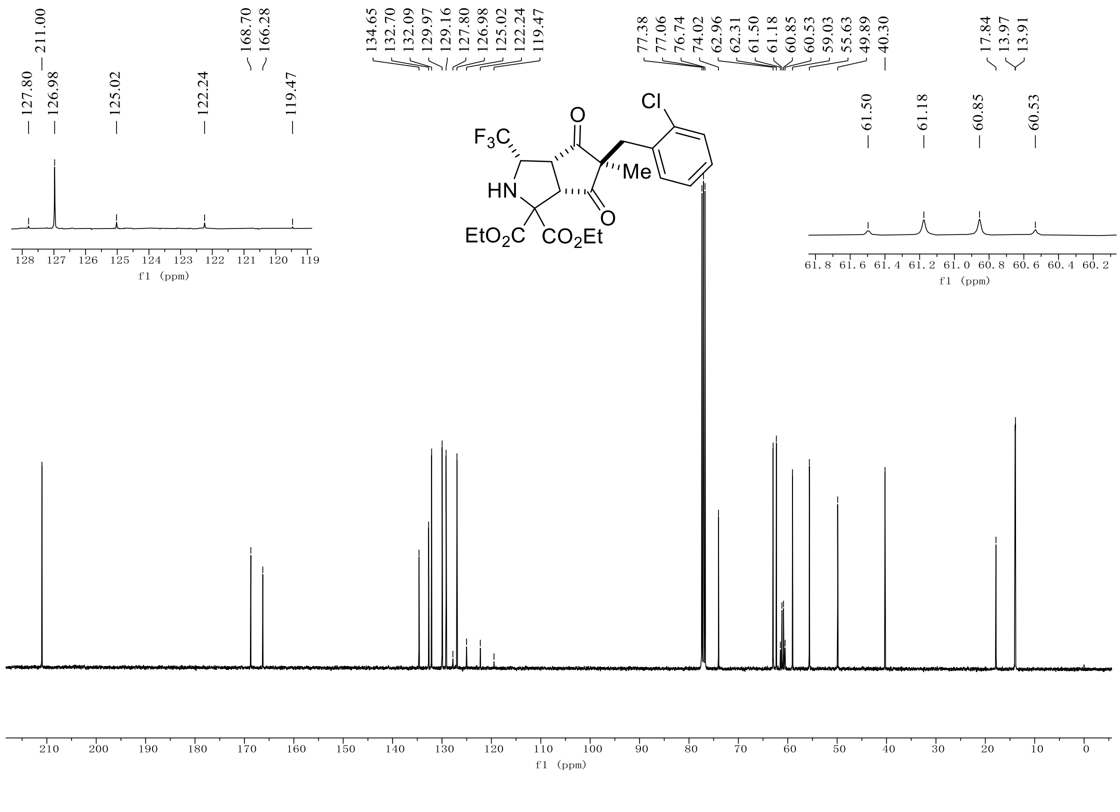

^{1}H NMR (400 MHz, $CDCl_3$) of **5g**

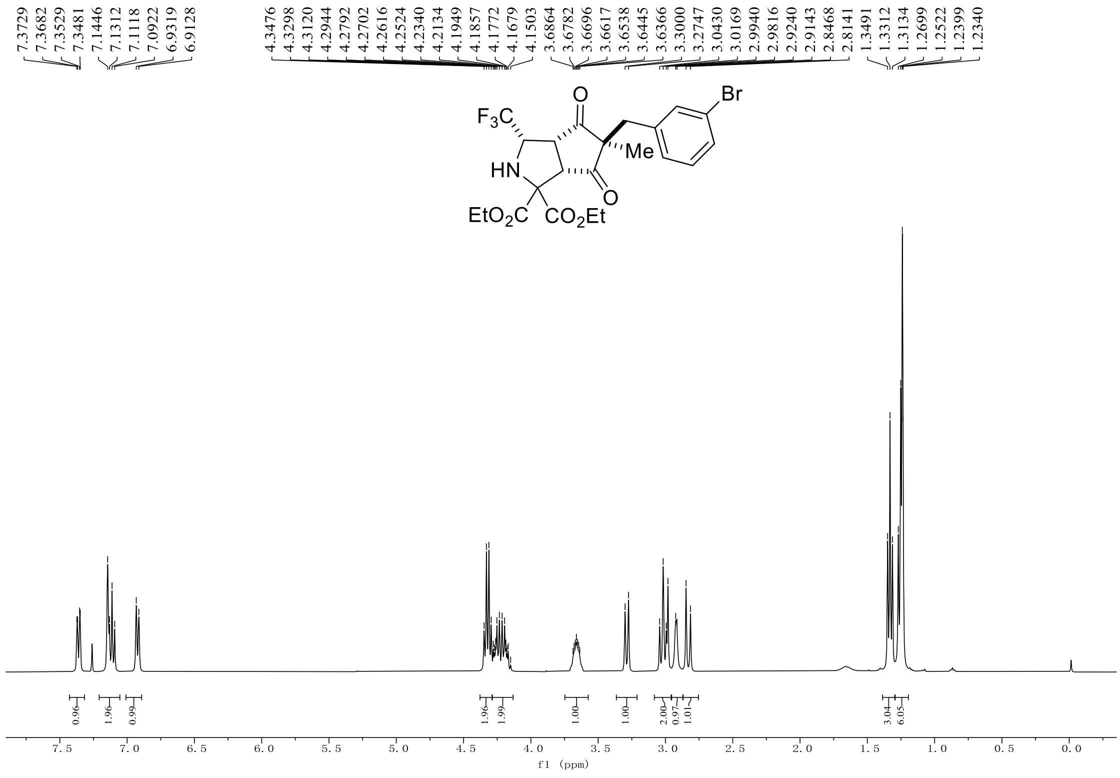

^{13}C{^{1}H} NMR (101 MHz, $CDCl_3$) of **5g**

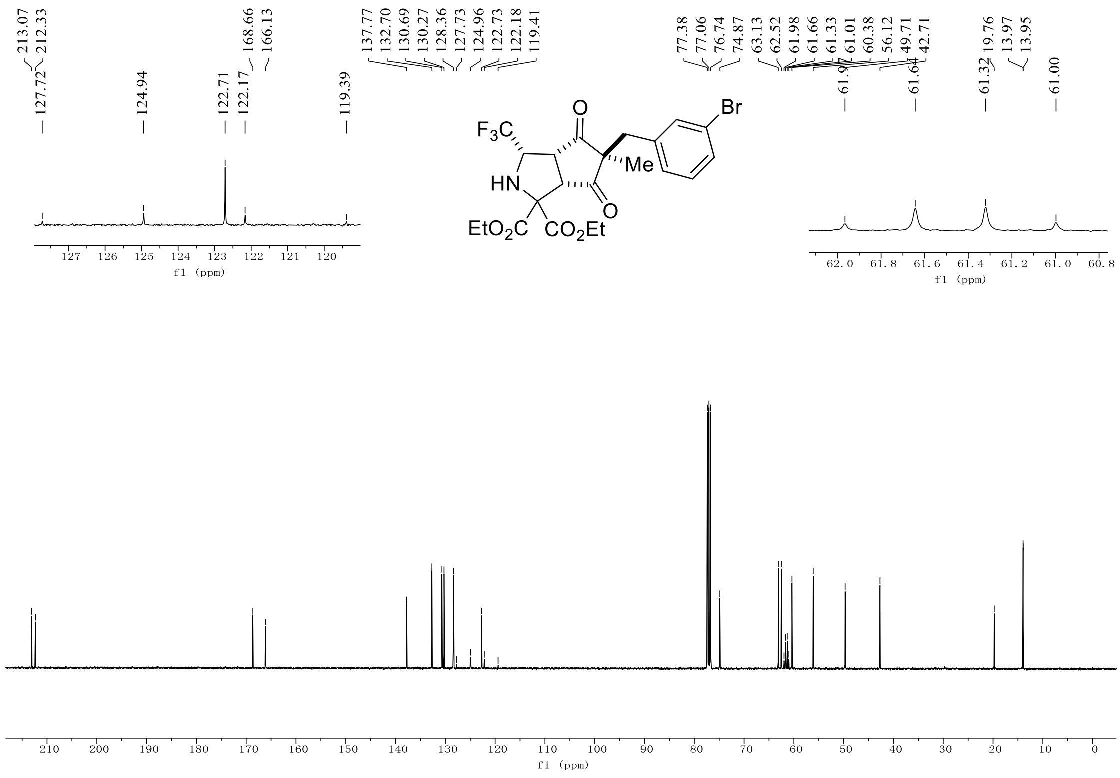

^{1}H NMR (400 MHz, $CDCl_3$) of **5h**

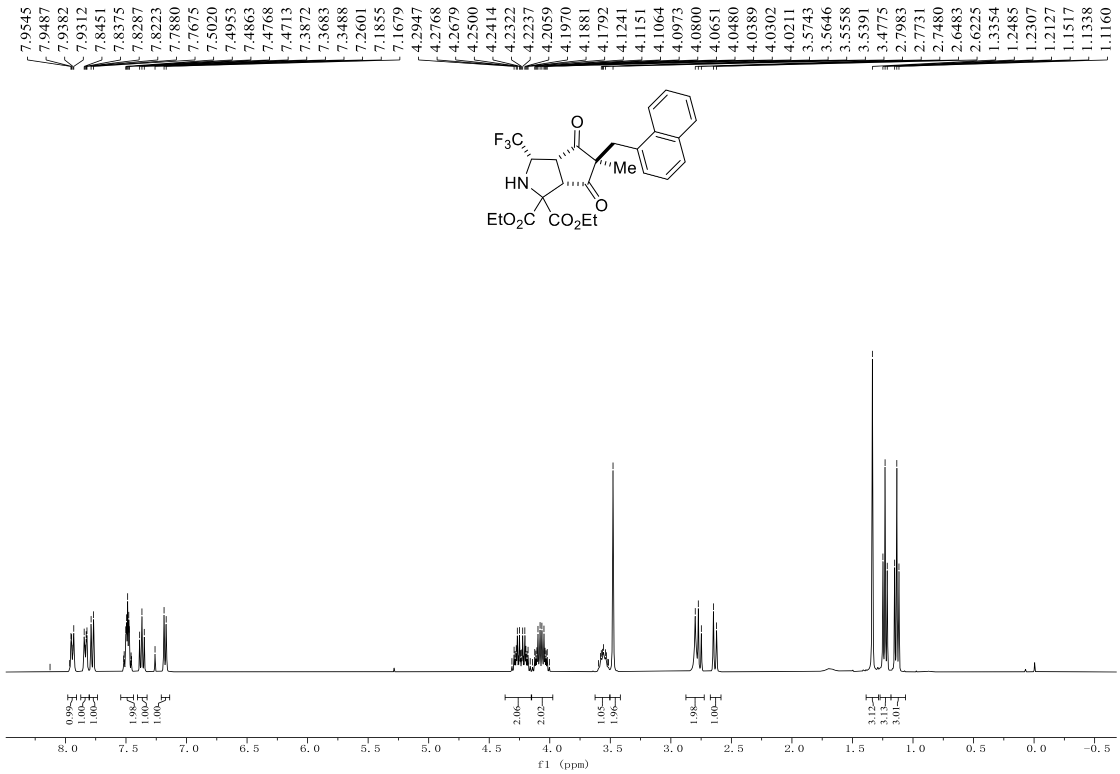

^{13}C{^{1}H} NMR (101 MHz, $CDCl_3$) of **5h**

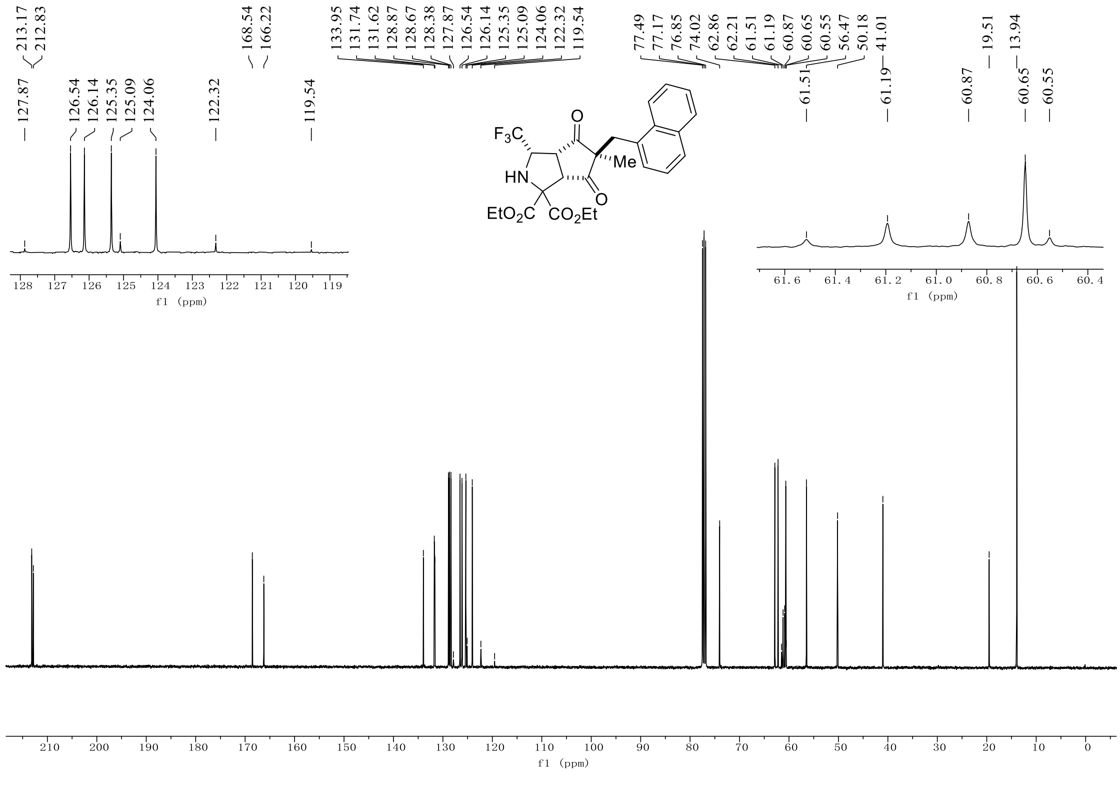

^{1}H NMR (400 MHz, $CDCl_3$) of **5i**

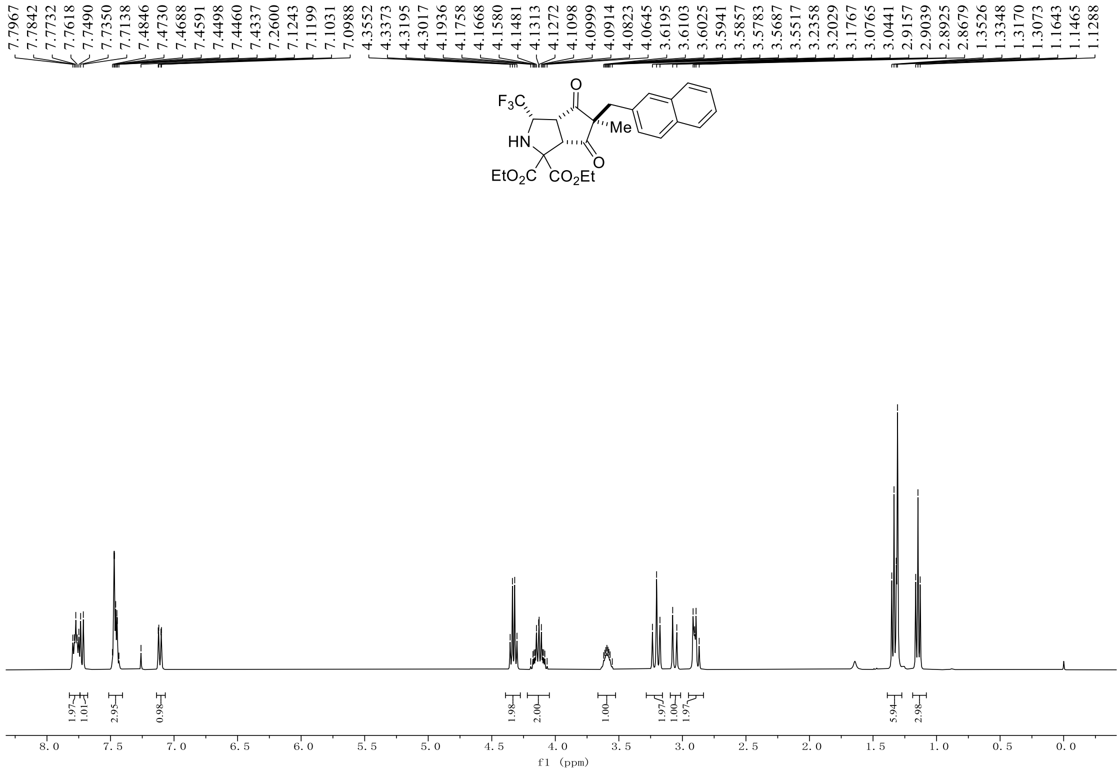

$^{13}C\{^{1}H\}$ NMR (101 MHz, $CDCl_3$) of **5i**

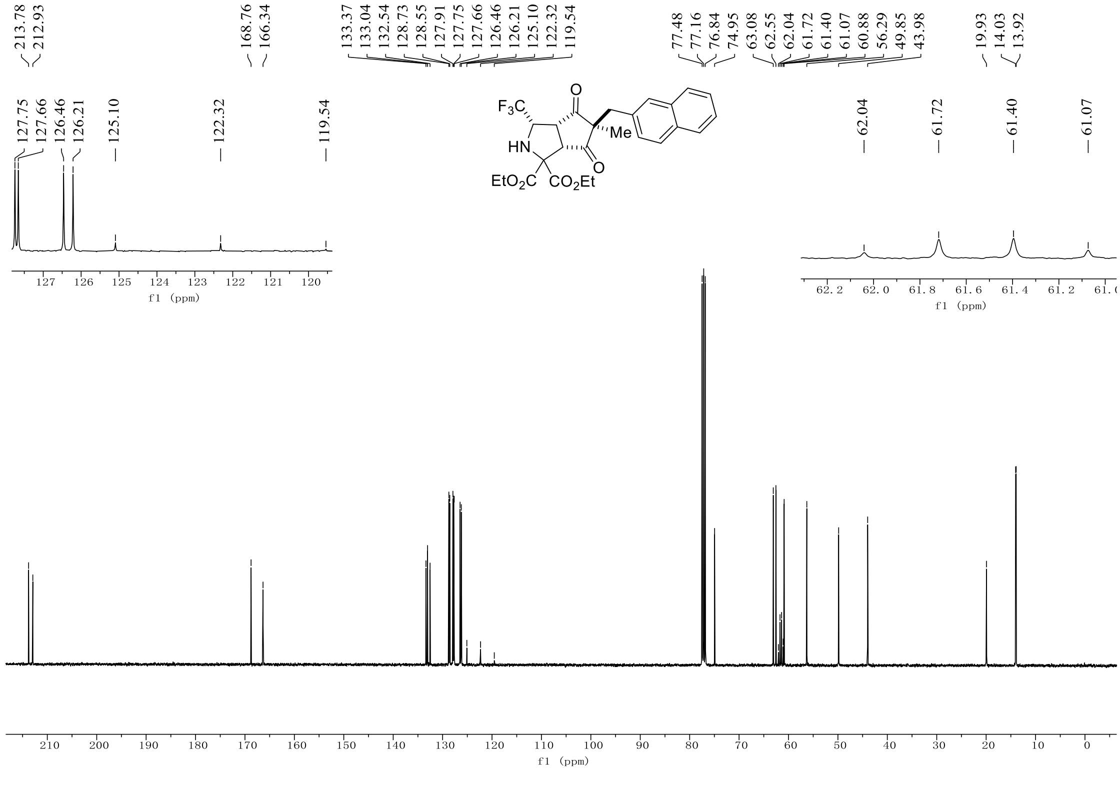

^{1}H NMR (300 MHz, DMSO-d_6) of **6**

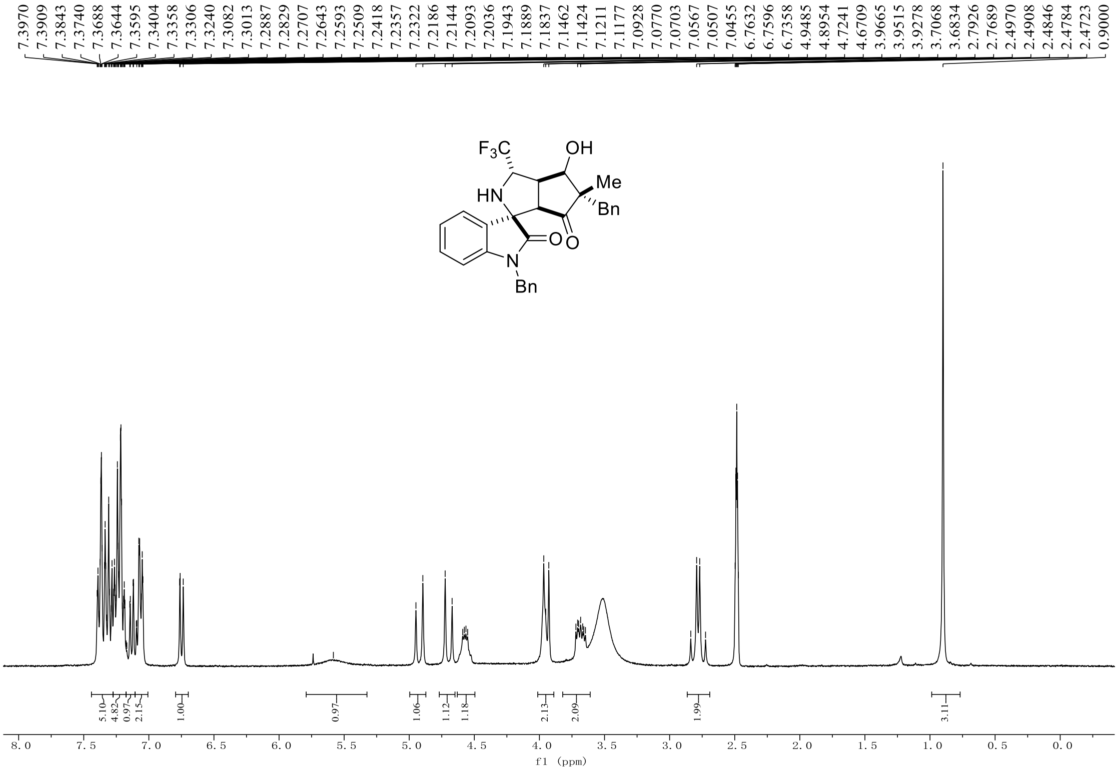

$^{13}C\{^{1}H\}$ NMR (75 MHz, $CDCl_3$) of **6**

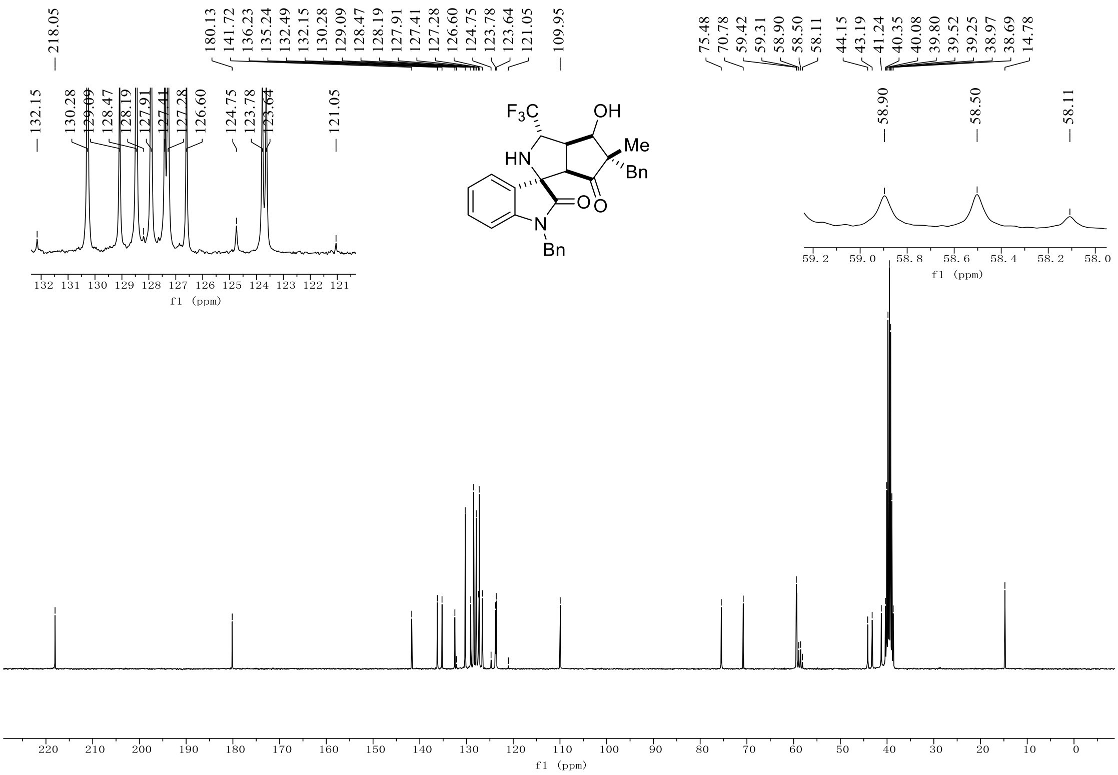

^1H-^1H COSY spectra for stereochemistry of product **6**

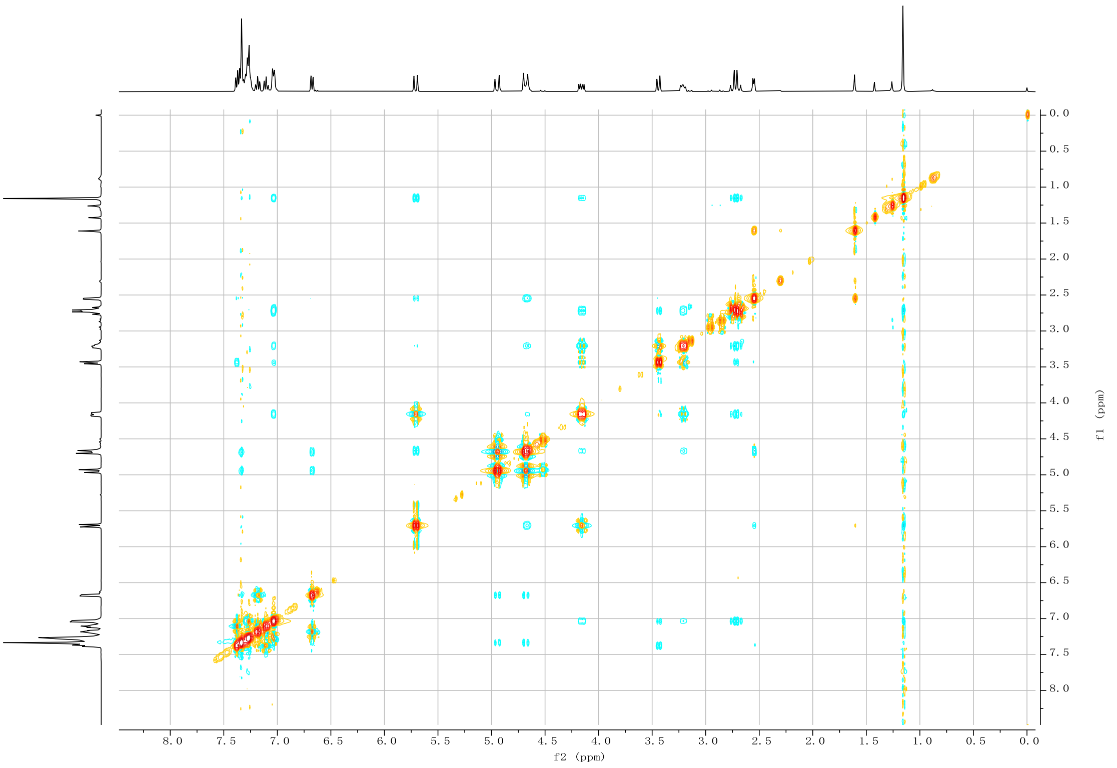

Attribution of hydrogen in compound **6**

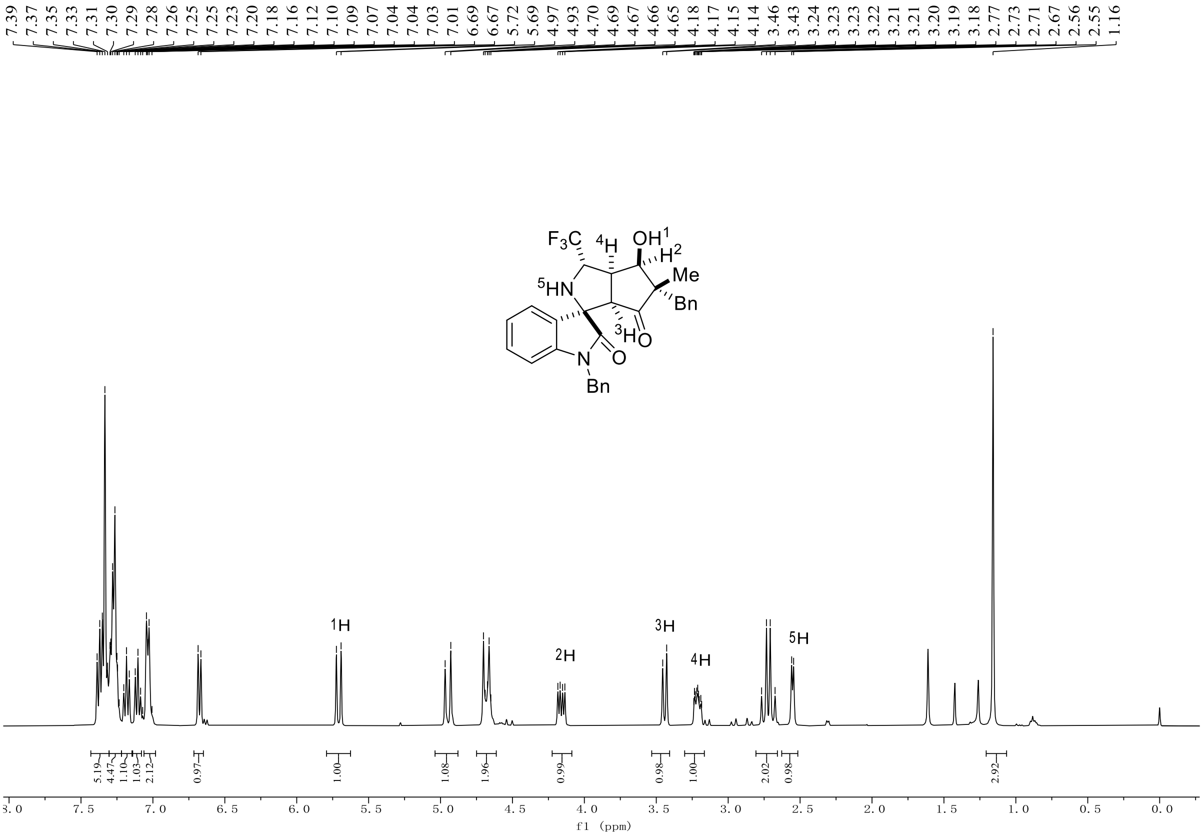

^{1}H NMR (300 MHz, $CDCl_3$) of **7**

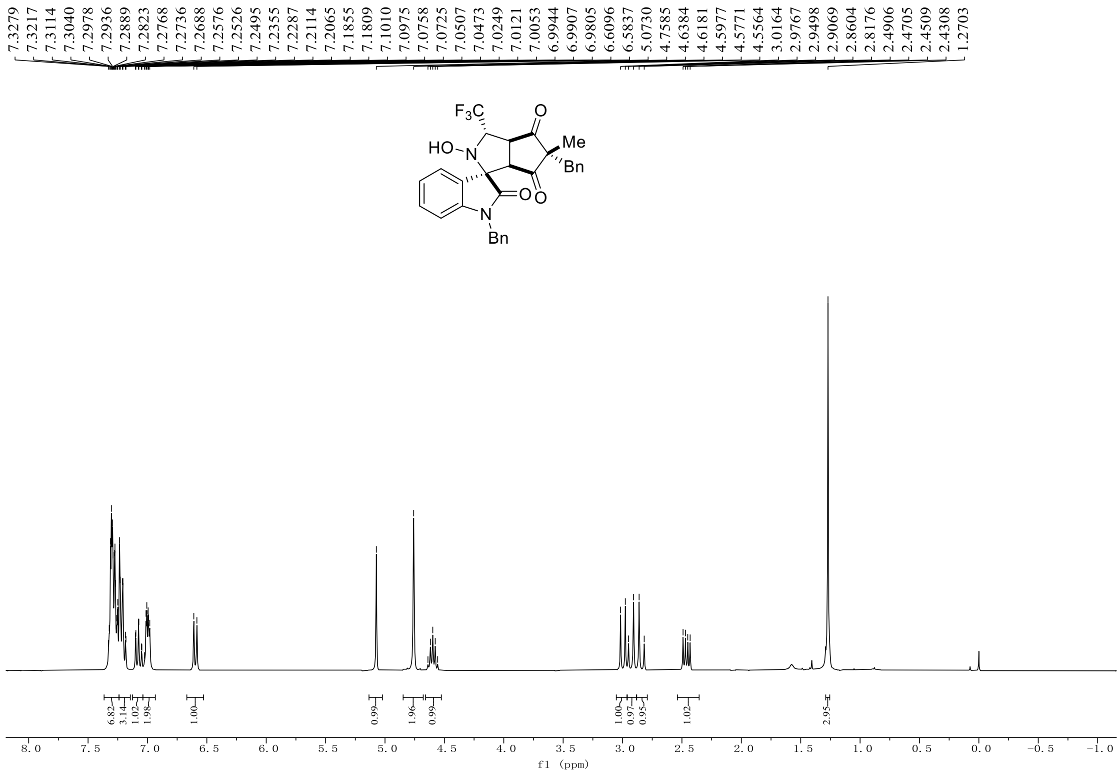

^{13}C{^{1}H} NMR (75 MHz, $CDCl_3$) of **7**

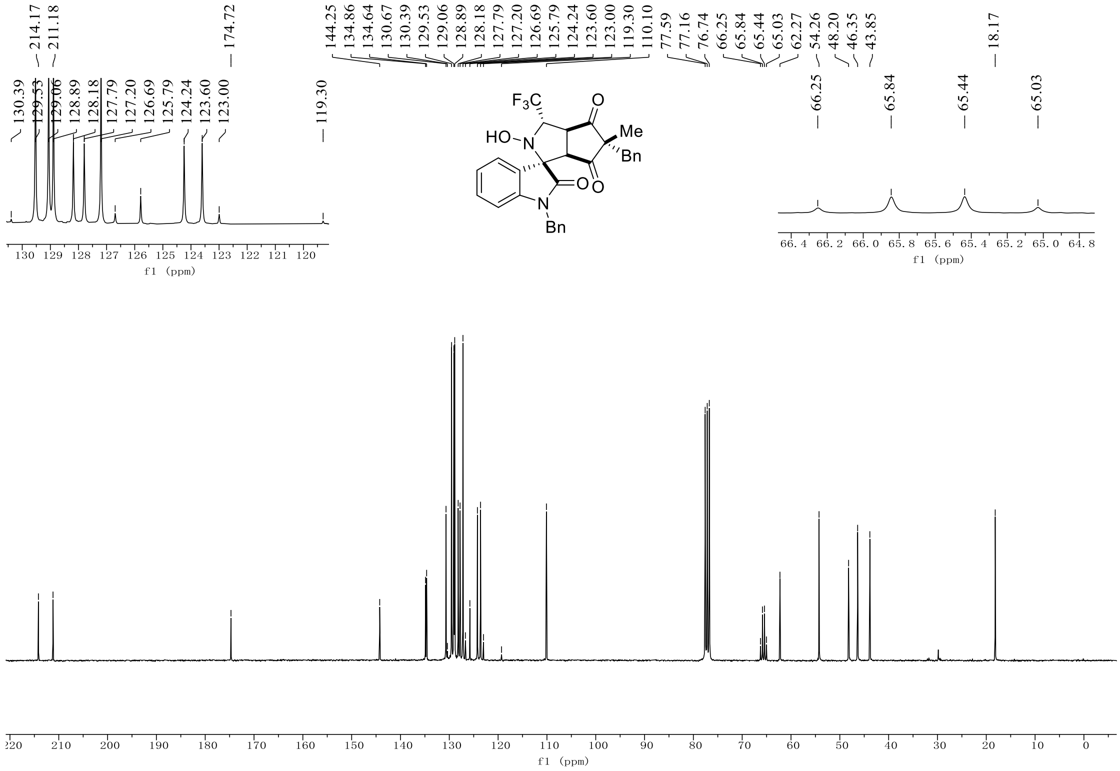

^{1}H NMR (400 MHz, $CDCl_3$) of **8**

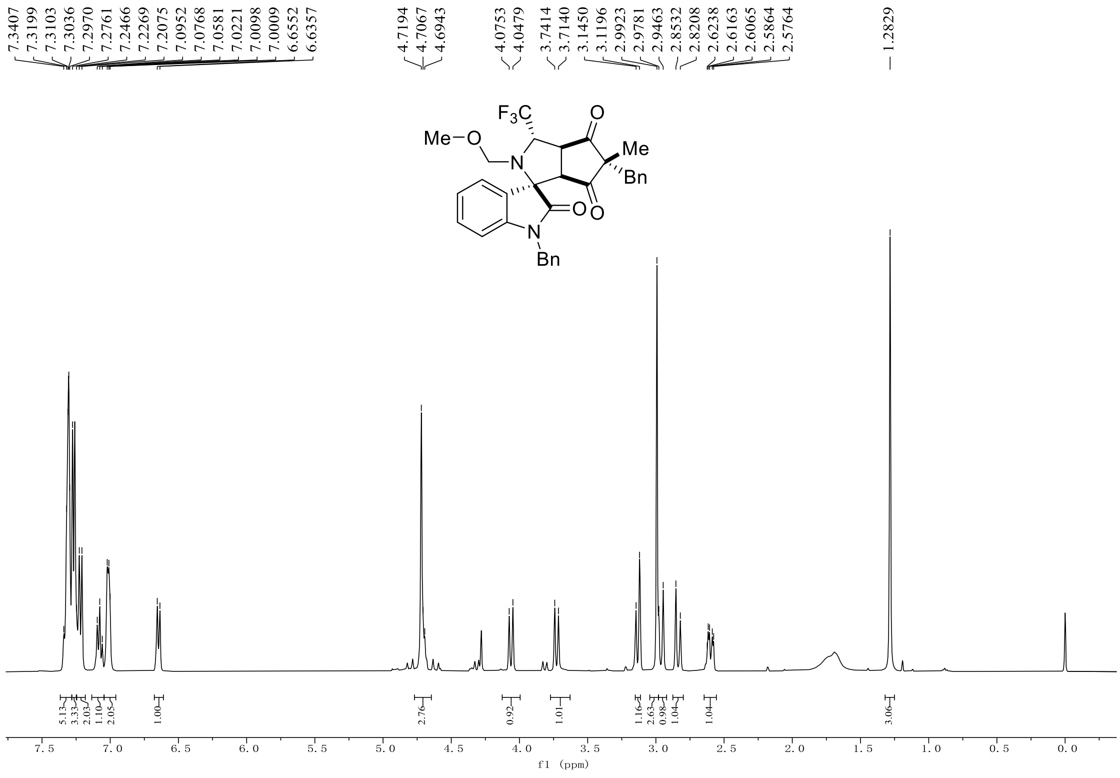

^{13}C{^{1}H} NMR (101 MHz, $CDCl_3$) of **8**

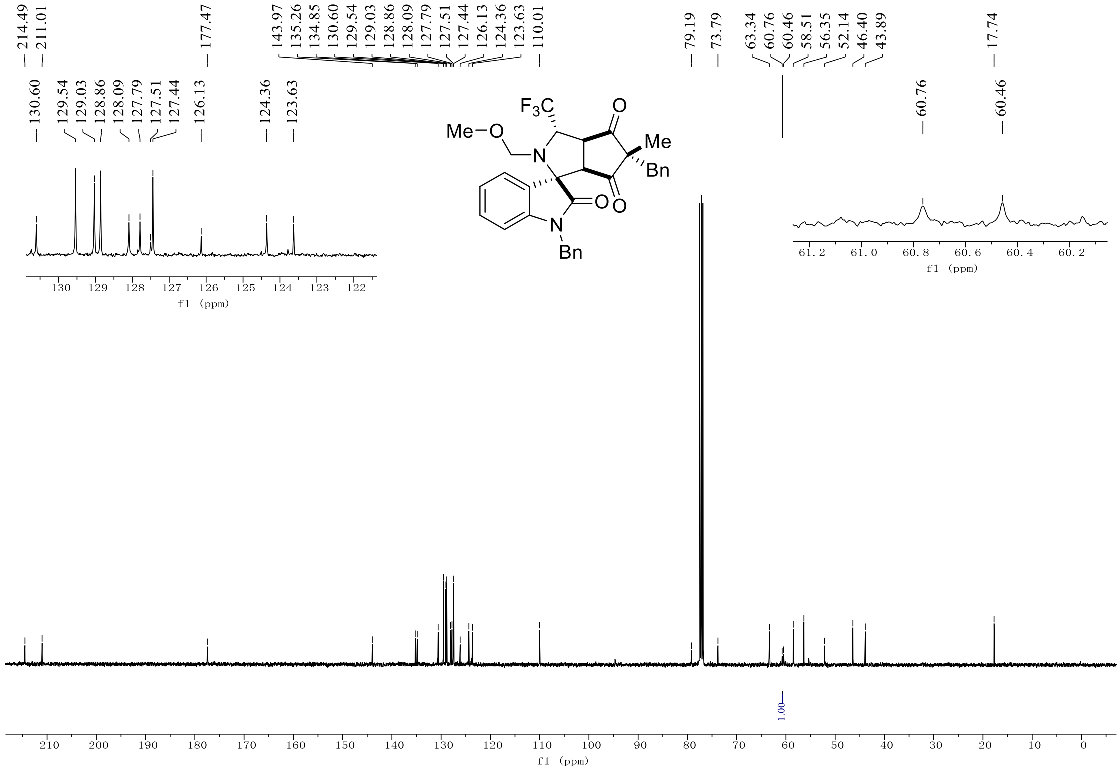

^{1}H NMR (400 MHz, DMSO-d_6) of **9**

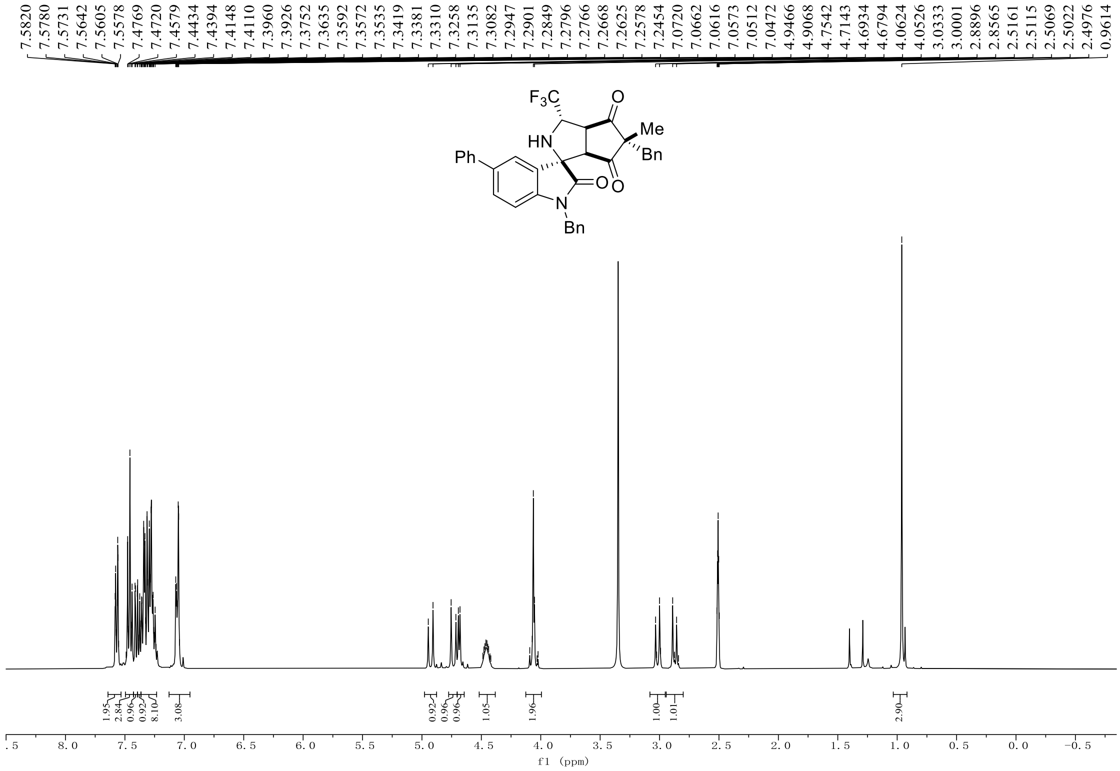

^{13}C{^{1}H} NMR (101 MHz, DMSO-d_6) of **9**

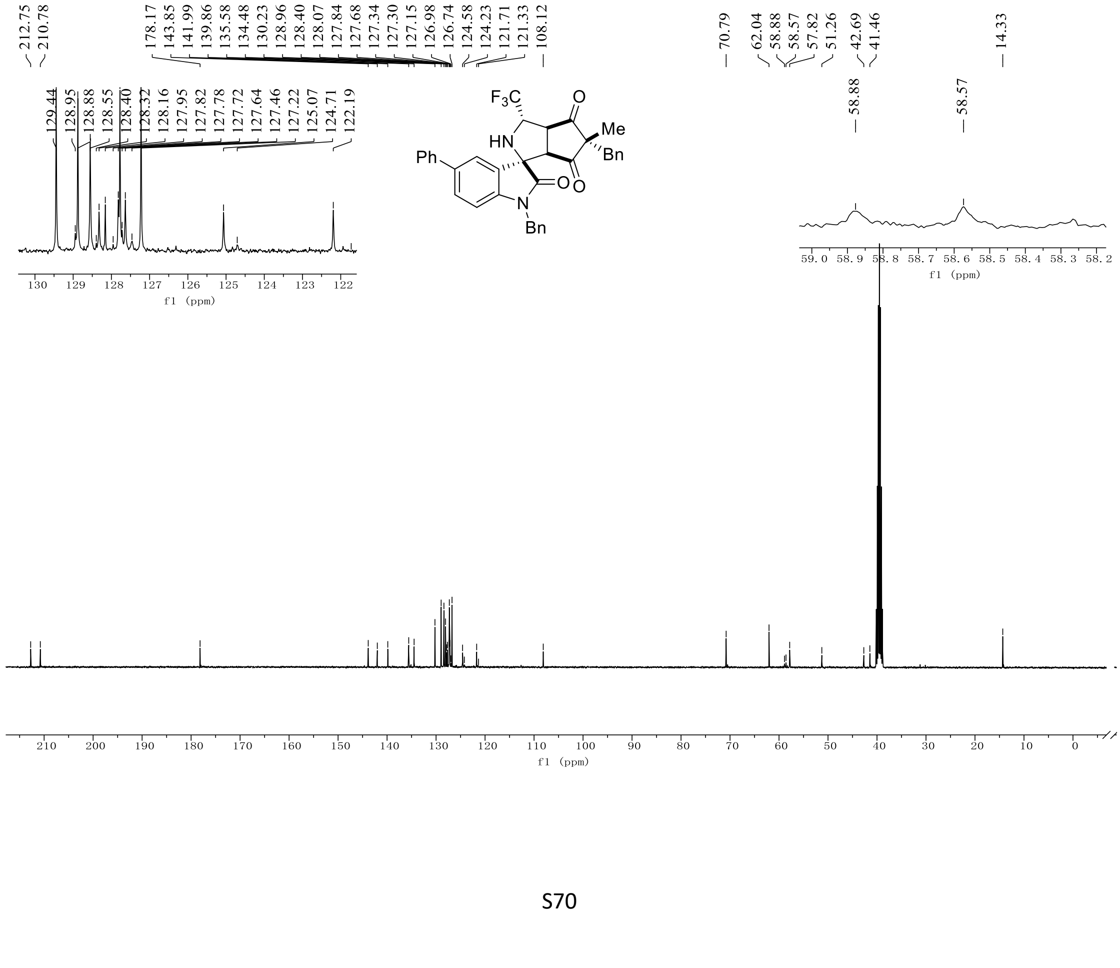

11. MS spectra for compounds 3, 5, 6, 7, 8, 9.

MS spectra of **3aa**

Custom Workflow Report

Agilent Trusted Answers

Compound Details

Cpd. 1: C30 H25 F3 N2 O3

Compound Spectra

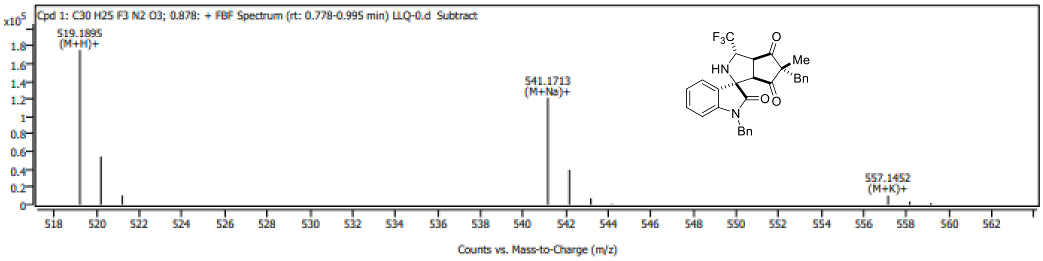

Spectrum Peaks

m/z	m/z (Calc)	Diff (ppm)	Abund	Height %	Height % (Calc)	Ion Species	Z
519.1895	519.1890	1.03	175394	100.00	100.00	(M+H)+	1
520.1925	520.1923	0.56	54535	31.09	33.59	(M+H)+	1
521.1969	521.1953	3.16	10699	6.10	6.08	(M+H)+	1
541.1713	541.1709	0.63	121518	100.00	100.00	(M+Na)+	1
542.1744	542.1742	0.38	39595	32.58	33.58	(M+Na)+	1
543.1776	543.1772	0.66	7280	5.99	6.08	(M+Na)+	1
544.1809	544.1801	1.51	933	0.77	0.78	(M+Na)+	1
557.1452	557.1449	0.49	10442	100.00	100.00	(M+K)+	1
558.1490	558.1481	1.56	3617	34.64	33.59	(M+K)+	1
559.1466	559.1467	-0.17	1795	17.19	13.30	(M+K)+	1

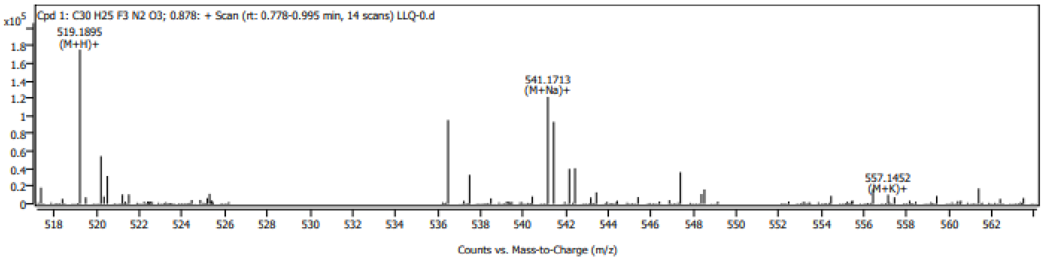

Spectrum Peaks

m/z	m/z (Calc)	Diff (ppm)	Abund	Height %	Height % (Calc)	Ion Species	Z
519.1895	519.1890	1.03	175394	100.00	100.00	(M+H)+	1
520.1925	520.1923	0.56	54535	31.09	33.59	(M+H)+	1
521.1969	521.1953	3.16	10699	6.10	6.08	(M+H)+	1
541.1713	541.1709	0.63	121518	100.00	100.00	(M+Na)+	1
542.1744	542.1742	0.38	39595	32.58	33.58	(M+Na)+	1
543.1776	543.1772	0.66	7280	5.99	6.08	(M+Na)+	1
544.1809	544.1801	1.51	933	0.77	0.78	(M+Na)+	1
557.1452	557.1449	0.49	10442	100.00	100.00	(M+K)+	1
558.1490	558.1481	1.56	3617	34.64	33.59	(M+K)+	1
559.1466	559.1467	-0.17	1795	17.19	13.30	(M+K)+	1

MS spectra of **3ba**

Custom Workflow Report

Agilent

Compound Details

Cpd. 1: C24 H21 F3 N2 O3

Compound Spectra

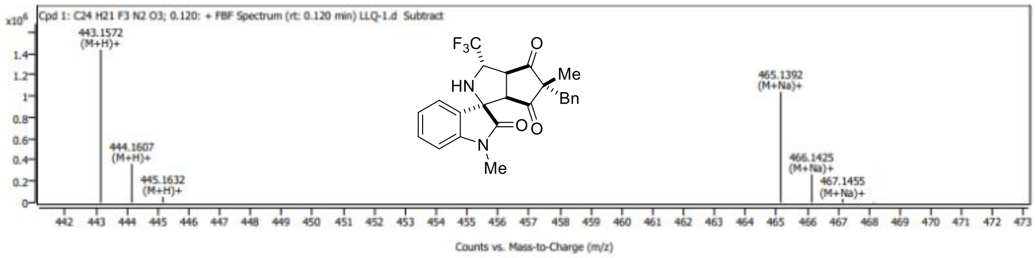

Spectrum Peaks

m/z	m/z (Calc)	Diff (ppm)	Abund	Height %	Height % (Calc)	Ion Species	Z
443.1572	443.1577	-1.12	1437005	100.00	100.00	(M+H)+	1
444.1607	444.1609	-0.53	364258	25.35	27.06	(M+H)+	1
445.1632	445.1638	-1.33	54690	3.81	4.13	(M+H)+	1
465.1392	465.1396	-0.87	1042003	100.00	100.00	(M+Na)+	1
466.1425	466.1429	-0.77	266375	25.56	27.04	(M+Na)+	1
467.1455	467.1457	-0.46	35954	3.45	4.13	(M+Na)+	1
468.1471	468.1485	-3.06	4482	0.43	0.46	(M+Na)+	1

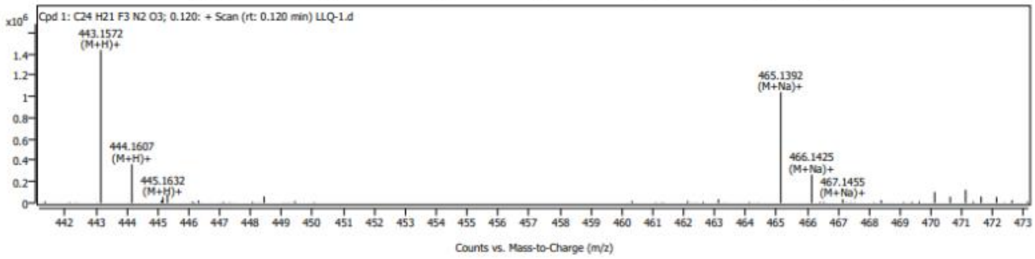

Spectrum Peaks

m/z	m/z (Calc)	Diff (ppm)	Abund	Height %	Height % (Calc)	Ion Species	Z
443.1572	443.1577	-1.12	1437005	100.00	100.00	(M+H)+	1
444.1607	444.1609	-0.53	364258	25.35	27.06	(M+H)+	1
445.1632	445.1638	-1.33	54690	3.81	4.13	(M+H)+	1
465.1392	465.1396	-0.87	1042003	100.00	100.00	(M+Na)+	1
466.1425	466.1429	-0.77	266375	25.56	27.04	(M+Na)+	1
467.1455	467.1457	-0.46	35954	3.45	4.13	(M+Na)+	1
468.1471	468.1485	-3.06	4482	0.43	0.46	(M+Na)+	1

MS spectra of **3ca**

Custom Workflow Report

Agilent

Compound Details

Cpd. 1: C25 H23 F3 N2 O3

Compound Spectra

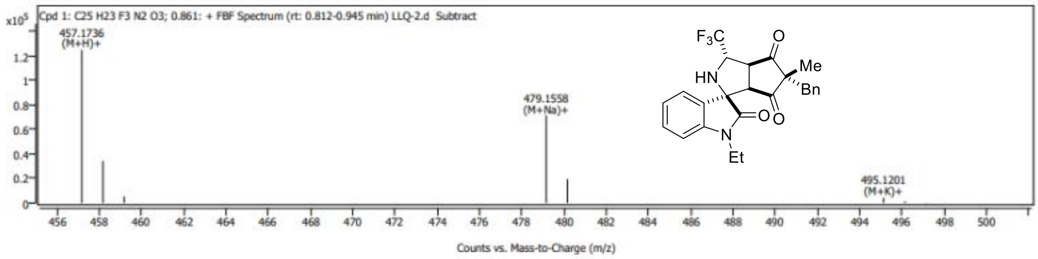

Spectrum Peaks

m/z	m/z (Calc)	Diff (ppm)	Abund	Height %	Height % (Calc)	Ion Species	Z
457.1736	457.1734	0.61	125137	100.00	100.00	(M+H)+	1
458.1768	458.1766	0.44	34441	27.52	28.16	(M+H)+	1
459.1791	459.1795	-0.75	5439	4.35	4.43	(M+H)+	1
479.1558	479.1553	0.99	71603	100.00	100.00	(M+Na)+	1
480.1593	480.1585	1.56	19577	27.34	28.15	(M+Na)+	1
495.1201	495.1292	-18.36	4328	100.00	100.00	(M+K)+	1
496.1332	496.1325	1.47	1424	32.90	28.16	(M+K)+	1
497.1351	497.1304	9.40	314	7.26	11.65	(M+K)+	1

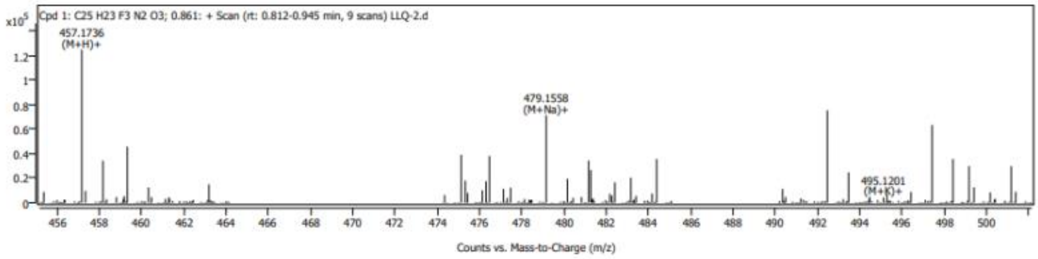

Spectrum Peaks

m/z	m/z (Calc)	Diff (ppm)	Abund	Height %	Height % (Calc)	Ion Species	Z
457.1736	457.1734	0.61	125137	100.00	100.00	(M+H)+	1
458.1768	458.1766	0.44	34441	27.52	28.16	(M+H)+	1
459.1791	459.1795	-0.75	5439	4.35	4.43	(M+H)+	1
479.1558	479.1553	0.99	71603	100.00	100.00	(M+Na)+	1
480.1593	480.1585	1.56	19577	27.34	28.15	(M+Na)+	1
495.1201	495.1292	-18.36	4328	100.00	100.00	(M+K)+	1
496.1332	496.1325	1.47	1424	32.90	28.16	(M+K)+	1
497.1351	497.1304	9.40	314	7.26	11.65	(M+K)+	1

MassHunter Qualitative Analysis   Page 1 of 1   Generated at 2:32 AM on 4/22/2022

MS spectra of **3da**

Custom Workflow Report

Agilent Trusted Answers

Compound Details

Cpd. 1: C26 H23 F3 N2 O3

Compound Spectra

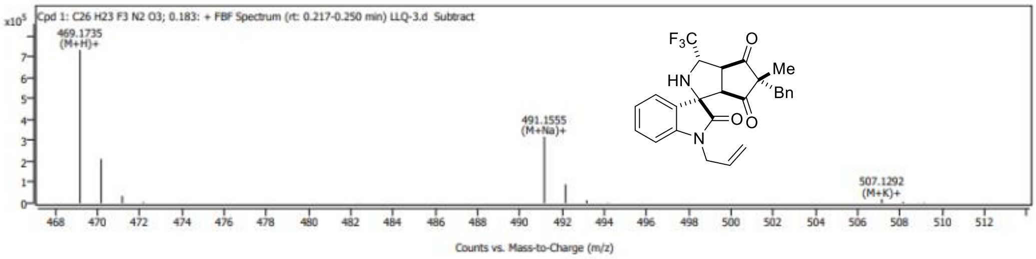

Spectrum Peaks

m/z	m/z (Calc)	Diff (ppm)	Abund	Height %	Height % (Calc)	Ion Species	Z
469.1735	469.1734	0.36	734519	100.00	100.00	(M+H)+	1
470.1767	470.1766	0.16	212012	28.86	29.24	(M+H)+	1
471.1788	471.1795	-1.56	34966	4.76	4.74	(M+H)+	1
472.1811	472.1823	-2.58	4588	0.62	0.55	(M+H)+	1
491.1555	491.1553	0.34	319411	100.00	100.00	(M+Na)+	1
492.1584	492.1585	-0.19	90504	28.33	29.23	(M+Na)+	1
493.1615	493.1615	0.00	14136	4.43	4.74	(M+Na)+	1
494.1635	494.1642	-1.44	2121	0.66	0.55	(M+Na)+	1
507.1292	507.1292	-0.07	20384	100.00	100.00	(M+K)+	1
508.1323	508.1325	-0.40	6441	31.60	29.24	(M+K)+	1
509.1310	509.1305	0.86	2394	11.74	11.96	(M+K)+	1

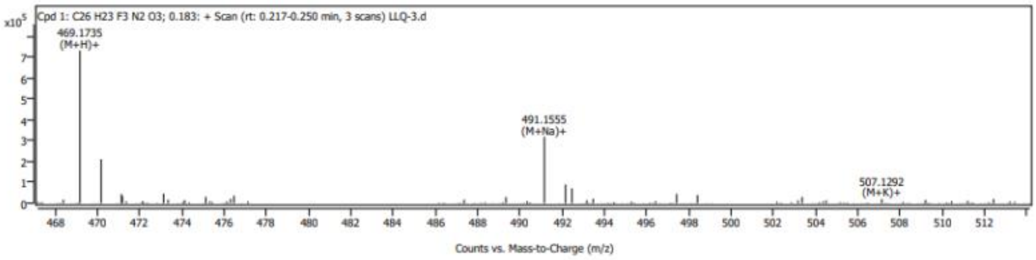

Spectrum Peaks

m/z	m/z (Calc)	Diff (ppm)	Abund	Height %	Height % (Calc)	Ion Species	Z
469.1735	469.1734	0.36	734519	100.00	100.00	(M+H)+	1
470.1767	470.1766	0.16	212012	28.86	29.24	(M+H)+	1
471.1788	471.1795	-1.56	34966	4.76	4.74	(M+H)+	1
472.1811	472.1823	-2.58	4588	0.62	0.55	(M+H)+	1
491.1555	491.1553	0.34	319411	100.00	100.00	(M+Na)+	1
492.1584	492.1585	-0.19	90504	28.33	29.23	(M+Na)+	1
493.1615	493.1615	0.00	14136	4.43	4.74	(M+Na)+	1
494.1635	494.1642	-1.44	2121	0.66	0.55	(M+Na)+	1
507.1292	507.1292	-0.07	20384	100.00	100.00	(M+K)+	1
508.1323	508.1325	-0.40	6441	31.60	29.24	(M+K)+	1
509.1310	509.1305	0.86	2394	11.74	11.96	(M+K)+	1

MS spectra of **3ea**

User Spectrum Plot Report

Name	LLQ-13	Rack Pos.		Instrument	Instrument 1	Operator	
Inj. Vol. (ul)	10	Plate Pos.		IRM Status	Some ions missed		
Data File	LLQ-13.d	Method (Acq)	1367.m	Comment		Acq. Time (Local)	4/15/2022 2:01:27 AM (UTC-07:00)

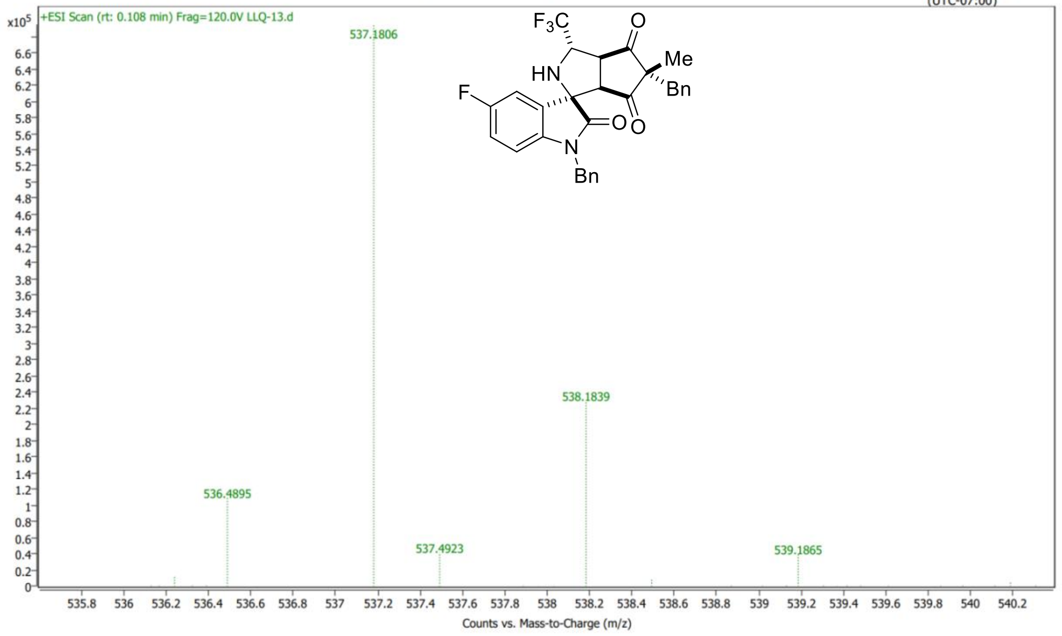

Page 1 of 1

Generated at 2:40 AM on 4/22/2022

MS spectra of **3fa**

User Spectrum Plot Report

Name	LLQ-14	Rack Pos.		Instrument	Instrument 1	Operator	
Inj. Vol. (ul)	10	Plate Pos.		IRM Status	Some ions missed		
Data File	LLQ-14.d	Method (Acq)	1367.m	Comment		Acq. Time (Local)	4/15/2022 2:03:33 AM (UTC-07:00)

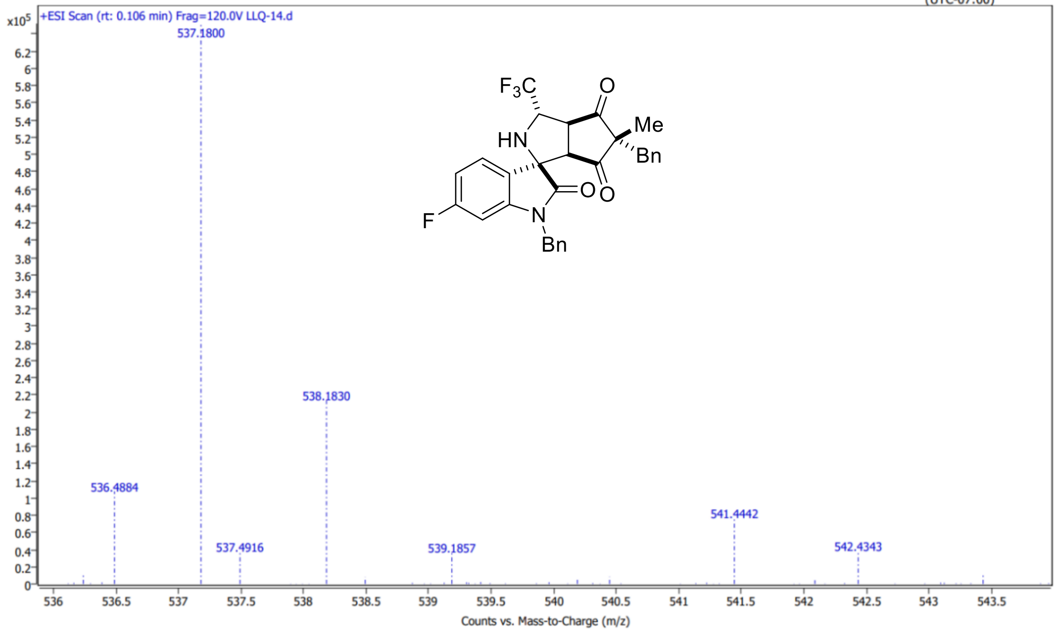

Page 1 of 1

Generated at 2:41 AM on 4/22/2022

MS spectra of **3ga**

Custom Workflow Report

Compound Details

Cpd. 1: C30 H24 Cl F3 N2 O3

Compound Spectra

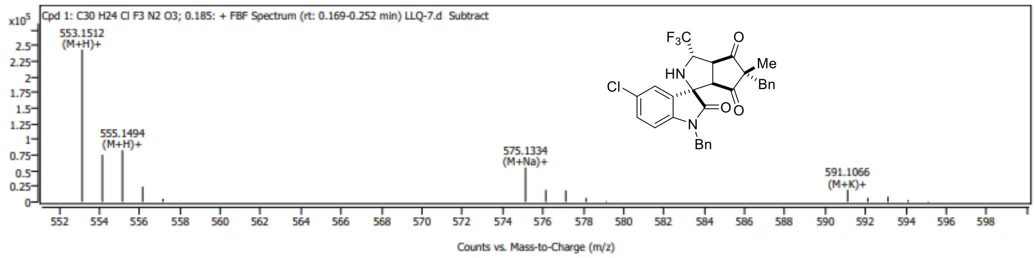

Spectrum Peaks

m/z	m/z (Calc)	Diff (ppm)	Abund	Height %	Height % (Calc)	Ion Species	Z
553.1512	553.1500	2.04	243609	100.00	100.00	(M+H)+	1
554.1541	554.1533	1.49	75418	30.96	33.58	(M+H)+	1
555.1494	555.1486	1.49	83021	34.08	38.07	(M+H)+	1
556.1521	556.1509	2.04	24430	10.03	11.52	(M+H)+	1
557.1545	557.1537	1.50	5195	2.13	2.02	(M+H)+	1
575.1334	575.1320	2.47	55249	100.00	100.00	(M+Na)+	1
576.1414	576.1352	10.75	19313	34.96	33.57	(M+Na)+	1
577.1330	577.1305	4.38	18540	33.56	38.07	(M+Na)+	1
578.1371	578.1329	7.30	6338	11.47	11.52	(M+Na)+	1
579.1373	579.1356	2.94	1482	2.68	2.02	(M+Na)+	1
591.1066	591.1059	1.09	19544	100.00	100.00	(M+K)+	1
592.1097	592.1092	0.89	6651	34.03	33.58	(M+K)+	1
593.1050	593.1044	1.05	8365	42.80	45.29	(M+K)+	1
594.1074	594.1069	0.90	3015	15.43	13.95	(M+K)+	1
595.1078	595.1055	3.88	1057	5.41	4.77	(M+K)+	1

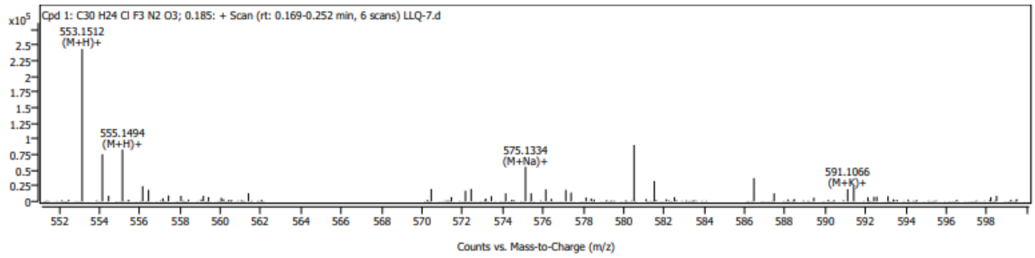

Spectrum Peaks

m/z	m/z (Calc)	Diff (ppm)	Abund	Height %	Height % (Calc)	Ion Species	Z
553.1512	553.1500	2.04	243609	100.00	100.00	(M+H)+	1
554.1541	554.1533	1.49	75418	30.96	33.58	(M+H)+	1
555.1494	555.1486	1.49	83021	34.08	38.07	(M+H)+	1
556.1521	556.1509	2.04	24430	10.03	11.52	(M+H)+	1
557.1545	557.1537	1.50	5195	2.13	2.02	(M+H)+	1
575.1334	575.1320	2.47	55249	100.00	100.00	(M+Na)+	1
576.1414	576.1352	10.75	19313	34.96	33.57	(M+Na)+	1
577.1330	577.1305	4.38	18540	33.56	38.07	(M+Na)+	1
578.1371	578.1329	7.30	6338	11.47	11.52	(M+Na)+	1
579.1373	579.1356	2.94	1482	2.68	2.02	(M+Na)+	1
591.1066	591.1059	1.09	19544	100.00	100.00	(M+K)+	1
592.1097	592.1092	0.89	6651	34.03	33.58	(M+K)+	1
593.1050	593.1044	1.05	8365	42.80	45.29	(M+K)+	1
594.1074	594.1069	0.90	3015	15.43	13.95	(M+K)+	1
595.1078	595.1055	3.88	1057	5.41	4.77	(M+K)+	1

MS spectra of **3ha**

Custom Workflow Report

Compound Details

Cpd. 1: C30 H24 Cl F3 N2 O3

Compound Spectra

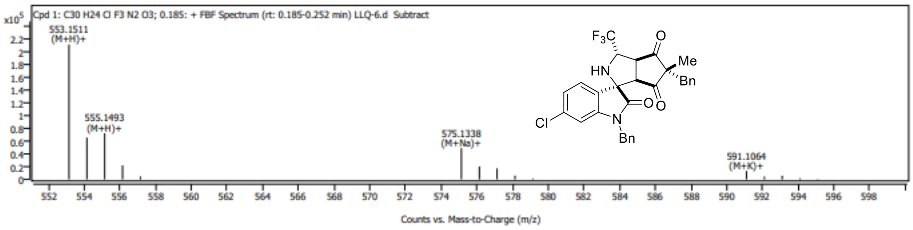

Spectrum Peaks

m/z	m/z (Calc)	Diff (ppm)	Abund	Height %	Height % (Calc)	Ion Species	Z
553.1511	553.1500	1.91	211201	100.00	100.00	(M+H)+	1
554.1541	554.1533	1.51	65510	31.02	33.58	(M+H)+	1
555.1493	555.1486	1.37	72373	34.27	38.07	(M+H)+	1
556.1518	556.1509	1.53	22089	10.46	11.52	(M+H)+	1
557.1548	557.1537	1.99	4463	2.11	2.02	(M+H)+	1
575.1338	575.1320	3.14	48986	100.00	100.00	(M+Na)+	1
576.1426	576.1352	12.82	20223	41.28	33.57	(M+Na)+	1
577.1326	577.1305	3.62	17236	35.18	38.07	(M+Na)+	1
578.1378	578.1329	8.45	5728	11.69	11.52	(M+Na)+	1
579.1418	579.1356	10.76	1576	3.22	2.02	(M+Na)+	1
591.1064	591.1059	0.79	13277	100.00	100.00	(M+K)+	1
592.1099	592.1092	1.30	4359	32.83	33.58	(M+K)+	1
593.1050	593.1044	1.09	5706	42.98	45.29	(M+K)+	1
594.1089	594.1069	3.46	1867	14.06	13.95	(M+K)+	1
595.1029	595.1055	-4.42	891	6.71	4.77	(M+K)+	1

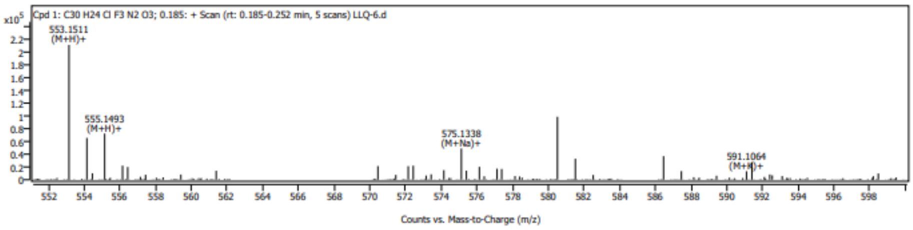

Spectrum Peaks

m/z	m/z (Calc)	Diff (ppm)	Abund	Height %	Height % (Calc)	Ion Species	Z
553.1511	553.1500	1.91	211201	100.00	100.00	(M+H)+	1
554.1541	554.1533	1.51	65510	31.02	33.58	(M+H)+	1
555.1493	555.1486	1.37	72373	34.27	38.07	(M+H)+	1
556.1518	556.1509	1.53	22089	10.46	11.52	(M+H)+	1
557.1548	557.1537	1.99	4463	2.11	2.02	(M+H)+	1
575.1338	575.1320	3.14	48986	100.00	100.00	(M+Na)+	1
576.1426	576.1352	12.82	20223	41.28	33.57	(M+Na)+	1
577.1326	577.1305	3.62	17236	35.18	38.07	(M+Na)+	1
578.1378	578.1329	8.45	5728	11.69	11.52	(M+Na)+	1
579.1418	579.1356	10.76	1576	3.22	2.02	(M+Na)+	1
591.1064	591.1059	0.79	13277	100.00	100.00	(M+K)+	1
592.1099	592.1092	1.30	4359	32.83	33.58	(M+K)+	1
593.1050	593.1044	1.09	5706	42.98	45.29	(M+K)+	1
594.1089	594.1069	3.46	1867	14.06	13.95	(M+K)+	1
595.1029	595.1055	-4.42	891	6.71	4.77	(M+K)+	1

MS spectra of **3ia**

Custom Workflow Report

Agilent

Compound Details

Cpd. 1: C30 H24 Cl F3 N2 O3

Compound Spectra

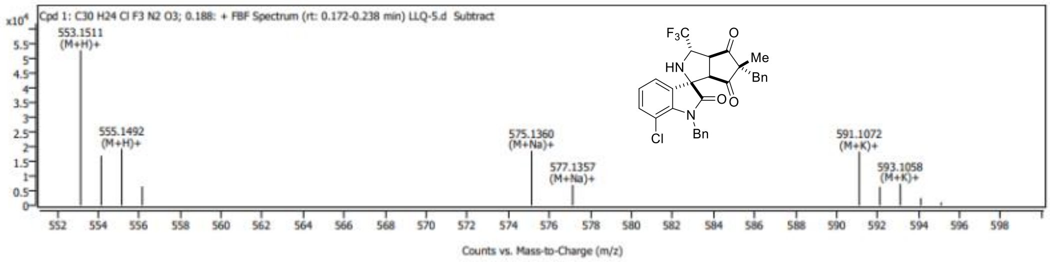

Spectrum Peaks

m/z	m/z (Calc)	Diff (ppm)	Abund	Height %	Height % (Calc)	Ion Species	Z
553.1511	553.1500	2.01	52565	100.00	100.00	(M+H)+	1
554.1546	554.1533	2.34	16787	31.94	33.58	(M+H)+	1
555.1492	555.1486	1.15	19212	36.55	38.07	(M+H)+	1
556.1526	556.1509	2.94	6427	12.23	11.52	(M+H)+	1
575.1360	575.1320	6.92	18502	100.00	100.00	(M+Na)+	1
577.1357	577.1305	9.07	6943	37.53	38.07	(M+Na)+	1
591.1072	591.1059	2.10	18238	100.00	100.00	(M+K)+	1
592.1108	592.1092	2.79	6278	34.42	33.58	(M+K)+	1
593.1058	593.1044	2.41	7312	40.09	45.29	(M+K)+	1
594.1090	594.1069	3.60	2471	13.55	13.95	(M+K)+	1
595.1030	595.1055	-4.29	1039	5.70	4.77	(M+K)+	1

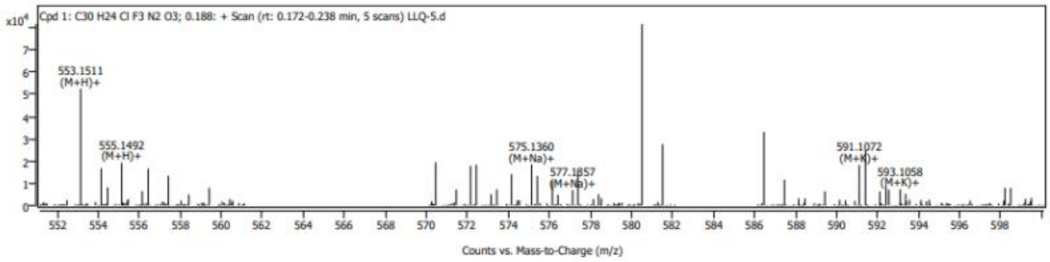

Spectrum Peaks

m/z	m/z (Calc)	Diff (ppm)	Abund	Height %	Height % (Calc)	Ion Species	Z
553.1511	553.1500	2.01	52565	100.00	100.00	(M+H)+	1
554.1546	554.1533	2.34	16787	31.94	33.58	(M+H)+	1
555.1492	555.1486	1.15	19212	36.55	38.07	(M+H)+	1
556.1526	556.1509	2.94	6427	12.23	11.52	(M+H)+	1
575.1360	575.1320	6.92	18502	100.00	100.00	(M+Na)+	1
577.1357	577.1305	9.07	6943	37.53	38.07	(M+Na)+	1
591.1072	591.1059	2.10	18238	100.00	100.00	(M+K)+	1
592.1108	592.1092	2.79	6278	34.42	33.58	(M+K)+	1
593.1058	593.1044	2.41	7312	40.09	45.29	(M+K)+	1
594.1090	594.1069	3.60	2471	13.55	13.95	(M+K)+	1
595.1030	595.1055	-4.29	1039	5.70	4.77	(M+K)+	1

MassHunter Qualitative Analysis Page 1 of 1 Generated at 2:32 AM on 4/22/2022

MS spectra of **3ja**

Custom Workflow Report

Compound Details

Cpd. 1: C30 H24 Br F3 N2 O3

Compound Spectra

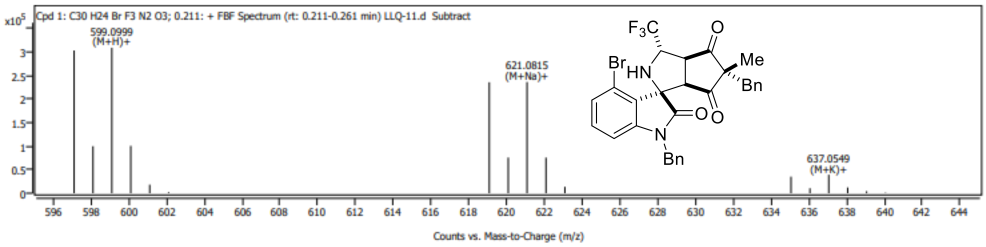

Spectrum Peaks

m/z	m/z (Calc)	Diff (ppm)	Abund	Height %	Height % (Calc)	Ion Species	Z
597.1014	597.0995	3.18	303368	98.12	96.75	(M+H)+	1
598.1044	598.1028	2.72	99940	32.33	32.49	(M+H)+	1
599.0999	599.0980	3.18	309167	100.00	100.00	(M+H)+	1
600.1024	600.1009	2.58	100665	32.56	32.36	(M+H)+	1
601.1055	601.1038	2.77	18138	5.87	5.80	(M+H)+	1
602.1082	602.1067	2.57	2120	0.69	0.74	(M+H)+	1
619.0831	619.0815	2.61	235798	99.80	96.76	(M+Na)+	1
620.0862	620.0847	2.35	75780	32.07	32.48	(M+Na)+	1
621.0815	621.0799	2.63	236277	100.00	100.00	(M+Na)+	1
622.0841	622.0828	2.03	75675	32.03	32.35	(M+Na)+	1
623.0868	623.0858	1.63	13552	5.74	5.79	(M+Na)+	1
635.0566	635.0554	1.86	35115	89.59	90.44	(M+K)+	1
636.0599	636.0586	1.94	10573	26.98	30.37	(M+K)+	1
637.0549	637.0538	1.67	39194	100.00	100.00	(M+K)+	1
638.0577	638.0568	1.37	12190	31.10	32.44	(M+K)+	1
639.0574	639.0554	3.14	4295	10.96	12.16	(M+K)+	1
640.0591	640.0567	3.72	1102	2.81	2.87	(M+K)+	1

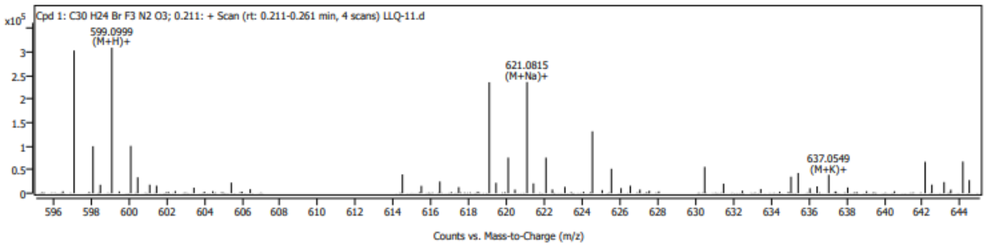

Spectrum Peaks

m/z	m/z (Calc)	Diff (ppm)	Abund	Height %	Height % (Calc)	Ion Species	Z
597.1014	597.0995	3.18	303368	98.12	96.75	(M+H)+	1
598.1044	598.1028	2.72	99940	32.33	32.49	(M+H)+	1
599.0999	599.0980	3.18	309167	100.00	100.00	(M+H)+	1
600.1024	600.1009	2.58	100665	32.56	32.36	(M+H)+	1
601.1055	601.1038	2.77	18138	5.87	5.80	(M+H)+	1
602.1082	602.1067	2.57	2120	0.69	0.74	(M+H)+	1
619.0831	619.0815	2.61	235798	99.80	96.76	(M+Na)+	1
620.0862	620.0847	2.35	75780	32.07	32.48	(M+Na)+	1
621.0815	621.0799	2.63	236277	100.00	100.00	(M+Na)+	1
622.0841	622.0828	2.03	75675	32.03	32.35	(M+Na)+	1
623.0868	623.0858	1.63	13552	5.74	5.79	(M+Na)+	1
635.0566	635.0554	1.86	35115	89.59	90.44	(M+K)+	1
636.0599	636.0586	1.94	10573	26.98	30.37	(M+K)+	1
637.0549	637.0538	1.67	39194	100.00	100.00	(M+K)+	1
638.0577	638.0568	1.37	12190	31.10	32.44	(M+K)+	1
639.0574	639.0554	3.14	4295	10.96	12.16	(M+K)+	1
640.0591	640.0567	3.72	1102	2.81	2.87	(M+K)+	1

MassHunter Qualitative Analysis   Page 1 of 1   Generated at 9:04 PM on 5/1/2022

MS spectra of **3ka**

Custom Workflow Report

Agilent

Compound Details

Cpd. 1: C30 H24 Br F3 N2 O3

Compound Spectra

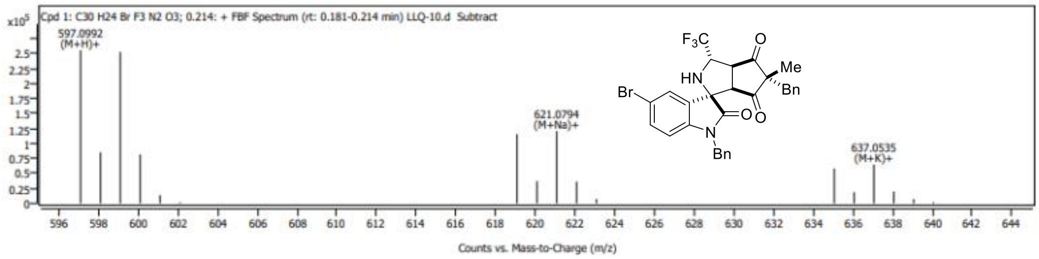

Spectrum Peaks

m/z	m/z (Calc)	Diff (ppm)	Abund	Height %	Height % (Calc)	Ion Species	Z
597.0992	597.0995	-0.61	255553	100.00	96.75	(M+H)+	1
598.1021	598.1028	-1.09	85199	33.34	32.49	(M+H)+	1
599.0977	599.0980	-0.49	252830	98.93	100.00	(M+H)+	1
600.1003	600.1009	-0.98	81816	32.02	32.36	(M+H)+	1
601.1033	601.1038	-0.87	13869	5.43	5.80	(M+H)+	1
602.1068	602.1067	0.19	1872	0.73	0.74	(M+H)+	1
619.0810	619.0815	-0.75	115690	95.80	96.76	(M+Na)+	1
620.0839	620.0847	-1.23	37375	30.95	32.48	(M+Na)+	1
621.0794	621.0799	-0.85	120767	100.00	100.00	(M+Na)+	1
622.0821	622.0828	-1.16	36341	30.09	32.35	(M+Na)+	1
623.0844	623.0858	-2.24	7433	6.15	5.79	(M+Na)+	1
635.0552	635.0554	-0.34	58299	90.13	90.44	(M+K)+	1
636.0582	636.0586	-0.63	18747	28.98	30.37	(M+K)+	1
637.0535	637.0538	-0.54	64681	100.00	100.00	(M+K)+	1
638.0563	638.0568	-0.68	19686	30.43	32.44	(M+K)+	1
639.0558	639.0554	0.67	7218	11.16	12.16	(M+K)+	1
640.0556	640.0567	-1.80	2018	3.12	2.87	(M+K)+	1

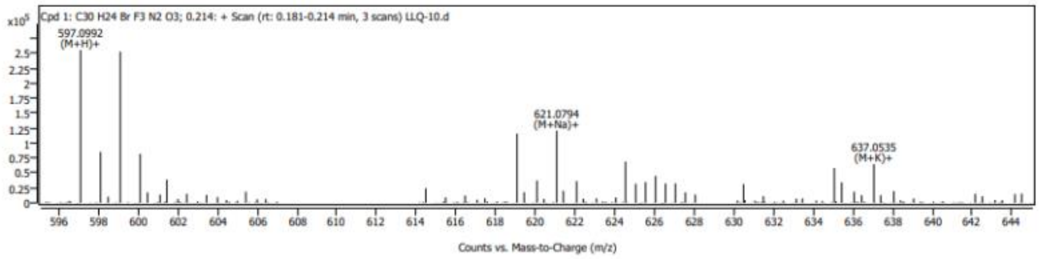

Spectrum Peaks

m/z	m/z (Calc)	Diff (ppm)	Abund	Height %	Height % (Calc)	Ion Species	Z
597.0992	597.0995	-0.61	255553	100.00	96.75	(M+H)+	1
598.1021	598.1028	-1.09	85199	33.34	32.49	(M+H)+	1
599.0977	599.0980	-0.49	252830	98.93	100.00	(M+H)+	1
600.1003	600.1009	-0.98	81816	32.02	32.36	(M+H)+	1
601.1033	601.1038	-0.87	13869	5.43	5.80	(M+H)+	1
602.1068	602.1067	0.19	1872	0.73	0.74	(M+H)+	1
619.0810	619.0815	-0.75	115690	95.80	96.76	(M+Na)+	1
620.0839	620.0847	-1.23	37375	30.95	32.48	(M+Na)+	1
621.0794	621.0799	-0.85	120767	100.00	100.00	(M+Na)+	1
622.0821	622.0828	-1.16	36341	30.09	32.35	(M+Na)+	1
623.0844	623.0858	-2.24	7433	6.15	5.79	(M+Na)+	1
635.0552	635.0554	-0.34	58299	90.13	90.44	(M+K)+	1
636.0582	636.0586	-0.63	18747	28.98	30.37	(M+K)+	1
637.0535	637.0538	-0.54	64681	100.00	100.00	(M+K)+	1
638.0563	638.0568	-0.68	19686	30.43	32.44	(M+K)+	1
639.0558	639.0554	0.67	7218	11.16	12.16	(M+K)+	1
640.0556	640.0567	-1.80	2018	3.12	2.87	(M+K)+	1

MassHunter Qualitative Analysis | Page 1 of 1 | Generated at 9:04 PM on 5/1/2022

MS spectra of **3la**

Custom Workflow Report

Compound Details

Cpd. 1: C30 H24 Br F3 N2 O3

Compound Spectra

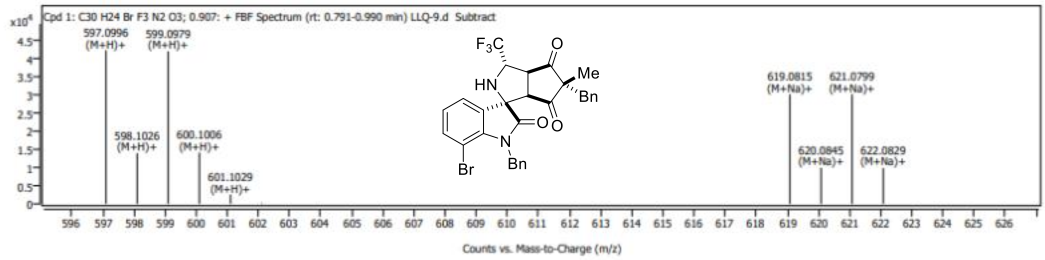

Spectrum Peaks

m/z	m/z (Calc)	Diff (ppm)	Abund	Height %	Height % (Calc)	Ion Species	Z
597.0996	597.0995	0.20	42059	100.00	96.75	(M+H)+	1
598.1026	598.1028	-0.20	13880	33.00	32.49	(M+H)+	1
599.0979	599.0980	-0.17	41789	99.36	100.00	(M+H)+	1
600.1006	600.1009	-0.44	14077	33.47	32.36	(M+H)+	1
601.1029	601.1038	-1.48	2532	6.02	5.80	(M+H)+	1
602.1077	602.1067	1.78	197	0.47	0.74	(M+H)+	1
619.0815	619.0815	0.01	30024	100.00	96.76	(M+Na)+	1
620.0845	620.0847	-0.31	9883	32.92	32.48	(M+Na)+	1
621.0799	621.0799	0.04	30021	99.99	100.00	(M+Na)+	1
622.0829	622.0828	0.02	9794	32.62	32.35	(M+Na)+	1

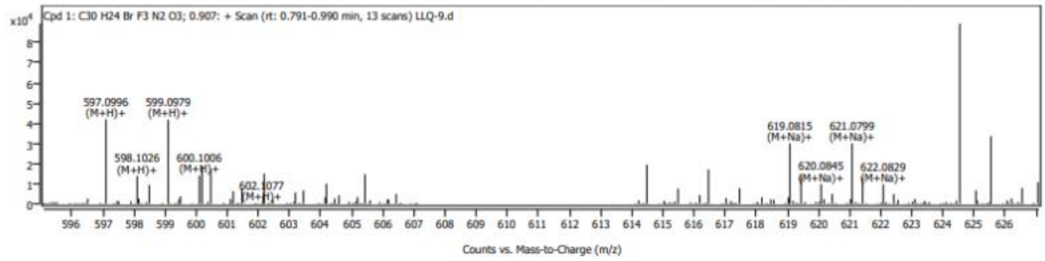

Spectrum Peaks

m/z	m/z (Calc)	Diff (ppm)	Abund	Height %	Height % (Calc)	Ion Species	Z
597.0996	597.0995	0.20	42059	100.00	96.75	(M+H)+	1
598.1026	598.1028	-0.20	13880	33.00	32.49	(M+H)+	1
599.0979	599.0980	-0.17	41789	99.36	100.00	(M+H)+	1
600.1006	600.1009	-0.44	14077	33.47	32.36	(M+H)+	1
601.1029	601.1038	-1.48	2532	6.02	5.80	(M+H)+	1
602.1077	602.1067	1.78	197	0.47	0.74	(M+H)+	1
619.0815	619.0815	0.01	30024	100.00	96.76	(M+Na)+	1
620.0845	620.0847	-0.31	9883	32.92	32.48	(M+Na)+	1
621.0799	621.0799	0.04	30021	99.99	100.00	(M+Na)+	1
622.0829	622.0828	0.02	9794	32.62	32.35	(M+Na)+	1

MS spectra of **3ma**

Custom Workflow Report

Compound Details

Cpd. 1: C31 H24 F6 N2 O3

Compound Spectra

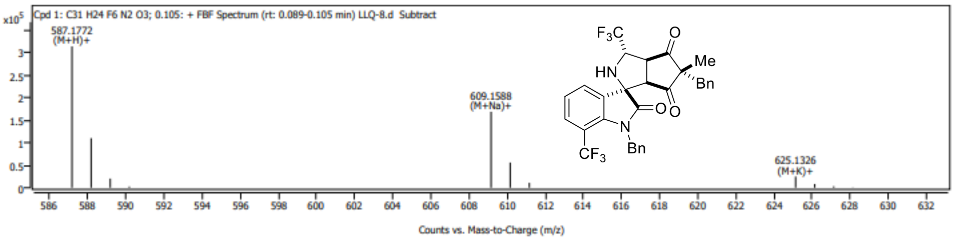

Spectrum Peaks

m/z	m/z (Calc)	Diff (ppm)	Abund	Height %	Height % (Calc)	Ion Species	Z
587.1772	587.1764	1.31	313107	100.00	100.00	(M+H)+	1
588.1801	588.1796	0.77	109910	35.10	34.66	(M+H)+	1
589.1832	589.1827	0.91	20259	6.47	6.44	(M+H)+	1
590.1821	590.1856	-5.82	2818	0.90	0.85	(M+H)+	1
609.1588	609.1583	0.75	168291	100.00	100.00	(M+Na)+	1
610.1620	610.1616	0.62	55804	33.16	34.65	(M+Na)+	1
611.1657	611.1646	1.85	10917	6.49	6.44	(M+Na)+	1
625.1326	625.1323	0.61	24811	100.00	100.00	(M+K)+	1
626.1361	626.1355	0.98	8215	33.11	34.66	(M+K)+	1
627.1330	627.1342	-1.98	3277	13.21	13.66	(M+K)+	1
628.1326	628.1356	-4.83	613	2.47	3.35	(M+K)+	1

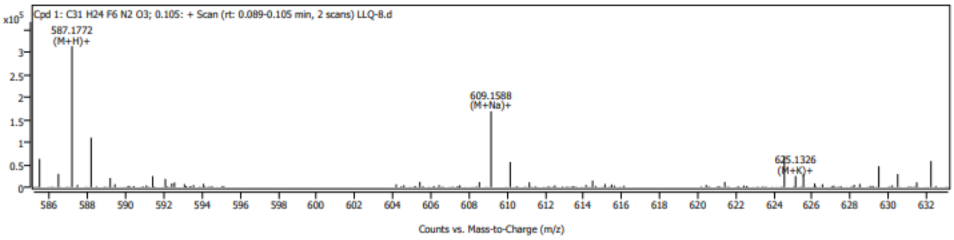

Spectrum Peaks

m/z	m/z (Calc)	Diff (ppm)	Abund	Height %	Height % (Calc)	Ion Species	Z
587.1772	587.1764	1.31	313107	100.00	100.00	(M+H)+	1
588.1801	588.1796	0.77	109910	35.10	34.66	(M+H)+	1
589.1832	589.1827	0.91	20259	6.47	6.44	(M+H)+	1
590.1821	590.1856	-5.82	2818	0.90	0.85	(M+H)+	1
609.1588	609.1583	0.75	168291	100.00	100.00	(M+Na)+	1
610.1620	610.1616	0.62	55804	33.16	34.65	(M+Na)+	1
611.1657	611.1646	1.85	10917	6.49	6.44	(M+Na)+	1
625.1326	625.1323	0.61	24811	100.00	100.00	(M+K)+	1
626.1361	626.1355	0.98	8215	33.11	34.66	(M+K)+	1
627.1330	627.1342	-1.98	3277	13.21	13.66	(M+K)+	1
628.1326	628.1356	-4.83	613	2.47	3.35	(M+K)+	1

MS spectra of **3na**

Custom Workflow Report

Agilent Trusted Answers

Compound Details

Cpd. 1: C31 H27 F3 N2 O4

Compound Spectra

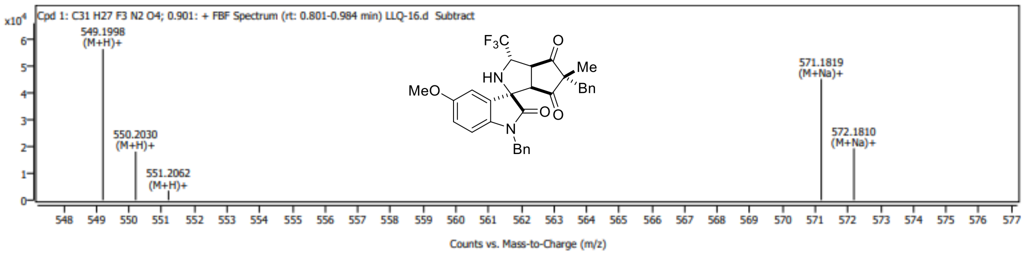

Spectrum Peaks

m/z	m/z (Calc)	Diff (ppm)	Abund	Height %	Height % (Calc)	Ion Species	Z
549.1998	549.1996	0.36	56027	100.00	100.00	(M+H)+	1
550.2030	550.2028	0.27	18008	32.14	34.73	(M+H)+	1
551.2062	551.2058	0.79	3524	6.29	6.67	(M+H)+	1
571.1819	571.1815	0.66	44962	100.00	100.00	(M+Na)+	1
572.1810	572.1848	-6.59	19238	42.79	34.72	(M+Na)+	1

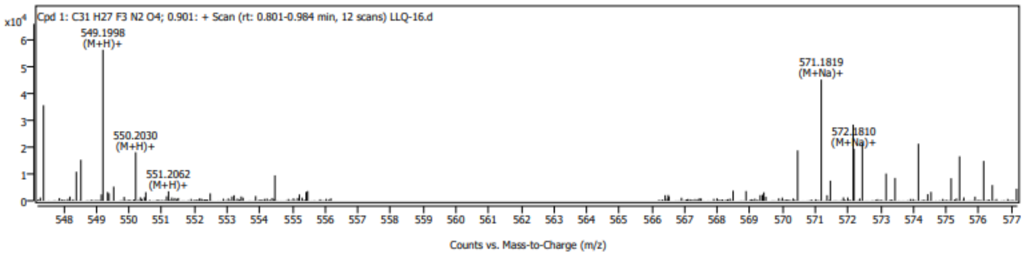

Spectrum Peaks

m/z	m/z (Calc)	Diff (ppm)	Abund	Height %	Height % (Calc)	Ion Species	Z
549.1998	549.1996	0.36	56027	100.00	100.00	(M+H)+	1
550.2030	550.2028	0.27	18008	32.14	34.73	(M+H)+	1
551.2062	551.2058	0.79	3524	6.29	6.67	(M+H)+	1
571.1819	571.1815	0.66	44962	100.00	100.00	(M+Na)+	1
572.1810	572.1848	-6.59	19238	42.79	34.72	(M+Na)+	1

MS spectra of **3oa**

Custom Workflow Report

Compound Details

Cpd. 1: C31 H27 F3 N2 O4

Compound Spectra

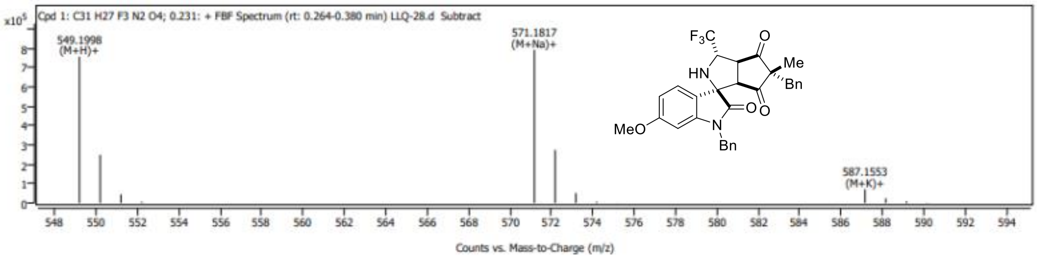

Spectrum Peaks

m/z	m/z (Calc)	Diff (ppm)	Abund	Height %	Height % (Calc)	Ion Species	Z
549.1998	549.1996	0.51	757256	100.00	100.00	(M+H)+	1
550.2030	550.2028	0.34	249121	32.90	34.73	(M+H)+	1
551.2056	551.2058	-0.39	44769	5.91	6.67	(M+H)+	1
552.2086	552.2086	0.03	6303	0.83	0.92	(M+H)+	1
571.1817	571.1815	0.41	792780	100.00	100.00	(M+Na)+	1
572.1849	572.1848	0.21	274659	34.65	34.72	(M+Na)+	1
573.1877	573.1877	0.01	51065	6.44	6.67	(M+Na)+	1
574.1900	574.1906	-0.97	6765	0.85	0.92	(M+Na)+	1
575.1910	575.1933	-4.09	879	0.11	0.10	(M+Na)+	1
587.1553	587.1555	-0.18	70701	100.00	100.00	(M+K)+	1
588.1586	588.1587	-0.11	24685	34.91	34.73	(M+K)+	1
589.1586	589.1575	1.93	9009	12.74	13.89	(M+K)+	1
590.1596	590.1589	1.28	2361	3.34	3.43	(M+K)+	1

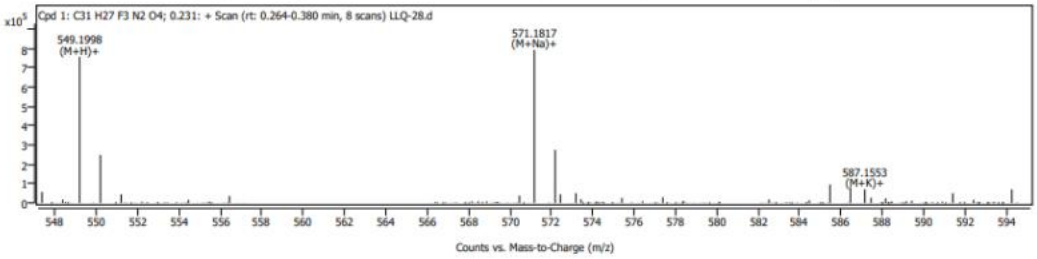

Spectrum Peaks

m/z	m/z (Calc)	Diff (ppm)	Abund	Height %	Height % (Calc)	Ion Species	Z
549.1998	549.1996	0.51	757256	100.00	100.00	(M+H)+	1
550.2030	550.2028	0.34	249121	32.90	34.73	(M+H)+	1
551.2056	551.2058	-0.39	44769	5.91	6.67	(M+H)+	1
552.2086	552.2086	0.03	6303	0.83	0.92	(M+H)+	1
571.1817	571.1815	0.41	792780	100.00	100.00	(M+Na)+	1
572.1849	572.1848	0.21	274659	34.65	34.72	(M+Na)+	1
573.1877	573.1877	0.01	51065	6.44	6.67	(M+Na)+	1
574.1900	574.1906	-0.97	6765	0.85	0.92	(M+Na)+	1
575.1910	575.1933	-4.09	879	0.11	0.10	(M+Na)+	1
587.1553	587.1555	-0.18	70701	100.00	100.00	(M+K)+	1
588.1586	588.1587	-0.11	24685	34.91	34.73	(M+K)+	1
589.1586	589.1575	1.93	9009	12.74	13.89	(M+K)+	1
590.1596	590.1589	1.28	2361	3.34	3.43	(M+K)+	1

MS spectra of **3pa**

Custom Workflow Report

Agilent Trusted Answers

Compound Details

Cpd. 1: C31 H27 F3 N2 O3

Compound Spectra

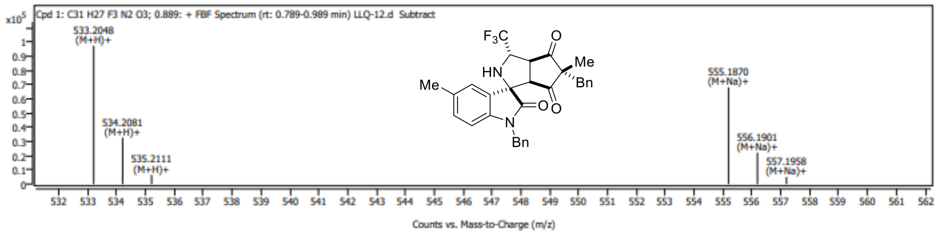

Spectrum Peaks

m/z	m/z (Calc)	Diff (ppm)	Abund	Height %	Height % (Calc)	Ion Species	z
533.2048	533.2047	0.30	97366	100.00	100.00	(M+H)+	1
534.2081	534.2079	0.38	32648	33.53	34.70	(M+H)+	1
535.2111	535.2109	0.34	6469	6.64	6.45	(M+H)+	1
555.1870	555.1866	0.72	67999	100.00	100.00	(M+Na)+	1
556.1901	556.1898	0.40	22122	22.53	34.68	(M+Na)+	1
557.1958	557.1929	5.30	4942	7.27	6.45	(M+Na)+	1

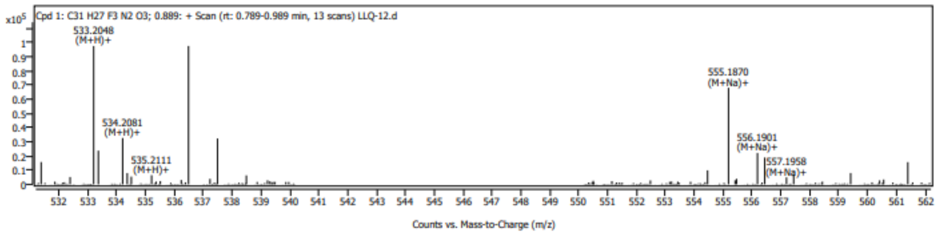

Spectrum Peaks

m/z	m/z (Calc)	Diff (ppm)	Abund	Height %	Height % (Calc)	Ion Species	z
533.2048	533.2047	0.30	97366	100.00	100.00	(M+H)+	1
534.2081	534.2079	0.38	32648	33.53	34.70	(M+H)+	1
535.2111	535.2109	0.34	6469	6.64	6.45	(M+H)+	1
555.1870	555.1866	0.72	67999	100.00	100.00	(M+Na)+	1
556.1901	556.1898	0.40	22122	32.53	34.68	(M+Na)+	1
557.1958	557.1929	5.30	4942	7.27	6.45	(M+Na)+	1

MS spectra of **3qa**

Custom Workflow Report

Compound Details

Cpd. 1: C32 H29 F3 N2 O3

Compound Spectra

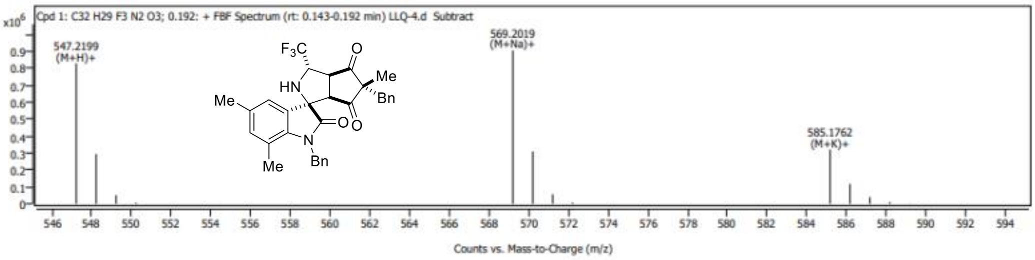

Spectrum Peaks

m/z	m/z (Calc)	Diff (ppm)	Abund	Height %	Height % (Calc)	Ion Species	z
547.2199	547.2203	-0.69	830104	100.00	100.00	(M+H)+	1
548.2234	548.2236	-0.21	294391	35.46	35.80	(M+H)+	1
549.2260	549.2266	-1.03	50994	6.14	6.84	(M+H)+	1
550.2289	550.2295	-1.10	6975	0.84	0.92	(M+H)+	1
569.2019	569.2022	-0.56	906449	100.00	100.00	(M+Na)+	1
570.2054	570.2055	-0.20	309395	34.13	35.79	(M+Na)+	1
571.2079	571.2086	-1.07	56075	6.19	6.83	(M+Na)+	1
572.2113	572.2115	-0.31	7156	0.79	0.92	(M+Na)+	1
585.1762	585.1762	-0.06	320930	100.00	100.00	(M+K)+	1
586.1792	586.1794	-0.36	117808	36.71	35.80	(M+K)+	1
587.1784	587.1783	0.21	39781	12.40	14.05	(M+K)+	1
588.1795	588.1796	-0.22	9833	3.06	3.50	(M+K)+	1
589.1821	589.1819	0.38	1761	0.55	0.59	(M+K)+	1
590.1844	590.1844	0.12	334	0.10	0.07	(M+K)+	1

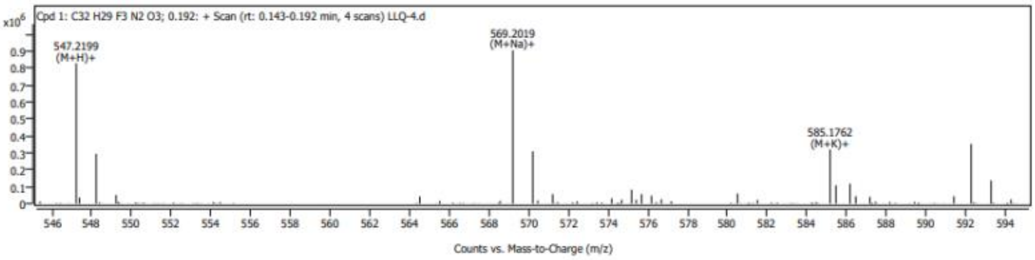

Spectrum Peaks

m/z	m/z (Calc)	Diff (ppm)	Abund	Height %	Height % (Calc)	Ion Species	z
547.2199	547.2203	-0.69	830104	100.00	100.00	(M+H)+	1
548.2234	548.2236	-0.21	294391	35.46	35.80	(M+H)+	1
549.2260	549.2266	-1.03	50994	6.14	6.84	(M+H)+	1
550.2289	550.2295	-1.10	6975	0.84	0.92	(M+H)+	1
569.2019	569.2022	-0.56	906449	100.00	100.00	(M+Na)+	1
570.2054	570.2055	-0.20	309395	34.13	35.79	(M+Na)+	1
571.2079	571.2086	-1.07	56075	6.19	6.83	(M+Na)+	1
572.2113	572.2115	-0.31	7156	0.79	0.92	(M+Na)+	1
585.1762	585.1762	-0.06	320930	100.00	100.00	(M+K)+	1
586.1792	586.1794	-0.36	117808	36.71	35.80	(M+K)+	1
587.1784	587.1783	0.21	39781	12.40	14.05	(M+K)+	1
588.1795	588.1796	-0.22	9833	3.06	3.50	(M+K)+	1
589.1821	589.1819	0.38	1761	0.55	0.59	(M+K)+	1
590.1844	590.1844	0.12	334	0.10	0.07	(M+K)+	1

MS spectra of **3ab**

Custom Workflow Report

Compound Details

Cpd. 1: C31 H27 F3 N2 O3

Compound Spectra

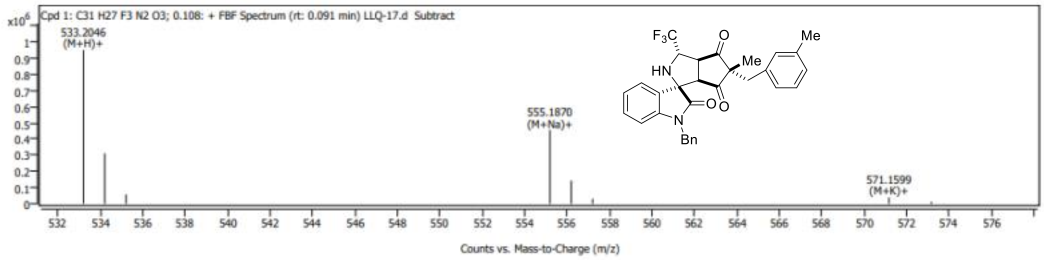

Spectrum Peaks

m/z	m/z (Calc)	Diff (ppm)	Abund	Height %	Height % (Calc)	Ion Species	Z
533.2046	533.2047	-0.05	951190	100.00	100.00	(M+H)+	1
534.2080	534.2079	0.17	312435	32.85	34.70	(M+H)+	1
535.2108	535.2109	-0.17	58263	6.13	6.45	(M+H)+	1
555.1870	555.1866	0.72	456690	100.00	100.00	(M+Na)+	1
556.1897	556.1898	-0.19	143636	31.45	34.68	(M+Na)+	1
557.1931	557.1929	0.39	31059	6.80	6.45	(M+Na)+	1
571.1599	571.1605	-1.16	39104	100.00	100.00	(M+K)+	1
573.1604	573.1625	-3.62	13165	33.67	13.67	(M+K)+	1

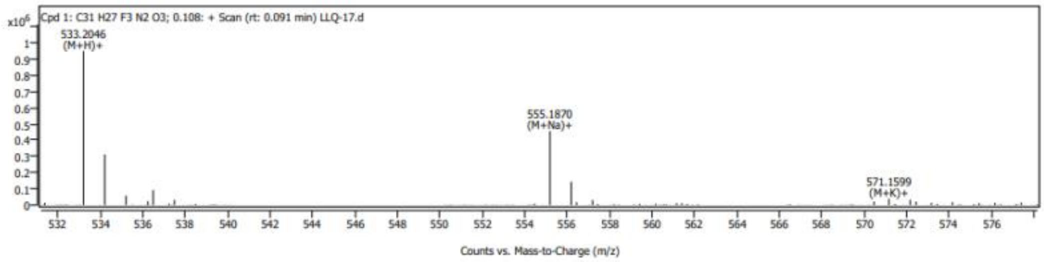

Spectrum Peaks

m/z	m/z (Calc)	Diff (ppm)	Abund	Height %	Height % (Calc)	Ion Species	Z
533.2046	533.2047	-0.05	951190	100.00	100.00	(M+H)+	1
534.2080	534.2079	0.17	312435	32.85	34.70	(M+H)+	1
535.2108	535.2109	-0.17	58263	6.13	6.45	(M+H)+	1
555.1870	555.1866	0.72	456690	100.00	100.00	(M+Na)+	1
556.1897	556.1898	-0.19	143636	31.45	34.68	(M+Na)+	1
557.1931	557.1929	0.39	31059	6.80	6.45	(M+Na)+	1
571.1599	571.1605	-1.16	39104	100.00	100.00	(M+K)+	1
573.1604	573.1625	-3.62	13165	33.67	13.67	(M+K)+	1

MS spectra of **3ac**

Custom Workflow Report

Agilent Trusted Answers

Compound Details

Cpd. 1: C31 H27 F3 N2 O3

Compound Spectra

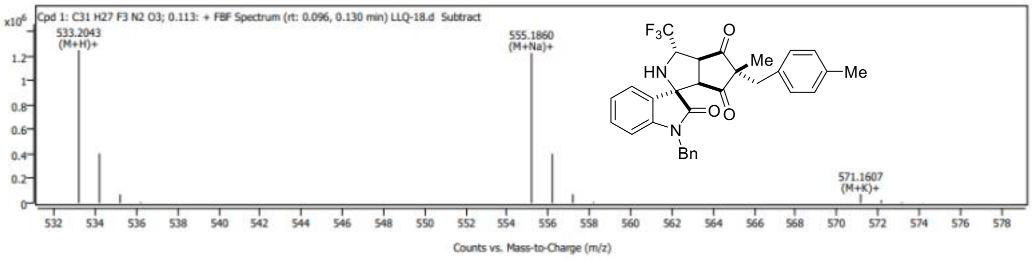

Spectrum Peaks

m/z	m/z (Calc)	Diff (ppm)	Abund	Height %	Height % (Calc)	Ion Species	Z
533.2043	533.2047	-0.75	1249956	100.00	100.00	(M+H)+	1
534.2077	534.2079	-0.36	406279	32.50	34.70	(M+H)+	1
535.2102	535.2109	-1.42	71651	5.73	6.45	(M+H)+	1
536.2131	536.2138	-1.41	9869	0.79	0.85	(M+H)+	1
555.1860	555.1866	-1.09	1229229	100.00	100.00	(M+Na)+	1
556.1895	556.1898	-0.64	404334	32.89	34.68	(M+Na)+	1
557.1921	557.1929	-1.48	72708	5.91	6.45	(M+Na)+	1
558.1950	558.1958	-1.34	9147	0.74	0.85	(M+Na)+	1
571.1607	571.1605	0.32	74153	100.00	100.00	(M+K)+	1
572.1640	572.1638	0.36	24006	32.37	34.70	(M+K)+	1
573.1623	573.1625	-0.40	8471	11.42	13.67	(M+K)+	1
574.1649	574.1639	1.71	2420	3.26	3.35	(M+K)+	1

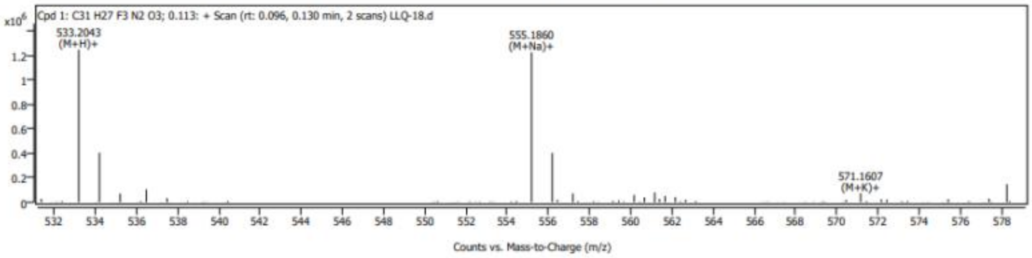

Spectrum Peaks

m/z	m/z (Calc)	Diff (ppm)	Abund	Height %	Height % (Calc)	Ion Species	Z
533.2043	533.2047	-0.75	1249956	100.00	100.00	(M+H)+	1
534.2077	534.2079	-0.36	406279	32.50	34.70	(M+H)+	1
535.2102	535.2109	-1.42	71651	5.73	6.45	(M+H)+	1
536.2131	536.2138	-1.41	9869	0.79	0.85	(M+H)+	1
555.1860	555.1866	-1.09	1229229	100.00	100.00	(M+Na)+	1
556.1895	556.1898	-0.64	404334	32.89	34.68	(M+Na)+	1
557.1921	557.1929	-1.48	72708	5.91	6.45	(M+Na)+	1
558.1950	558.1958	-1.34	9147	0.74	0.85	(M+Na)+	1
571.1607	571.1605	0.32	74153	100.00	100.00	(M+K)+	1
572.1640	572.1638	0.36	24006	32.37	34.70	(M+K)+	1
573.1623	573.1625	-0.40	8471	11.42	13.67	(M+K)+	1
574.1649	574.1639	1.71	2420	3.26	3.35	(M+K)+	1

MassHunter Qualitative Analysis | Page 1 of 1 | Generated at 9:04 PM on 5/1/2022

MS spectra of **3ad**

Custom Workflow Report

Compound Details

Cpd. 1: C31 H27 F3 N2 O4

Compound Spectra

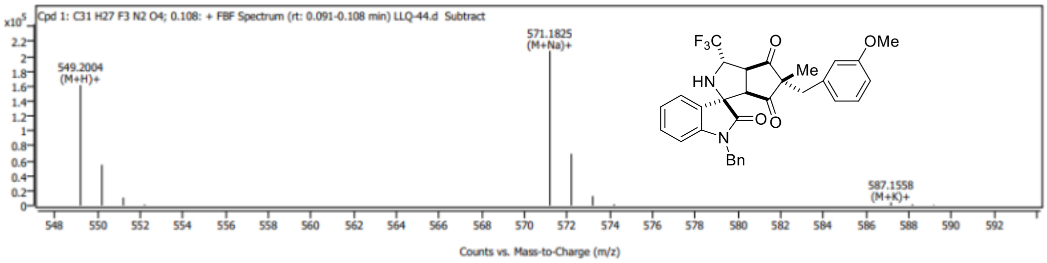

Spectrum Peaks

m/z	m/z (Calc)	Diff (ppm)	Abund	Height %	Height % (Calc)	Ion Species	Z
549.2004	549.1996	1.50	161291	100.00	100.00	(M+H)+	1
550.2034	550.2028	1.11	54645	33.88	34.73	(M+H)+	1
551.2064	551.2058	1.13	10644	6.60	6.67	(M+H)+	1
552.2094	552.2086	1.35	1491	0.92	0.92	(M+H)+	1
571.1825	571.1815	1.70	207364	100.00	100.00	(M+Na)+	1
572.1856	572.1848	1.44	69409	33.47	34.72	(M+Na)+	1
573.1888	573.1877	1.93	12746	6.15	6.67	(M+Na)+	1
574.1906	574.1906	0.05	1875	0.90	0.92	(M+Na)+	1
587.1558	587.1555	0.66	4241	100.00	100.00	(M+K)+	1
588.1603	588.1587	2.76	1799	42.42	34.73	(M+K)+	1
589.1601	589.1575	4.41	874	20.62	13.89	(M+K)+	1

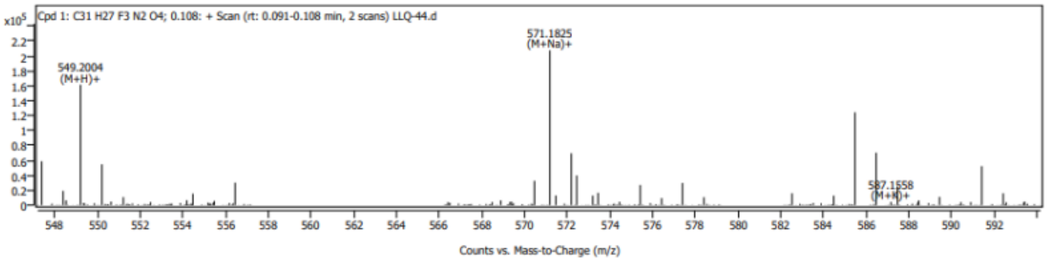

Spectrum Peaks

m/z	m/z (Calc)	Diff (ppm)	Abund	Height %	Height % (Calc)	Ion Species	Z
549.2004	549.1996	1.50	161291	100.00	100.00	(M+H)+	1
550.2034	550.2028	1.11	54645	33.88	34.73	(M+H)+	1
551.2064	551.2058	1.13	10644	6.60	6.67	(M+H)+	1
552.2094	552.2086	1.35	1491	0.92	0.92	(M+H)+	1
571.1825	571.1815	1.70	207364	100.00	100.00	(M+Na)+	1
572.1856	572.1848	1.44	69409	33.47	34.72	(M+Na)+	1
573.1888	573.1877	1.93	12746	6.15	6.67	(M+Na)+	1
574.1906	574.1906	0.05	1875	0.90	0.92	(M+Na)+	1
587.1558	587.1555	0.66	4241	100.00	100.00	(M+K)+	1
588.1603	588.1587	2.76	1799	42.42	34.73	(M+K)+	1
589.1601	589.1575	4.41	874	20.62	13.89	(M+K)+	1

Header
Data

MS spectra of **3ae**

Custom Workflow Report

Agilent Trusted Answers

Compound Details

Cpd. 1: C30 H24 F4 N2 O3

Compound Spectra

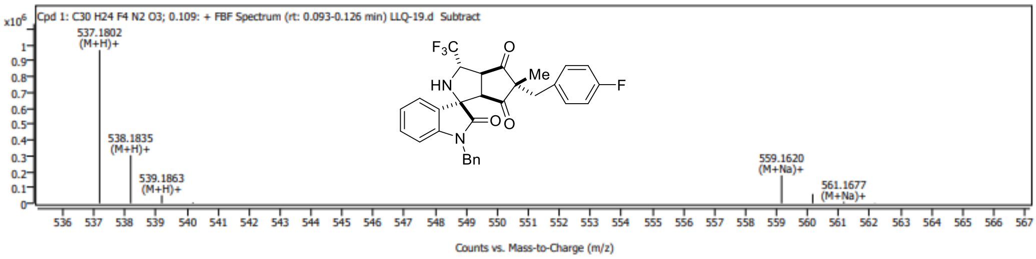

Spectrum Peaks

m/z	m/z (Calc)	Diff (ppm)	Abund	Height %	Height % (Calc)	Ion Species	Z
537.1802	537.1796	1.14	967172	100.00	100.00	(M+H)+	1
538.1835	538.1828	1.28	303638	31.39	33.58	(M+H)+	1
539.1863	539.1858	0.82	50820	5.25	6.08	(M+H)+	1
540.1898	540.1887	1.98	5759	0.60	0.78	(M+H)+	1
559.1620	559.1615	0.76	176686	100.00	100.00	(M+Na)+	1
560.1650	560.1648	0.44	59109	33.45	33.57	(M+Na)+	1
561.1677	561.1678	-0.14	10244	5.80	6.07	(M+Na)+	1
562.1677	562.1707	-5.33	1864	1.06	0.78	(M+Na)+	1

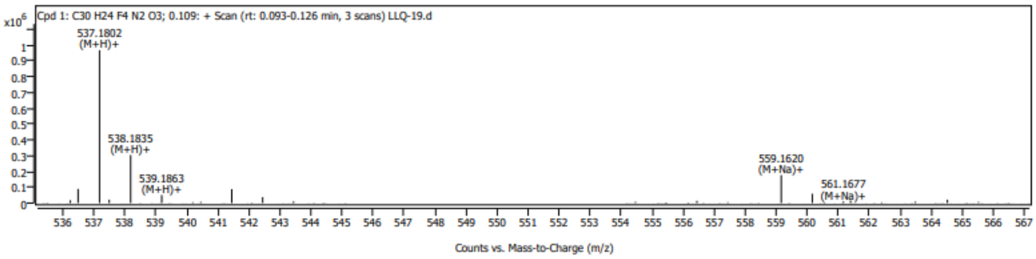

Spectrum Peaks

m/z	m/z (Calc)	Diff (ppm)	Abund	Height %	Height % (Calc)	Ion Species	Z
537.1802	537.1796	1.14	967172	100.00	100.00	(M+H)+	1
538.1835	538.1828	1.28	303638	31.39	33.58	(M+H)+	1
539.1863	539.1858	0.82	50820	5.25	6.08	(M+H)+	1
540.1898	540.1887	1.98	5759	0.60	0.78	(M+H)+	1
559.1620	559.1615	0.76	176686	100.00	100.00	(M+Na)+	1
560.1650	560.1648	0.44	59109	33.45	33.57	(M+Na)+	1
561.1677	561.1678	-0.14	10244	5.80	6.07	(M+Na)+	1
562.1677	562.1707	-5.33	1864	1.06	0.78	(M+Na)+	1

MS spectra of **3af**

Custom Workflow Report

Compound Details

Cpd. 1: C30 H24 Cl F3 N2 O3

Compound Spectra

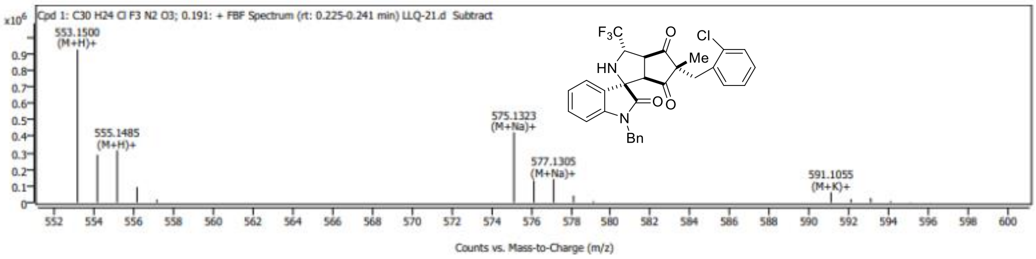

Spectrum Peaks

m/z	m/z (Calc)	Diff (ppm)	Abund	Height %	Height % (Calc)	Ion Species	z
553.1500	553.1500	-0.01	924458	100.00	100.00	(M+H)+	1
554.1533	554.1533	-0.01	288363	31.19	33.58	(M+H)+	1
555.1485	555.1486	-0.02	316246	34.21	38.07	(M+H)+	1
556.1508	556.1509	-0.14	93362	10.10	11.52	(M+H)+	1
557.1528	557.1537	-1.53	17738	1.92	2.02	(M+H)+	1
575.1323	575.1320	0.57	424342	100.00	100.00	(M+Na)+	1
576.1358	576.1352	0.98	133305	31.41	33.57	(M+Na)+	1
577.1305	577.1305	0.02	141336	33.31	38.07	(M+Na)+	1
578.1331	578.1329	0.34	42062	9.91	11.52	(M+Na)+	1
579.1355	579.1356	-0.14	8025	1.89	2.02	(M+Na)+	1
591.1055	591.1059	-0.74	61998	100.00	100.00	(M+K)+	1
592.1091	592.1092	-0.14	20432	32.96	33.58	(M+K)+	1
593.1035	593.1044	-1.55	26998	43.55	45.29	(M+K)+	1
594.1063	594.1069	-1.03	8530	13.76	13.95	(M+K)+	1
595.1063	595.1055	1.30	2317	3.74	4.77	(M+K)+	1
596.1077	596.1067	1.72	614	0.99	1.09	(M+K)+	1

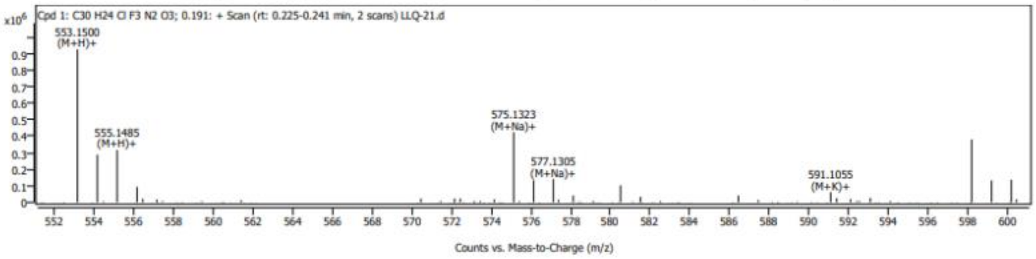

Spectrum Peaks

m/z	m/z (Calc)	Diff (ppm)	Abund	Height %	Height % (Calc)	Ion Species	z
553.1500	553.1500	-0.01	924458	100.00	100.00	(M+H)+	1
554.1533	554.1533	-0.01	288363	31.19	33.58	(M+H)+	1
555.1485	555.1486	-0.02	316246	34.21	38.07	(M+H)+	1
556.1508	556.1509	-0.14	93362	10.10	11.52	(M+H)+	1
557.1528	557.1537	-1.53	17738	1.92	2.02	(M+H)+	1
575.1323	575.1320	0.57	424342	100.00	100.00	(M+Na)+	1
576.1358	576.1352	0.98	133305	31.41	33.57	(M+Na)+	1
577.1305	577.1305	0.02	141336	33.31	38.07	(M+Na)+	1
578.1331	578.1329	0.34	42062	9.91	11.52	(M+Na)+	1
579.1355	579.1356	-0.14	8025	1.89	2.02	(M+Na)+	1
591.1055	591.1059	-0.74	61998	100.00	100.00	(M+K)+	1
592.1091	592.1092	-0.14	20432	32.96	33.58	(M+K)+	1
593.1035	593.1044	-1.55	26998	43.55	45.29	(M+K)+	1
594.1063	594.1069	-1.03	8530	13.76	13.95	(M+K)+	1
595.1063	595.1055	1.30	2317	3.74	4.77	(M+K)+	1
596.1077	596.1067	1.72	614	0.99	1.09	(M+K)+	1

MassHunter Qualitative Analysis   Page 1 of 1   Generated at 2:32 AM on 4/22/2022

MS spectra of **3ag**

Custom Workflow Report

Agilent

Compound Details

Cpd. 1: C30 H24 Cl F3 N2 O3

Compound Spectra

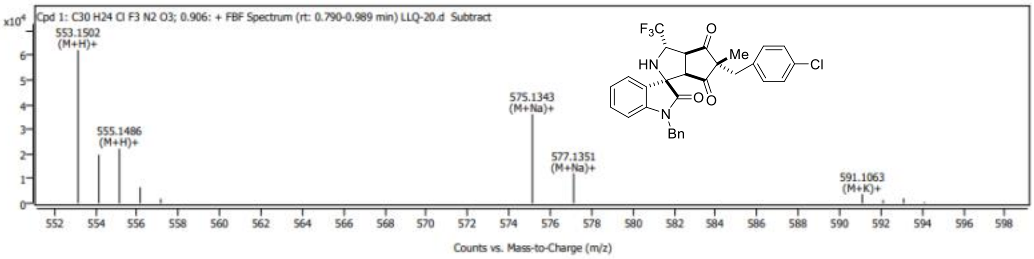

Spectrum Peaks

m/z	m/z (Calc)	Diff (ppm)	Abund	Height %	Height % (Calc)	Ion Species	Z
553.1502	553.1500	0.39	62205	100.00	100.00	(M+H)+	1
554.1534	554.1533	0.28	19713	31.69	33.58	(M+H)+	1
555.1486	555.1486	0.02	22174	35.65	38.07	(M+H)+	1
556.1516	556.1509	1.16	6497	10.44	11.52	(M+H)+	1
557.1539	557.1537	0.33	1829	2.94	2.02	(M+H)+	1
575.1343	575.1320	3.98	36214	100.00	100.00	(M+Na)+	1
577.1351	577.1305	7.91	12213	33.72	38.07	(M+Na)+	1
591.1063	591.1059	0.63	3754	100.00	100.00	(M+K)+	1
592.1090	592.1092	-0.32	1362	36.29	33.58	(M+K)+	1
593.1048	593.1044	0.75	1992	53.07	45.29	(M+K)+	1
594.1063	594.1069	-0.99	476	12.69	13.95	(M+K)+	1

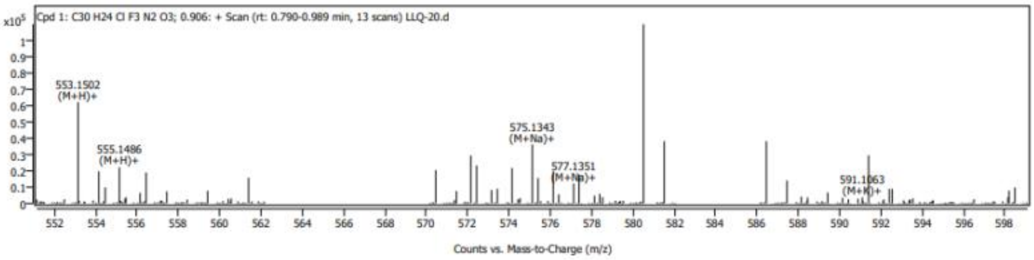

Spectrum Peaks

m/z	m/z (Calc)	Diff (ppm)	Abund	Height %	Height % (Calc)	Ion Species	Z
553.1502	553.1500	0.39	62205	100.00	100.00	(M+H)+	1
554.1534	554.1533	0.28	19713	31.69	33.58	(M+H)+	1
555.1486	555.1486	0.02	22174	35.65	38.07	(M+H)+	1
556.1516	556.1509	1.16	6497	10.44	11.52	(M+H)+	1
557.1539	557.1537	0.33	1829	2.94	2.02	(M+H)+	1
575.1343	575.1320	3.98	36214	100.00	100.00	(M+Na)+	1
577.1351	577.1305	7.91	12213	33.72	38.07	(M+Na)+	1
591.1063	591.1059	0.63	3754	100.00	100.00	(M+K)+	1
592.1090	592.1092	-0.32	1362	36.29	33.58	(M+K)+	1
593.1048	593.1044	0.75	1992	53.07	45.29	(M+K)+	1
594.1063	594.1069	-0.99	476	12.69	13.95	(M+K)+	1

MassHunter Qualitative Analysis Page 1 of 1 Generated at 2:32 AM on 4/22/2022

MS spectra of **3ah**

Custom Workflow Report

Agilent

Compound Details

Cpd. 1: C30 H24 Br F3 N2 O3

Compound Spectra

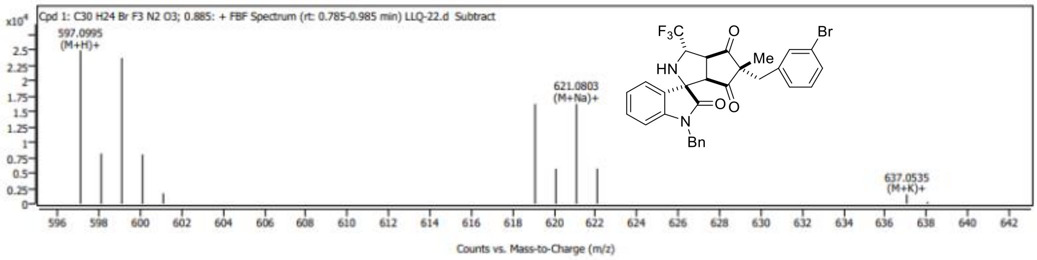

Spectrum Peaks

m/z	m/z (Calc)	Diff (ppm)	Abund	Height %	Height % (Calc)	Ion Species	z
597.0995	597.0995	-0.04	24938	100.00	96.75	(M+H)+	1
598.1027	598.1028	-0.13	8163	32.73	32.49	(M+H)+	1
599.0981	599.0980	0.23	23722	95.12	100.00	(M+H)+	1
600.1008	600.1009	-0.21	8023	32.17	32.36	(M+H)+	1
601.1036	601.1038	-0.43	1678	6.73	5.80	(M+H)+	1
619.0813	619.0815	-0.28	16249	99.93	96.76	(M+Na)+	1
620.0847	620.0847	-0.03	5695	35.03	32.48	(M+Na)+	1
621.0803	621.0799	0.63	16260	100.00	100.00	(M+Na)+	1
622.0829	622.0828	0.09	5729	35.24	32.35	(M+Na)+	1
637.0535	637.0538	-0.50	1513	100.00	100.00	(M+K)+	1
638.0540	638.0568	-4.38	302	19.97	32.44	(M+K)+	1

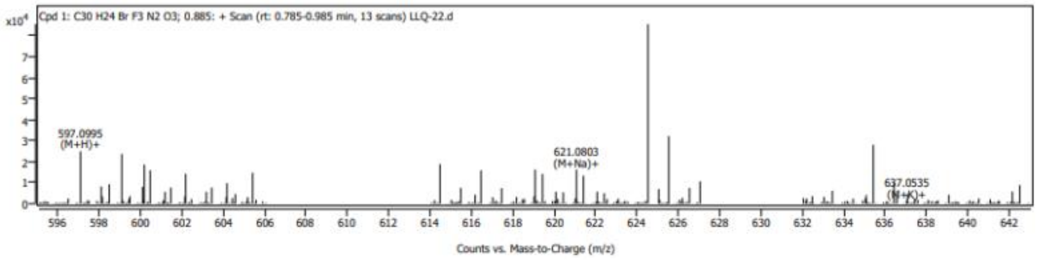

Spectrum Peaks

m/z	m/z (Calc)	Diff (ppm)	Abund	Height %	Height % (Calc)	Ion Species	z
597.0995	597.0995	-0.04	24938	100.00	96.75	(M+H)+	1
598.1027	598.1028	-0.13	8163	32.73	32.49	(M+H)+	1
599.0981	599.0980	0.23	23722	95.12	100.00	(M+H)+	1
600.1008	600.1009	-0.21	8023	32.17	32.36	(M+H)+	1
601.1036	601.1038	-0.43	1678	6.73	5.80	(M+H)+	1
619.0813	619.0815	-0.28	16249	99.93	96.76	(M+Na)+	1
620.0847	620.0847	-0.03	5695	35.03	32.48	(M+Na)+	1
621.0803	621.0799	0.63	16260	100.00	100.00	(M+Na)+	1
622.0829	622.0828	0.09	5729	35.24	32.35	(M+Na)+	1
637.0535	637.0538	-0.50	1513	100.00	100.00	(M+K)+	1
638.0540	638.0568	-4.38	302	19.97	32.44	(M+K)+	1

MassHunter Qualitative Analysis   Page 1 of 1   Generated at 2:32 AM on 4/22/2022

MS spectra of **3ai**

Custom Workflow Report

Compound Details

Cpd. 1: C30 H24 Br F3 N2 O3

Compound Spectra

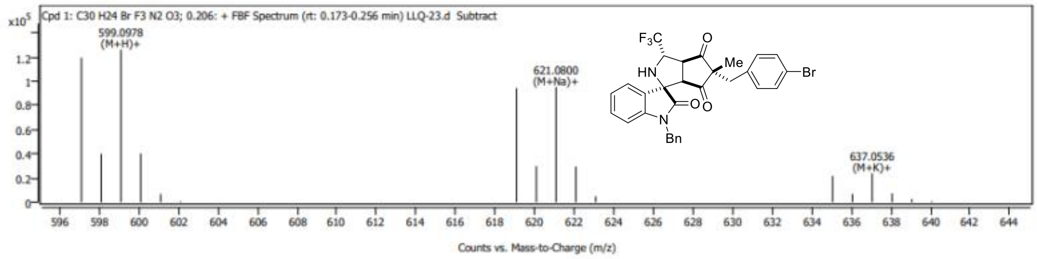

Spectrum Peaks

m/z	m/z (Calc)	Diff (ppm)	Abund	Height %	Height % (Calc)	Ion Species	Z
597.0995	597.0995	0.04	119468	94.78	96.75	(M+H)+	1
598.1026	598.1028	-0.26	39986	31.72	32.49	(M+H)+	1
599.0978	599.0980	-0.28	126047	100.00	100.00	(M+H)+	1
600.1007	600.1009	-0.27	40211	31.90	32.36	(M+H)+	1
601.1034	601.1038	-0.74	6793	5.39	5.80	(M+H)+	1
602.1060	602.1067	-1.13	792	0.63	0.74	(M+H)+	1
619.0815	619.0815	0.04	94233	98.93	96.76	(M+Na)+	1
620.0846	620.0847	-0.24	29844	31.33	32.48	(M+Na)+	1
621.0800	621.0799	0.12	95251	100.00	100.00	(M+Na)+	1
622.0829	622.0828	0.04	29519	30.99	32.35	(M+Na)+	1
623.0856	623.0858	-0.33	4931	5.18	5.79	(M+Na)+	1
635.0552	635.0554	-0.32	21773	90.91	90.44	(M+K)+	1
636.0581	636.0586	-0.92	6745	28.16	30.37	(M+K)+	1
637.0536	637.0538	-0.32	23949	100.00	100.00	(M+K)+	1
638.0570	638.0568	0.35	7323	30.58	32.44	(M+K)+	1
639.0566	639.0554	1.85	2610	10.90	12.16	(M+K)+	1
640.0568	640.0567	0.02	834	3.48	2.87	(M+K)+	1

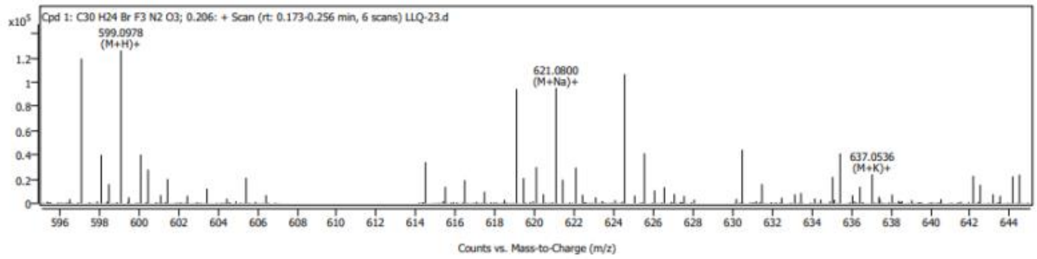

Spectrum Peaks

m/z	m/z (Calc)	Diff (ppm)	Abund	Height %	Height % (Calc)	Ion Species	Z
597.0995	597.0995	0.04	119468	94.78	96.75	(M+H)+	1
598.1026	598.1028	-0.26	39986	31.72	32.49	(M+H)+	1
599.0978	599.0980	-0.28	126047	100.00	100.00	(M+H)+	1
600.1007	600.1009	-0.27	40211	31.90	32.36	(M+H)+	1
601.1034	601.1038	-0.74	6793	5.39	5.80	(M+H)+	1
602.1060	602.1067	-1.13	792	0.63	0.74	(M+H)+	1
619.0815	619.0815	0.04	94233	98.93	96.76	(M+Na)+	1
620.0846	620.0847	-0.24	29844	31.33	32.48	(M+Na)+	1
621.0800	621.0799	0.12	95251	100.00	100.00	(M+Na)+	1
622.0829	622.0828	0.04	29519	30.99	32.35	(M+Na)+	1
623.0856	623.0858	-0.33	4931	5.18	5.79	(M+Na)+	1
635.0552	635.0554	-0.32	21773	90.91	90.44	(M+K)+	1
636.0581	636.0586	-0.92	6745	28.16	30.37	(M+K)+	1
637.0536	637.0538	-0.32	23949	100.00	100.00	(M+K)+	1
638.0570	638.0568	0.35	7323	30.58	32.44	(M+K)+	1
639.0566	639.0554	1.85	2610	10.90	12.16	(M+K)+	1
640.0568	640.0567	0.02	834	3.48	2.87	(M+K)+	1

MassHunter Qualitative Analysis Page 1 of 1 Generated at 9:04 PM on 5/1/2022

MS spectra of **3aj**

Custom Workflow Report

Agilent

Compound Details

Cpd. 1: C34 H27 F3 N2 O3

Compound Spectra

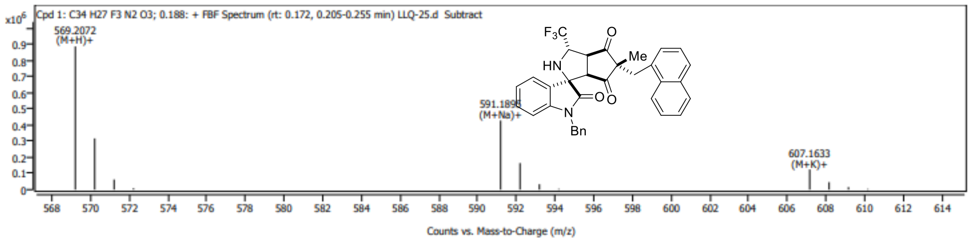

Spectrum Peaks

m/z	m/z (Calc)	Diff (ppm)	Abund	Height %	Height % (Calc)	Ion Species	Z
569.2072	569.2047	4.56	886025	100.00	100.00	(M+H)+	1
570.2107	570.2079	4.96	317127	35.79	37.94	(M+H)+	1
571.2134	571.2110	4.20	62585	7.06	7.61	(M+H)+	1
572.2160	572.2139	3.68	8611	0.97	1.07	(M+H)+	1
591.1895	591.1866	4.92	428151	100.00	100.00	(M+Na)+	1
592.1924	592.1899	4.32	164430	38.40	37.93	(M+Na)+	1
593.1954	593.1929	4.22	33447	7.81	7.61	(M+Na)+	1
594.1976	594.1959	2.86	4683	1.09	1.07	(M+Na)+	1
607.1633	607.1605	4.56	124773	100.00	100.00	(M+K)+	1
608.1663	608.1638	4.16	46378	37.17	37.94	(M+K)+	1
609.1658	609.1629	4.86	15524	12.44	14.83	(M+K)+	1
610.1675	610.1641	5.57	4336	3.47	3.81	(M+K)+	1

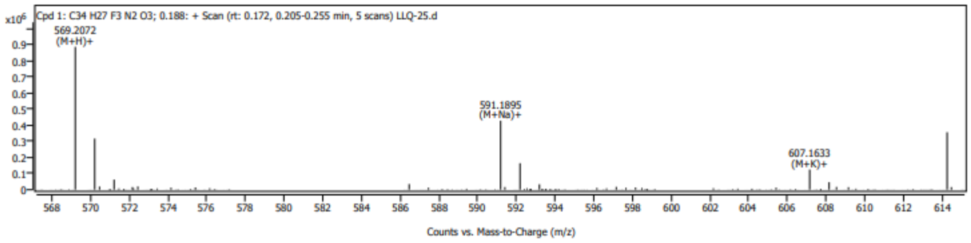

Spectrum Peaks

m/z	m/z (Calc)	Diff (ppm)	Abund	Height %	Height % (Calc)	Ion Species	Z
569.2072	569.2047	4.56	886025	100.00	100.00	(M+H)+	1
570.2107	570.2079	4.96	317127	35.79	37.94	(M+H)+	1
571.2134	571.2110	4.20	62585	7.06	7.61	(M+H)+	1
572.2160	572.2139	3.68	8611	0.97	1.07	(M+H)+	1
591.1895	591.1866	4.92	428151	100.00	100.00	(M+Na)+	1
592.1924	592.1899	4.32	164430	38.40	37.93	(M+Na)+	1
593.1954	593.1929	4.22	33447	7.81	7.61	(M+Na)+	1
594.1976	594.1959	2.86	4683	1.09	1.07	(M+Na)+	1
607.1633	607.1605	4.56	124773	100.00	100.00	(M+K)+	1
608.1663	608.1638	4.16	46378	37.17	37.94	(M+K)+	1
609.1658	609.1629	4.86	15524	12.44	14.83	(M+K)+	1
610.1675	610.1641	5.57	4336	3.47	3.81	(M+K)+	1

MS spectra of **3ak**

Custom Workflow Report

Compound Details

Cpd. 1: C34 H27 F3 N2 O3

Compound Spectra

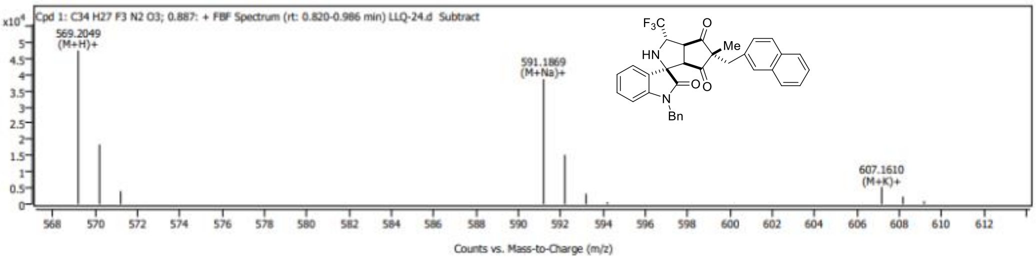

Spectrum Peaks

m/z	m/z (Calc)	Diff (ppm)	Abund	Height %	Height % (Calc)	Ion Species	Z
569.2049	569.2047	0.35	47470	100.00	100.00	(M+H)+	1
570.2081	570.2079	0.32	18422	38.81	37.94	(M+H)+	1
571.2108	571.2110	-0.25	4035	8.50	7.61	(M+H)+	1
591.1869	591.1866	0.45	38621	100.00	100.00	(M+Na)+	1
592.1900	592.1899	0.23	15245	39.47	37.93	(M+Na)+	1
593.1933	593.1929	0.64	3223	8.35	7.61	(M+Na)+	1
594.1998	594.1959	6.63	632	1.64	1.07	(M+Na)+	1
607.1610	607.1605	0.72	5308	100.00	100.00	(M+K)+	1
608.1647	608.1638	1.42	2312	43.55	37.94	(M+K)+	1
609.1623	609.1629	-1.01	907	17.10	14.83	(M+K)+	1

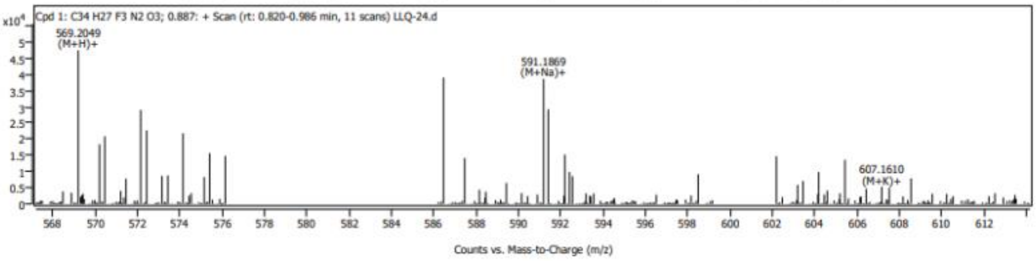

Spectrum Peaks

m/z	m/z (Calc)	Diff (ppm)	Abund	Height %	Height % (Calc)	Ion Species	Z
569.2049	569.2047	0.35	47470	100.00	100.00	(M+H)+	1
570.2081	570.2079	0.32	18422	38.81	37.94	(M+H)+	1
571.2108	571.2110	-0.25	4035	8.50	7.61	(M+H)+	1
591.1869	591.1866	0.45	38621	100.00	100.00	(M+Na)+	1
592.1900	592.1899	0.23	15245	39.47	37.93	(M+Na)+	1
593.1933	593.1929	0.64	3223	8.35	7.61	(M+Na)+	1
594.1998	594.1959	6.63	632	1.64	1.07	(M+Na)+	1
607.1610	607.1605	0.72	5308	100.00	100.00	(M+K)+	1
608.1647	608.1638	1.42	2312	43.55	37.94	(M+K)+	1
609.1623	609.1629	-1.01	907	17.10	14.83	(M+K)+	1

MS spectra of **3aI**

Custom Workflow Report

Agilent

Compound Details

Cpd. 1: C31 H27 F3 N2 O3

Compound Spectra

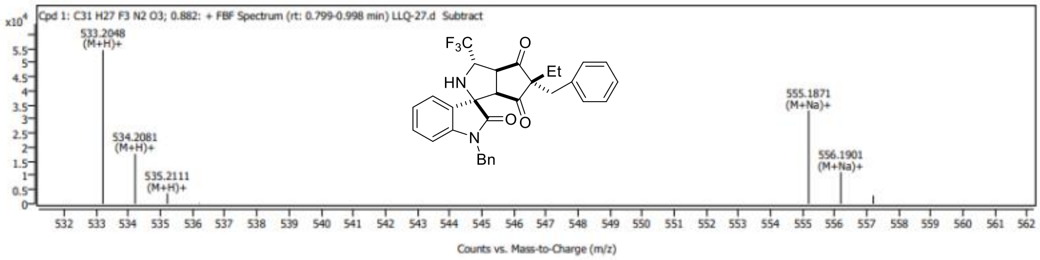

Spectrum Peaks

m/z	m/z (Calc)	Diff (ppm)	Abund	Height %	Height % (Calc)	Ion Species	z
533.2048	533.2047	0.26	54477	100.00	100.00	(M+H)+	1
534.2081	534.2079	0.45	17702	32.49	34.70	(M+H)+	1
535.2111	535.2109	0.24	3723	6.83	6.45	(M+H)+	1
536.2122	536.2138	-3.13	189	0.35	0.85	(M+H)+	1
555.1871	555.1866	0.91	33120	100.00	100.00	(M+Na)+	1
556.1901	556.1898	0.53	11242	33.94	34.68	(M+Na)+	1
557.1988	557.1929	10.68	2914	8.80	6.45	(M+Na)+	1

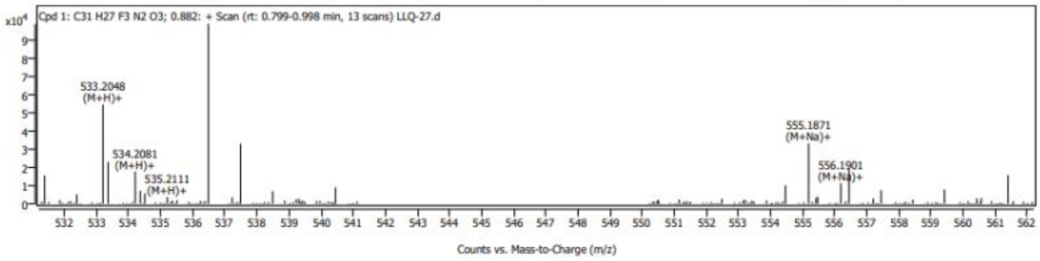

Spectrum Peaks

m/z	m/z (Calc)	Diff (ppm)	Abund	Height %	Height % (Calc)	Ion Species	z
533.2048	533.2047	0.26	54477	100.00	100.00	(M+H)+	1
534.2081	534.2079	0.45	17702	32.49	34.70	(M+H)+	1
535.2111	535.2109	0.24	3723	6.83	6.45	(M+H)+	1
536.2122	536.2138	-3.13	189	0.35	0.85	(M+H)+	1
555.1871	555.1866	0.91	33120	100.00	100.00	(M+Na)+	1
556.1901	556.1898	0.53	11242	33.94	34.68	(M+Na)+	1
557.1988	557.1929	10.68	2914	8.80	6.45	(M+Na)+	1

MS spectra of **3am**

Custom Workflow Report

Compound Details

Cpd. 1: C31 H26 Br F3 N2 O3

Compound Spectra

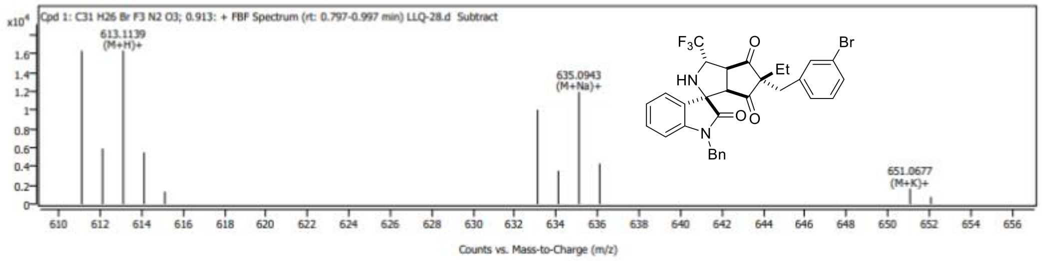

Spectrum Peaks

m/z	m/z (Calc)	Diff (ppm)	Abund	Height %	Height % (Calc)	Ion Species	Z
611.1149	611.1152	-0.36	16313	99.88	96.41	(M+H)+	1
612.1180	612.1184	-0.75	5900	36.13	33.44	(M+H)+	1
613.1139	613.1136	0.36	16333	100.00	100.00	(M+H)+	1
614.1165	614.1166	-0.18	5487	33.60	33.34	(M+H)+	1
615.1214	615.1195	3.07	1330	8.14	6.13	(M+H)+	1
633.0968	633.0971	-0.57	10034	84.04	96.41	(M+Na)+	1
634.1001	634.1004	-0.39	3547	29.71	33.43	(M+Na)+	1
635.0943	635.0956	-2.07	11940	100.00	100.00	(M+Na)+	1
636.0973	636.0985	-1.95	4297	35.99	33.33	(M+Na)+	1
651.0677	651.0695	-2.75	1642	100.00	100.00	(M+K)+	1
652.0729	652.0724	0.76	760	46.29	33.43	(M+K)+	1

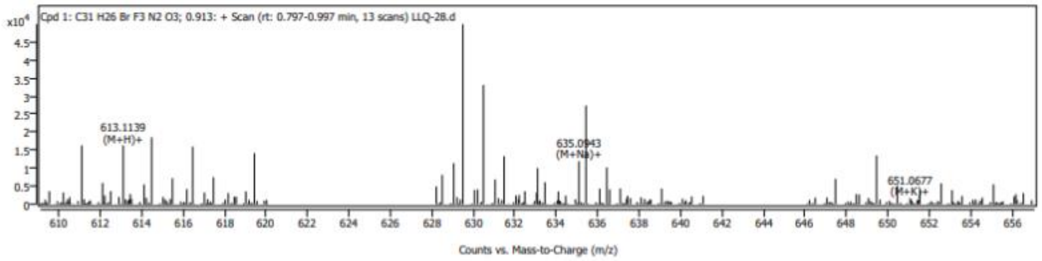

Spectrum Peaks

m/z	m/z (Calc)	Diff (ppm)	Abund	Height %	Height % (Calc)	Ion Species	Z
611.1149	611.1152	-0.36	16313	99.88	96.41	(M+H)+	1
612.1180	612.1184	-0.75	5900	36.13	33.44	(M+H)+	1
613.1139	613.1136	0.36	16333	100.00	100.00	(M+H)+	1
614.1165	614.1166	-0.18	5487	33.60	33.34	(M+H)+	1
615.1214	615.1195	3.07	1330	8.14	6.13	(M+H)+	1
633.0968	633.0971	-0.57	10034	84.04	96.41	(M+Na)+	1
634.1001	634.1004	-0.39	3547	29.71	33.43	(M+Na)+	1
635.0943	635.0956	-2.07	11940	100.00	100.00	(M+Na)+	1
636.0973	636.0985	-1.95	4297	35.99	33.33	(M+Na)+	1
651.0677	651.0695	-2.75	1642	100.00	100.00	(M+K)+	1
652.0729	652.0724	0.76	760	46.29	33.43	(M+K)+	1

MS spectra of **3an**

Custom Workflow Report

Agilent

Compound Details

Cpd. 1: C31 H26 Br F3 N2 O3

Compound Spectra

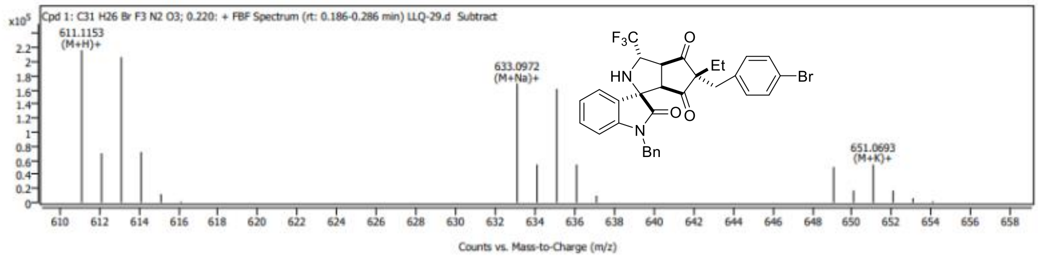

Spectrum Peaks

m/z	m/z (Calc)	Diff (ppm)	Abund	Height %	Height % (Calc)	Ion Species	Z
611.1153	611.1152	0.29	216361	100.00	96.41	(M+H)+	1
612.1184	612.1184	-0.05	70057	32.38	33.44	(M+H)+	1
613.1138	613.1136	0.20	206466	95.43	100.00	(M+H)+	1
614.1165	614.1166	-0.12	71674	33.13	33.34	(M+H)+	1
615.1194	615.1195	-0.12	11955	5.53	6.13	(M+H)+	1
616.1229	616.1224	0.88	1633	0.75	0.80	(M+H)+	1
633.0972	633.0971	0.08	169227	100.00	96.41	(M+Na)+	1
634.1003	634.1004	-0.12	53984	31.90	33.43	(M+Na)+	1
635.0956	635.0956	0.00	161508	95.44	100.00	(M+Na)+	1
636.0986	636.0985	0.07	53971	31.89	33.33	(M+Na)+	1
637.1010	637.1015	-0.70	9759	5.77	6.13	(M+Na)+	1
649.0710	649.0710	-0.14	50521	93.62	90.14	(M+K)+	1
650.0741	650.0743	-0.33	17269	32.00	31.26	(M+K)+	1
651.0693	651.0695	-0.24	53963	100.00	100.00	(M+K)+	1
652.0722	652.0724	-0.40	17167	31.81	33.43	(M+K)+	1
653.0720	653.0712	1.29	6342	11.75	12.48	(M+K)+	1
654.0721	654.0725	-0.55	1996	3.70	3.00	(M+K)+	1

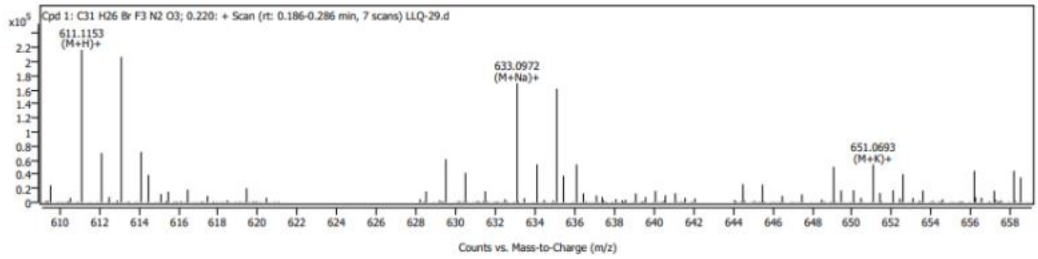

Spectrum Peaks

m/z	m/z (Calc)	Diff (ppm)	Abund	Height %	Height % (Calc)	Ion Species	Z
611.1153	611.1152	0.29	216361	100.00	96.41	(M+H)+	1
612.1184	612.1184	-0.05	70057	32.38	33.44	(M+H)+	1
613.1138	613.1136	0.20	206466	95.43	100.00	(M+H)+	1
614.1165	614.1166	-0.12	71674	33.13	33.34	(M+H)+	1
615.1194	615.1195	-0.12	11955	5.53	6.13	(M+H)+	1
616.1229	616.1224	0.88	1633	0.75	0.80	(M+H)+	1
633.0972	633.0971	0.08	169227	100.00	96.41	(M+Na)+	1
634.1003	634.1004	-0.12	53984	31.90	33.43	(M+Na)+	1
635.0956	635.0956	0.00	161508	95.44	100.00	(M+Na)+	1
636.0986	636.0985	0.07	53971	31.89	33.33	(M+Na)+	1
637.1010	637.1015	-0.70	9759	5.77	6.13	(M+Na)+	1
649.0710	649.0710	-0.14	50521	93.62	90.14	(M+K)+	1
650.0741	650.0743	-0.33	17269	32.00	31.26	(M+K)+	1
651.0693	651.0695	-0.24	53963	100.00	100.00	(M+K)+	1
652.0722	652.0724	-0.40	17167	31.81	33.43	(M+K)+	1
653.0720	653.0712	1.29	6342	11.75	12.48	(M+K)+	1
654.0721	654.0725	-0.55	1996	3.70	3.00	(M+K)+	1

MassHunter Qualitative Analysis | Page 1 of 1 | Generated at 9:04 PM on 5/1/2022

MS spectra of **5a**

Custom Workflow Report

Agilent Trusted Answers

Compound Details

Cpd. 1: C22 H24 F3 N O6

Compound Spectra

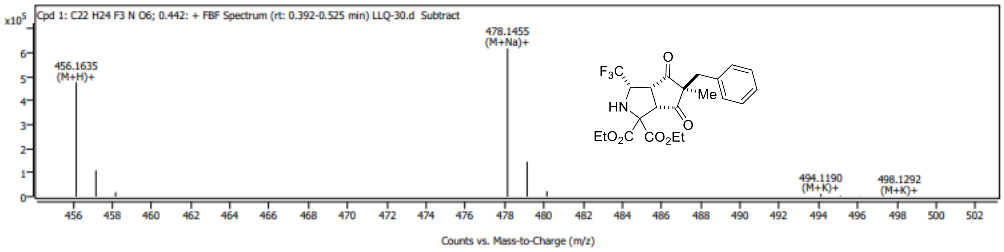

Spectrum Peaks

m/z	m/z (Calc)	Diff (ppm)	Abund	Height %	Height % (Calc)	Ion Species	z
456.1635	456.1628	1.42	478204	100.00	100.00	(M+H)+	1
457.1665	457.1662	0.79	109749	22.95	24.68	(M+H)+	1
458.1691	458.1688	0.83	18224	3.81	4.15	(M+H)+	1
478.1455	478.1448	1.53	619194	100.00	100.00	(M+Na)+	1
479.1487	479.1481	1.18	145815	23.55	24.66	(M+Na)+	1
480.1513	480.1507	1.28	22963	3.71	4.15	(M+Na)+	1
494.1190	494.1187	0.56	10602	100.00	100.00	(M+K)+	1
495.1223	495.1220	0.63	2895	27.30	24.68	(M+K)+	1
496.1200	496.1197	0.63	1254	11.83	11.37	(M+K)+	1
498.1292	498.1238	10.71	54	0.51	0.35	(M+K)+	1

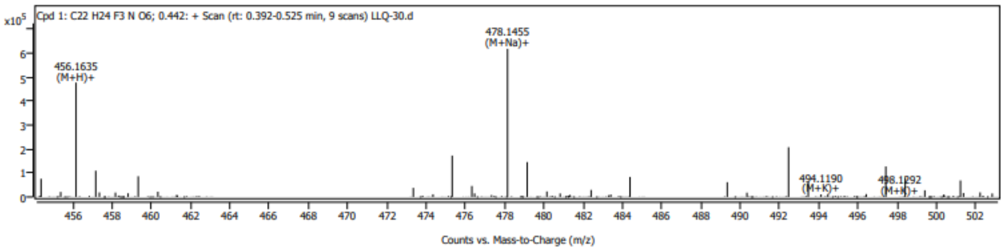

Spectrum Peaks

m/z	m/z (Calc)	Diff (ppm)	Abund	Height %	Height % (Calc)	Ion Species	z
456.1635	456.1628	1.42	478204	100.00	100.00	(M+H)+	1
457.1665	457.1662	0.79	109749	22.95	24.68	(M+H)+	1
458.1691	458.1688	0.83	18224	3.81	4.15	(M+H)+	1
478.1455	478.1448	1.53	619194	100.00	100.00	(M+Na)+	1
479.1487	479.1481	1.18	145815	23.55	24.66	(M+Na)+	1
480.1513	480.1507	1.28	22963	3.71	4.15	(M+Na)+	1
494.1190	494.1187	0.56	10602	100.00	100.00	(M+K)+	1
495.1223	495.1220	0.63	2895	27.30	24.68	(M+K)+	1
496.1200	496.1197	0.63	1254	11.83	11.37	(M+K)+	1
498.1292	498.1238	10.71	54	0.51	0.35	(M+K)+	1

MS spectra of **5b**

Custom Workflow Report

Agilent

Compound Details

Cpd. 1: C23 H26 F3 N O6

Compound Spectra

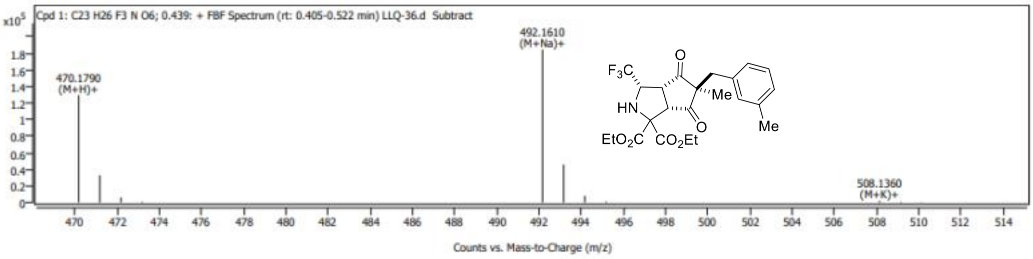

Spectrum Peaks

m/z	m/z (Calc)	Diff (ppm)	Abund	Height %	Height % (Calc)	Ion Species	Z
470.1790	470.1785	1.16	129387	100.00	100.00	(M+H)+	1
471.1819	471.1818	0.30	32758	25.32	25.78	(M+H)+	1
472.1845	472.1845	0.07	6056	4.68	4.42	(M+H)+	1
473.1891	473.1871	4.29	1011	0.78	0.57	(M+H)+	1
492.1610	492.1604	1.22	184478	100.00	100.00	(M+Na)+	1
493.1642	493.1638	0.96	45706	24.78	25.77	(M+Na)+	1
494.1668	494.1664	0.77	8016	4.35	4.42	(M+Na)+	1
495.1714	495.1690	4.77	1259	0.68	0.57	(M+Na)+	1
508.1360	508.1344	3.16	2260	100.00	100.00	(M+K)+	1
509.1418	509.1377	8.02	834	36.88	25.78	(M+K)+	1
510.1344	510.1355	-2.19	486	21.50	11.64	(M+K)+	1

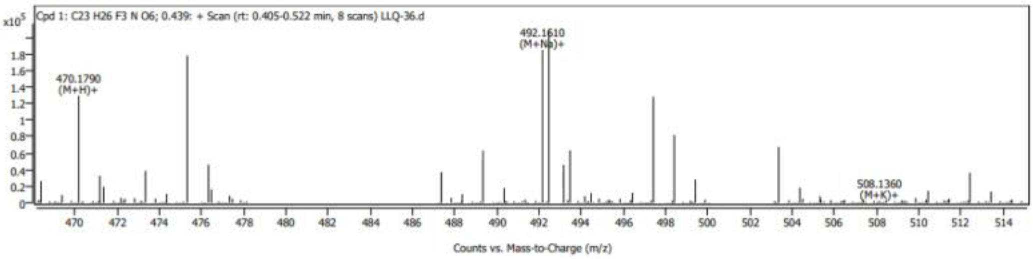

Spectrum Peaks

m/z	m/z (Calc)	Diff (ppm)	Abund	Height %	Height % (Calc)	Ion Species	Z
470.1790	470.1785	1.16	129387	100.00	100.00	(M+H)+	1
471.1819	471.1818	0.30	32758	25.32	25.78	(M+H)+	1
472.1845	472.1845	0.07	6056	4.68	4.42	(M+H)+	1
473.1891	473.1871	4.29	1011	0.78	0.57	(M+H)+	1
492.1610	492.1604	1.22	184478	100.00	100.00	(M+Na)+	1
493.1642	493.1638	0.96	45706	24.78	25.77	(M+Na)+	1
494.1668	494.1664	0.77	8016	4.35	4.42	(M+Na)+	1
495.1714	495.1690	4.77	1259	0.68	0.57	(M+Na)+	1
508.1360	508.1344	3.16	2260	100.00	100.00	(M+K)+	1
509.1418	509.1377	8.02	834	36.88	25.78	(M+K)+	1
510.1344	510.1355	-2.19	486	21.50	11.64	(M+K)+	1

MassHunter Qualitative Analysis   Page 1 of 1   Generated at 1:19 AM on 11/13/2022

MS spectra of **5c**

Custom Workflow Report

Agilent

Compound Details

Cpd. 1: C23 H26 F3 N O6

Compound Spectra

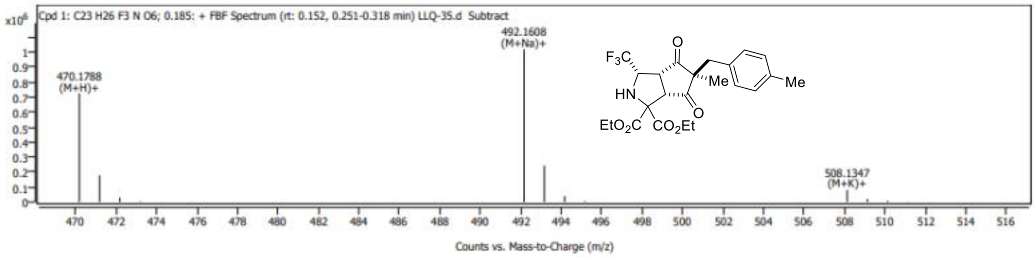

Spectrum Peaks

m/z	m/z (Calc)	Diff (ppm)	Abund	Height %	Height % (Calc)	Ion Species	Z
470.1788	470.1785	0.63	723910	100.00	100.00	(M+H)+	1
471.1820	471.1818	0.39	177929	24.58	25.78	(M+H)+	1
472.1846	472.1845	0.37	28781	3.98	4.42	(M+H)+	1
473.1874	473.1871	0.57	4052	0.56	0.57	(M+H)+	1
492.1608	492.1604	0.69	1018487	100.00	100.00	(M+Na)+	1
493.1640	493.1638	0.43	242763	23.84	25.77	(M+Na)+	1
494.1665	494.1664	0.25	39401	3.87	4.42	(M+Na)+	1
495.1695	495.1690	1.02	5249	0.52	0.57	(M+Na)+	1
508.1347	508.1344	0.65	80836	100.00	100.00	(M+K)+	1
509.1378	509.1377	0.18	20636	25.53	25.78	(M+K)+	1
510.1355	510.1355	0.06	8481	10.49	11.64	(M+K)+	1
511.1362	511.1375	-2.53	1862	2.30	2.43	(M+K)+	1
512.1335	512.1396	-11.85	222	0.27	0.38	(M+K)+	1

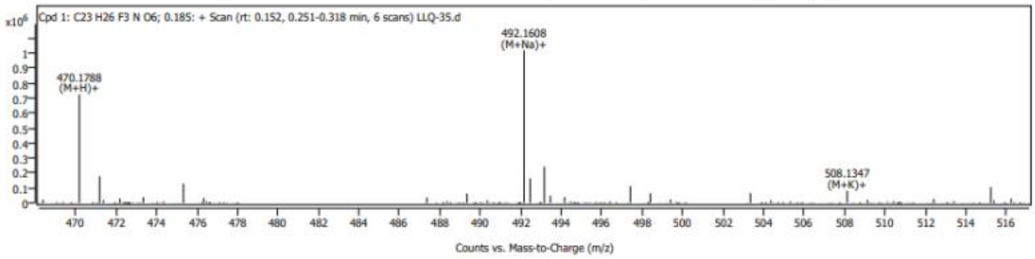

Spectrum Peaks

m/z	m/z (Calc)	Diff (ppm)	Abund	Height %	Height % (Calc)	Ion Species	Z
470.1788	470.1785	0.63	723910	100.00	100.00	(M+H)+	1
471.1820	471.1818	0.39	177929	24.58	25.78	(M+H)+	1
472.1846	472.1845	0.37	28781	3.98	4.42	(M+H)+	1
473.1874	473.1871	0.57	4052	0.56	0.57	(M+H)+	1
492.1608	492.1604	0.69	1018487	100.00	100.00	(M+Na)+	1
493.1640	493.1638	0.43	242763	23.84	25.77	(M+Na)+	1
494.1665	494.1664	0.25	39401	3.87	4.42	(M+Na)+	1
495.1695	495.1690	1.02	5249	0.52	0.57	(M+Na)+	1
508.1347	508.1344	0.65	80836	100.00	100.00	(M+K)+	1
509.1378	509.1377	0.18	20636	25.53	25.78	(M+K)+	1
510.1355	510.1355	0.06	8481	10.49	11.64	(M+K)+	1
511.1362	511.1375	-2.53	1862	2.30	2.43	(M+K)+	1
512.1335	512.1396	-11.85	222	0.27	0.38	(M+K)+	1

MS spectra of **5d**

Custom Workflow Report

Agilent | Trusted Answers

Compound Details

Cpd. 1: C23 H26 F3 N O7

Compound Spectra

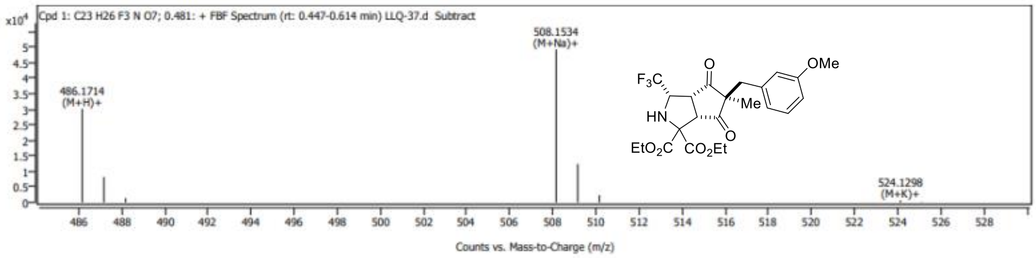

Spectrum Peaks

m/z	m/z (Calc)	Diff (ppm)	Abund	Height %	Height % (Calc)	Ion Species	Z
486.1714	486.1734	-4.18	30213	100.00	100.00	(M+H)+	1
487.1751	487.1767	-3.42	8180	27.07	25.82	(M+H)+	1
488.1784	488.1793	-1.81	1359	4.50	4.64	(M+H)+	1
508.1534	508.1554	-3.87	49310	100.00	100.00	(M+Na)+	1
509.1571	509.1587	-3.16	12408	25.16	25.81	(M+Na)+	1
510.1600	510.1612	-2.36	2342	4.75	4.63	(M+Na)+	1
524.1298	524.1293	0.96	694	100.00	100.00	(M+K)+	1
525.1235	525.1326	-17.43	153	22.07	25.82	(M+K)+	1

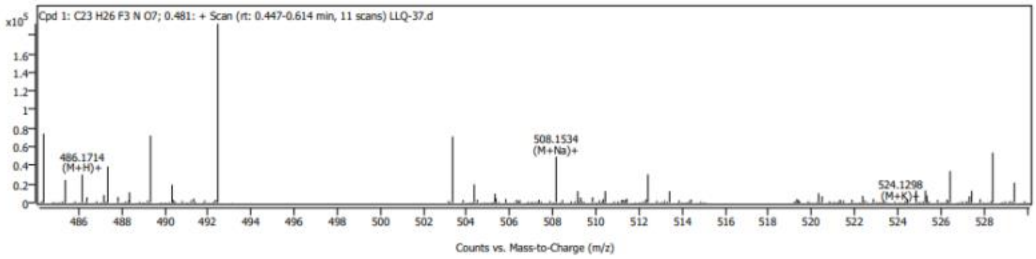

Spectrum Peaks

m/z	m/z (Calc)	Diff (ppm)	Abund	Height %	Height % (Calc)	Ion Species	Z
486.1714	486.1734	-4.18	30213	100.00	100.00	(M+H)+	1
487.1751	487.1767	-3.42	8180	27.07	25.82	(M+H)+	1
488.1784	488.1793	-1.81	1359	4.50	4.64	(M+H)+	1
508.1534	508.1554	-3.87	49310	100.00	100.00	(M+Na)+	1
509.1571	509.1587	-3.16	12408	25.16	25.81	(M+Na)+	1
510.1600	510.1612	-2.36	2342	4.75	4.63	(M+Na)+	1
524.1298	524.1293	0.96	694	100.00	100.00	(M+K)+	1
525.1235	525.1326	-17.43	153	22.07	25.82	(M+K)+	1

Header
Data

MassHunter Qualitative Analysis | Page 1 of 1 | Generated at 2:46 AM on 1/11/2023

MS spectra of **5e**

Custom Workflow Report

Agilent | Trusted Answers

Compound Details

Cpd. 1: C22 H23 F4 N O6

Compound Spectra

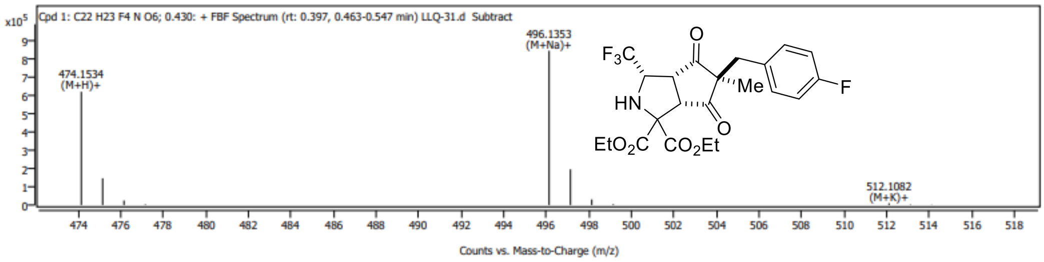

Spectrum Peaks

m/z	m/z (Calc)	Diff (ppm)	Abund	Height %	Height % (Calc)	Ion Species	Z
474.1534	474.1534	-0.05	621233	100.00	100.00	(M+H)+	1
475.1567	475.1567	-0.16	146644	23.61	24.66	(M+H)+	1
476.1590	476.1593	-0.72	24736	3.98	4.15	(M+H)+	1
477.1613	477.1620	-1.37	3595	0.58	0.52	(M+H)+	1
496.1353	496.1354	-0.10	846512	100.00	100.00	(M+Na)+	1
497.1387	497.1387	-0.01	195950	23.15	24.65	(M+Na)+	1
498.1408	498.1413	-1.02	30778	3.64	4.14	(M+Na)+	1
499.1505	499.1439	13.19	4712	0.56	0.52	(M+Na)+	1
512.1082	512.1093	-2.13	9629	100.00	100.00	(M+K)+	1
513.1130	513.1126	0.70	2841	29.50	24.67	(M+K)+	1
514.1099	514.1103	-0.65	1529	15.88	11.36	(M+K)+	1

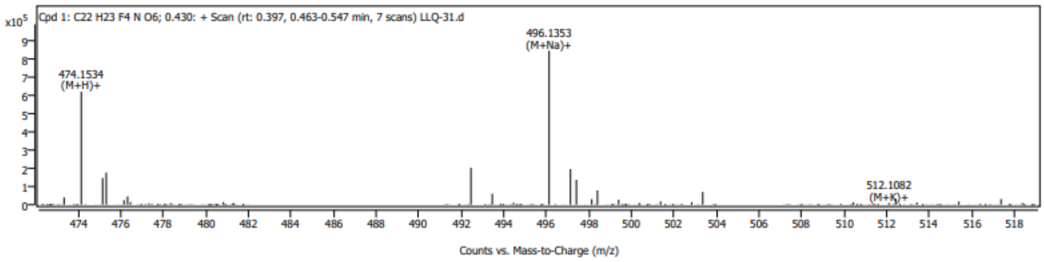

Spectrum Peaks

m/z	m/z (Calc)	Diff (ppm)	Abund	Height %	Height % (Calc)	Ion Species	Z
474.1534	474.1534	-0.05	621233	100.00	100.00	(M+H)+	1
475.1567	475.1567	-0.16	146644	23.61	24.66	(M+H)+	1
476.1590	476.1593	-0.72	24736	3.98	4.15	(M+H)+	1
477.1613	477.1620	-1.37	3595	0.58	0.52	(M+H)+	1
496.1353	496.1354	-0.10	846512	100.00	100.00	(M+Na)+	1
497.1387	497.1387	-0.01	195950	23.15	24.65	(M+Na)+	1
498.1408	498.1413	-1.02	30778	3.64	4.14	(M+Na)+	1
499.1505	499.1439	13.19	4712	0.56	0.52	(M+Na)+	1
512.1082	512.1093	-2.13	9629	100.00	100.00	(M+K)+	1
513.1130	513.1126	0.70	2841	29.50	24.67	(M+K)+	1
514.1099	514.1103	-0.65	1529	15.88	11.36	(M+K)+	1

MS spectra of **5f**

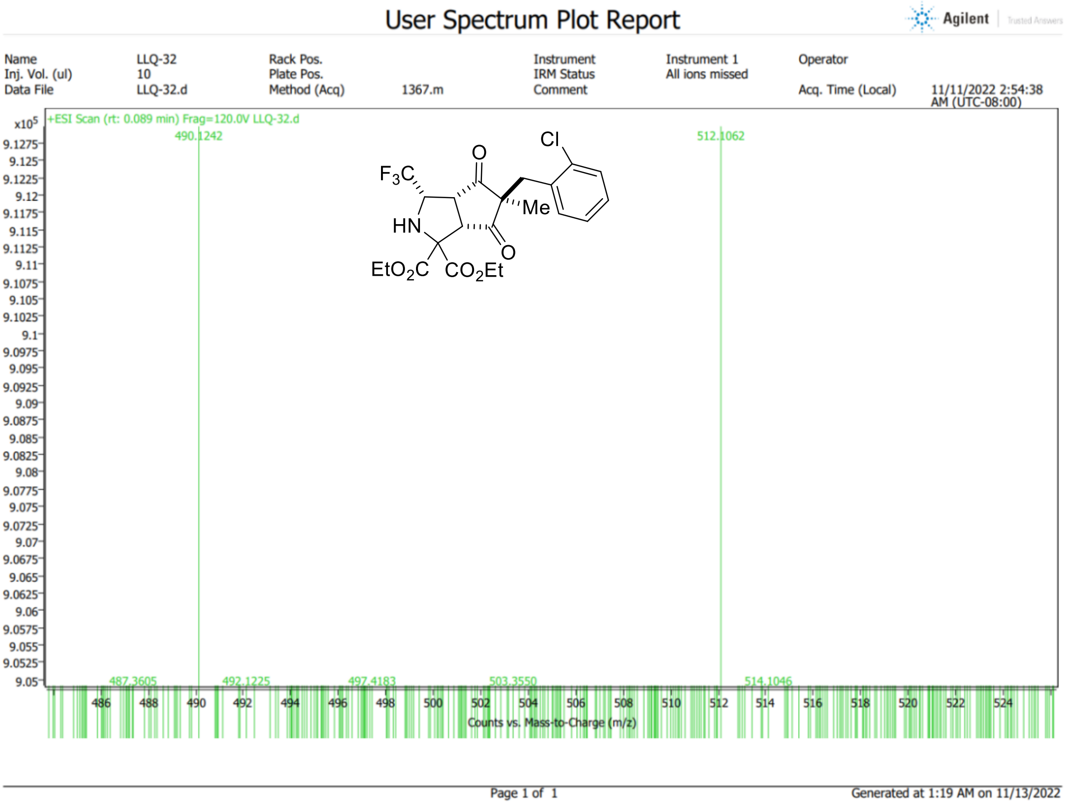

MS spectra of **5g**

Custom Workflow Report

Agilent Trusted Answers

Compound Details

Cpd. 1: C22 H23 Br F3 N O6

Compound Spectra

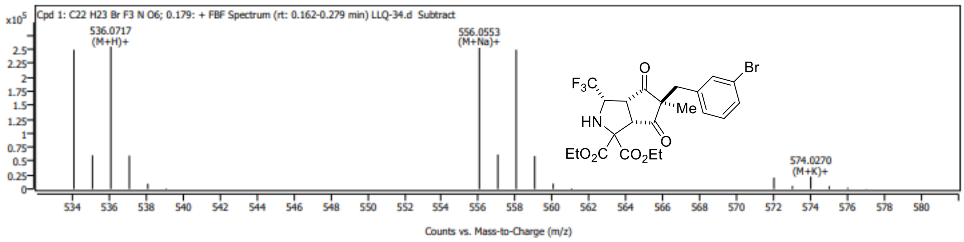

Spectrum Peaks

m/z	m/z (Calc)	Diff (ppm)	Abund	Height %	Height % (Calc)	Ion Species	Z
534.0732	534.0734	-0.23	249547	97.87	98.60	(M+H)+	1
535.0763	535.0767	-0.72	60417	23.69	24.32	(M+H)+	1
536.0717	536.0716	0.05	254979	100.00	100.00	(M+H)+	1
537.0745	537.0748	-0.50	59885	23.49	24.17	(M+H)+	1
538.0768	538.0773	-0.92	9460	3.71	4.03	(M+H)+	1
539.0788	539.0799	-2.09	1325	0.52	0.51	(M+H)+	1
556.0553	556.0553	0.03	252877	100.00	98.60	(M+Na)+	1
557.0583	557.0586	-0.63	61675	24.39	24.31	(M+Na)+	1
558.0535	558.0536	-0.22	249600	98.70	100.00	(M+Na)+	1
559.0565	559.0567	-0.39	59086	23.37	24.16	(M+Na)+	1
560.0593	560.0593	0.01	9815	3.88	4.03	(M+Na)+	1
561.0622	561.0619	0.53	1481	0.59	0.51	(M+Na)+	1
572.0287	572.0292	-0.88	20300	91.59	92.05	(M+K)+	1
573.0317	573.0325	-1.49	5399	24.36	22.70	(M+K)+	1
574.0270	574.0275	-0.83	22163	100.00	100.00	(M+K)+	1
575.0300	575.0307	-1.05	5016	22.63	24.20	(M+K)+	1
576.0291	576.0283	1.40	2519	11.37	10.50	(M+K)+	1
577.0370	577.0303	11.47	454	2.05	2.10	(M+K)+	1

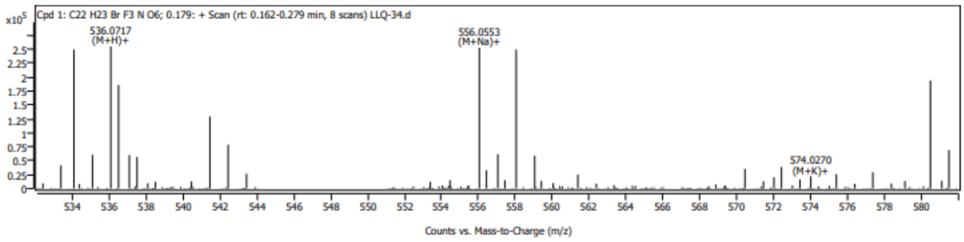

Spectrum Peaks

m/z	m/z (Calc)	Diff (ppm)	Abund	Height %	Height % (Calc)	Ion Species	Z
534.0732	534.0734	-0.23	249547	97.87	98.60	(M+H)+	1
535.0763	535.0767	-0.72	60417	23.69	24.32	(M+H)+	1
536.0717	536.0716	0.05	254979	100.00	100.00	(M+H)+	1
537.0745	537.0748	-0.50	59885	23.49	24.17	(M+H)+	1
538.0768	538.0773	-0.92	9460	3.71	4.03	(M+H)+	1
539.0788	539.0799	-2.09	1325	0.52	0.51	(M+H)+	1
556.0553	556.0553	0.03	252877	100.00	98.60	(M+Na)+	1
557.0583	557.0586	-0.63	61675	24.39	24.31	(M+Na)+	1
558.0535	558.0536	-0.22	249600	98.70	100.00	(M+Na)+	1
559.0565	559.0567	-0.39	59086	23.37	24.16	(M+Na)+	1
560.0593	560.0593	0.01	9815	3.88	4.03	(M+Na)+	1
561.0622	561.0619	0.53	1481	0.59	0.51	(M+Na)+	1
572.0287	572.0292	-0.88	20300	91.59	92.05	(M+K)+	1
573.0317	573.0325	-1.49	5399	24.36	22.70	(M+K)+	1
574.0270	574.0275	-0.83	22163	100.00	100.00	(M+K)+	1
575.0300	575.0307	-1.05	5016	22.63	24.20	(M+K)+	1
576.0291	576.0283	1.40	2519	11.37	10.50	(M+K)+	1
577.0370	577.0303	11.47	454	2.05	2.10	(M+K)+	1

MS spectra of **5h**

Custom Workflow Report

Agilent | Trusted Answers

Compound Details

Cpd. 1: C26 H26 F3 N O6

Compound Spectra

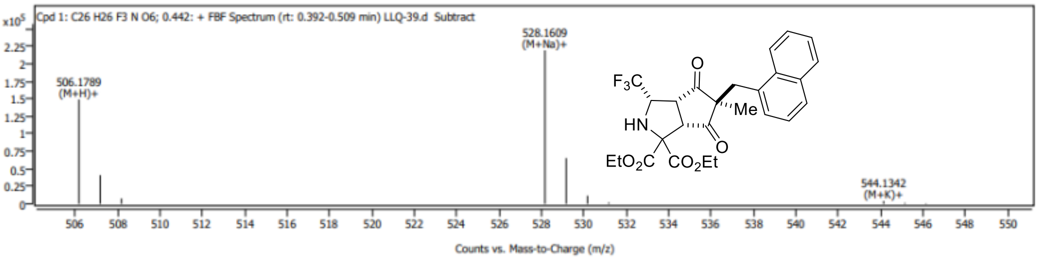

Spectrum Peaks

m/z	m/z (Calc)	Diff (ppm)	Abund	Height %	Height % (Calc)	Ion Species	Z
506.1789	506.1785	0.84	149107	100.00	100.00	(M+H)+	1
507.1818	507.1818	0.02	40963	27.47	29.03	(M+H)+	1
508.1846	508.1846	0.11	7736	5.19	5.29	(M+H)+	1
528.1609	528.1604	0.80	219260	100.00	100.00	(M+Na)+	1
529.1640	529.1638	0.54	65329	29.80	29.01	(M+Na)+	1
530.1671	530.1665	1.03	11653	5.31	5.29	(M+Na)+	1
531.1723	531.1692	5.92	1940	0.88	0.72	(M+Na)+	1
544.1342	544.1344	-0.34	3950	100.00	100.00	(M+K)+	1
545.1378	545.1377	0.21	1465	37.08	29.03	(M+K)+	1
546.1329	546.1359	-5.51	895	22.66	12.51	(M+K)+	1

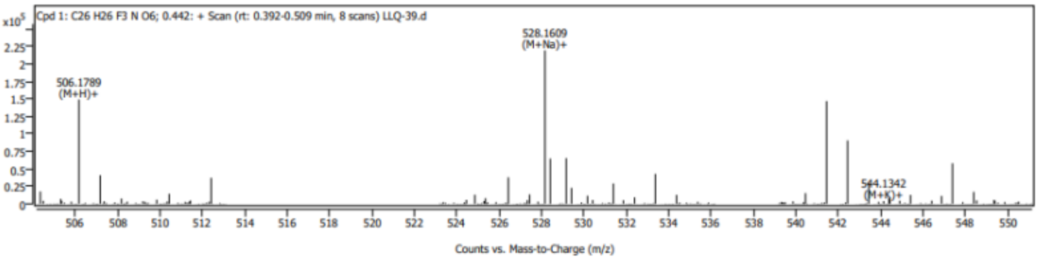

Spectrum Peaks

m/z	m/z (Calc)	Diff (ppm)	Abund	Height %	Height % (Calc)	Ion Species	Z
506.1789	506.1785	0.84	149107	100.00	100.00	(M+H)+	1
507.1818	507.1818	0.02	40963	27.47	29.03	(M+H)+	1
508.1846	508.1846	0.11	7736	5.19	5.29	(M+H)+	1
528.1609	528.1604	0.80	219260	100.00	100.00	(M+Na)+	1
529.1640	529.1638	0.54	65329	29.80	29.01	(M+Na)+	1
530.1671	530.1665	1.03	11653	5.31	5.29	(M+Na)+	1
531.1723	531.1692	5.92	1940	0.88	0.72	(M+Na)+	1
544.1342	544.1344	-0.34	3950	100.00	100.00	(M+K)+	1
545.1378	545.1377	0.21	1465	37.08	29.03	(M+K)+	1
546.1329	546.1359	-5.51	895	22.66	12.51	(M+K)+	1

MS spectra of **5i**

Custom Workflow Report

Agilent Trusted Answers

Compound Details

Cpd. 1: C26 H26 F3 N O6

Compound Spectra

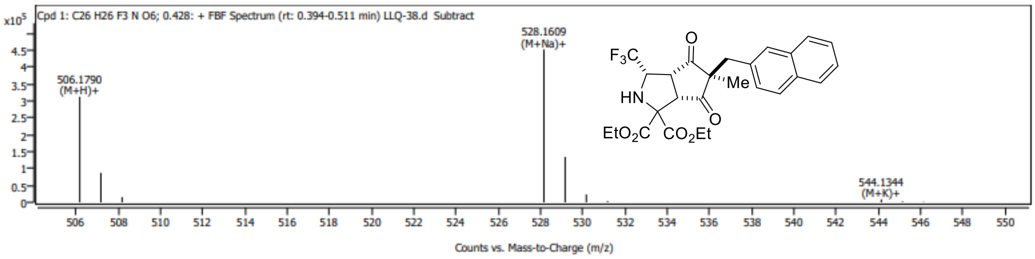

Spectrum Peaks

m/z	m/z (Calc)	Diff (ppm)	Abund	Height %	Height % (Calc)	Ion Species	Z
506.1790	506.1785	0.94	312723	100.00	100.00	(M+H)+	1
507.1818	507.1818	-0.01	87332	27.93	29.03	(M+H)+	1
508.1846	508.1846	0.01	15353	4.91	5.29	(M+H)+	1
528.1609	528.1604	0.87	453006	100.00	100.00	(M+Na)+	1
529.1639	529.1638	0.30	134582	29.71	29.01	(M+Na)+	1
530.1668	530.1665	0.56	23509	5.19	5.29	(M+Na)+	1
531.1711	531.1692	3.50	3579	0.79	0.72	(M+Na)+	1
544.1344	544.1344	-0.05	8885	100.00	100.00	(M+K)+	1
545.1373	545.1377	-0.76	2667	30.02	29.03	(M+K)+	1
546.1365	546.1359	1.18	1417	15.95	12.51	(M+K)+	1

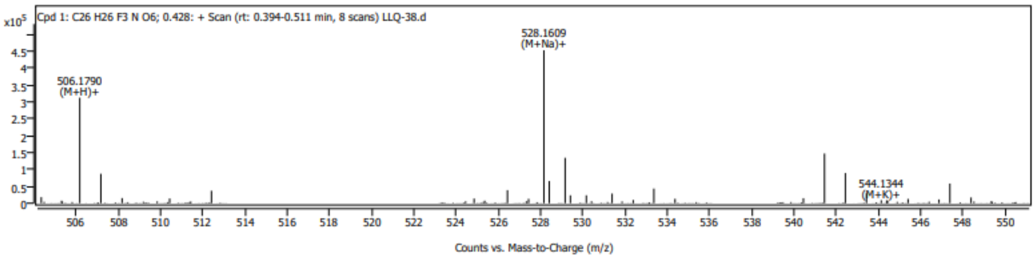

Spectrum Peaks

m/z	m/z (Calc)	Diff (ppm)	Abund	Height %	Height % (Calc)	Ion Species	Z
506.1790	506.1785	0.94	312723	100.00	100.00	(M+H)+	1
507.1818	507.1818	-0.01	87332	27.93	29.03	(M+H)+	1
508.1846	508.1846	0.01	15353	4.91	5.29	(M+H)+	1
528.1609	528.1604	0.87	453006	100.00	100.00	(M+Na)+	1
529.1639	529.1638	0.30	134582	29.71	29.01	(M+Na)+	1
530.1668	530.1665	0.56	23509	5.19	5.29	(M+Na)+	1
531.1711	531.1692	3.50	3579	0.79	0.72	(M+Na)+	1
544.1344	544.1344	-0.05	8885	100.00	100.00	(M+K)+	1
545.1373	545.1377	-0.76	2667	30.02	29.03	(M+K)+	1
546.1365	546.1359	1.18	1417	15.95	12.51	(M+K)+	1

MS spectra of **6**

Custom Workflow Report

Agilent | Trusted Answers

Compound Details

Cpd. 1: C30 H27 F3 N2 O3

Compound Spectra

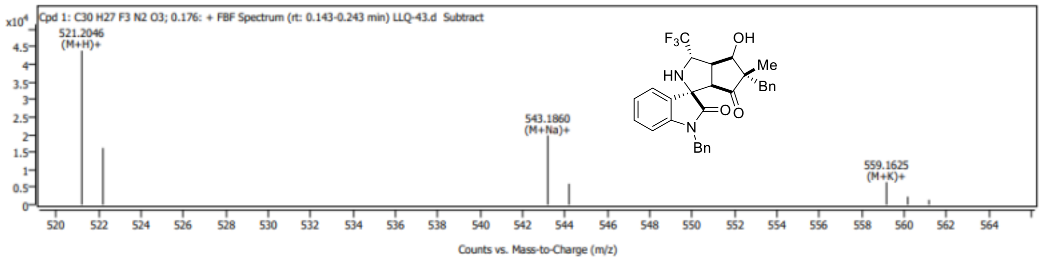

Spectrum Peaks

m/z	m/z (Calc)	Diff (ppm)	Abund	Height %	Height % (Calc)	Ion Species	Z
521.2046	521.2047	-0.09	43941	100.00	100.00	(M+H)+	1
522.2081	522.2079	0.37	16182	36.83	33.61	(M+H)+	1
543.1860	543.1866	-1.18	19771	100.00	100.00	(M+Na)+	1
544.1895	544.1898	-0.59	5979	30.24	33.60	(M+Na)+	1
559.1625	559.1605	3.45	6441	100.00	100.00	(M+K)+	1
560.1669	560.1638	5.49	2305	35.79	33.62	(M+K)+	1
561.1661	561.1624	6.61	1391	21.60	13.31	(M+K)+	1

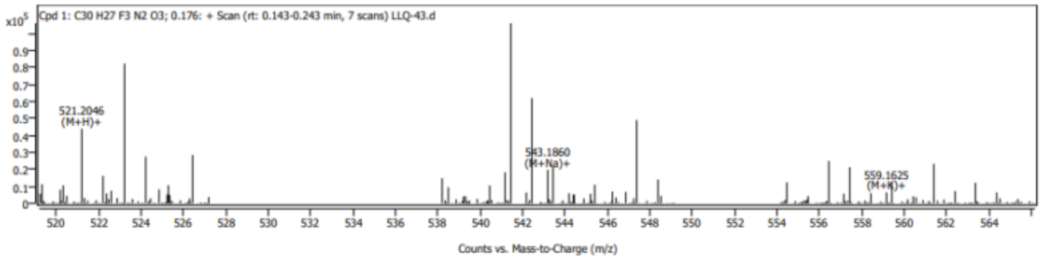

Spectrum Peaks

m/z	m/z (Calc)	Diff (ppm)	Abund	Height %	Height % (Calc)	Ion Species	Z
521.2046	521.2047	-0.09	43941	100.00	100.00	(M+H)+	1
522.2081	522.2079	0.37	16182	36.83	33.61	(M+H)+	1
543.1860	543.1866	-1.18	19771	100.00	100.00	(M+Na)+	1
544.1895	544.1898	-0.59	5979	30.24	33.60	(M+Na)+	1
559.1625	559.1605	3.45	6441	100.00	100.00	(M+K)+	1
560.1669	560.1638	5.49	2305	35.79	33.62	(M+K)+	1
561.1661	561.1624	6.61	1391	21.60	13.31	(M+K)+	1

Header
Data

MS spectra of **7**

Custom Workflow Report

Agilent Trusted Answers

Compound Details

Cpd. 1: C30 H25 F3 N2 O4

Compound Spectra

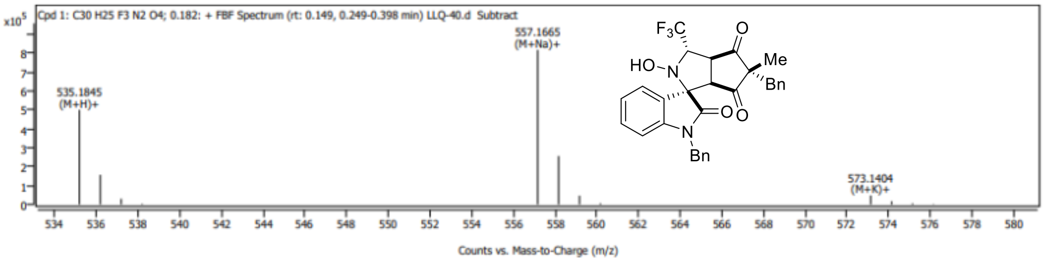

Spectrum Peaks

m/z	m/z (Calc)	Diff (ppm)	Abund	Height %	Height % (Calc)	Ion Species	Z
535.1845	535.1839	1.11	499625	100.00	100.00	(M+H)+	1
536.1876	536.1872	0.78	156286	31.28	33.63	(M+H)+	1
537.1906	537.1901	0.81	29855	5.98	6.30	(M+H)+	1
538.1934	538.1929	0.89	3873	0.78	0.85	(M+H)+	1
557.1665	557.1659	1.07	816319	100.00	100.00	(M+Na)+	1
558.1697	558.1691	1.10	255837	31.34	33.62	(M+Na)+	1
559.1724	559.1721	0.67	46112	5.65	6.30	(M+Na)+	1
560.1755	560.1749	1.09	6473	0.79	0.85	(M+Na)+	1
573.1404	573.1398	1.10	47321	100.00	100.00	(M+K)+	1
574.1441	574.1430	1.88	16274	34.39	33.63	(M+K)+	1
575.1437	575.1417	3.42	6286	13.28	13.52	(M+K)+	1
576.1480	576.1431	8.49	2228	4.71	3.28	(M+K)+	1

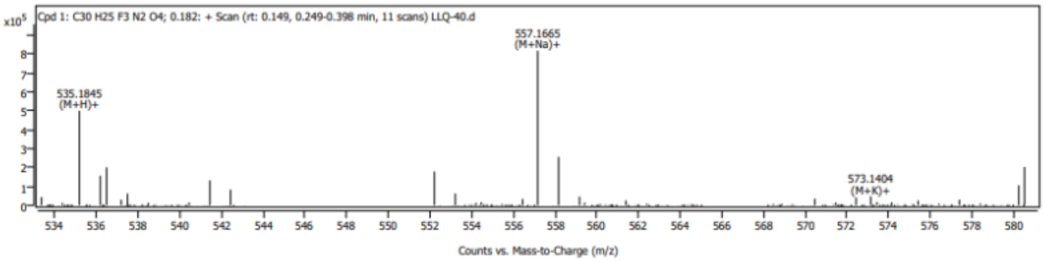

Spectrum Peaks

m/z	m/z (Calc)	Diff (ppm)	Abund	Height %	Height % (Calc)	Ion Species	Z
535.1845	535.1839	1.11	499625	100.00	100.00	(M+H)+	1
536.1876	536.1872	0.78	156286	31.28	33.63	(M+H)+	1
537.1906	537.1901	0.81	29855	5.98	6.30	(M+H)+	1
538.1934	538.1929	0.89	3873	0.78	0.85	(M+H)+	1
557.1665	557.1659	1.07	816319	100.00	100.00	(M+Na)+	1
558.1697	558.1691	1.10	255837	31.34	33.62	(M+Na)+	1
559.1724	559.1721	0.67	46112	5.65	6.30	(M+Na)+	1
560.1755	560.1749	1.09	6473	0.79	0.85	(M+Na)+	1
573.1404	573.1398	1.10	47321	100.00	100.00	(M+K)+	1
574.1441	574.1430	1.88	16274	34.39	33.63	(M+K)+	1
575.1437	575.1417	3.42	6286	13.28	13.52	(M+K)+	1
576.1480	576.1431	8.49	2228	4.71	3.28	(M+K)+	1

MS spectra of **8**

Custom Workflow Report

Compound Details

Cpd. 1: C32 H29 F3 N2 O4

Compound Spectra

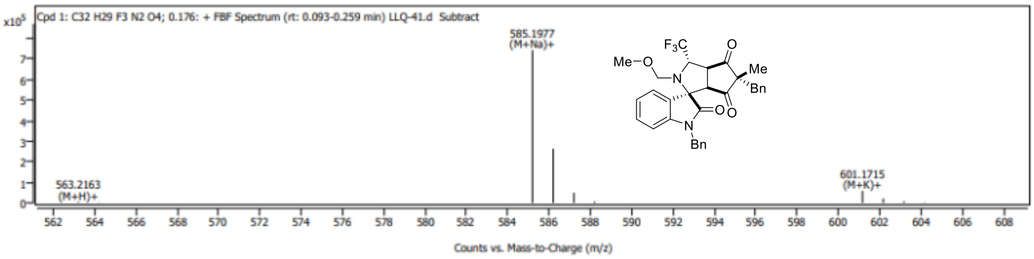

Spectrum Peaks

m/z	m/z (Calc)	Diff (ppm)	Abund	Height %	Height % (Calc)	Ion Species	Z
563.2163	563.2152	1.90	4693	100.00	100.00	(M+H)+	1
564.2198	564.2185	2.28	1732	36.91	35.84	(M+H)+	1
585.1977	585.1972	0.93	743671	100.00	100.00	(M+Na)+	1
586.2011	586.2004	1.20	263711	35.46	35.83	(M+Na)+	1
587.2037	587.2034	0.47	49381	6.64	7.05	(M+Na)+	1
588.2062	588.2063	-0.05	6684	0.90	0.99	(M+Na)+	1
601.1715	601.1711	0.72	58570	100.00	100.00	(M+K)+	1
602.1751	602.1744	1.23	21619	36.91	35.84	(M+K)+	1
603.1754	603.1732	3.57	7725	13.19	14.27	(M+K)+	1
604.1790	604.1746	7.23	2286	3.90	3.58	(M+K)+	1

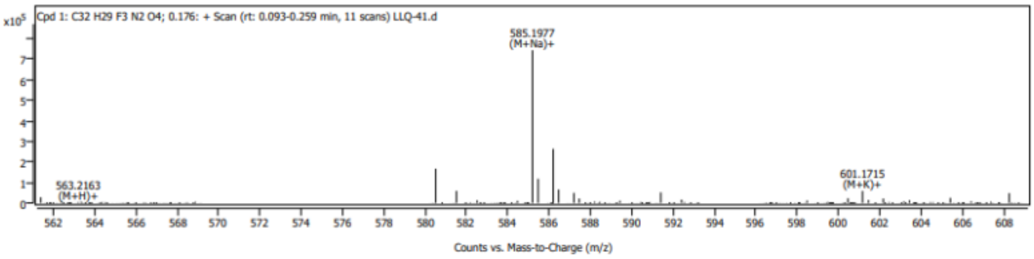

Spectrum Peaks

m/z	m/z (Calc)	Diff (ppm)	Abund	Height %	Height % (Calc)	Ion Species	Z
563.2163	563.2152	1.90	4693	100.00	100.00	(M+H)+	1
564.2198	564.2185	2.28	1732	36.91	35.84	(M+H)+	1
585.1977	585.1972	0.93	743671	100.00	100.00	(M+Na)+	1
586.2011	586.2004	1.20	263711	35.46	35.83	(M+Na)+	1
587.2037	587.2034	0.47	49381	6.64	7.05	(M+Na)+	1
588.2062	588.2063	-0.05	6684	0.90	0.99	(M+Na)+	1
601.1715	601.1711	0.72	58570	100.00	100.00	(M+K)+	1
602.1751	602.1744	1.23	21619	36.91	35.84	(M+K)+	1
603.1754	603.1732	3.57	7725	13.19	14.27	(M+K)+	1
604.1790	604.1746	7.23	2286	3.90	3.58	(M+K)+	1

Header
Data

MS spectra of **9**

Custom Workflow Report

Compound Details

Cpd. 1: C36 H29 F3 N2 O3

Compound Spectra

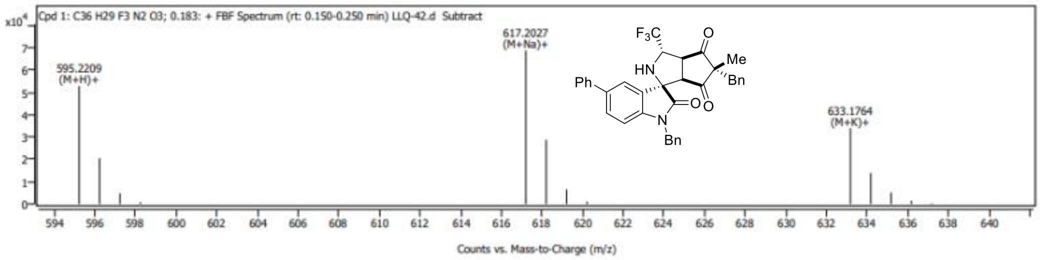

Spectrum Peaks

m/z	m/z (Calc)	Diff (ppm)	Abund	Height %	Height % (Calc)	Ion Species	Z
595.2209	595.2203	0.94	52887	100.00	100.00	(M+H)+	1
596.2241	596.2236	0.87	20525	38.81	40.13	(M+H)+	1
597.2284	597.2267	2.89	4818	9.11	8.46	(M+H)+	1
598.2320	598.2296	4.00	838	1.58	1.24	(M+H)+	1
617.2027	617.2022	0.78	68885	100.00	100.00	(M+Na)+	1
618.2062	618.2055	1.03	28814	41.83	40.12	(M+Na)+	1
619.2099	619.2086	2.06	6634	9.63	8.45	(M+Na)+	1
620.2156	620.2116	6.47	1132	1.64	1.24	(M+Na)+	1
633.1764	633.1762	0.39	34057	100.00	100.00	(M+K)+	1
634.1800	634.1795	0.91	13924	40.88	40.13	(M+K)+	1
635.1800	635.1788	1.92	5160	15.15	15.67	(M+K)+	1
636.1814	636.1800	2.27	1511	4.44	4.14	(M+K)+	1
637.1883	637.1821	9.69	385	1.13	0.75	(M+K)+	1

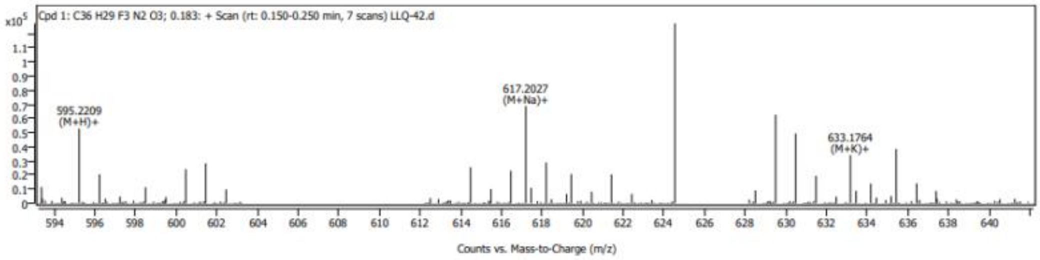

Spectrum Peaks

m/z	m/z (Calc)	Diff (ppm)	Abund	Height %	Height % (Calc)	Ion Species	Z
595.2209	595.2203	0.94	52887	100.00	100.00	(M+H)+	1
596.2241	596.2236	0.87	20525	38.81	40.13	(M+H)+	1
597.2284	597.2267	2.89	4818	9.11	8.46	(M+H)+	1
598.2320	598.2296	4.00	838	1.58	1.24	(M+H)+	1
617.2027	617.2022	0.78	68885	100.00	100.00	(M+Na)+	1
618.2062	618.2055	1.03	28814	41.83	40.12	(M+Na)+	1
619.2099	619.2086	2.06	6634	9.63	8.45	(M+Na)+	1
620.2156	620.2116	6.47	1132	1.64	1.24	(M+Na)+	1
633.1764	633.1762	0.39	34057	100.00	100.00	(M+K)+	1
634.1800	634.1795	0.91	13924	40.88	40.13	(M+K)+	1
635.1800	635.1788	1.92	5160	15.15	15.67	(M+K)+	1
636.1814	636.1800	2.27	1511	4.44	4.14	(M+K)+	1
637.1883	637.1821	9.69	385	1.13	0.75	(M+K)+	1

Header
Data
